# Implications of Climate Change for Bird Conservation in the Southwestern U.S. under Three Alternative Futures

**DOI:** 10.1371/journal.pone.0144089

**Published:** 2015-12-23

**Authors:** Megan M. Friggens, Deborah M. Finch

**Affiliations:** United States Forest Service, Rocky Mountain Research Station, Albuquerque, New Mexico, United States of America; Università degli Studi di Napoli Federico II, ITALY

## Abstract

Future expected changes in climate and human activity threaten many riparian habitats, particularly in the southwestern U.S. Using Maximum Entropy (MaxEnt3.3.3) modeling, we characterized habitat relationships and generated spatial predictions of habitat suitability for the Lucy’s warbler (*Oreothlypis luciae*), the Southwestern willow flycatcher (*Empidonax traillii extimus*) and the Western yellow-billed cuckoo (*Coccyzus americanus*). Our goal was to provide site- and species-specific information that can be used by managers to identify areas for habitat conservation and/or restoration along the Rio Grande in New Mexico. We created models of suitable habitat for each species based on collection and survey samples and climate, biophysical, and vegetation data. We projected habitat suitability under future climates by applying these models to conditions generated from three climate models for 2030, 2060 and 2090. By comparing current and future distributions, we identified how habitats are likely to change as a result of changing climate and the consequences of those changes for these bird species. We also examined whether land ownership of high value sites shifts under changing climate conditions. Habitat suitability models performed well. Biophysical characteristics were more important that climate conditions for predicting habitat suitability with distance to water being the single most important predictor. Climate, though less important, was still influential and led to declines of suitable habitat of more than 60% by 2090. For all species, suitable habitat tended to shrink over time within the study area leaving a few core areas of high importance. Overall, climate changes will increase habitat fragmentation and reduce breeding habitat patch size. The best strategy for conserving bird species within the Rio Grande will include measures to maintain and restore critical habitat refugia. This study provides an example of a presence-only habitat model that can be used to inform the management of species at intermediate scales.

## Introduction

Wildlife managers face unprecedented challenges with increasing pressures from land degradation and loss and climate change. Measures to conserve species must increasingly consider land conversion, population growth, invasive species, and disrupted disturbance regimes all within a complicated matrix of mixed land ownership and management priorities. Climate change, human population growth, development of floodplains, and grazing are the primary drivers of habitat degradation across southwestern riparian habitats [1, 2]. Declines in riparian forests, or bosques, threatened many neotropical migrant birds which rely on these areas as important stopover sites and breeding habitat [3]. Birds have been identified as highly vulnerable to climate impacts and substantial shifts in their geographic range are expected to result in an overall decline in species richness [4, 5]. The degree to which birds will be negatively impacted by climate change increases with increasing reliance on specialized or limited habitats for breeding and stopover sites [5]. Thus, many of the neotropical migrants that rely on dwindling southwestern riparian habitats are likely to be highly vulnerable to climate impacts [3, 6].

Climate change predictions for the southwestern U.S., which include increasing air and water temperatures, longer and more severe drought and reduced snowpack are likely to exacerbate water shortages that lead to declines in riparian habitat. These changes will worsen the issues faced by many southwestern species including the endangered southwestern willow flycatcher and the threatened yellow-billed cuckoo [1, 7, 8]. The future of riparian associated species is tenuous and we cannot delay actions that help preserve these habitats and species. However, management and conservation of riparian species is confounded by the complexity of their habitats, which regularly undergo reorganization due to changing water levels, drought, fire and other disturbances [2], and the multijurisdictional nature of river systems. Though many interagency programs exist to plan across jurisdiction boundary (e.g. Middle Rio Grande Endangered species Collaborative (MRGESACP), Upper Colorado River Endangered Fish Recover Program [9]) climate related changes in species and habitat distributions could change the geographic relevance of current conservation and restoration activities in unpredictable ways. Therefore, managers need to know how and where species’ habitat change is likely and be able to evaluate these in context of land use and management.

**Fig 1:**
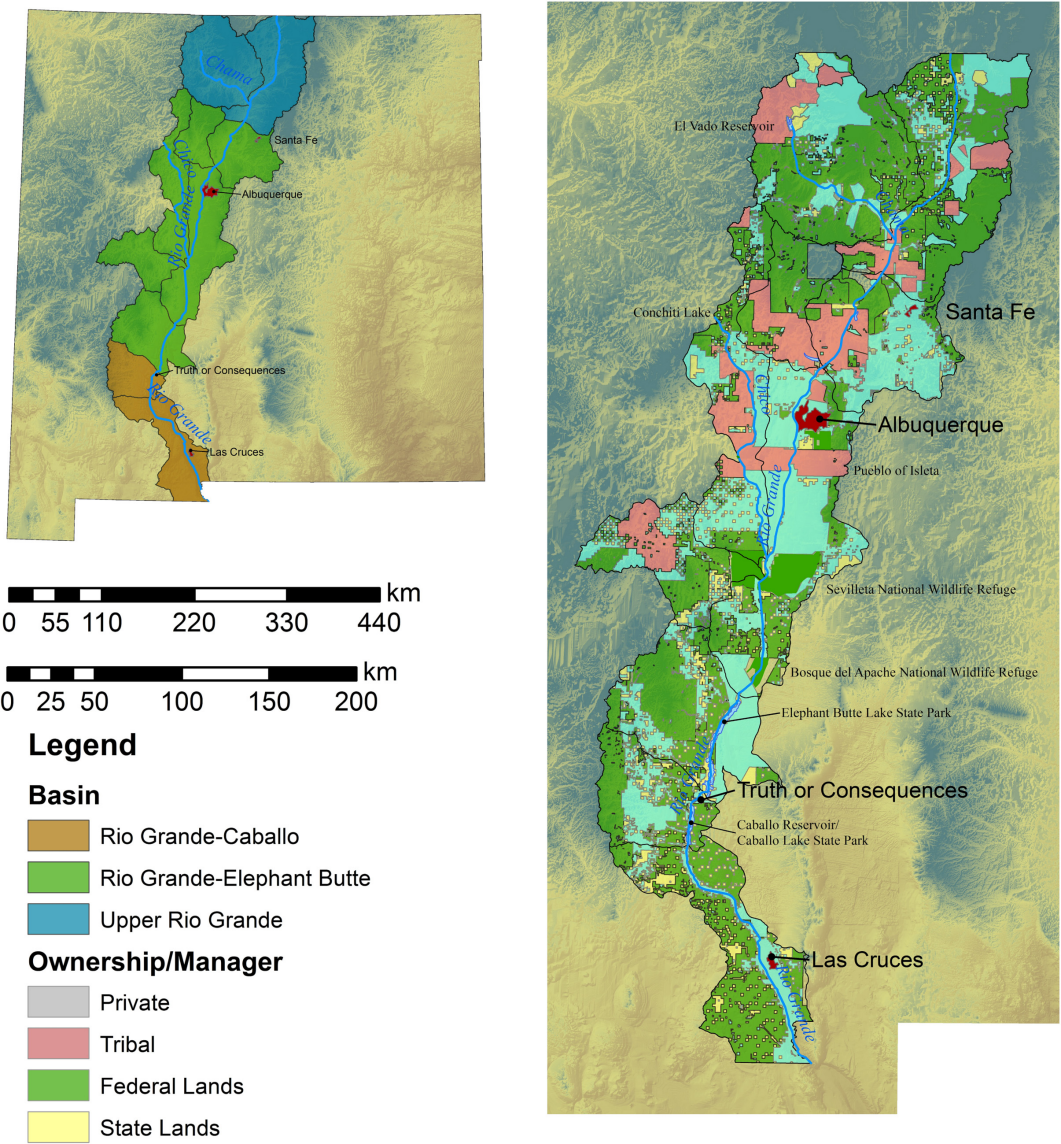
Study site showing major basins and land ownership patterns.

As a first step for planning for species conservation under changing climate, we assess the potential climate impacts on habitat for three bird species that breed within riparian habitats along the Rio Grande (Fig 1). Our objective is to identify the future suitability of sites along the Rio Grande for conservation and restoration [10]. The Rio Grande is a critical source of water and habitat for wildlife and human populations [11–13] and was considered the most endangered river in America by the national conservation group American Rivers in 1993 [14]. More recently, the Rio Grande was designated one of the top 10 rivers at risk in the world by the World Wildlife Federation [15]. The Rio Grande bosque is important for multiple riparian obligate species including the endangered southwestern willow flycatcher (*Empidonax traillii extimus*) and New Mexico meadow jumping mouse (*Zapus luteus*). Demand for water has altered flows and lowered water tables across the Southwest with negative impacts for riparian forest habitats and corresponding declines in species that rely upon them [7]. Invasive species such as tamarisk (*Tamarix* spp.) and Russian olive (*Elaeagnus angustifolia*) have also reduced natural bosque habitat with detrimental effects for many wildlife species [11]. Collectively, these impacts are estimated to have led to a decrease of important wetland habitats by as much as 93% since 1918, the greatest historical decline of any floodplain community [13, 14]. Climate change is expected to reduce flow levels and lead to an increased rate of loss of natural bosque habitats as well as potentially increasing invasive species like tamarisk [6].

We focus on three migratory bird species representing a diversity of life histories: the Lucy’s warbler (*Oreothlypis luciae*), the Southwestern willow flycatcher (*Empidonax traillii extimus*) and the Western yellow-billed cuckoo (*Coccyzus americanus*). Lucy’s warblers are small insect eating neotropical migrant species that breed within Arizona and New Mexico. They are a cavity nesting species [16, 17] that typically occur in lowland areas and are associated with a range of bosque habitats, but most commonly riparian mesquite habitats [16]. While little is known about population trends for Lucy’s warblers, local extirpations have been noted due to habitat loss, in particular mesquite [18], and cowbird parasitism. Recent declines in screwbean mesquite (*Prosopis pubescens*) along the warbler’s traditional breeding range may continue under changing climates [18]. However, of the species considered in this study, Lucy’s warblers have the most southern range and appear well suited to arid habitats [16]. In addition, Lucy’s warblers may also be able to utilize sites with tamarisk and are known to have expanded into tamarisk dominated habitats on the Colorado River in the Grand Canyon. The southwestern willow flycatcher is a small, insect-eating neotropical migrant that winters in central American and probably south America [19]. This species has been on the U.S. Endangered Species list since 1995, primarily due to loss of its preferred nesting habitats. The flycatcher nests exclusively in riparian habitats where it is strongly associated with dense understory vegetation, tending to build nests in flooded areas dominated by willow habitat [20, 21]. Climate related changes in bosque habitat and the duration of water flows are likely to negatively impact this species [6]. The Western yellow-billed cuckoo is also a neotropical migrant that nests exclusively within riparian habitats [22]. Like the flycatcher, the yellow-billed cuckoo is associated with riparian habitats with willow and cottonwood [22]. Cuckoo populations in New Mexico have declined precipitously since the 1960’s as humans have altered riparian habitats [23] and this species is considered sensitive to increasing drought and wildfire in New Mexico (Governor’s Task Force on Drought). Yellow-billed cuckoos were recently listed as threatened due to dramatic population declines over the last 30 years that may be directly related to climate impacts, particularly as they influence insect prey populations [8].

Ecological niche models (ENM) are an excellent method to identify site suitability with respect to conservation planning under climate change (e.g. assist with place based assessments [24]). ENMs can assist management of endangered species by identifying areas of high importance in current and future time periods as well as identify areas with shared suitability for multiple species and potential for refugia [25]. A number of models and methods have been applied to estimate bird response to climate change (e.g. [5, 26, 27]). Niche models in particular can provide important information on where and how bird species’ range may shift in response to climate changes [28] (though this idea is not supported by all [29] but see [30]). To assess the potential impacts of climate change for birds inhabiting the Rio Grande, we use maximum entropy methods (MaxEnt 3.3.3 [31]; http://www.cs.princeton.edu/~schapire/maxent/) to relate climate and biotic characteristics to species presence. MaxEnt has been used to estimate distribution of suitable habitat for many wildlife species [32–37] and to explore potential changes in suitable habitat as well as the importance of individual variables for predicting habitat suitability. Specifically, we ask: 1) Are these bird species likely to lose habitat under future climate regimes? And, 2) Will climate-related changes in habitat require new landscape planning strategies? Through our analysis we show that declines in suitable habitat are likely under both mild and extreme climate scenarios though each species appears to be sensitive to a unique set of conditions. Importantly, declines in suitable habitat can be characterized as contractions rather than latitudinal or elevational shifts, and we identify critical refugia that appear to remain suitable for these species through the end of the century.

## Methods

### Site Description

The study site is focused within three basins selected based on their proximity (within 50km) to the Rio Grande (Fig 1). Three primary streams drain into the Rio Grande within New Mexico: 1) the Rio Chama, the most significant tributary, 2) the Jemez River, and 3) the San Jose/Rio Puerco Drainage. The headwaters of the Rio Grande constitute the upper Rio Grande watershed and begin in Colorado where the river flows fast through a forest mountain landscape [38] constituting big sagebrush shrubland (20%), Pinyon Juniper woodland (16%), Ponderosa Pine woodland (12%), and various mixed conifer woodland types (13%). Further south, the Rio Grande-Santa Fe Watershed (HUC8 13020201) consists of Pinyon Juniper woodlands (35%), Juniper woodlands and savanna (16%), Semi-desert grasslands (16%), and other mixed conifer woodlands, riparian woodlands and shrublands and grassland habitats (remaining 12%). At Albuquerque, the river broadens and slows as it enters a landscape of sloping flood plains and grassland desert habitats [39, 40]. From Albuquerque, the Rio Grande flows another 515 km to the international boundary between New Mexico and Texas. Most of this section is contained within the El Paso-Las Cruces watershed (HUC8 13030102) and is characterized as a Chihuahuan desert habitat (33%), Chihuahuan semidesert grasslands (18%), dune and sand scrub (15%), and mesquite upland scrub (10%) [38].

Climate and Hydrology: Annual precipitation ranges from 1120 mm in the northern reach to less than 200 mm in the southern portion. Approximately 70% of precipitation that drives river flow derives from the northernmost part of the Rio Grande basin. The Rio Grande flow is characterized by a spring peak, generally between early April and mid-May, corresponding to spring snow melt followed by a lesser peak in late summer as monsoon storms provides additional precipitation. Fall and winter flows are generally lower [41]. The historic flow regime of the river has been greatly impacted by irrigation diversions and agricultural reservoirs in the lower part of the system [39, 42]. Most notably, irrigation activities have increased the relative magnitude and duration of summer peak flows while reducing flow levels associated with snowmelt fundamentally altering the phenology of the system and causing water table levels to decline. In addition, several modifications affect natural flow regimes, including the Rio Grande Reservoir in the headwaters, Cochiti Reservoir about 80 miles north of Albuquerque, and Elephant Butte and Caballo Reservoirs south of Albuquerque.

### Species data

This study did not engage in new data collection. Location data for each bird species was obtained from museum records and previous studies by the Rocky Mountain Research Station (RMRS) [43]. We downloaded museum records for observations made within the study area during breeding season (June-September) and covering the period 1970–2013 through the ORNIS 2 Portal (ornis2.ornisnet.org). Museum collection information comes from a variety of sources that presumably were vetted for appropriate permit guidelines or were collected previous to these standards. We excluded records with a reported location inaccuracy greater than 500 meters. All data locations were converted to NAD1983. Where record datum was not defined, we assumed WGS84. In the event that an observation’s datum was misidentified (does not conform to our assumption that it is WGS84), a reported location may be misplaced by up to 500 meters. This margin of error was deemed acceptable since we are not concerned with site specific characteristics but rather shifts in the overall presence of suitable habitat, the modeling of which concerns relatively low resolution data (on the order of 1+ km).

We also used species presence data collected from survey work conducted by the RMRS in the 1990 and 2000’s [43]. Field site access for the point count surveys were provided by the Middle Rio Grande Conservancy District and the Bosque Del Apache National Wildlife Refuge [43, 44]. No animals were handled as part of these surveys and no animal use permits were required. Surveys were conducted along the Middle Rio Grande Valley from Albuquerque to the Bosque del Apache Wildlife Refuge (Fig 1) to assess the impacts of restoration on bird occupancy [45, 46]. Breeding bird point counts were conducted at 12 research sites. Within each site, eight point count stations were established on transects running along a north south gradient. Each station was sampled every other week for an average of 5 total samples from May 5 through August 15. At each point, observers recorded the species, sex, age and distance of any birds seen or heard in an eight minute period. Again, all data locations were converted to NAD1983. Collectively, museum and field data provided 63, 22, and 96 observations for Lucy’s warbler, Southwestern willow flycatcher, and Western yellow-billed cuckoo, respectively.

### Data Layers

#### Biophysical variables

Elevation layers were derived from 1 degree digital elevation model for Colorado, New Mexico, Utah and Arizona, from Data Basin (http://app.databasin.org) originally produced by the United States Geological Survey (USGS). We used the slope tool in ArcMap 10.1 to generate a slope data layer from the elevation data.

Creeks and river features were extracted from the National Hydrography Dataset -24k (gway_1933069_03_NHD24k). We created a vector file of all named and perennial creek and river features and merged it with a layer representing reservoirs and ponds greater than 0.2 km^2^ in area. We calculated Euclidean distance (ArcMap 10.1) in kilometers from the water features and used this to create a layer representing distance to water.

#### Habitat variables

**Fig 2:**
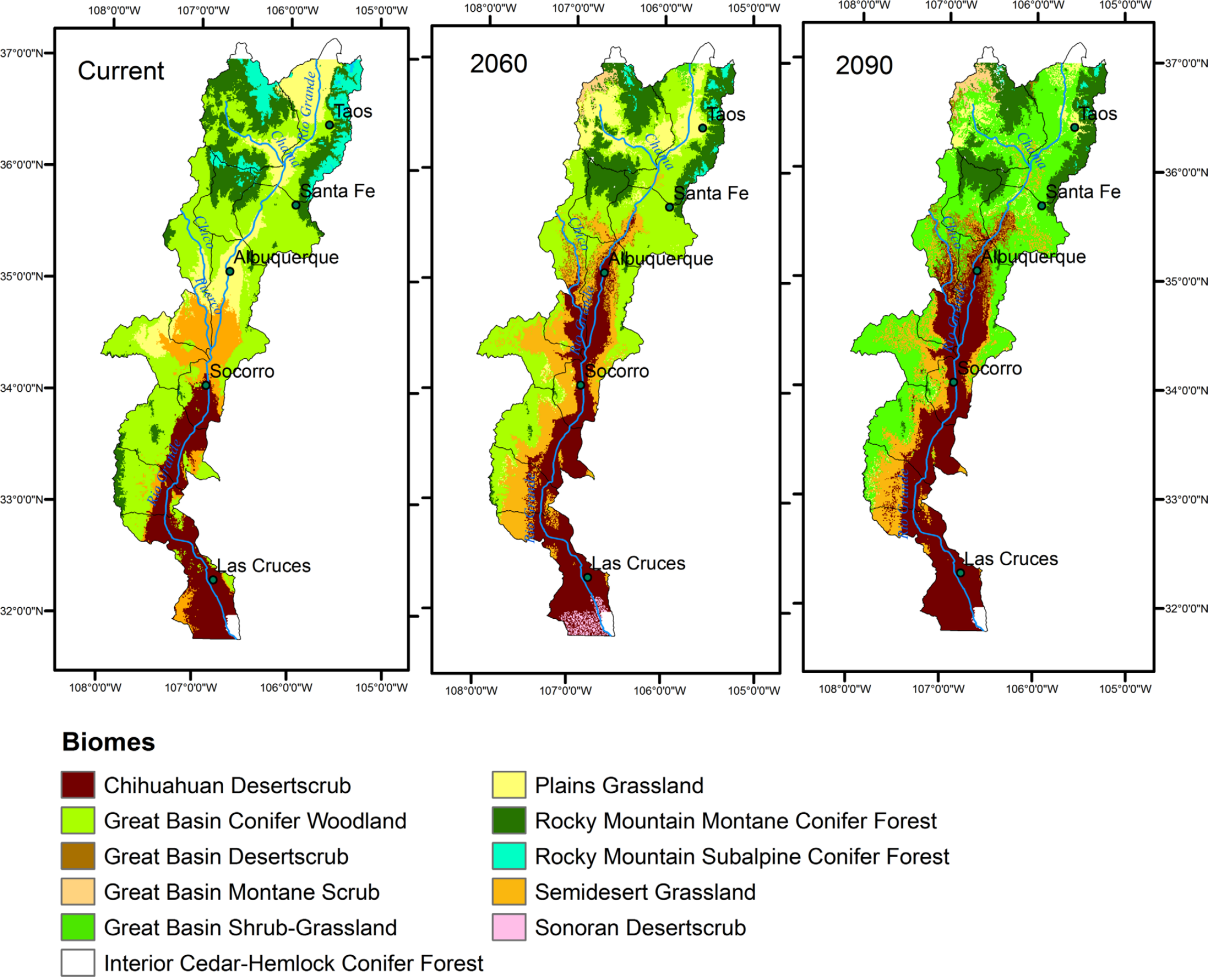
Rehfeldt (2006; 2012) projections for biomes (Brown, 1998) for three time periods in the study area within New Mexico. Projections were made for habitats under an A2 emission scenario.

Raster layers representing 25 biomes at four distinct time periods (current, 2030, 2060, and 2090) were downloaded from http://forest.moscowfsl.wsu.edu/climate/publications.php [47, 48]. Biome classifications and change over time are shown in Fig 2. Rehfeldt 2006 used random forests to predict distribution of biomes for contemporary and future (2030, 2060, 2090) time periods under three GCM and two emission scenarios. Projections were generated at a 1 km resolution. Final results were presented as a consensus raster where pixels were classified as a biome when at least 4 model runs supported that outcome.

#### Environmental Variables

Climate data used for this analysis is based upon the World Climate Research Programme's (WCRP's) Coupled Model Intercomparison Project phase 3 (CMIP3) multi-model dataset. We downloaded climate data based on three general circulation models (GCMs) under the A2 emission scenario from the downscaled CMIP3 and CMIP5 Climate and Hydrology Projections archive at http://gdo-dcp.ucllnl.org/downscaled_cmip_projections/ [49]. We selected GCMs to represent the range of predicted conditions that might occur within NM under a scenario of increasing emissions (See S1 Appendix). Data was downloaded for historic (1950–2013) and future (2020–2100) time periods. The models were: 1) Canadian Center for Climate Modeling and analysis (CCC) using the CGCM3.1 model, 2) Met office, Hadley Centre (HAD), using the HAdCM3 model, and (3) Geophysical Fluid Dynamics laboratory (GFD) using the CM2.1 model (See S1 Appendix). Data is provided at the resolution of 1/8 degree. We consider these GCMs to represent the range of scientific approaches used by climate modeling communities [50]. The SRESA2 emission scenario is considered the high emission pathway where technological change and economic growth is more fragmented and accompanied by slower, higher population growth (Emissions pathway descriptions and illustrations are available online at the IPCC Data Distribution Centre). At the time of this analysis, observed trends in CO_2_ and other emissions had exceeded estimates under the SRES A2 higher emission scenarios [51, 52].

We downloaded monthly values for two time periods, 1950–2013 and 2020–2100, for the following variables: Total precipitation (mm), mean maximum daily temperature (°C), mean minimum daily temperature (°C), soil moisture content (state 1st day of month, mm), snow water equivalent in snow pack (state 1st day of month, mm), stream flow (surface runoff + base flow (mm)), actual ET (mm), natural vegetation PET (mm), and open water surface PET (mm). From temperature and precipitation variables, we calculated 19 bioclimate variables [26](also see workslim.org/bioclim) commonly used for species distribution models for four time periods (1970–2013 “current”, 2020–2040 “2030”, 2050–2070 “2060”, and 2080–2100 “2090”) (R 3.0.1, SAS 9.3). In addition we calculated an aridity index (P/PET) where an AI<0.2 indicates arid/hyperarid and AI>0.5 indicating dry conditions. We then created a series of raster layers for each time period and each variable in ArcMap 10.1.

#### MaxEnt modeling

Maximum Entropy modeling was performed to provide an index of breeding habitat suitability [53]. We used the Maxent program (version 3.3.1 –see http://www.cs.princeton.edu/~schapire/maxent, see [31]). We used bilinear interpolation to resample climate, biome and distance to water layers to a pixel size of 0.0083° by 0.0083° (corresponding to elevation and slope layers). The processed climatic variables (at the original resolution), all resampled variables, and the occurrence localities are available upon request.

Maxent uses a maximum likelihood method to model species' distributions by generating a probability distribution over the pixels in a grid of the study area, where observed species presence are the sample points within the study area. The probability distribution is estimated to maximize entropy (i.e., that is the closest to uniform) under a set of constraints or variables assumed to represent suitable habitat values at species occurrence locations. MaxEnt measures how well the predicted distribution fits the sample points as compared to a uniform distribution, which is used to represent the probability distribution of the model (e.g. represents a measure of the likelihood of the samples). In this way, MaxEnt determines what characteristics of used areas are more suitable than those of a background sample [53].

**Fig 3:**
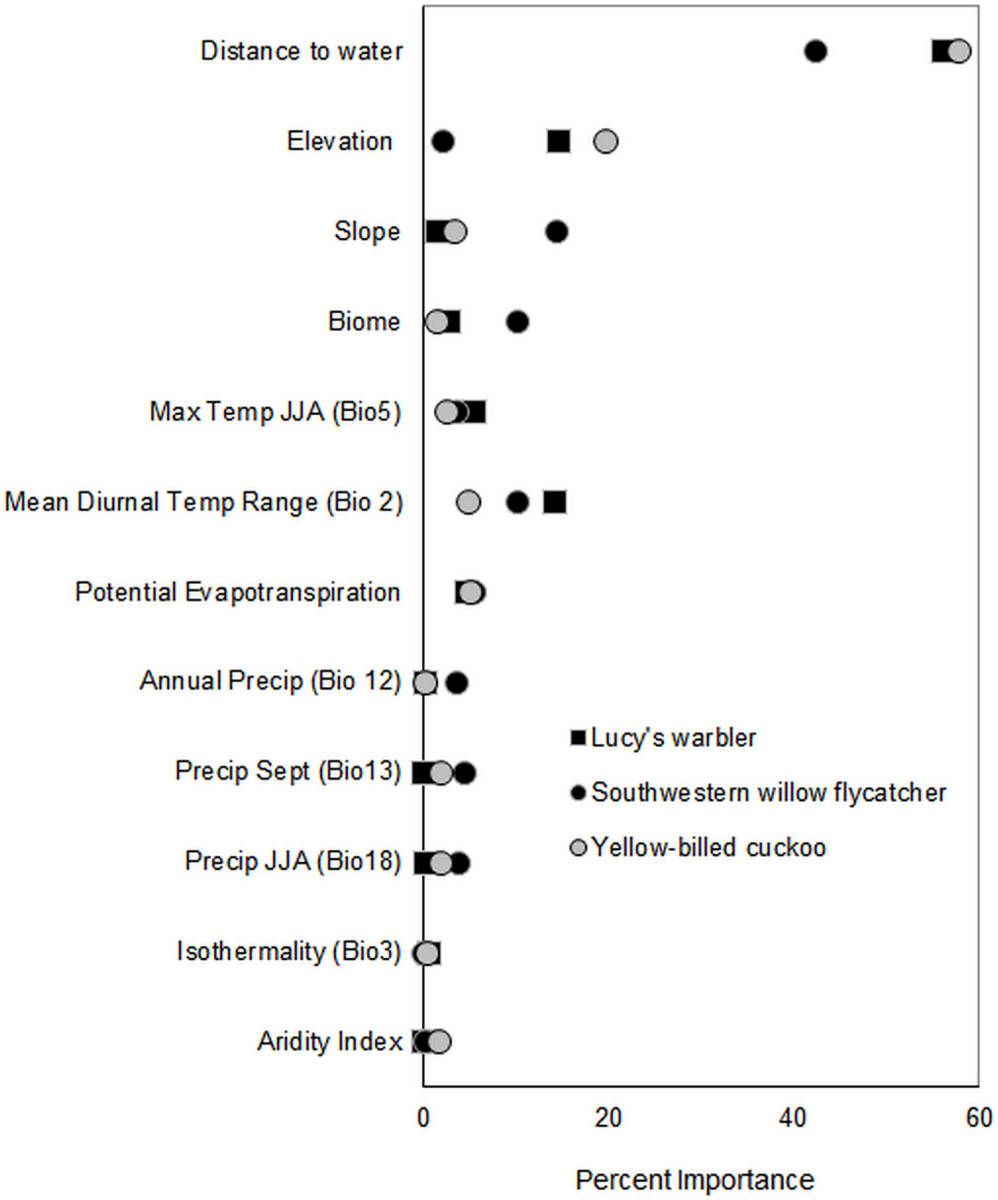
Variable importance for 12 predictors of suitable habitat for three bird species. Importance indicates contribution of variable to model fit.

Based on the literature and other modeling work [5, 26, 54] we selected variables we believed would be important for determining (or limiting) species distribution [55]. Important variables include those describing average and extreme temperature, annual and seasonal precipitation, climatic variability (e.g. Isothermality), and biophysical characteristics. Data analysis was generated using SAS/STAT software, Version 9.3 of the SAS System for Windows. Our final models included 12 variables hypothesized to be important to breeding bird habitat (Fig 3). These include 6 bioclimate variables in addition to annual potential evapotranspiration of natural vegetation (petnatveg), elevation, slope, distance to water and biome. Potential evapotranspiration was included to represent potential productivity changes under warmer weather conditions. Potential evapotranspiration has been related to species richness of insects, an important food base for birds [56]. We ran MaxEnt algorithms with all 11 continuous environmental and biophysical variables and one categorical predictor, biome (see [32] for discussion of including categorical variables).

For all models, we used the default regularization value of 1 because we did not detect signs of overfitting. We used a bias correction file to account for potential sampling bias that can occur with presence only data, particularly as found in museum data [57, 58]. Specifically, we created a sampling layer by delineating a10 km buffer area around the observed presence for all bird species. This buffer zone represented the typical areas and habitats surveyed for birds along the Rio Grande. Bias correction is achieved by directing MaxEnt to draw background data, which is used to validate the model, from the buffer area.

Each model was calibrated by splitting species occurrence data into training (15%) and testing (85%) sets. We used 15-fold cross-validation and created an average model for each algorithm to assess model fit. Training and testing subsets were randomly selected for each of 15 model iterations. We used threshold-independent Area Under the Curve (AUC) of the Receiving Operator Characteristic (ROC) curve as a metric of model accuracy. The AUC value ranges between 0.5 and 1.0, where values of 0.5 indicate no difference between scores of specificity and sensitivity and scores of 1.0 indicate no overlap between the distributions of the scores. As noted by Yackulic et al. 2013[58], if detection covaries with covariates used in the model, the AUC values represent how well the model classifies detections versus background (which may or may not represent a true absence). For this reason, AUC values may not be a good measure of whether a particular model is good or not and caution is advised [31, 59, 60]. For the purposes of the current analysis, we use the MaxEnt generated AUC values to generate an idea of how well our predictor variables can explain presence of species and assume presence is correlated with suitable habitat. We used the Boyce index [61, 62] as an additional measure of model predictive power. The Boyce index relies on a predicted to expected presence frequency (PE) to compare model results to what would be expected from chance alone. To calculate PE, we defined six classes of habitat suitability from MaxEnt output (ranging from low to high suitability). Predicted frequency is calculated as the number of presence points within each class divided by the total number of points. Expected frequency is the number of evaluation or grid points within each class divided by the total number of points. The Boyce index varies from -1 to 1 where negative values indicate an incorrect model, values near neutral represent random and positive values indicate the model is consistent with observed presence values. If the model is good, PE will be higher for higher suitability classes and can be assessed using Spearman’s rho.

We used a jackknife analysis to determine individual variable importance. To do this, a series of models is run that excludes each variable in turn. Variable importance is measured as the increase in gain of the model with and without variables. Gain is measured as model fit where a likelihood statistic is used to maximize the probability of presences in relation to background data. MaxEnt assigns a percentage to the environmental variable based on the increase in gain associated with the variable. Through this process, Maxent assesses the contribution of each variable to the model gain and its importance for predicting presence of species (Fig 3).

#### Change to suitable habitat

We converted MaxEnt logistic output to data representing suitable versus nonsuitable habitat by using a threshold value equalizing sensitivity and specificity for training samples. This method is assumed to equalize the risk of over and under specifying actual suitability [63]. We choose this measure to maximize the likelihood that this analysis would accurately identify areas suitable for each species but also capture potential sites of interest (e.g. [64]). We created new layers representing thresholded model output to create consensus maps of predicted change where consensus was determined by the prediction of presence by at least two models (Figures A-C in S1 File). We also assessed results from individual models based upon each GCM to estimate the range of possible outcomes. We compared the distribution of suitable habitat over different land groups using Bureau of Land Management’s (BLM) New Mexico “SurfaceManagementAgency” dataset (vector file from communicator.gov/ArcGIS/rest/services/SurfaceManagementAgency/MapServer).

## Results

### Model and variable performance

Models performed well and AUC values for the Lucy’s warbler were 0.95 (±0.019), 0.97 (±0.02), and 0.95 (±0.02) under CGCM3.1, HadCM3 and GDFL CM 2.1 scenarios, respectively. AUC for the southwestern willow flycatcher were 0.83 (±0.50), 0.85 (±0.493), and 0.87 (±0.49) for CGCM3.1, HadCM3 and GDFL CM 2.1 scenarios, respectively. For the yellow-billed cuckoo, AUC were 0.94 (±0.03), 0.95 (±0.03), and 0.94 (±0.03) for CGCM3.1, HadCM3 and GDFL CM 2.1 scenarios, respectively. Boyce Index values ranged from 0.942 to 1 indicating model predictions were consistent with the observed distribution of presences. The predicted to expected ratios were positively and significantly (P = 0.017–0.003) related to suitability classes for all species. Distance to water was the most important variable for all species (Fig 3, S2 Appendix). Lambda files for each model run are available in S3 Appendix.

Elevation, distance to water and diurnal variation in temperature accounted for the great majority of the variation in suitable habitat for Lucy’s warblers (Fig 3). In general suitable habitat was associated with areas found nearer water and at lower elevations (Response curves are available in S3 Appendix). Suitability of habitat was greater for areas that experienced greater range of diurnal temperatures and intermediate values of July maximum temperatures. Suitability was negatively related to low potential evapotranspiration of natural vegetation but increased with increasing values above a certain threshold.

Distance to water, slope, diurnal range of temperature and biome were the most important variables for predicting southwestern willow flycatcher habitat suitability (Fig 3). Suitability was negatively associated with riparian areas located within Great Basin Conifer Woodland and Semidesert Grassland biomes and positively associated with riparian habitats within the Plains grassland biomes (S2 and S3 Appendices). Flycatcher habitat suitability decreased with increasing distance from water. Slope contributed as both linear and hinge functions with higher quality habitat associated more strongly with areas with low slope. Mean diurnal range of temperatures that fell within values of 17 and 19°C were most strongly associated with suitable habitat whereas areas with ranges less than 17°C were less suitable.

For the yellow-billed cuckoo, distance to water and elevation were very important with potential annual evapotranspiration, slope and precipitation during JJA somewhat important (less than <10% permutation importance) (Fig 3). Overall, cuckoo habitat suitability declined with increasing distance from water and elevation (S2 and S3 Appendices). Interactions were also found for the cuckoo that were not found for other species (S3 Appendix). Specifically suitability was negatively associated with mean diurnal temperature*distance to water, Isothermality*elevation. Though not among the most important variables, cuckoo habitat suitability was strongly and negatively associated with maximum July temperature for all three models. In general, suitability declined with increasing values of petnatveg at low values but increased with increasing values after a minimum threshold was reached.

### Changes to distribution of suitable habitat

**Fig 4:**
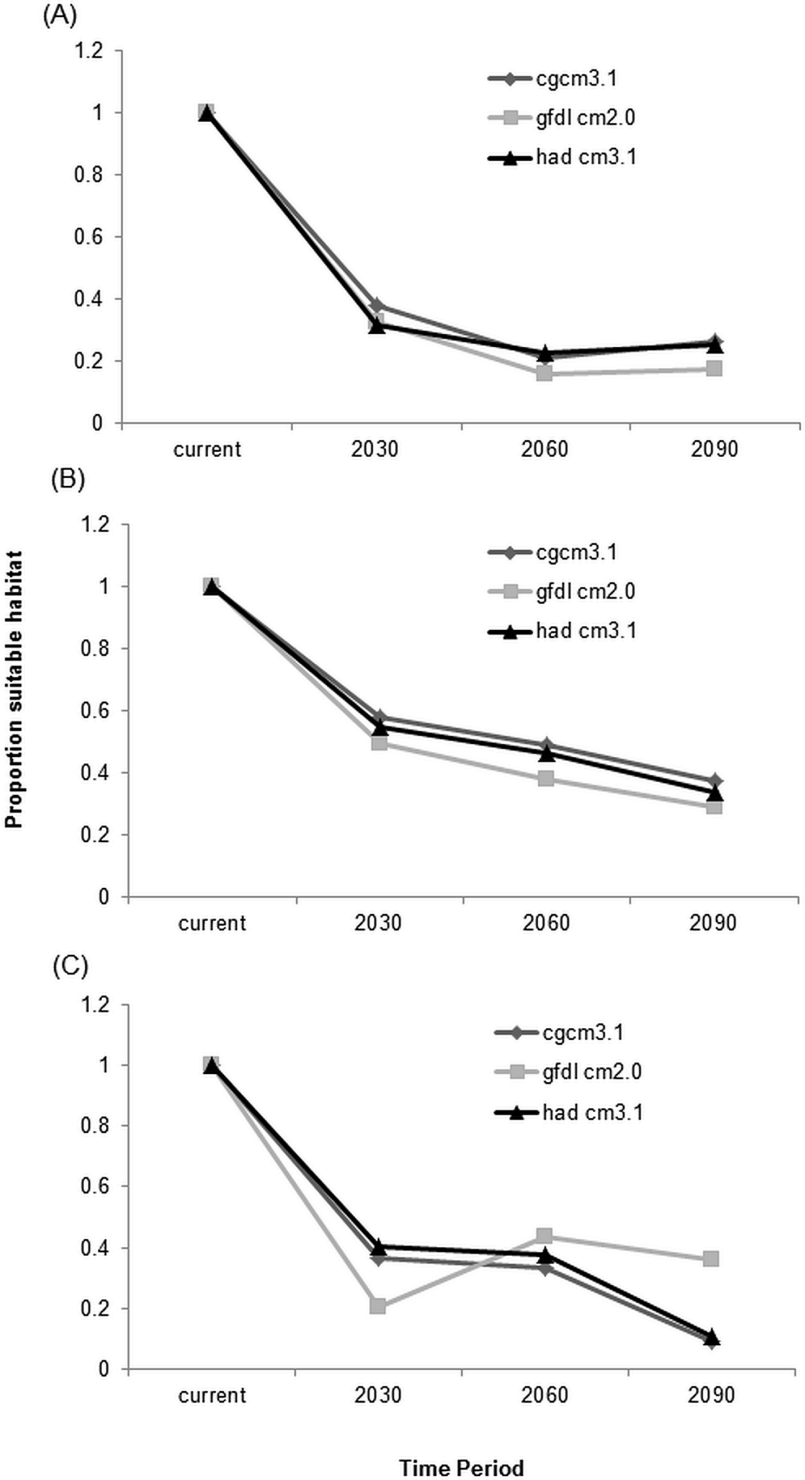
Change in suitable habitat for three bird species under three climate scenarios. A) Lucy’s Warbler; B) Southwestern willow flycatcher; C) Western yellow-billed cuckoo.

**Fig 5:**
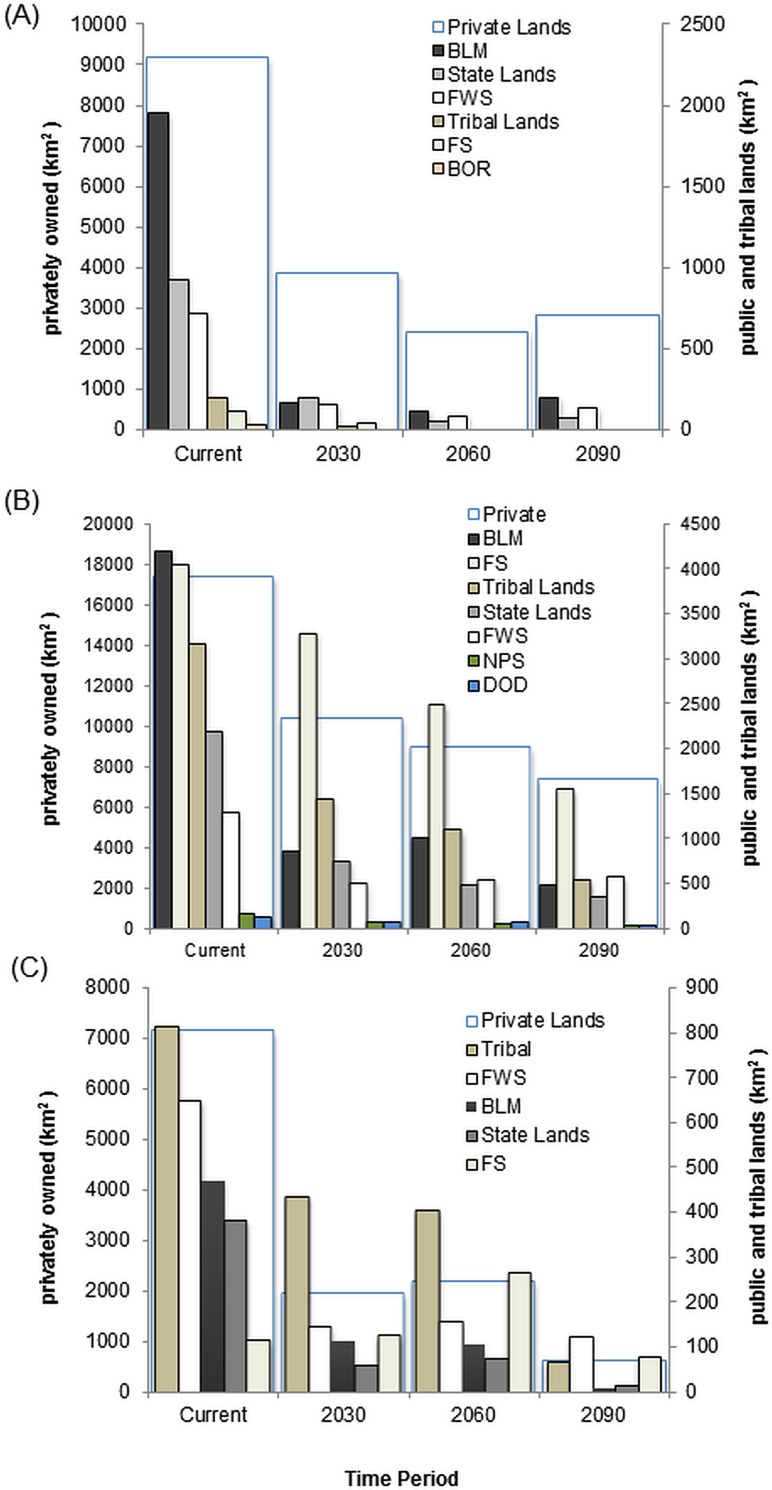
Availability of suitable habitat for three bird species under three climate scenarios. A) Lucy’s Warbler; B) Southwestern willow flycatcher; C) Western yellow-billed cuckoo. Habitat predictions are shown for the most important (>50% area) land management or ownership categories. For all three species, the greatest proportion of suitable habitat is found on privately owned land. BLM = Bureau of Land Management, BOR = U.S. Bureau of Reclamation, DOD = U.S. Department of Defense, FS = U.S. Forest Service, FWS = U.S. Fish and Wildlife Service, NPS = National Park Service.

Predictions for suitable habitat were similar for each species under all three scenarios:

All three experienced declines in area of suitable habitat in future periods (Figs 4 and 5).

By 2090, suitable habitat for Lucy’s warbler will decline by 30–65% (Fig 4 and Figure A in S1 File) with most of the remaining suitable habitat located in the middle Rio Grande Valley. Losses in the southern part of its current predicted distribution are not made up by a shift northward (Fig 4). Suitable habitat is found predominately (well over 50%) on privately owned land for all time periods (Fig 5). Federal land holdings comprise about 15% of predicted suitable habitat where it is primarily within the jurisdiction of the Bureau of Land Management (BLM).

Southwestern willow flycatcher habitat is distributed throughout the riparian corridor within the study area (Fig 4 and Figure B in S1 File). Habitat declines were greatest in low elevation areas along the middle reach of the Rio Grande (when combining all three models). Estimated suitable habitat declines by 30 to 65% across the three models (Fig 4 and Figure B in S1 File). Most of the current suitable habitat is found on private lands with Federal lands comprising the next largest group (Fig 5). The US Forest Service (USFS) holds most of the federal land predicted to be suitable for the flycatcher across all time periods (Fig 5). The importance of other agencies, such as BLM, state land and tribal lands decreases over time as they become an increasingly smaller percentage of land holdings.

Yellow-billed cuckoos are estimated to lose 65–98% of their suitable habitat (Fig 4 and Figure C in S1 File). Current predicted habitat is well dispersed along the study area but becomes more concentrated in northern or middle regions over time (Fig 4 and Figure C in S1 File). Private and Tribal land comprises the majority of current and future suitable habitat (Fig 5). Current distributions are predominately on tribal lands followed by U.S. Fish and Wildlife Service (USFWS) and BLM. By 2090, USFWS becomes the primary land management agency followed by Tribal lands (Fig 5). Remaining agencies manage only a very small (<1000 km) portion of the areas predicted to remain suitable.

### Refugia

**Fig 6:**
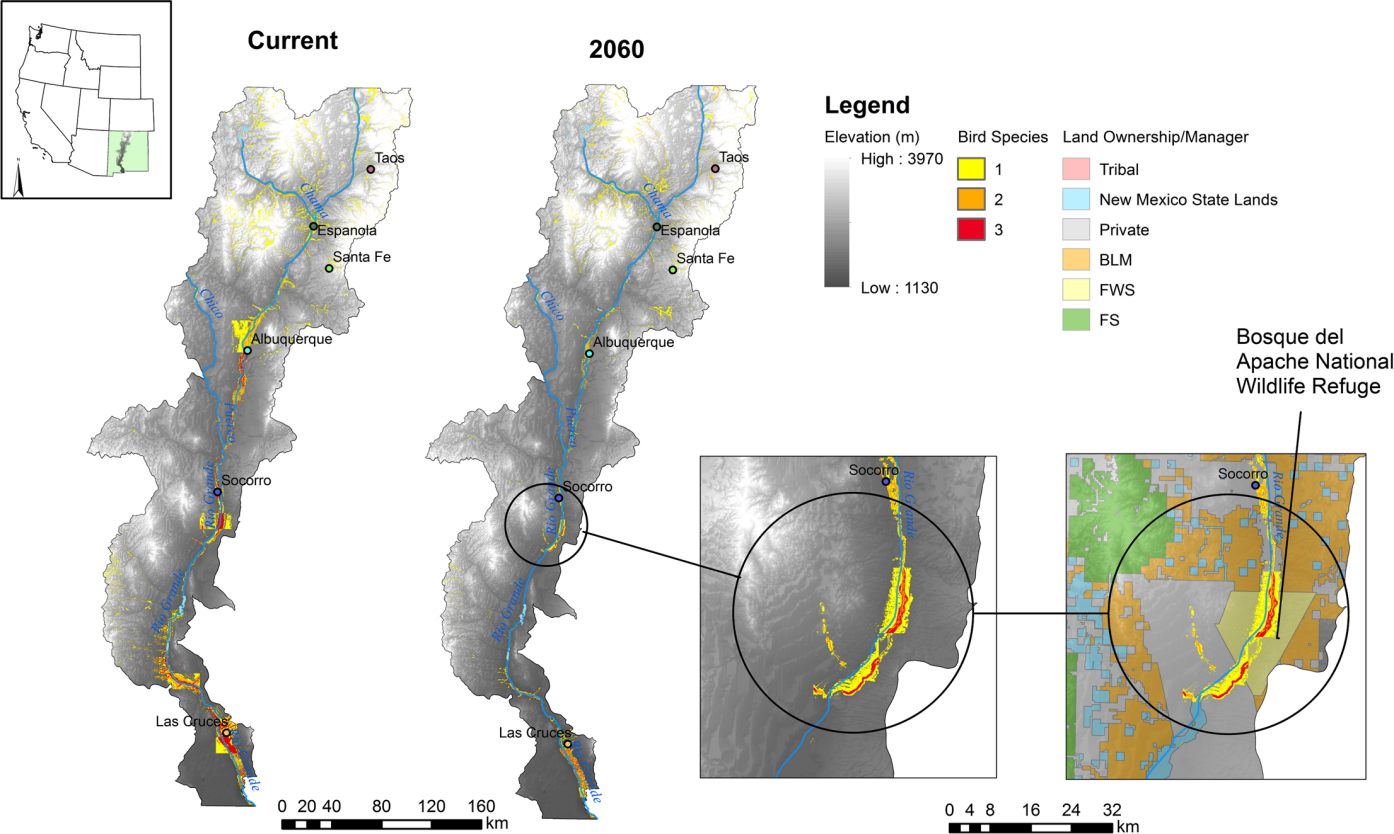
Current and mid-century projections of suitable habitat for three bird species, Lucy’s warbler, Southwestern willow flycatcher, and Yellow-billed cuckoo. Colors indicate predicted habitat for one (yellow), two (orange), or all three (red) species in current and future time periods. Each species’ predicted suitable habitat is based on a 2/3 consensus model (see text). Inset: Expanded area shows the largest suitable patch area for all three species under future climate conditions is found within and adjacent to Bosque del Apache National Wildlife Refuge. BLM = Bureau of Land Management, FS = U.S. Forest Service, FWS = U.S. Fish and Wildlife Service.

Areas suitable for all three bird species declines dramatically as early as 2030 (Fig 6). Two areas, Bosque del Apache National Wildlife Refuge and habitat around the Caballo and Elephant Butte reservoirs are most likely to sustain breeding populations over time (Fig 6).

## Discussion

Our analysis indicates potentially large declines in suitable habitat for three bird species that breed within the Rio Grande Bosque. Distributions of suitable habitat within the study area did not shift in a linear fashion so much as recede into higher elevation or wetland sites (e.g. Bosque del Apache, Fig 6, S1 File). As a result, remaining habitats for these bird species are likely to become smaller and more concentrated. Currently, and over time, much of the suitable habitat for these species is found on privately owned lands (Fig 5). Still, there are substantial shifts in expected suitable habitat across lands managed by federal and tribal entities, particularly for the Southwestern willow flycatcher and yellow-billed cuckoo. Because the population size of any species is a function of area of suitable habitat as well as local density of individuals [65], we cannot expect the loss of habitat to affect a species in a universal manner across it range. Specifically, areas that support high densities of individuals will have a greater impact on the population. Thus, those areas showing highest suitability may not all be created equal and further studies are needed to identify which might be most important for species conservation.

### Future habitat characteristics for three bird species

Biophysical variables were the most important predictors of habitat suitability for these species (Fig 3). Previous studies have noted similar patterns. Johnson et al. (2012) found terrain ruggedness, which was taken to represent a variety of biophysical factors including slope, to be the most important predictor variable for yellow-billed cuckoos in a study that used satellite imagery. Slope was directly incorporated into our models but was not among the most important variables in terms of permutation importance for explaining cuckoo habitat (Fig 3). For both Lucy’s warbler and the cuckoo, elevation presented a much stronger predictor of suitable habitat. In contrast, slope was among the more important predictors for the southwestern willow flycatcher, which may reflect its association with floodplains. The flycatcher presence data used in this analysis included observations from northern, higher elevation sites, and this is reflected in estimates of current suitable habitat under all three climate scenarios (Figure C in S1 File). Tellingly, elevation was not as strong an influence on predicted distribution for the flycatcher as it was for the other species, whose samples came almost entirely from lower elevation areas along the Rio Grande south of Albuquerque. This may mean that our models underestimate potential suitable habitat for the cuckoo and warbler, because they exclude potentially suitable habitats in the northern, higher elevations sites. In addition, slope varies little near the Rio Grande in the southern portion of the study area as compared to the relatively mountainous northern areas. Therefore, it could be expected that due to the limited range of slope values associated with sample sites, this analysis would not detect a relationship between slope and warbler or cuckoo presence.

Distance to water was consistently important for all species. In part, this is an artifact of the strong spatial autocorrelation that exists between the river and species presence- a not unexpected occurrence for the birds of this study, which breed along a relatively narrow corridor within riparian forests. One concern of allowing Maxent to consider background points from areas adjacent to actual observations is that it may introduce bias by restricting potential estimates to areas near rivers, where most observations are made. However, distance to water was consistently the most important predictor of suitable habitats, supporting the notion that species tend to be proximate to the water itself as compared to randomly selected points within the riparian corridor. Further, the nature of habitat used by riparian dependent species is such that many of the concerns of bias sampling (e.g. near roads or riparian areas) are likely real covariates for the presence of species. However, the used of a bias correction file did appear to improve our ability to detect future suitable habitats (i.e. model performance). Importantly, our estimates of future conditions included neither potential changes in available water, nor changes to the river as a result of continuous and prolong droughts. Such changes are likely to reduce available habitat even further than is estimated here.

Potential evaporation (PET) of natural vegetation influenced predictions of suitable habitat for all three species. PET is one way that precipitation leaves the system and is determined by type and characteristics of pre-existing vegetation. Potential evapotranspiration, net primary productivity, temperature, and variation in temperature (considered a measure of thermal energy) have been positively associated with insect richness [56]. In the arid west, associations with water for the cuckoo [66, 67] and southwestern willow flycatcher [21] are assumed to relate to their requirement for humid nest sites. It is also noteworthy that these areas are likely to contain a greater amount of prey items than the surrounding desert and scrub habitats. Willow flycatchers are dependent upon localized food sources, largely insects, during nesting and years of low rainfall and resulting reduced food supplies have been associated with lower reproductive success in the flycatcher [21]. Perhaps this explains why the presence of water in the form of open bodies, cienagas, marshy seeps appears to be an important correlate for the flycatcher [68] though nest sites are known to remain active in areas several years after water has gone. Similarly, interannual fluctuations in western yellow-billed cuckoo populations have been associated with changes in food supply where the rapid breeding cycle of the cuckoo is thought to depend upon a large supply of relatively large (katydid, caterpillars, cicadas) prey items [67]. Kirkpatrick et al. 2009 [69] [70]found that the relative abundance and richness of bird species was positively associated with the extent of surface water and aerial arthropod abundance. Bird diversity was also higher (68% more species) in riparian areas versus upland areas perhaps reflecting differences in cover and food resources. Collectively these studies point to the importance of riparian habitats as both cover and food resources. The BOR hydrological models used in this analysis base their calculations of PET on output of historic vegetation and land cover classes [71] and do not account for changes in land cover. It is therefore likely that projected changes in potential evapotranspiration of natural vegetation will vary from the predictions made here with additional negative impacts possible where land use or invasive species reduce natural riparian habitats.

Among the species analyzed, the yellow-billed cuckoo appeared to be the least influenced by climate variables though all species showed some relationship to temperature range, max July temperature and precipitation during JJA (Lucy’s warbler excluded, Fig 3). Climate variability may be quite important for species where they affect the margins of population [10]. Lucy’s warblers and Western yellow-billed cuckoos predominately inhabit the southern half of our study area, whereas the Southwestern willow flycatcher tends to breed throughout the area. We explored two variables, isothermality and annual temperature range, to represent response to weather variability [26]. Isothermality represents the relative influence of daily (average for each month) versus annual temperature fluctuations. Mean annual temperature range (bio7) represents the range of extreme temperature conditions. These measures were generally not important for the birds studied here, perhaps due to the seasonal presence of species in this area. Of the two, mean diurnal range did vary with predicted suitability for Lucy’s warbler and the Southwestern willow flycatcher. The flycatcher appears to associate with sites with intermediate values of diurnal temperature whereas the warbler was associated with areas with higher range of temperatures. Within our study area, climate variability can be dramatic within a relatively small area due to elevation gradients. Specifically, mean climate variation is greater in the north due to the relatively greater elevation gradients. Northern areas are also cooler on average. However, diurnal extremes are greater in the south where days can get very hot and nights cool. Whether the observed associations in this study represent biological limitations or are simply correlates remains to be determined.

Species with strong associations with vegetation characteristics are expected to be highly vulnerable to future shifts in climate conditions if those conditions result in a total turnover in associated habitat [4]. The degree to which species are likely to be negative impacted increases with specialized habitat requirements or reliance on multiple geographically distance habitats (e.g. as with migrants) [5]. The likelihood is low that habitats will shift distribution in unison with climate conditions and predictions that show substantial shifts (Fig 2) indicate a large risk for species inhabiting areas along the Rio Grande. Given variations in individual species’ capacity to shift range or survive new climate conditions, it is not unimaginable that new habitats may form, with unknown consequences for the species assessed in this study. In this analysis, the willow flycatcher was positively associated with riparian areas within the Plains Grassland biome and negatively associated with Semidesert Grassland, a biome that is predicted to increase substantially (Fig 2). Woodland and forested habitats also show large replacement by Chihuahuan Desert Scrub and Semi-Desert grasslands (Fig 2). Though we did not find a statistically significant association between biome and suitable habitat for Lucy’s warbler and yellow-billed cuckoo, these species are also at risk of experiencing declines in associated habitat. Cottonwood-willow habitat important to both Lucy’s warblers [16] and the yellow-billed cuckoo [72, 73] is expected to decline under climate changes due to reduced water availability [6]. Further, potential increases in riparian scrub habitat may reduce the likelihood of cuckoo presence [74]. Recent declines of nearly 50% in screwbean mesquite is a potential source of concern for Lucy’s warblers [18]. Declines in mesquite habitat as a result of development and flow regulation [18] are likely to continue under climate change without efforts to restore natural flood cycles and reduce urban growth along the river.

### Management implications

Though our models performed well, we must use caution when interpreting these results because the analysis assumes that species presence can be predicted solely by characteristics of a landscape and associated climate. In reality, it is likely that additional factors may be important for predicting species’ presence and species’ response to climate changes. Of note, the potential for predicted reductions in suitable habitat may lead to substantial increases in competitive pressures between these species as well as others not considered in the current analysis. Habitat patterns are a known driver of wildlife population dynamics but analysis of habitat suitability may not accurately represent species response to change [36] because ecological niche models often do not consider colonization or competitive interactions [75]. Additionally, temporal components and predation impacts on species distribution are only measurable to the degree that they can be predicted, which typically do not account for complicated interactions. Still, ecological niche models have proven useful for a variety of purposes including mapping potential ranges under changing conditions, for different time periods, and to identify potential new populations [32, 34–36, 65, 76]. In general, ecological niche analyses are best used for situations where the goal is to identify areas unlikely to support species survival [77]. Indeed, others [65] note that while species may not be present at locations with high suitability, it is very likely that they will not be found where low suitability is predicted and thus they are best used to predict the absence of species. It may also be, given valid model assumptions, that estimates of suitability can be used to infer abundance where areas of high suitability indicate greater abundance and areas with low suitability low abundance [65]. Though we do not attempt to estimate abundance and advise caution in assigning certainty to the future of these species based on these models alone, we present these results as a first assessment of the direction and magnitude of climate change impacts on riparian obligate species in the Rio Grande.

Riparian habitats along the Rio Grande provide critical nest and stopover sites [3]. This analysis begins to identify high priority stopover sites and migration habitats under climate change. Such information is critical for conservation strategies that aim to increase the long-term persistence of migrating species [3]. Our models show increasing fragmentation or isolation of suitable habitats for all three species (Figures A-C in S1 File). Fragmentation has the potential to affect not only habitat availability but gene flow, particularly where fragmentation acts as a barrier, the latter of which may be most important for predicting species persistence [10]. Increased weather perturbation could also cause temporary absences in habitat that is moderately fragmented (as might occur at the edge of a species’ range) and ultimately cause permanent retractions in species’ distribution [10]. These issues have large consequences for species like the yellow-billed cuckoo whose rapid breeding cycle and dependence upon a large supply of relatively large prey items indicates a requirement for larger tracks of forest [67].

Opdam et al. 2004 [10] identify several important strategies to minimize the compounding issues of climate change and fragmentation. Namely, maintaining spatial cohesion through stabilizing core critical areas and maintaining heterogeneity and permeability of landscape for species travel. If the modeled projections of this analysis are correct, many areas are unlikely to provide suitable nesting habitat under climate change. Therefore, the best strategy for conserving wildlife species along the Rio Grande will begin with the preservation and restoration of critical riparian habitats and conditions as well as the return of natural flood pulses [46]. Fire remains a serious threat in these habitats [46] and studies have shown both positive and negative response of wildlife to fire treatment efforts within the Rio Grande [44, 45]. However, the effectiveness of restoration activities, species relocations, and even the timing of water releases to improve wildlife habitat quality may change in the future due to the effects of warming and changes to precipitation regimes. Species tolerance to future conditions is also an important consideration because it could limit natural dispersal and colonization processes and confound efforts to restore sites where transitions to more arid conditions are eminent.

From this analysis, it appears that current refugia may become increasingly important. Bosque del Apache National Wildlife Refuge and wetlands surrounding Elephant butte and Caballo reservoirs, in particular, show strong potential to support breeding birds throughout the next century (Fig 6). Within these areas, care must be taken to address structural and compositional environmental conditions that are likely important for providing suitable habitats and will help systems remain resilient in the face of climate change. Lucy’s warblers are likely to benefit from management practices that encourage diverse age classes and composition among riparian habitats[16]. Structural heterogeneity is a defining characteristic of cuckoo habitat at small scales (72 ha or smaller) though this relationship changes at larger scales where greater heterogeneity probably represents greater fragmentation of natural vegetation types [67]. Engagement with the public and programs relating to conservation easements and restoration are also critical given the high percentage of lands under private ownership (Figures A-C in S1 File).

Given our current understanding of climate impacts, preservation and restoration of functional riparian habitats will be critical for the three bird species assess in this study. Equally important to riparian conservation efforts, which must consider down and upstream effects, is to view these trends more broadly and include considerations of multiple species needs, land ownership patterns, and the potential of sites to supply not only wildlife habitat but other ecosystem services. In this pursuit we have provided information on expected change to wildlife habitat that can be integrated with other considerations to best inform management strategies under climate change.

## Supporting Information

S1 Appendix Selection of climate scenarios for the current study. Fig 1. Legend inclusive. Modeled change in monthly average temperature and precipitation under three climate models.
(PDF)

S2 Appendix MaxEnt output for three bird species in New Mexico. (PDF)

S3 Appendix Lamda files for MaxEnt runs for three bird species in New Mexico. (XLSX)

S1 File Predicted change in suitable habitat for three bird species. (PDF)
