# Supplementary material for: Implications of Climate Change for Bird Conservation in the Southwestern U.S. under Three Alternative Futures
Source: PLoS One. 2015 Dec 23;10(12):e0144089. doi: 10.1371/journal.pone.0144089 (PMC4689447; doi:10.1371/journal.pone.0144089)
Supplement: S1 Appendix — Fig 1. Legend inclusive. Modeled change in monthly average temperature and precipitation under three climate models. (PDF) [file pone.0144089.s001.pdf]

S1. Appendix 1. Climate model selection for current analysis.

The following is supplied as supplemental information for the manuscript "Implications of climate change for bird conservation in the southwestern U.S. under three alternative futures" by Friggens and Finch.

In general, assessments of individual climate model performance as determined by comparing observed versus modeled historical trends shows considerable variability according to the parameter of interest. [1] in a similar analysis selected one model from each model group ECHAM4, HadCM3, and GFDL to represent global dry, medium and wet projections, respectively (e.g. [2]). Importantly those models that most accurately predicted 20th century precipitation, HadCM3, HadGEM, MRI-CGCM3.2 and MIROC3.2 predict different changes for future conditions in the southwestern U.S. HadCM3 was found to slightly overestimate winter precipitation and GFDL2.1 led to the greatest over estimation [3]. For precipitation during the monsoon season (July through September), CGCM2.3 project small decreases whereas HadCM3 a slight increase in monsoon precipitation [3, 4]. In an analysis of model capacity to accurately describe southwestern precipitation, (Garfin et al. 2010 ranked the models selected for this analysis as intermediate. [5] found the second generation HadCM2 accurately predicted onset and characteristics of precipitation. When comparing predictions of mean summer temperature for GFDL and CGCM3 models, HadCM3 tended to predict greater increases in temperature (hotter than observed) and GFDL predicted lower temperatures than observed [6]. Others report HadCM3 and ECHAM5 (not considered in our analysis) perform best with SW temperature, precipitations and anomalies [7, 8]. Notaro reports the top five performing models as ECHAM3, HadCM3, CCSM3.0, HadGem1, and CNRM-CM3. For this analysis, relative predictions among the models show HadCM3 generated intermediate increases in mean temperature (+4.6 by 2090) and slight increasing annual precipitation by 2090, GFDL predicts the greatest increase in mean temperature (+5.16) and greatest decline in precipitation, and CGCM3.1 generates intermediate temperature increases (+4.82) and moderate declines in annual precipitation (Fig 1).

Projected changes for the Rio Grande Basin show increasing mean and max temperatures for all months from 1990's levels [9]. Overall, precipitation is expected to increase through the 2020's and 2050's but decline during the later decades of the century [9]. Mean annual precipitation is expected to decrease thought the extent and direction of change varies throughout the year (Fig 2). The models used in this analysis show different patterns for precipitation: HadCM3.1

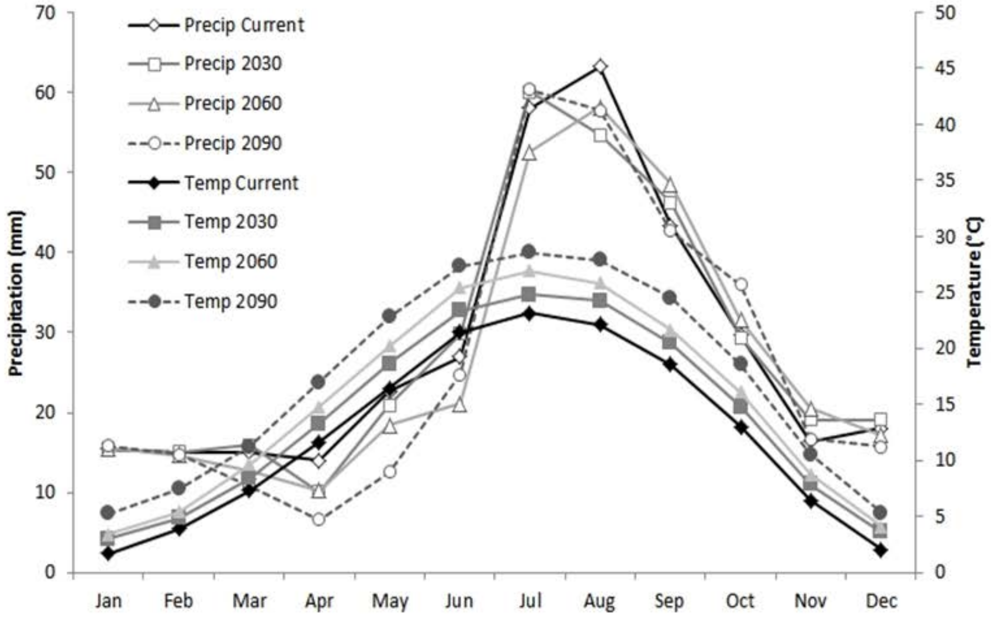

Fig 1. Average temperature and precipitation for current and future time periods over the study area. Climate data were compiled and averaged from downscaled CMIP3 (Gangopadhyay et al. 2011) climate and hydrological projections under three GCMs (CGCM 3.1, GFDL cm2.0, and Had cm3.1).

predictions by 2090 show increasing summer precipitation but the greater declines for winter months, whereas GFDL and CGCM 3.1 show more consistent declines across the entire year (Fig 2). The BOR hydrological projections rely on a VIC hydrological model that estimates water balance at each model grid cell. The VIC model applied in the BOR water projections gives precipitation one of two fates: runoff or evapotranspiration. Runoff is also expected to show some increase in the first half of the century but declines starting in the 2050's [9]. Winter season runoff shows increasing trends whereas spring summer levels generally decrease. The latter corresponds with expected declines in snow water equivalent from 1990's levels [9].

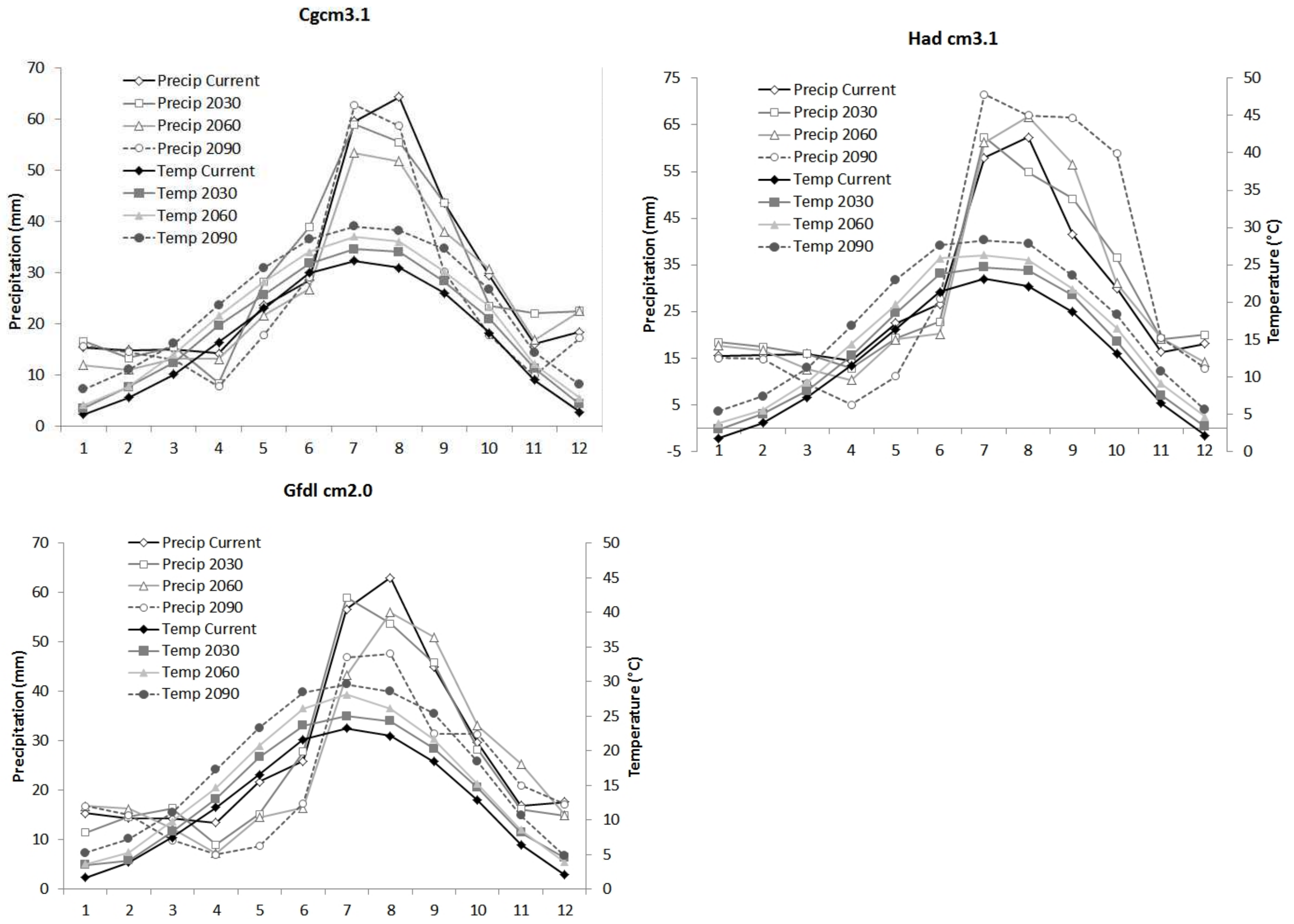

Figure 2. Mean monthly temperature and precipitation for the Rio Grande Basin Climate for three GCMs. Each time period represents 20-year span with the exception of current, which represents conditions averaged over 1970-2013. Climate data were compiled from downscaled CMIP3) climate and hydrological projections (Gangopadhyay et al. 2011).

References:

1. Huntley B, Collingham YC, Green RE, Hilton GM, Rahbek C, Willis SG. Potential impacts of climatic change upon geographical distributions of birds. Ibis. 2006;148(s1):8-28.
2. Cubasch U, Meehl GA, Boer GJ, Stouffer RJ, Dix M, Noda A, et al. Projections of future climate change. J.T. Houghton, Y. Ding, D.J. Griggs, M. Noguer, P.J. Van Der Linden, X.Dai KM, et al., editors: Cambridge: Cambridge University Press; 2001.
3. McAfee SA, Russell JL, Goodman PJ. Evaluating IPCC AR4 cool-season precipitation simulations and projections for impacts assessment over North America. Climate dynamics. 2011;37(11-12):2271-87.
4. Garfin GM, Eischeid JK, Lenart MT, Cole KL, Ironside K, Cobb N. Downscaling climate projections in topographically diverse landscapes of the Colorado Plateau in the arid southwestern United States. The Colorado Plateau IV: Shaping Conservation Through Science and Management. 2010:21.
5. Arritt RW, Goering DC, Anderson CJ. The North American monsoon system in the Hadley Centre coupled ocean-atmosphere GCM. Geophys Res Lett. 2000 Feb 15;27(4):565-8. PubMed PMID: WOS:000085343600031. English.
6. Salazar E, Sansó B, Finley A, Hammerling D, Steinsland I, Wang X, et al. Comparing and Blending Regional Climate Model Predictions for the American Southwest. JABES. 2011 2011/12/01;16(4):586-605. English.
7. Notaro M, Mauss A, Williams JW. Projected vegetation changes for the American Southwest: combined dynamic modeling and bioclimatic-envelope approach. Ecological Applications. 2012;22(4):1365-88.
8. Dominguez F, Cañon J, Valdes J. IPCC-AR4 climate simulations for the Southwestern US: the importance of future ENSO projections. Climatic Change. 2010 2010/04/01;99(3-4):499-514. English.
9. Brekke L. West-wide climate risk assessments: bias-corrected and spatially downscaled surface water projections: US Department of the Interior, Bureau of Reclamation, Technical Service Center; 2011.
