# Supplementary material for: Implications of Climate Change for Bird Conservation in the Southwestern U.S. under Three Alternative Futures
Source: PLoS One. 2015 Dec 23;10(12):e0144089. doi: 10.1371/journal.pone.0144089 (PMC4689447; doi:10.1371/journal.pone.0144089)
Supplement: S2 Appendix — (PDF) [file pone.0144089.s002.pdf]

S2. Appendix. MaxEnt Output for 3 Bird Species in New Mexico.

The following is supplied as supplemental information for the manuscript "Implications of Climate Change for Bird Conservation in the Southwestern U.S. under three Alternative Futures" by Friggens and Finch.

Individual .tif or .png files are available for each variable of 15 permutations upon request (meganfriggens@fs.fed.us).

Naming conventions used in this document:

LUWA= Lucy's Warbler
WIFL = Southwestern Willow Flycatcher
YBCU = Yellow-billed cuckoo

For each bird species, models were created for 3 GCM outputs that can be determined by variable suffixes:
variable_1 = Cgcm3.1
variable_2 = Gdfl 2.0
variable_3 = Had cm3.0

Variable codes are as follows:

Annual Precipitation (prcpan)
Precipitation September (Bio13)
Precipitation JJA (Bio18)
Aridity Index (AI)
Potential annual evapotranspiration of natural vegetation (petnatvan)
Mean Diurnal range Temperature (Bio 2)
Isothermality (Bio3)
Max Temperature JJA (Bio5)
Biome
Distance to water (Dist)
Elevation (DEM)
Slope

The following files are presented in order of species and GCM (e.g. LUWA_1, LUWA_2, LUWA_3, WIFL_1, etc).

Replicated maxent model for LUWA

This page summarizes the results of 15-fold cross-validation for LUWA, created Tue Aug 05 13:21:14 MDT 2014 using Maxent version 3.3.3k. The individual models are here: [0] [1] [2] [3] [4] [5] [6] [7] [8] [9] [10] [11] [12] [13] [14]

Analysis of omission/commission

The following picture shows the test omission rate and predicted area as a function of the cumulative threshold, averaged over the replicate runs. The omission rate should be close to the predicted omission, because of the definition of the cumulative threshold.

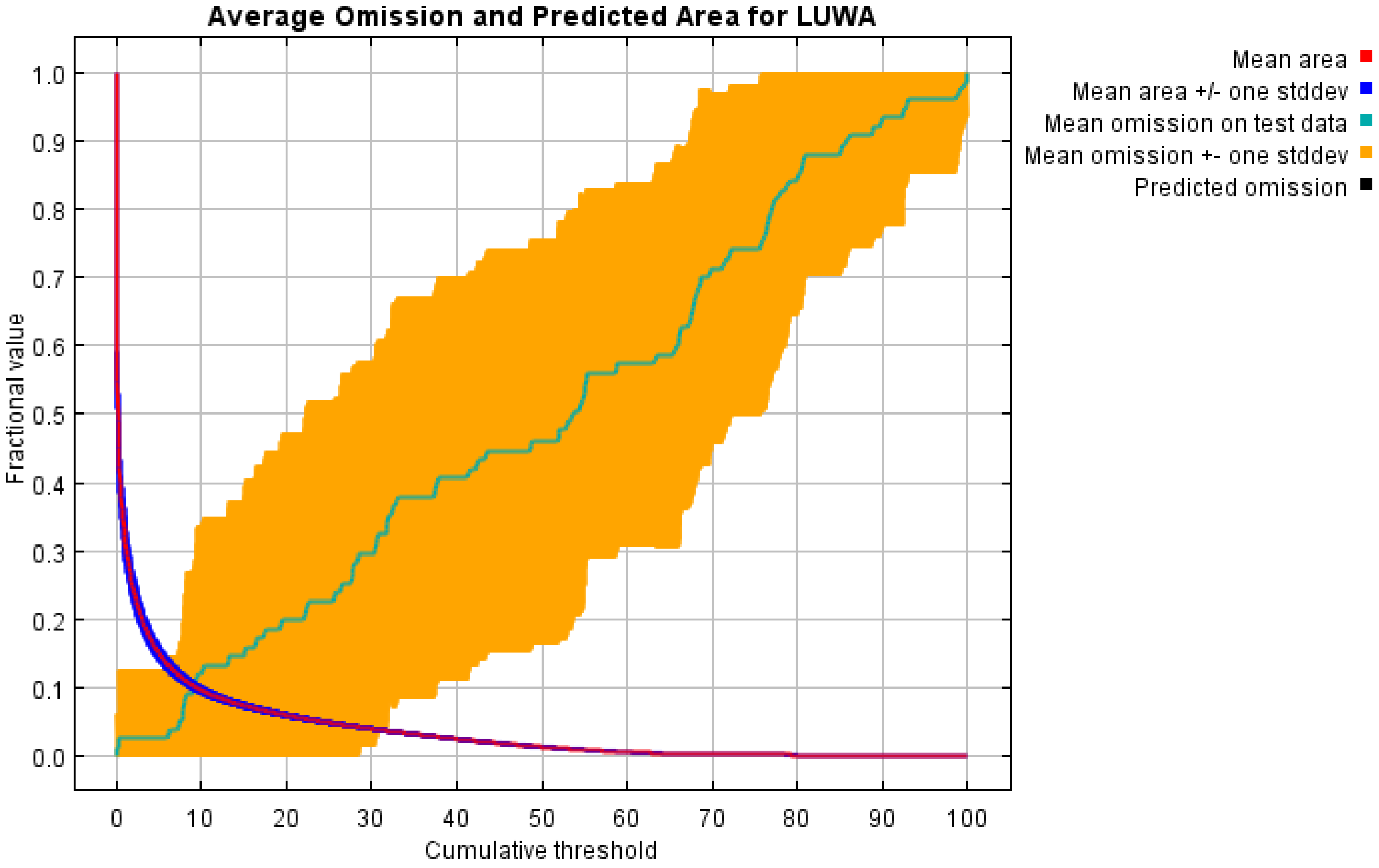

The next picture is the receiver operating characteristic (ROC) curve for the same data, again averaged over the replicate runs. Note that the specificity is defined using predicted area, rather than true commission (see the paper by Phillips, Anderson and Schapire cited on the help page for discussion of what this means). The average test AUC for the replicate runs is 0.957, and the standard deviation is 0.055.

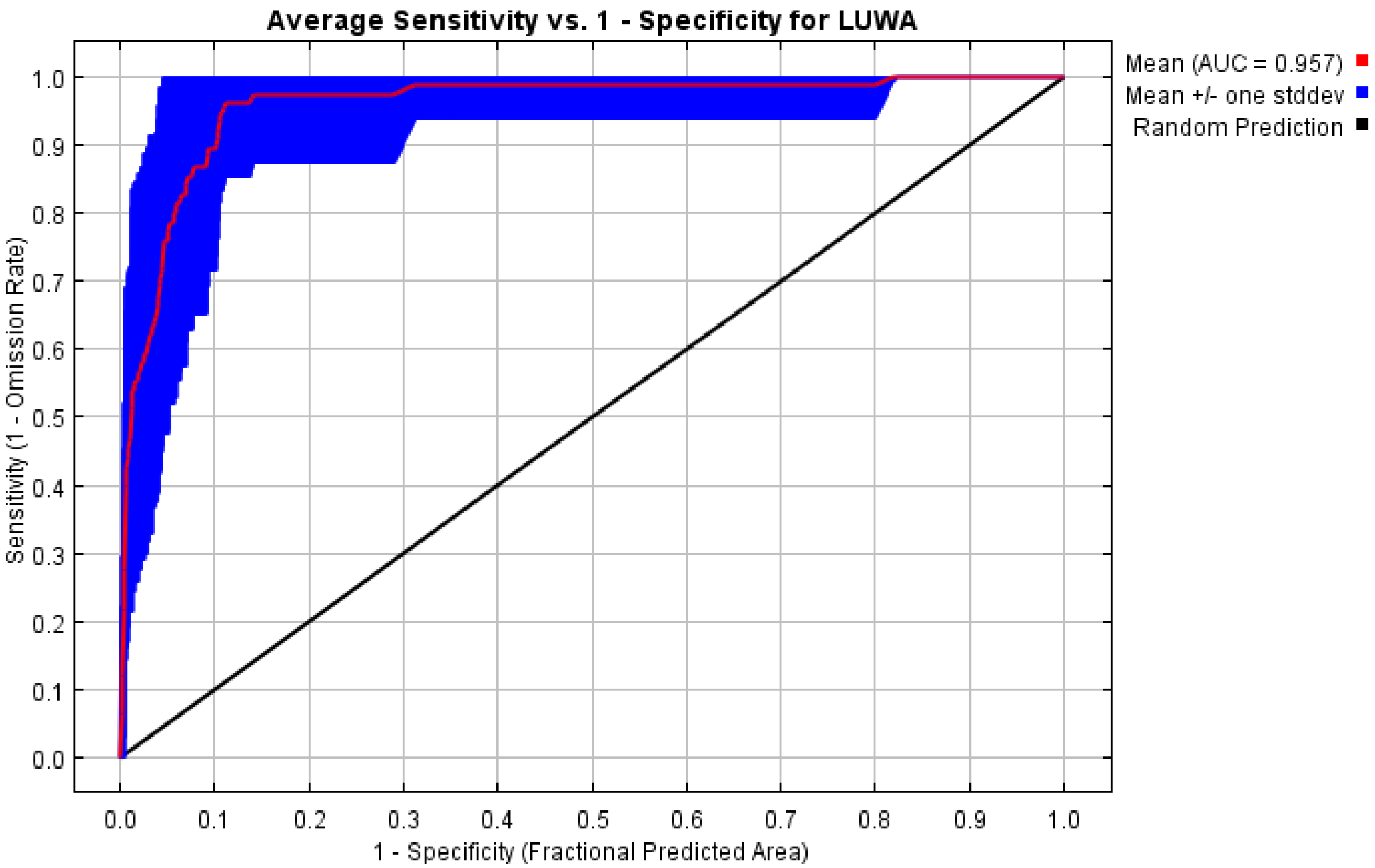

Pictures of the model

The following two pictures show the point-wise mean and standard deviation of the 15 output grids. Other available summary grids are min, max and median.

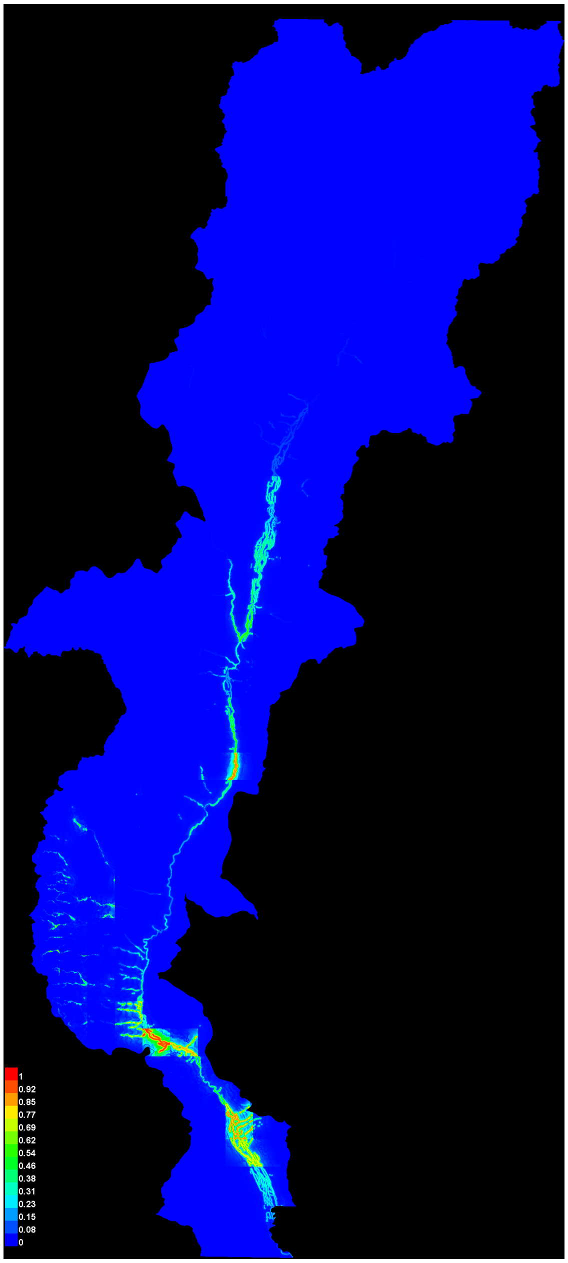

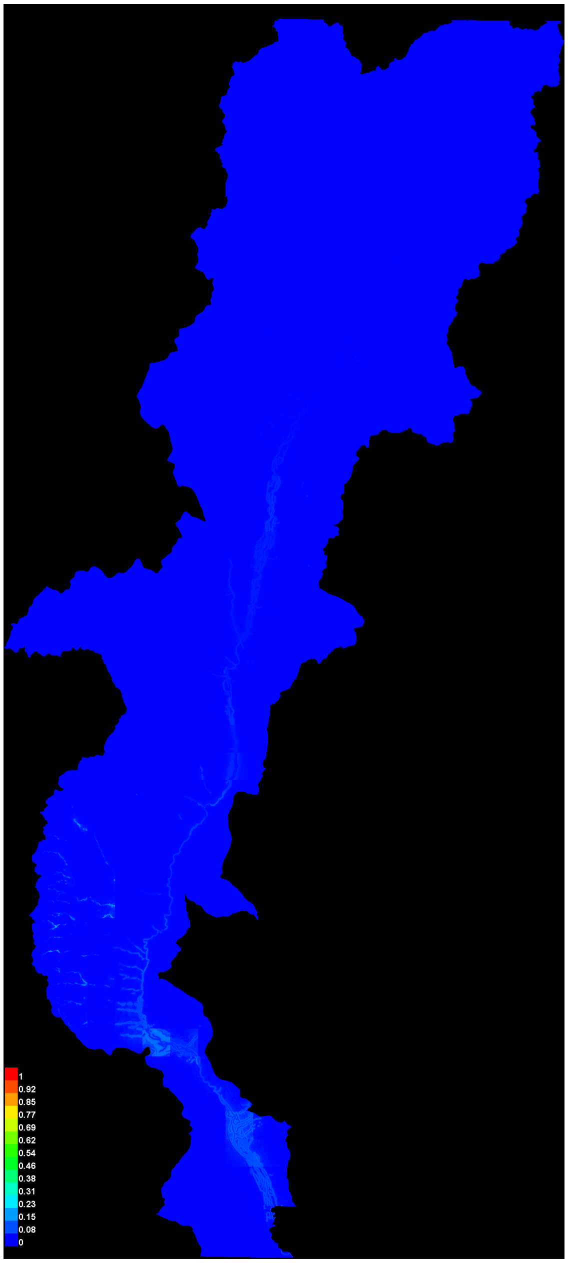

The following two pictures show the point-wise mean and standard deviation of the 15 models applied to the environmental layers in Envir_2030_ascii. Other available summary grids are min, max and median.

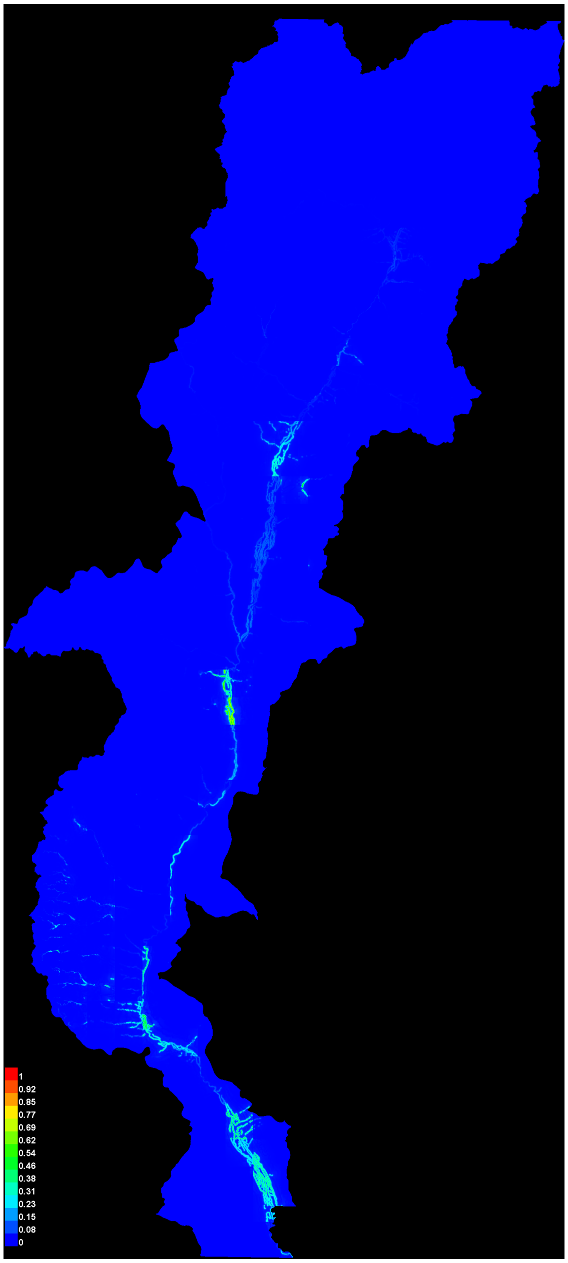

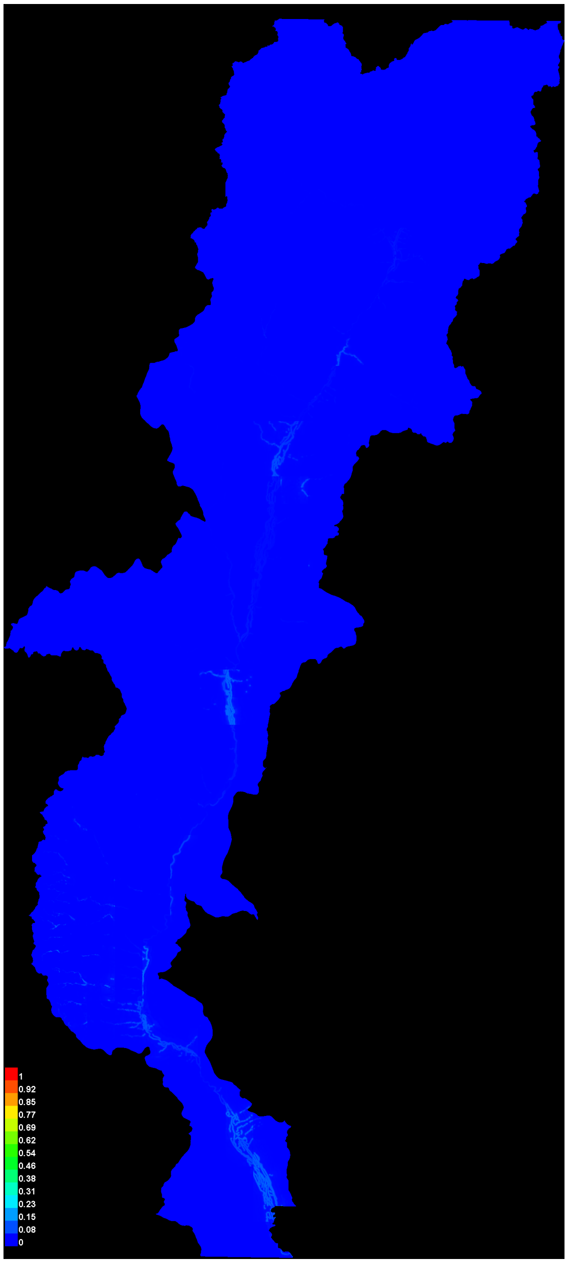

The following two pictures show the point-wise mean and standard deviation of the 15 models applied to the environmental layers in Envir_2060_ascii. Other available summary grids are min, max and median.

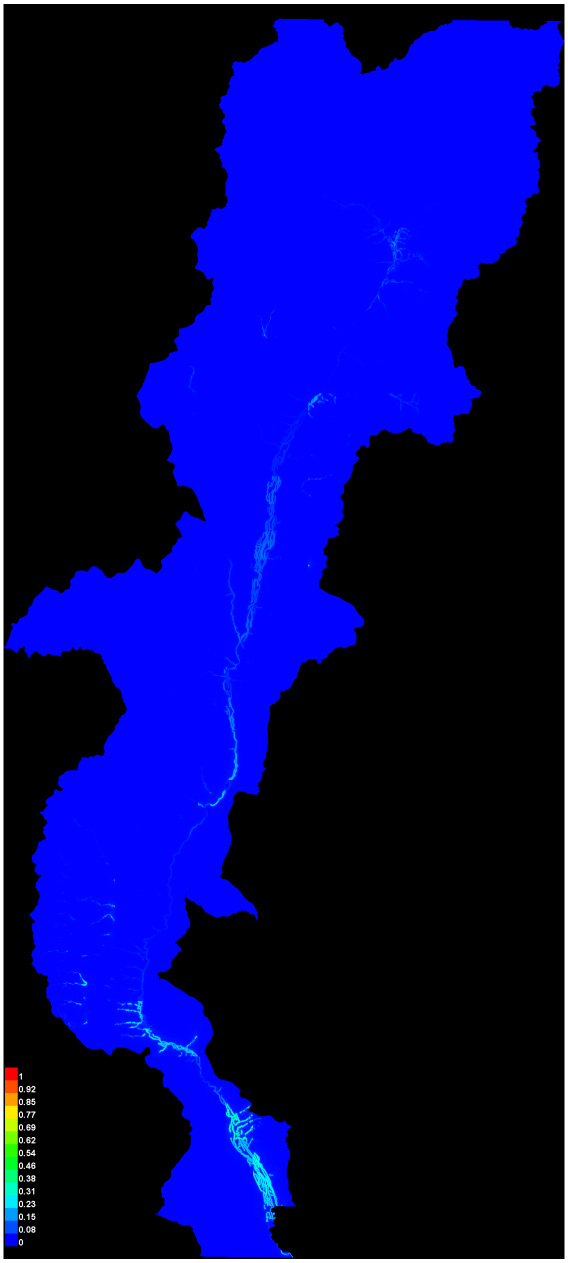

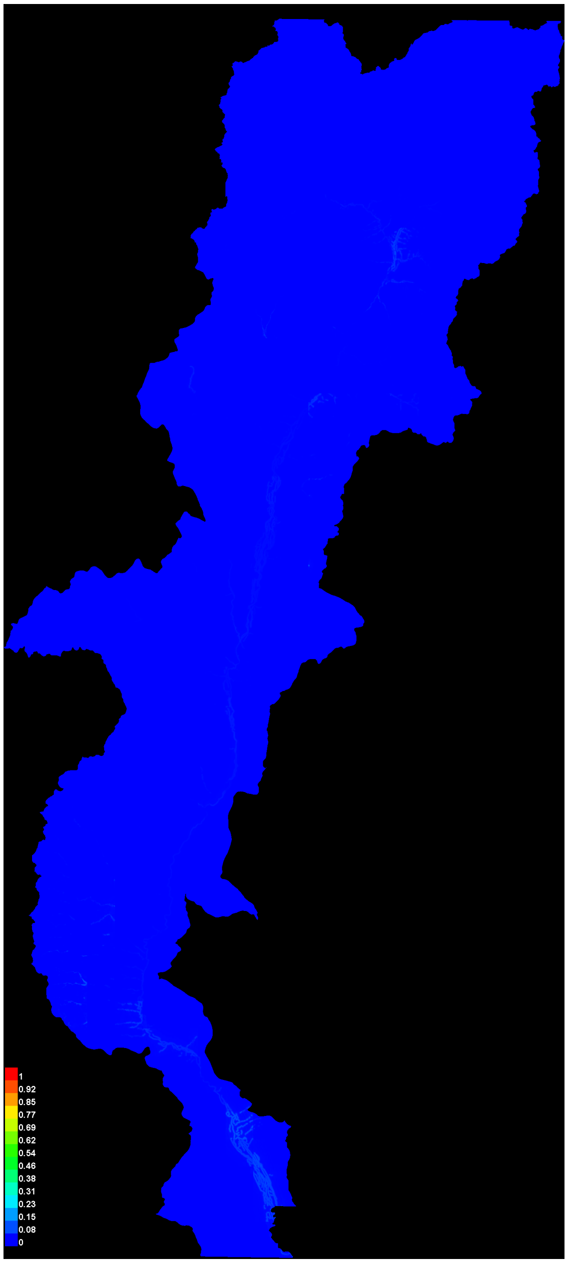

The following two pictures show the point-wise mean and standard deviation of the 15 models applied to the environmental layers in Envir_2090_ascii. Other available summary grids are min, max and median.

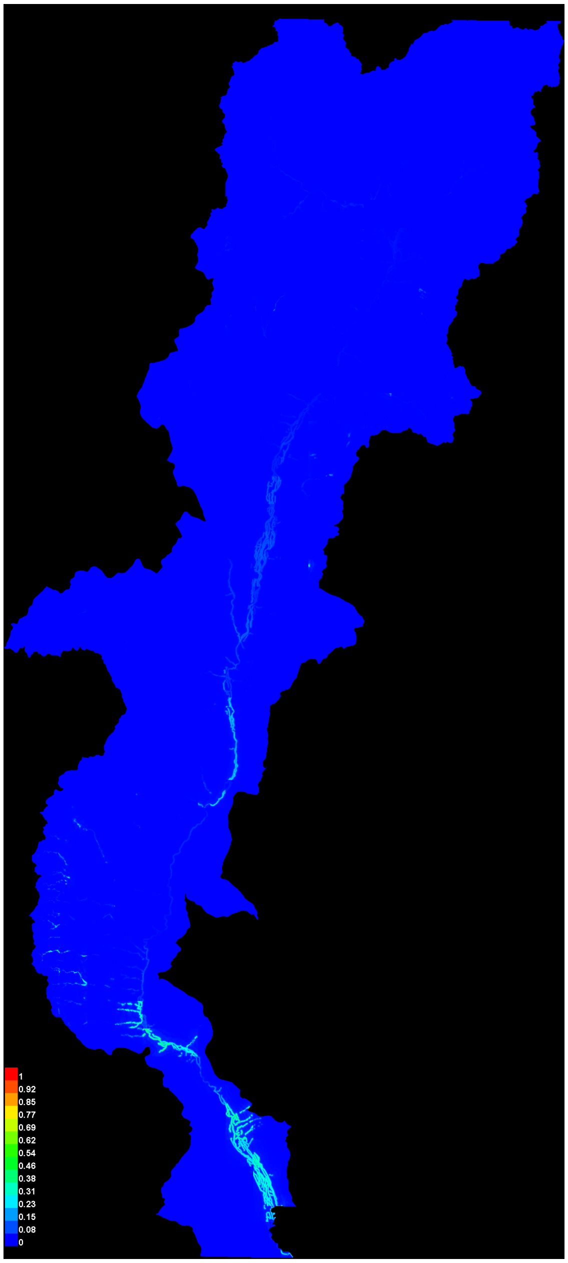

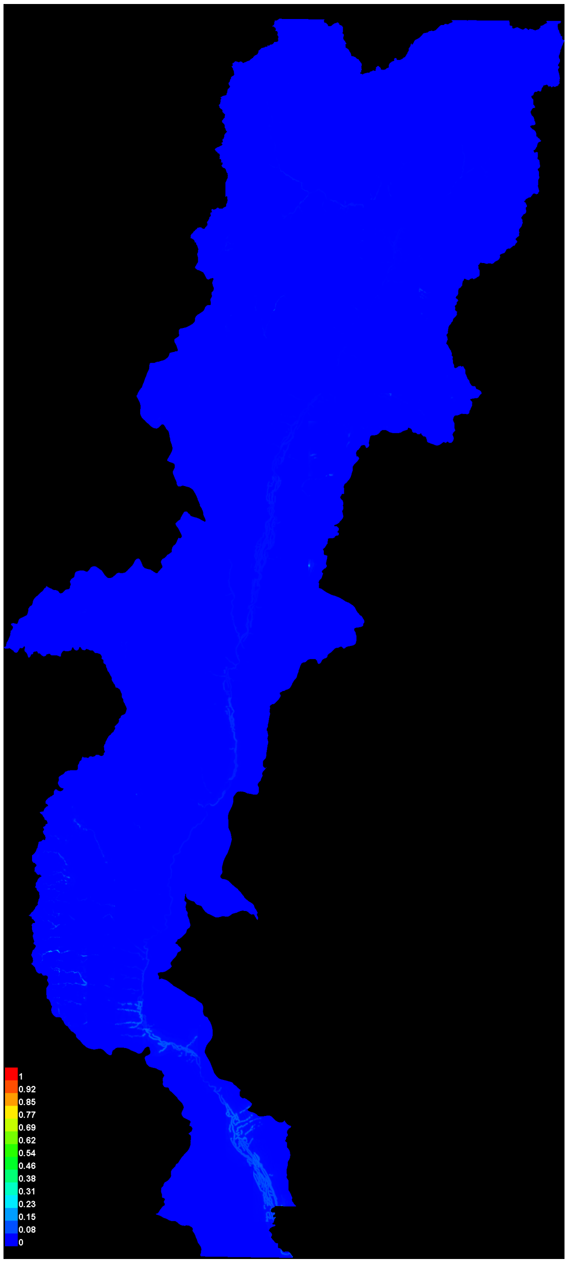

Response curves

These curves show how each environmental variable affects the Maxent prediction. The curves show how the logistic prediction changes as each environmental variable is varied, keeping all other environmental variables at their average sample value. Click on a response curve to see a larger version. Note that the curves can be hard to interpret if you have strongly correlated variables, as the model may depend on the correlations in ways that are not evident in the curves. In other words, the curves show the marginal effect of changing exactly one variable, whereas the model may take advantage of sets of variables changing together. The curves show the mean response of the 15 replicate Maxent runs (red) and and the mean +/- one standard deviation (blue, two shades for categorical variables).

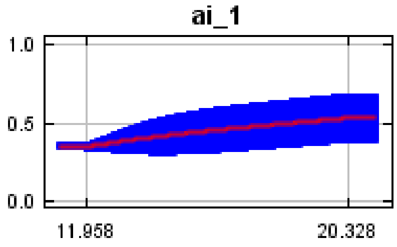

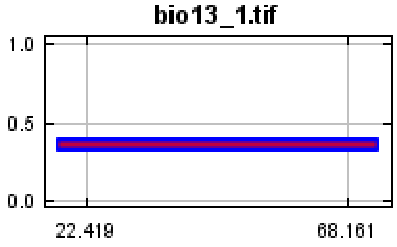

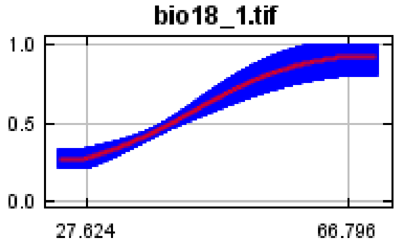

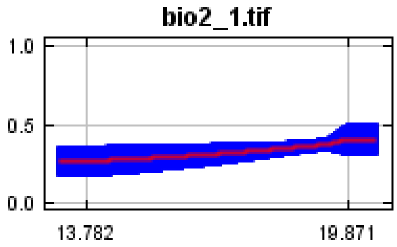

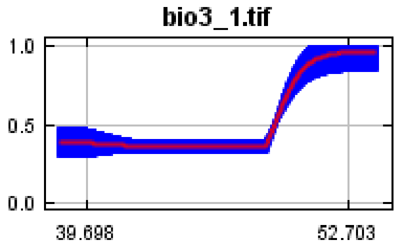

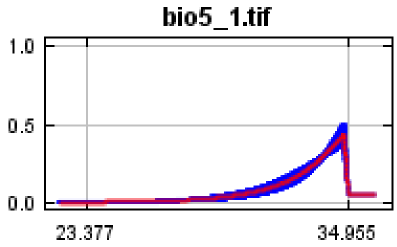

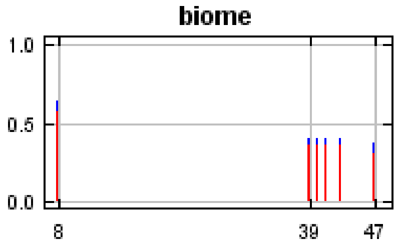

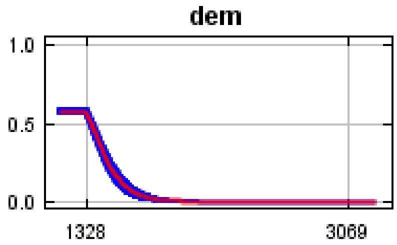

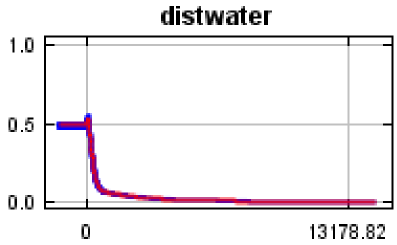

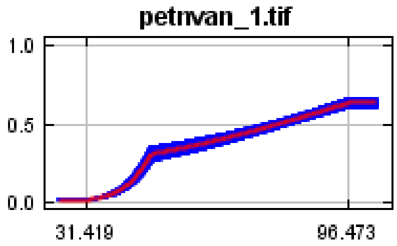

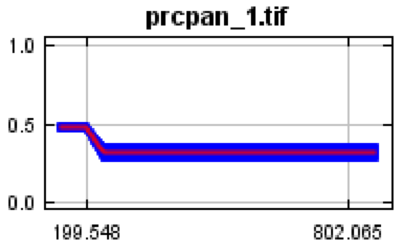

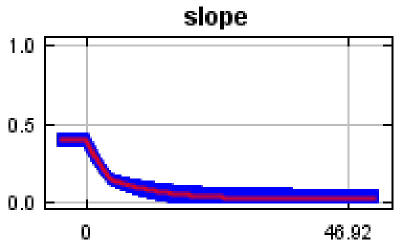

In contrast to the above marginal response curves, each of the following curves represents a different model, namely, a Maxent model created using only the corresponding variable. These plots reflect the dependence of predicted suitability both on the selected variable and on dependencies induced by correlations between the selected variable and other variables. They may be easier to interpret if there are strong correlations between variables.

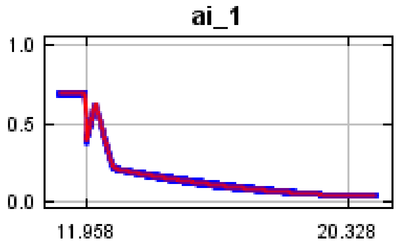

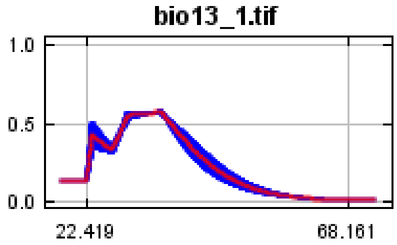

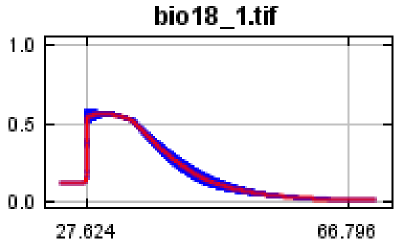

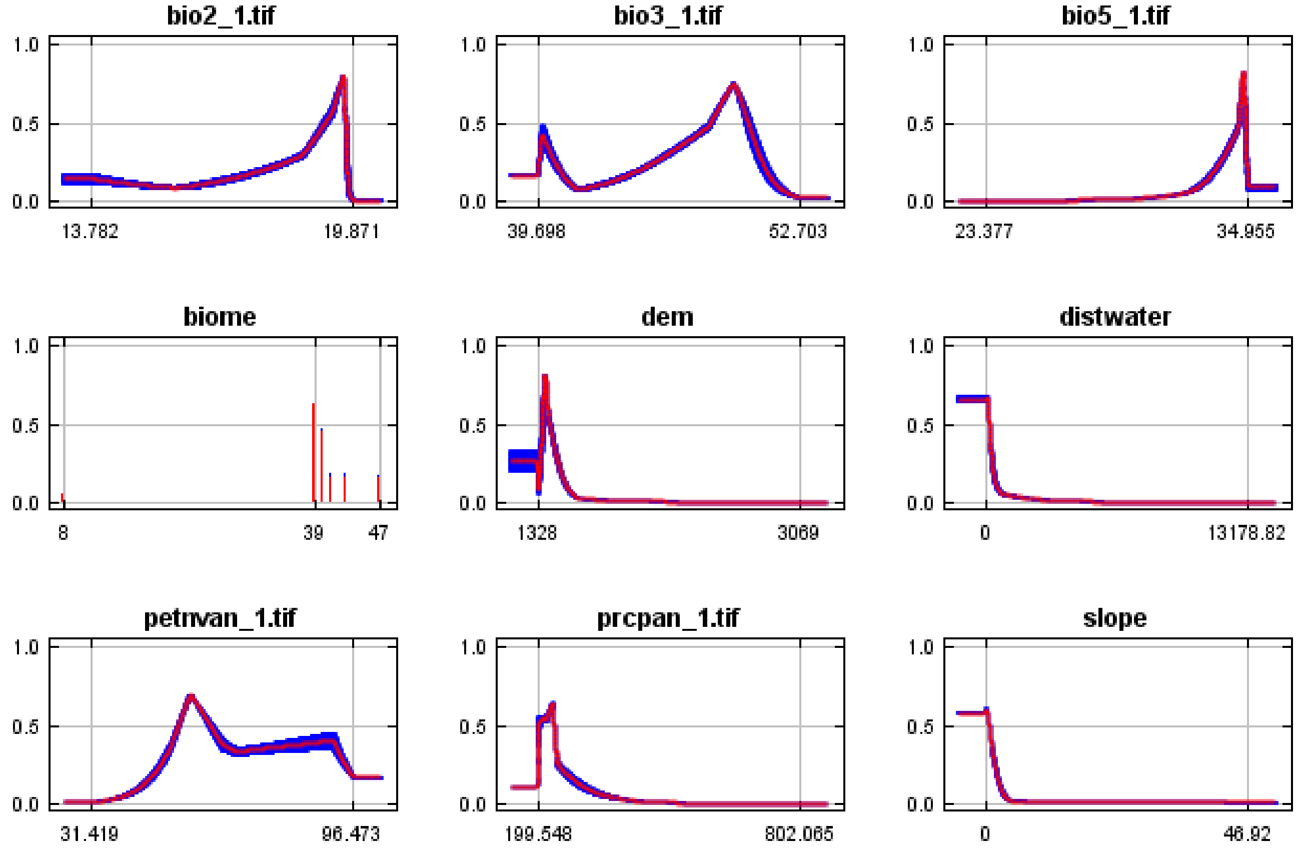

Analysis of variable contributions

The following table gives estimates of relative contributions of the environmental variables to the Maxent model. To determine the first estimate, in each iteration of the training algorithm, the increase in regularized gain is added to the contribution of the corresponding variable, or subtracted from it if the change to the absolute value of lambda is negative. For the second estimate, for each environmental variable in turn, the values of that variable on training presence and background data are randomly permuted. The model is reevaluated on the permuted data, and the resulting drop in training AUC is shown in the table, normalized to percentages. As with the variable jackknife, variable contributions should be interpreted with caution when the predictor variables are correlated. Values shown are averages over replicate runs.

Variable	Percent contribution	Permutation importance
distwater	56.1	26.9
bio2_1.tif	15.1	0.1
dem	13.4	39.3
bio5_1.tif	5.3	13.1
petnvan_1.tif	5	8.1
biome	2.7	0.2
slope	1.7	1.5
bio3_1.tif	0.6	5.3

bio18_1.tif	0.1	4.8
prcpan_1.tif	0.1	0.5
ai_1	0	0.2
bio13_1.tif	0	0

The following picture shows the results of the jackknife test of variable importance. The environmental variable with highest gain when used in isolation is distwater, which therefore appears to have the most useful information by itself. The environmental variable that decreases the gain the most when it is omitted is distwater, which therefore appears to have the most information that isn't present in the other variables. Values shown are averages over replicate runs.

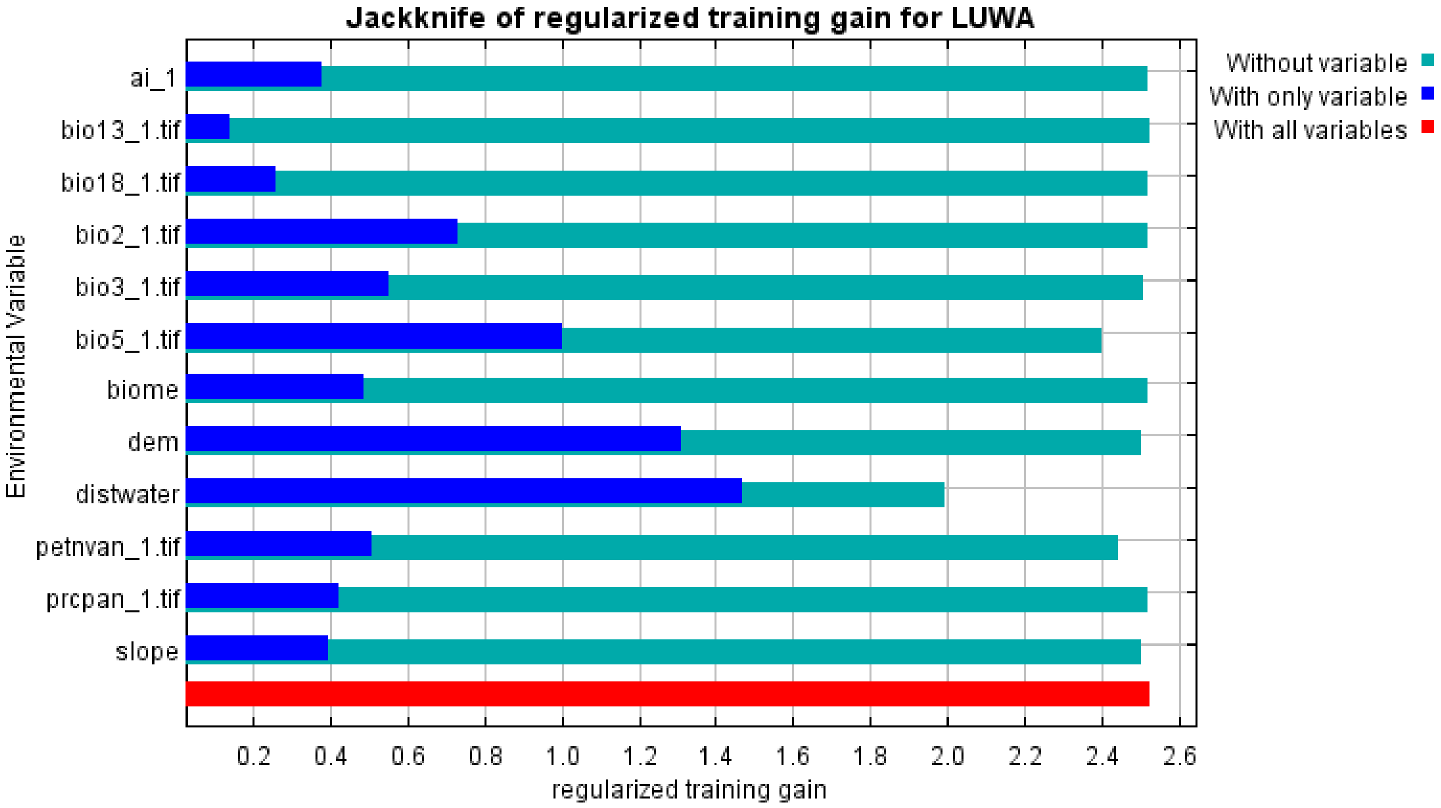

The next picture shows the same jackknife test, using test gain instead of training gain. Note that conclusions about which variables are most important can change, now that we're looking at test data.

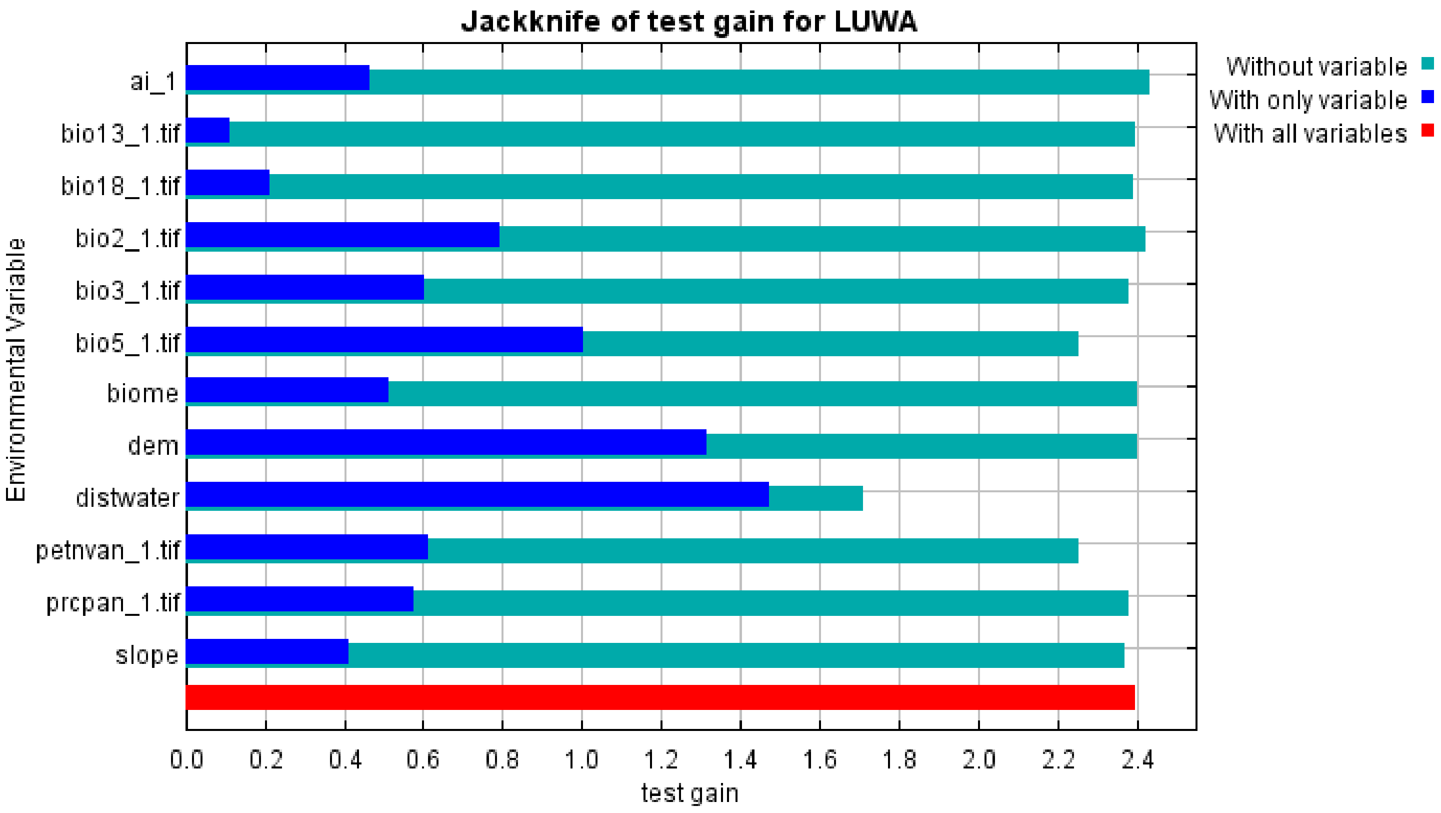

Lastly, we have the same jackknife test, using AUC on test data.

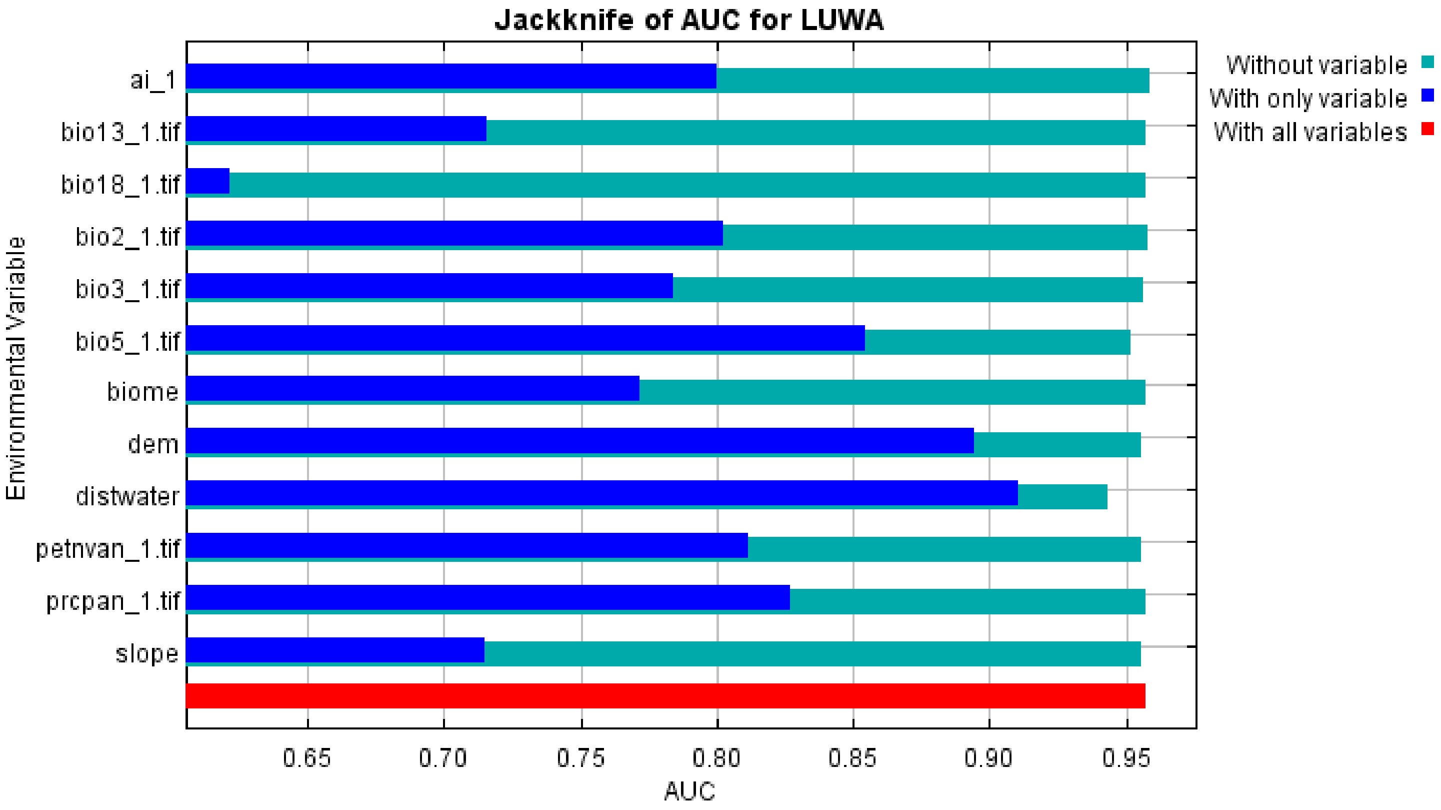

Command line to repeat this species model: java density.MaxEnt nowarnings noprefixes -E "" -E LUWA responsecurves jackknife outputdirectory=D:\MaxEnt4\BirdsCCC

"projectionlayers=D:\Ascii_Files\Envir_2030_ascii, D:\Ascii_Files\Envir_2060_ascii, D:\Ascii_Files\Envir_2090_ascii" samplesfile=D:\Data\RioGrande\Birds\allbirds.csv environmentallayers=D:\Ascii_Files\Envir_Curr_ascii randomseed noaskoverwrite biasfile=D:\Data\RioGrande\Birds\all_bias.asc replicates=15 nooutputgrids maximumiterations=5000 biastype=3 "applythresholdrule=equal training sensitivity and specificity" -N ai_2 -N ai_3 -N bio10_1 -N bio10_1.tif -N bio10_2 -N bio10_2.tif -N bio10_3 -N bio10_3.tif -N bio11_1 -N bio11_1.tif -N bio11_2 -N bio11_2.tif -N bio11_3 -N bio11_3.tif -N bio13_2.tif -N bio13_3.tif -N bio14_1.tif -N bio14_2.tif -N bio14_3.tif -N bio16_1.tif -N bio16_2.tif -N bio16_3.tif -N bio17_.tif -N bio17_2.tif -N bio17_3.tif -N bio17_all.tif -N bio18_2.tif -N bio18_3.tif -N bio19_1.tif -N bio19_2.tif -N bio19_3.tif -N bio2_2.tif -N bio2_3.tif -N bio3_2.tif -N bio3_3.tif -N bio5_2.tif -N bio5_3.tif -N bio6_1.tif -N bio6_2.tif -N bio6_3.tif -N bio7_1.tif -N bio7_2.tif -N bio7_3.tif -N etan_1.tif -N etan_2.tif -N etan_3.tif -N etdjf_1.tif -N etdjf_2.tif -N etdjf_3.tif -N etjas_1.tif -N etjas_2.tif -N etjas_3.tif -N etjfm_1.tif -N etjfm_2.tif -N etjfm_3.tif -N etjja_1.tif -N etjja_2.tif -N etjja_3.tif -N etjja_all.tif -N petnvan_2.tif -N petnvan_3.tif -N petnvdjf_1.tif -N petnvdjf_2.tif -N petnvdjf_3.tif -N petnvjas_1.tif -N petnvjas_2.tif -N petnvjas_3.tif -N petnvjfm_1.tif -N petnvjfm_2.tif -N petnvjfm_3.tif -N petnvjja_1.tif -N petnvjja_2.tif -N petnvjja_3.tif -N petwan_1.tif -N petwan_2.tif -N petwan_3.tif -N petwdjf_1.tif -N petwdjf_2.tif -N petwdjf_3.tif -N petwjas_1.tif -N petwjas_2.tif -N petwjas_3.tif -N petwjas_all.tif -N petwjfm_1.tif -N petwjfm_2.tif -N petwjfm_3.tif -N petwjja_1.tif -N petwjja_2.tif -N petwjja_3.tif -N prcpan_2.tif -N prcpan_3.tif -N prcpan_all.tif -N runoffan_1.tif -N runoffan_2.tif -N runoffan_3.tif -N runoffdjf_1.tif -N runoffdjf_2.tif -N runoffdjf_3.tif -N runoffjas_1.tif -N runoffjas_2.tif -N runoffjas_3.tif -N runoffjas_all.tif -N runoffjfm_1.tif -N runoffjfm_2.tif -N runoffjfm_3.tif -N runoffjja_1.tif -N runoffjja_2.tif -N runoffjja_3.tif -N smcan_1.tif -N smcan_2.tif -N smcan_3.tif -N smcdjf_1.tif -N smcdjf_2.tif -N smcdjf_3.tif -N smcjas_1.tif -N smcjas_2.tif -N smcjas_3.tif -N smcjfm_1.tif -N smcjfm_2.tif -N smcjfm_3.tif -N smcjja_1.tif -N smcjja_2.tif -N smcjja_3.tif -N swean_1.tif -N swean_2.tif -N swean_3.tif -N swedjf_1.tif -N swedjf_2.tif -N swedjf_3.tif -N swejfm_1.tif -N swejfm_2.tif -N swejfm_3.tif -N tave_1.tif -N tave_2.tif -N tave_3.tif -N tmax_1.tif -N tmax_2.tif -N tmax_3.tif -N tmin_1.tif -N tmin_2.tif -N tmin_3.tif -t biome

Replicated maxent model for LUWA

This page summarizes the results of 15-fold cross-validation for LUWA, created Mon Aug 04 15:10:32 MDT 2014 using Maxent version 3.3.3k. The individual models are here: [0] [1] [2] [3] [4] [5] [6] [7] [8] [9] [10] [11] [12] [13] [14]

Analysis of omission/commission

The following picture shows the test omission rate and predicted area as a function of the cumulative threshold, averaged over the replicate runs. The omission rate should be close to the predicted omission, because of the definition of the cumulative threshold.

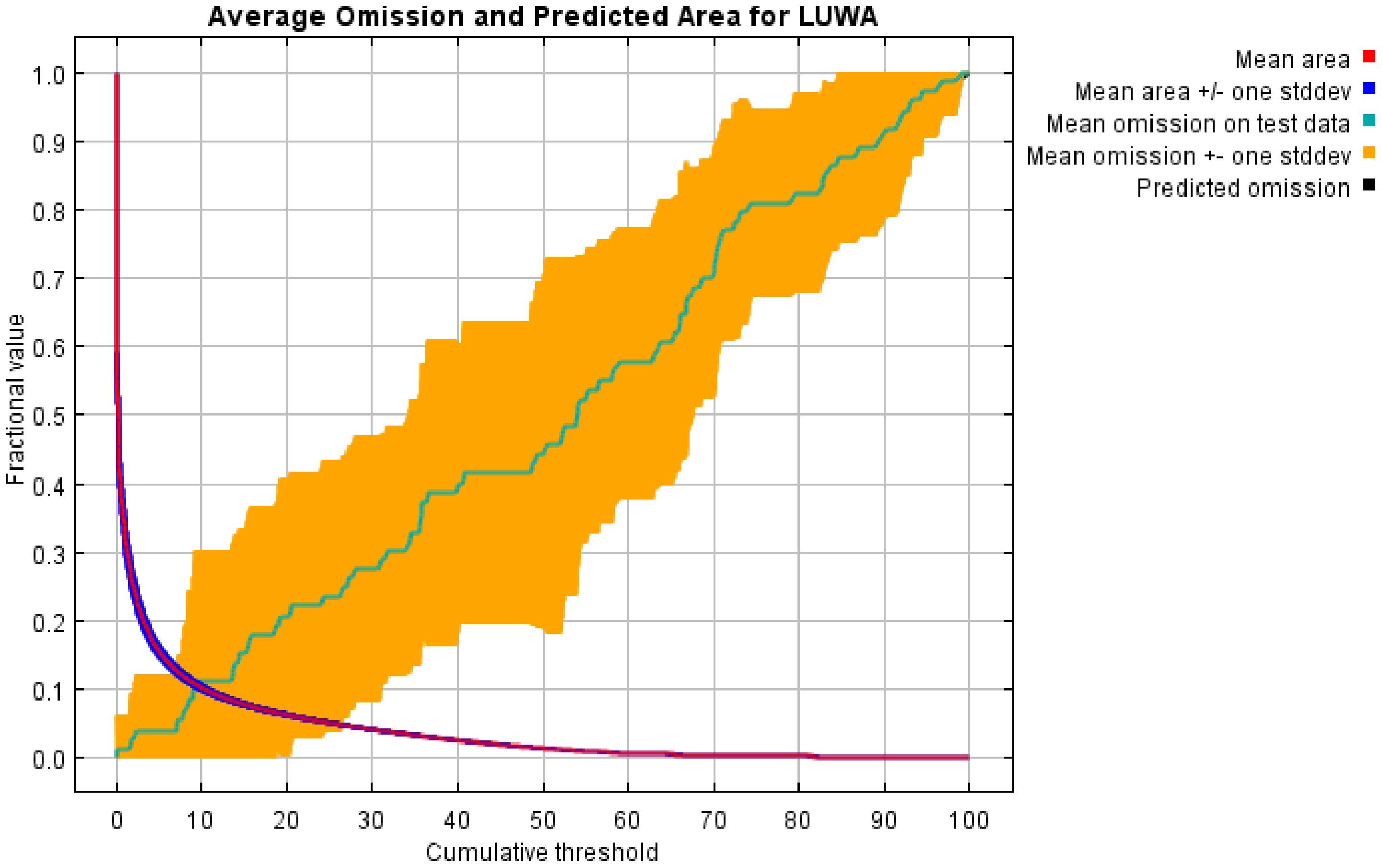

The next picture is the receiver operating characteristic (ROC) curve for the same data, again averaged over the replicate runs. Note that the specificity is defined using predicted area, rather than true commission (see the paper by Phillips, Anderson and Schapire cited on the help page for discussion of what this means). The average test AUC for the replicate runs is 0.955, and the standard deviation is 0.043.

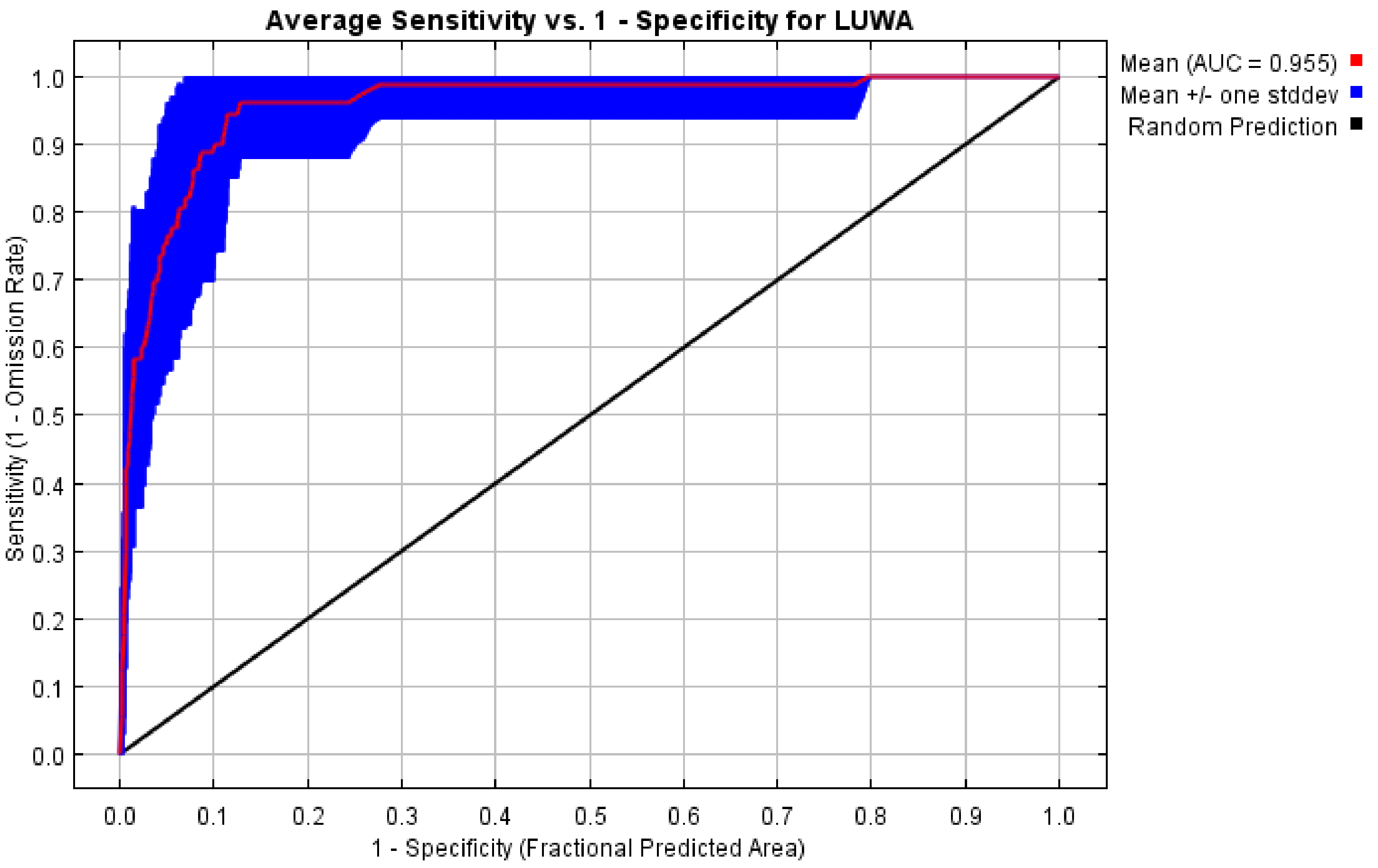

Pictures of the model

The following two pictures show the point-wise mean and standard deviation of the 15 output grids. Other available summary grids are min, max and median.

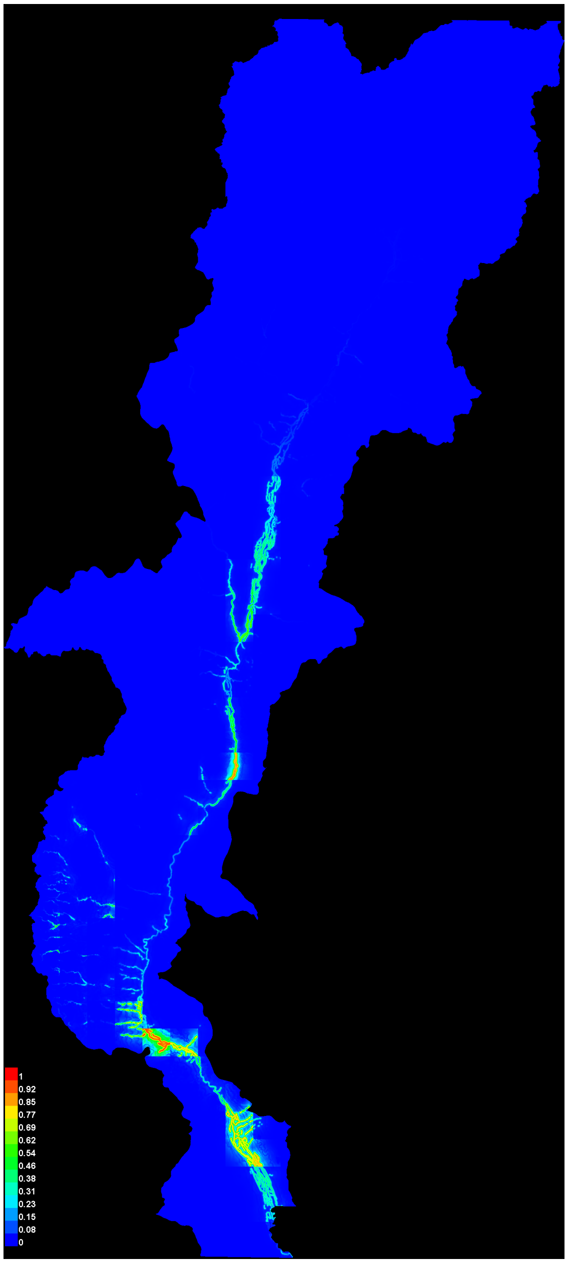

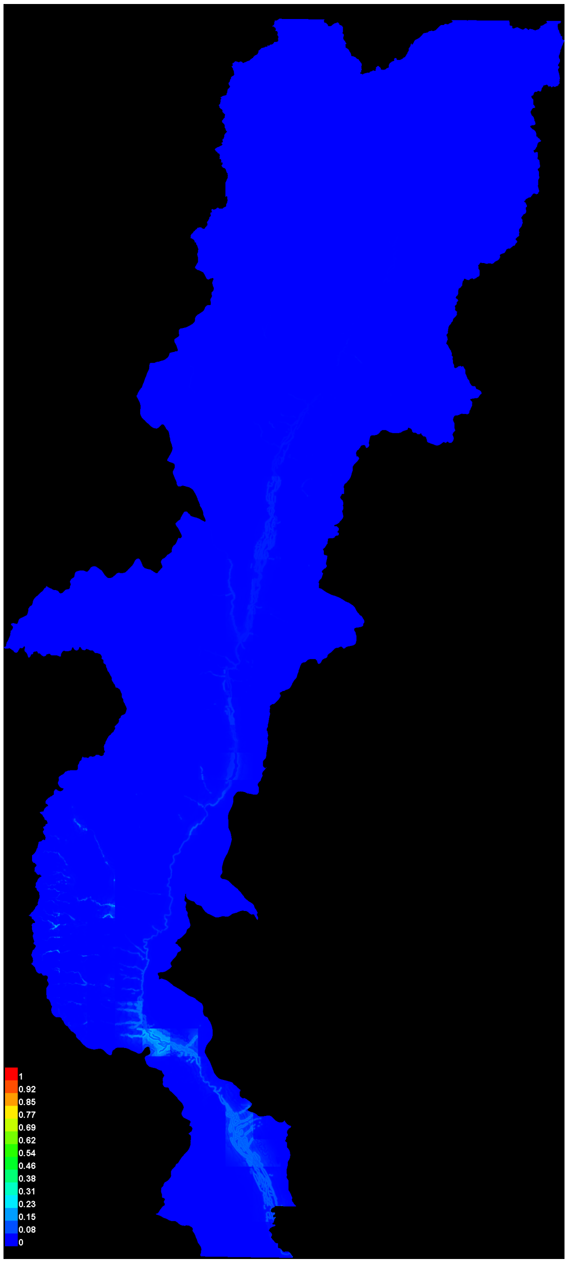

The following two pictures show the point-wise mean and standard deviation of the 15 models applied to the environmental layers in Envir_2030_ascii. Other available summary grids are min, max and median.

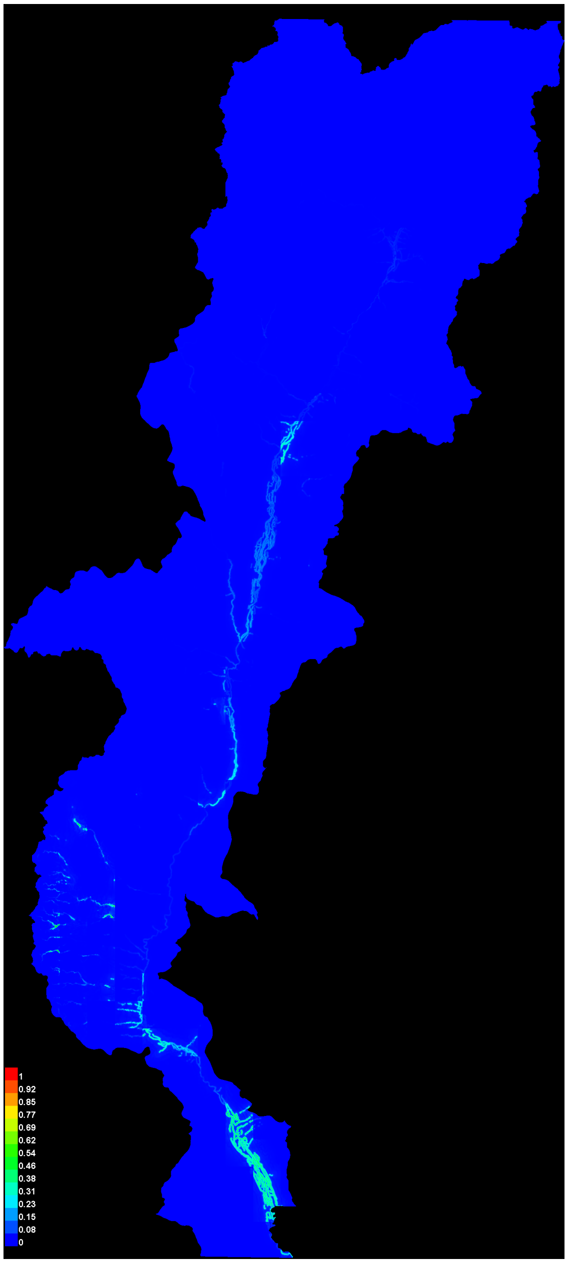

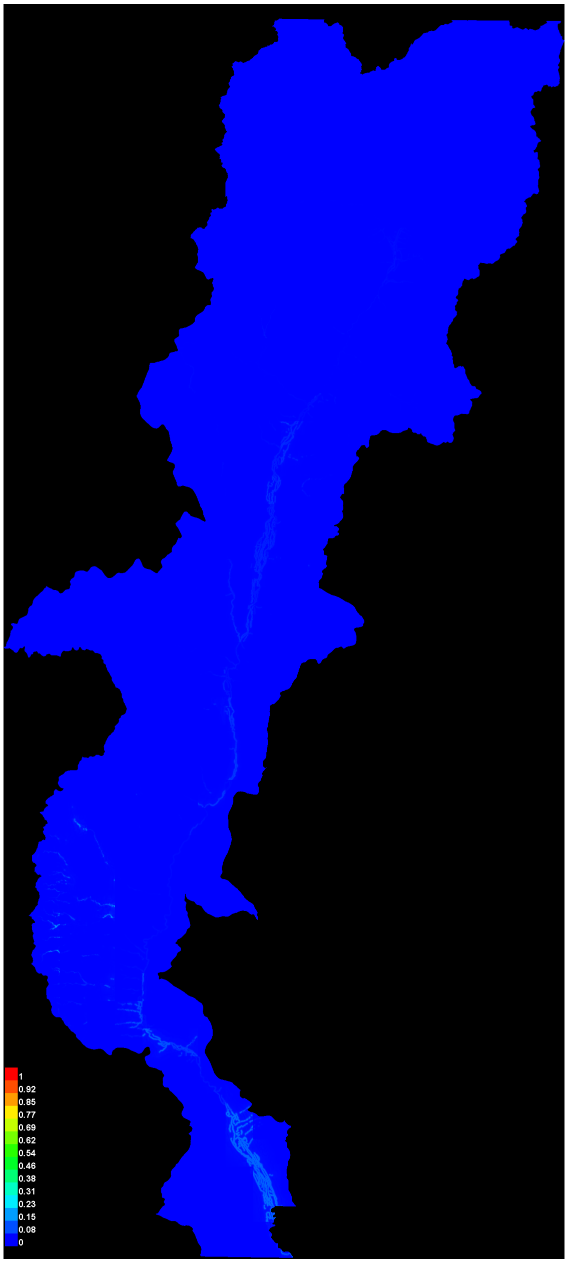

The following two pictures show the point-wise mean and standard deviation of the 15 models applied to the environmental layers in Envir_2060_ascii. Other available summary grids are min, max and median.

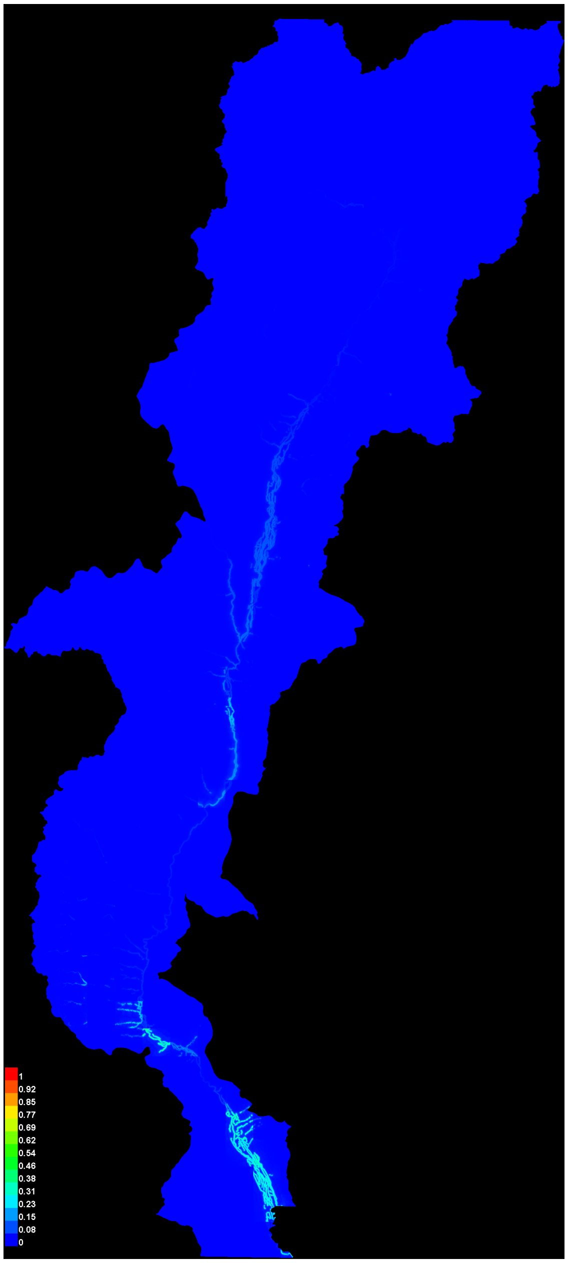

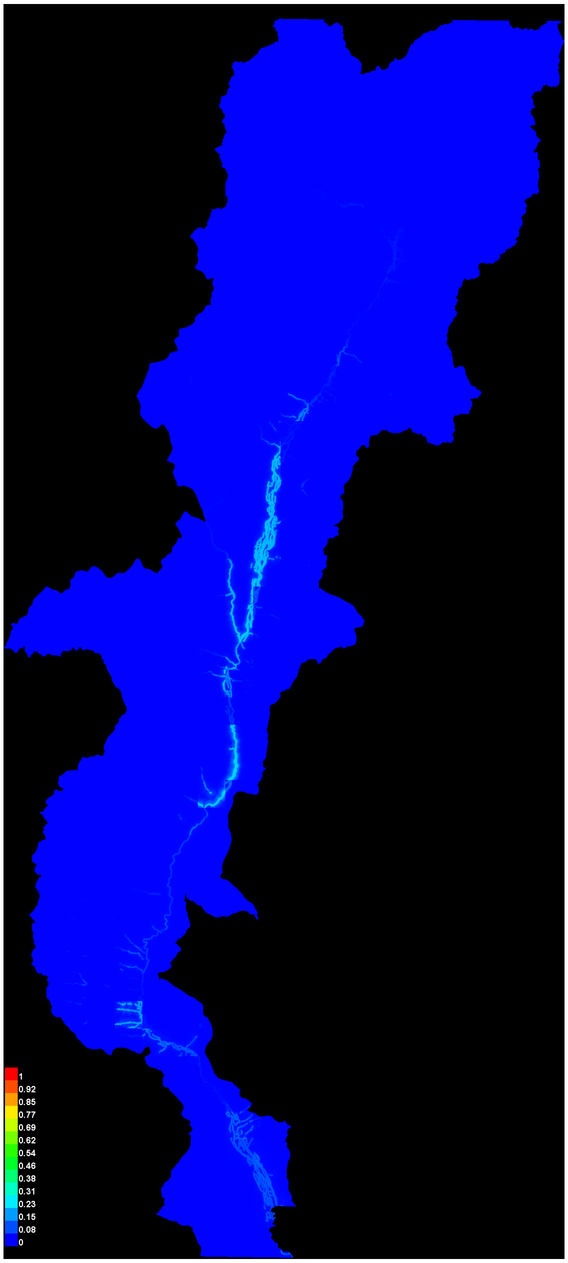

The following two pictures show the point-wise mean and standard deviation of the 15 models applied to the environmental layers in Envir_2090_ascii. Other available summary grids are min, max and median.

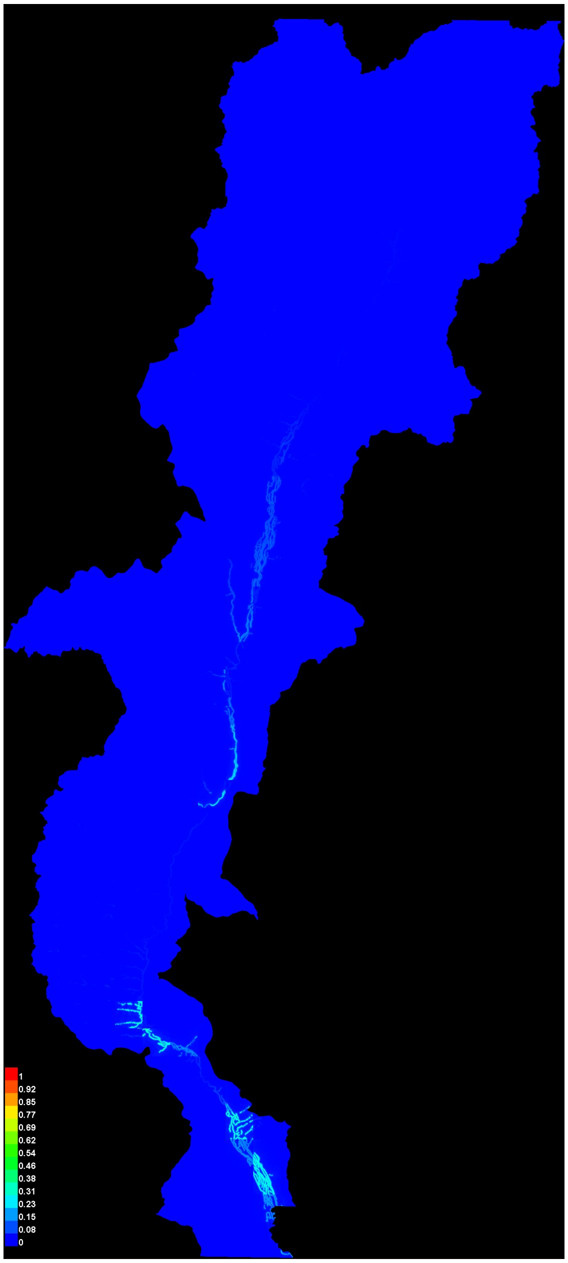

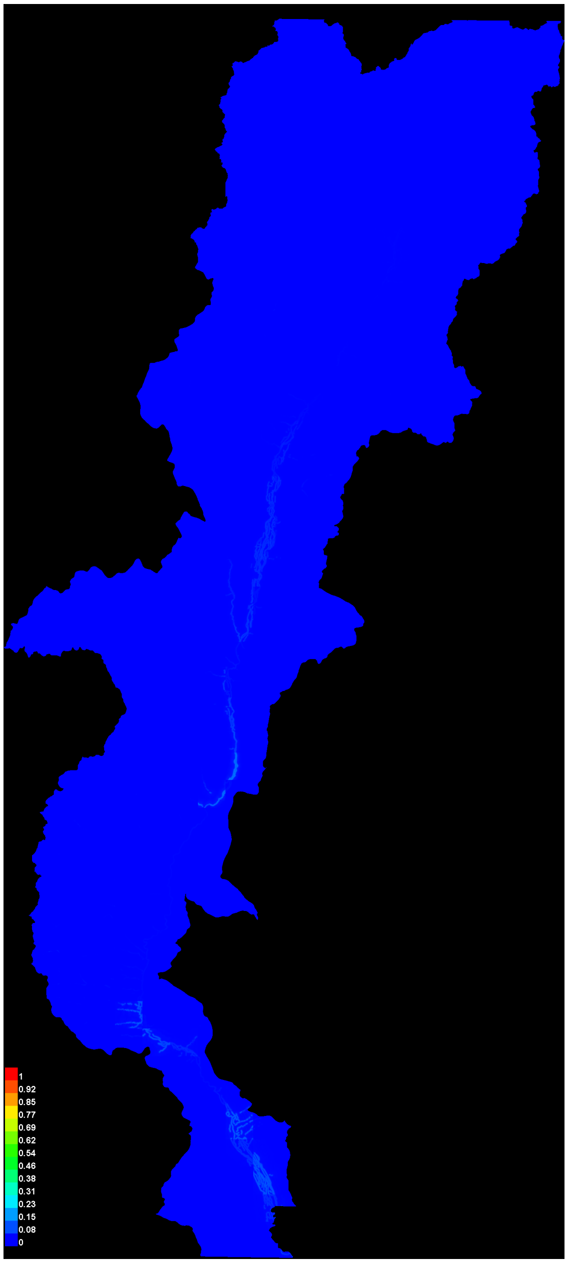

Response curves

These curves show how each environmental variable affects the Maxent prediction. The curves show how the logistic prediction changes as each environmental variable is varied, keeping all other environmental variables at their average sample value. Click on a response curve to see a larger version. Note that the curves can be hard to interpret if you have strongly correlated variables, as the model may depend on the correlations in ways that are not evident in the curves. In other words, the curves show the marginal effect of changing exactly one variable, whereas the model may take advantage of sets of variables changing together. The curves show the mean response of the 15 replicate Maxent runs (red) and and the mean +/- one standard deviation (blue, two shades for categorical variables).

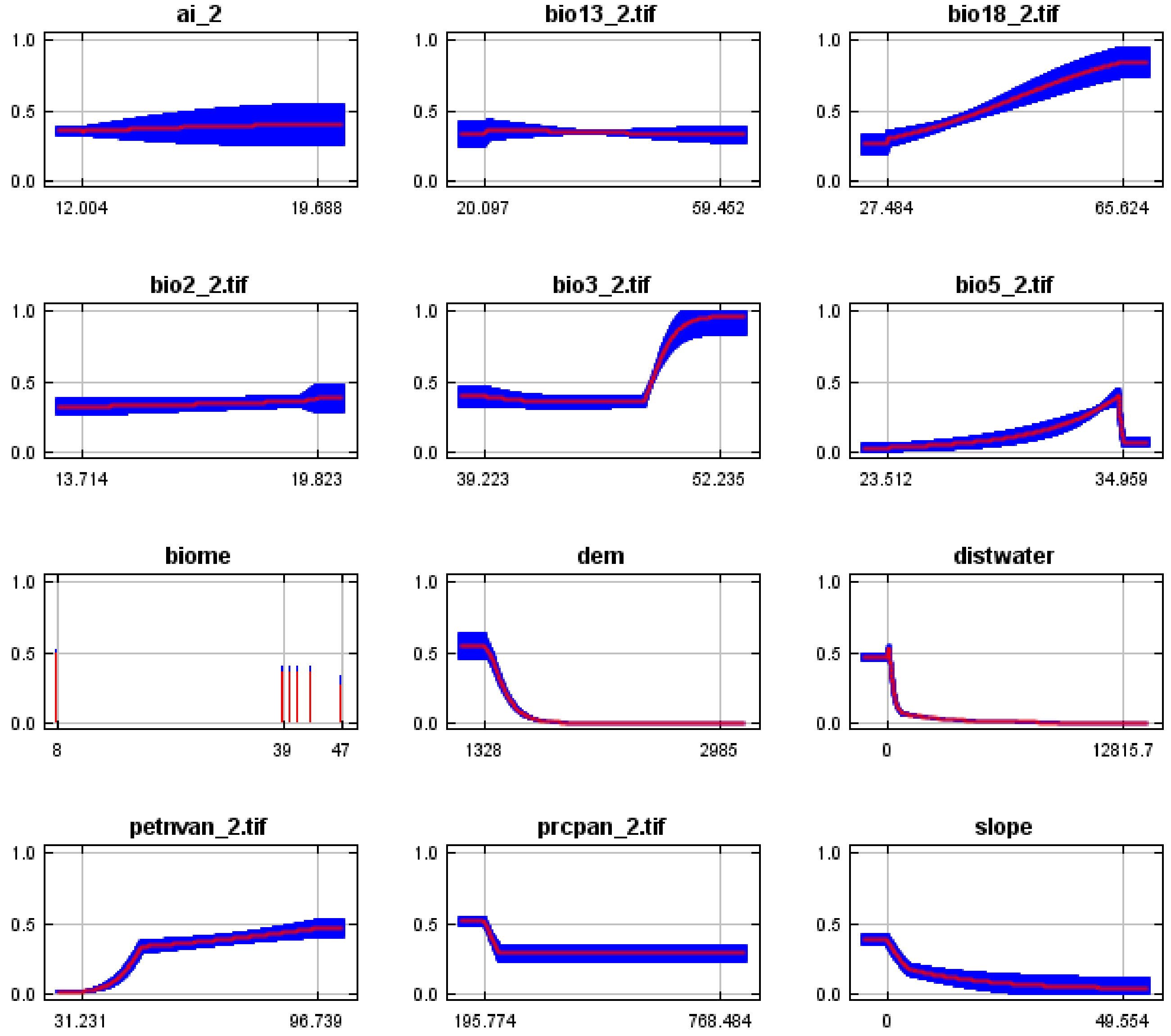

In contrast to the above marginal response curves, each of the following curves represents a different model, namely, a Maxent model created using only the corresponding variable. These plots reflect the dependence of predicted suitability both on the selected variable and on dependencies induced by correlations between the selected variable and other variables. They may be easier to interpret if there are strong correlations between variables.

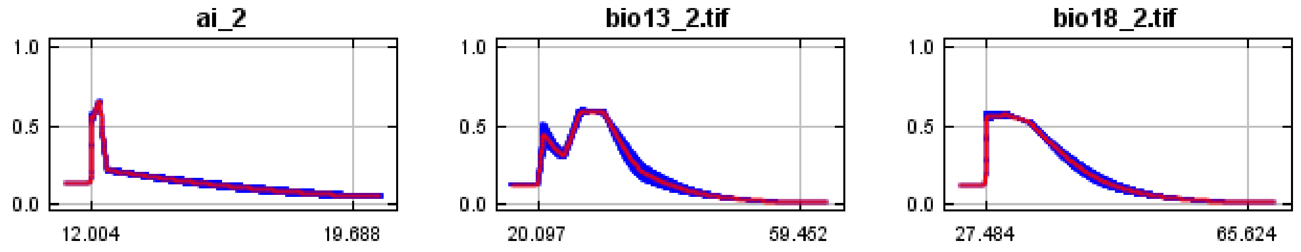

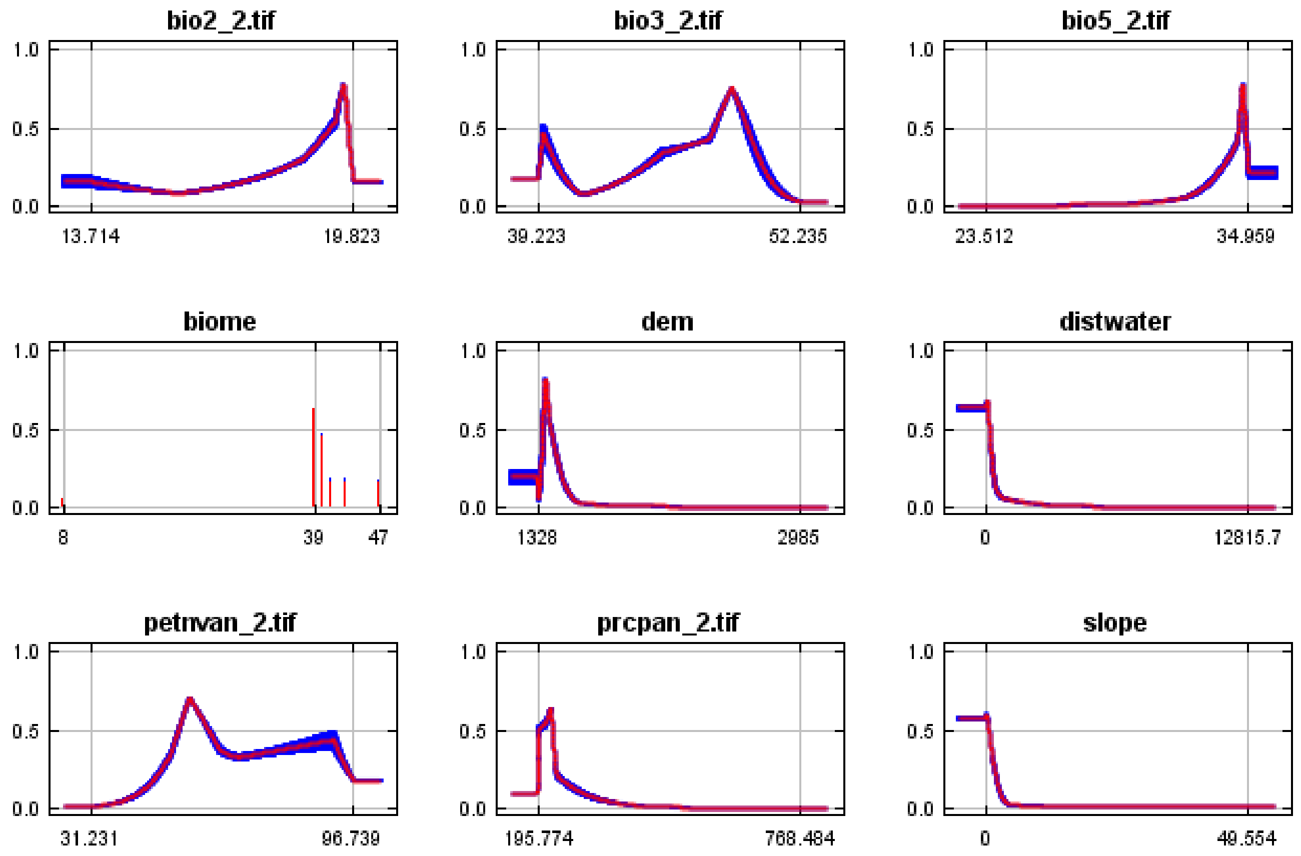

Analysis of variable contributions

The following table gives estimates of relative contributions of the environmental variables to the Maxent model. To determine the first estimate, in each iteration of the training algorithm, the increase in regularized gain is added to the contribution of the corresponding variable, or subtracted from it if the change to the absolute value of lambda is negative. For the second estimate, for each environmental variable in turn, the values of that variable on training presence and background data are randomly permuted. The model is reevaluated on the permuted data, and the resulting drop in training AUC is shown in the table, normalized to percentages. As with the variable jackknife, variable contributions should be interpreted with caution when the predictor variables are correlated. Values shown are averages over replicate runs.

Variable	Percent contribution	Permutation importance
distwater	56.7	28.5
bio2_2.tif	14.1	0
dem	12.5	47.2
bio5_2.tif	6.9	4.4
petnvan_2.tif	4.9	7.4
biome	3.1	1.1
slope	1.1	2.6
bio3_2.tif	0.6	5.7

prcpan_2.tif	0.2	0.8
bio18_2.tif	0	2
ai_2	0	0.1
bio13_2.tif	0	0.1

The following picture shows the results of the jackknife test of variable importance. The environmental variable with highest gain when used in isolation is distwater, which therefore appears to have the most useful information by itself. The environmental variable that decreases the gain the most when it is omitted is distwater, which therefore appears to have the most information that isn't present in the other variables. Values shown are averages over replicate runs.

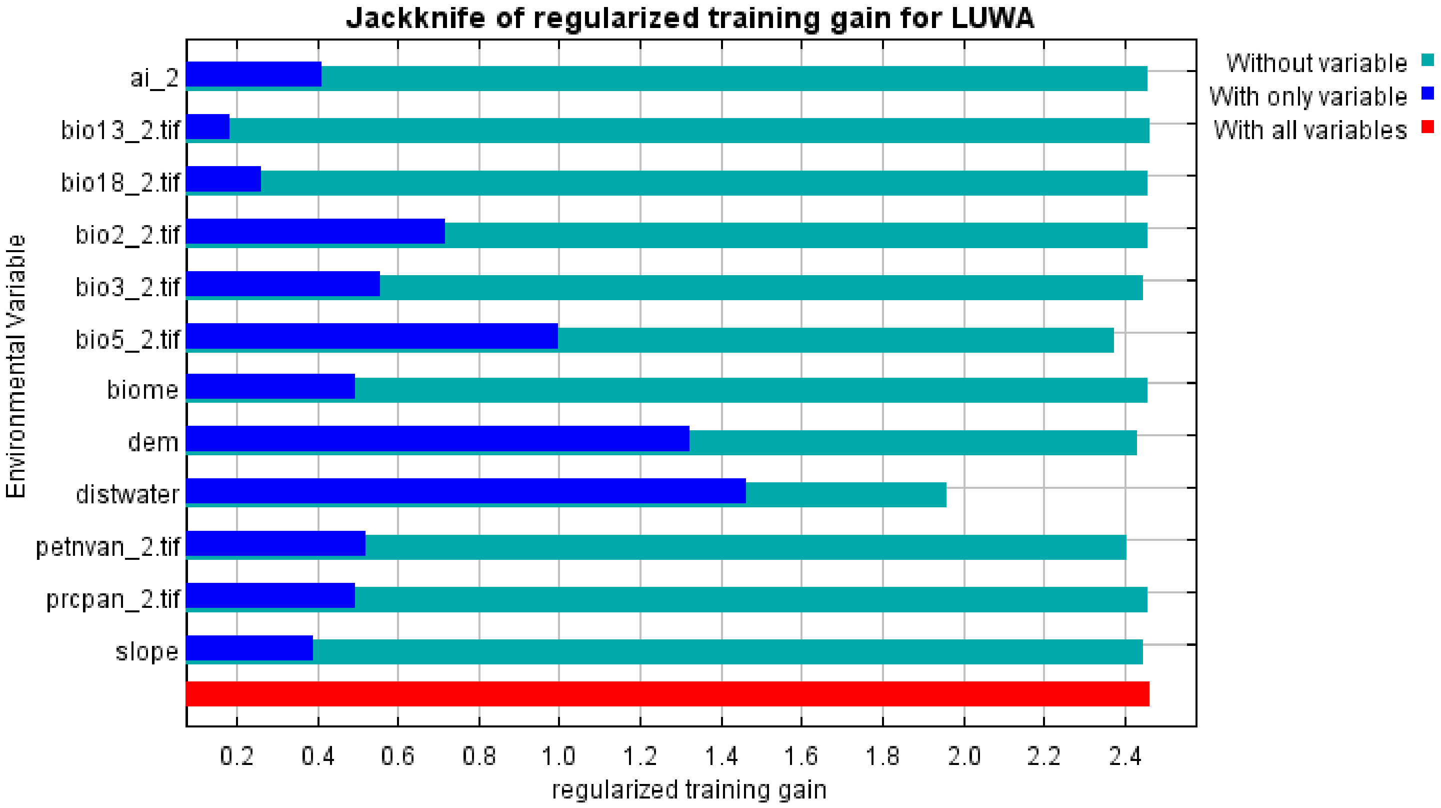

The next picture shows the same jackknife test, using test gain instead of training gain. Note that conclusions about which variables are most important can change, now that we're looking at test data.

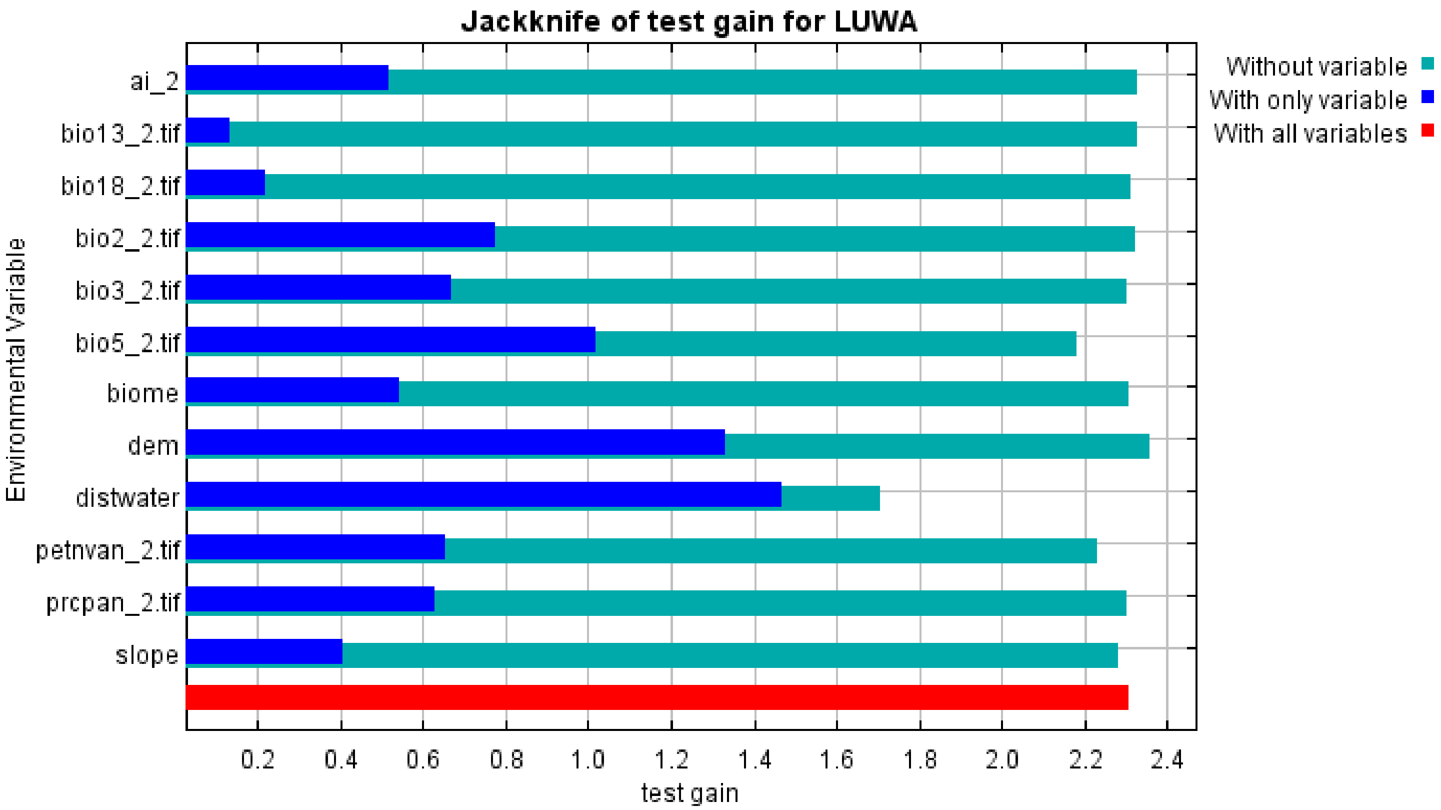

Lastly, we have the same jackknife test, using AUC on test data.

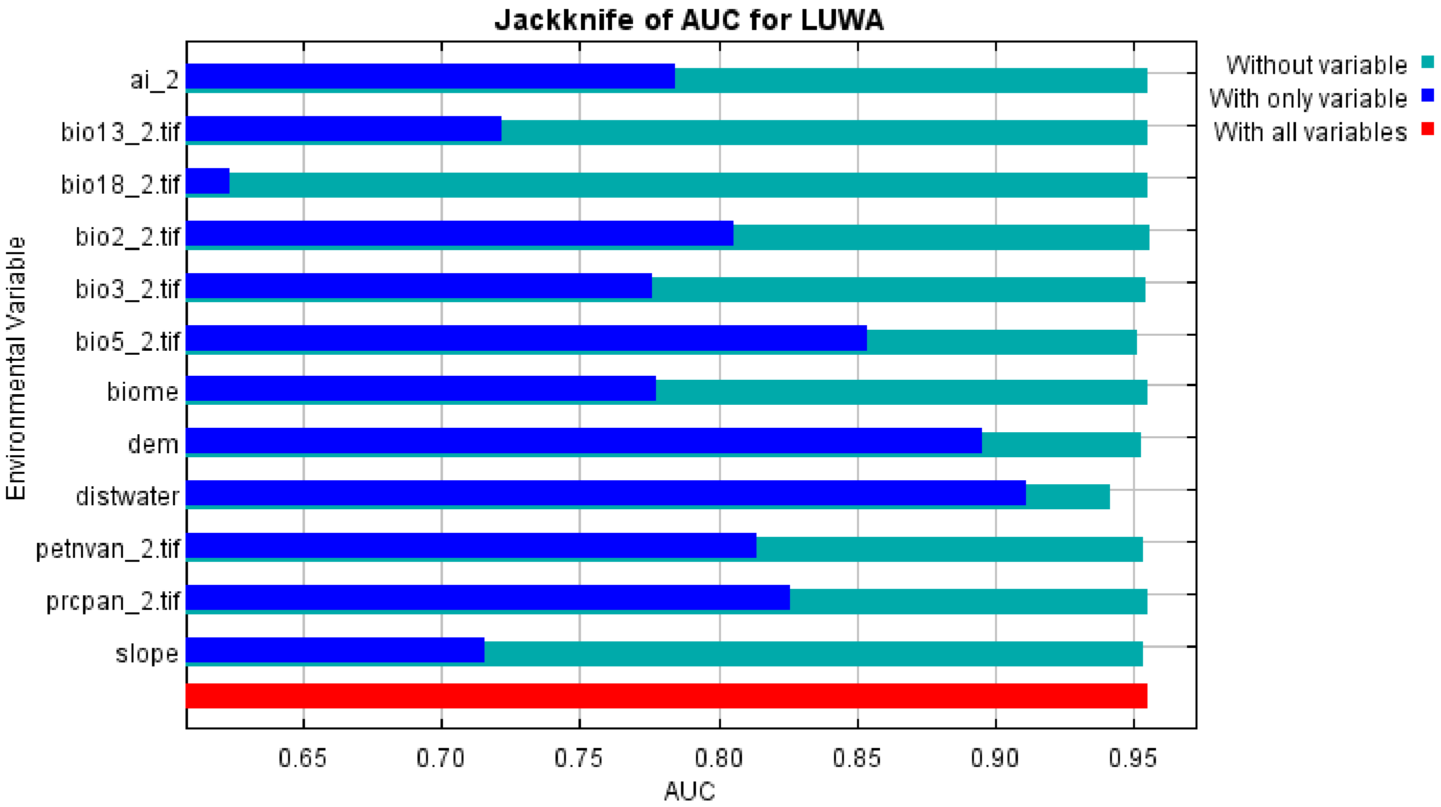

Command line to repeat this species model: java density.MaxEnt nowarnings noprefixes -E "" -E LUWA responsecurves jackknife outputdirectory=D:\MaxEnt4\BirdsGd

"projectionlayers=D:\Ascii_Files\Envir_2030_ascii, D:\Ascii_Files\Envir_2060_ascii, D:\Ascii_Files\Envir_2090_ascii" samplesfile=D:\Data\RioGrande\Birds\allbirds.csv environmentallayers=D:\Ascii_Files\Envir_Curr_ascii randomseed biasfile=D:\Data\RioGrande\Birds\all_bias.asc replicates=15 nooutputgrids maximumiterations=5000 biastype=3 "applythresholdrule=equal training sensitivity and specificity" -N ai_1 -N ai_3 -N bio10_1 -N bio10_1.tif -N bio10_2 -N bio10_2.tif -N bio10_3 -N bio10_3.tif -N bio11_1 -N bio11_1.tif -N bio11_2 -N bio11_2.tif -N bio11_3 -N bio11_3.tif -N bio13_1.tif -N bio13_3.tif -N bio14_1.tif -N bio14_2.tif -N bio14_3.tif -N bio16_1.tif -N bio16_2.tif -N bio16_3.tif -N bio17_.tif -N bio17_2.tif -N bio17_3.tif -N bio17_all.tif -N bio18_1.tif -N bio18_3.tif -N bio19_1.tif -N bio19_2.tif -N bio19_3.tif -N bio2_1.tif -N bio2_3.tif -N bio3_1.tif -N bio3_3.tif -N bio5_1.tif -N bio5_3.tif -N bio6_1.tif -N bio6_2.tif -N bio6_3.tif -N bio7_1.tif -N bio7_2.tif -N bio7_3.tif -N etan_1.tif -N etan_2.tif -N etan_3.tif -N etdjf_1.tif -N etdjf_2.tif -N etdjf_3.tif -N etjas_1.tif -N etjas_2.tif -N etjas_3.tif -N etjfm_1.tif -N etjfm_2.tif -N etjfm_3.tif -N etjja_1.tif -N etjja_2.tif -N etjja_3.tif -N etjja_all.tif -N petnvan_1.tif -N petnvan_3.tif -N petnvdjf_1.tif -N petnvdjf_2.tif -N petnvdjf_3.tif -N petnvjas_1.tif -N petnvjas_2.tif -N petnvjas_3.tif -N petnvjfm_1.tif -N petnvjfm_2.tif -N petnvjfm_3.tif -N petnvjja_1.tif -N petnvjja_2.tif -N petnvjja_3.tif -N petwan_1.tif -N petwan_2.tif -N petwan_3.tif -N petwdjf_1.tif -N petwdjf_2.tif -N petwdjf_3.tif -N petwjas_1.tif -N petwjas_2.tif -N petwjas_3.tif -N petwjas_all.tif -N petwjfm_1.tif -N petwjfm_2.tif -N petwjfm_3.tif -N petwjja_1.tif -N petwjja_2.tif -N petwjja_3.tif -N prcpan_1.tif -N prcpan_3.tif -N prcpan_all.tif -N runoffan_1.tif -N runoffan_2.tif -N runoffan_3.tif -N runoffdjf_1.tif -N runoffdjf_2.tif -N runoffdjf_3.tif -N runoffjas_1.tif -N runoffjas_2.tif -N runoffjas_3.tif -N runoffjas_all.tif -N runoffjfm_1.tif -N runoffjfm_2.tif -N runoffjfm_3.tif -N runoffjja_1.tif -N runoffjja_2.tif -N runoffjja_3.tif -N smcan_1.tif -N smcan_2.tif -N smcan_3.tif -N smcdjf_1.tif -N smcdjf_2.tif -N smcdjf_3.tif -N smcjas_1.tif -N smcjas_2.tif -N smcjas_3.tif -N smcjfm_1.tif -N smcjfm_2.tif -N smcjfm_3.tif -N smcjja_1.tif -N smcjja_2.tif -N smcjja_3.tif -N swean_1.tif -N swean_2.tif -N swean_3.tif -N swedjf_1.tif -N swedjf_2.tif -N swedjf_3.tif -N swejfm_1.tif -N swejfm_2.tif -N swejfm_3.tif -N tave_1.tif -N tave_2.tif -N tave_3.tif -N tmax_1.tif -N tmax_2.tif -N tmax_3.tif -N tmin_1.tif -N tmin_2.tif -N tmin_3.tif -t biome

Replicated maxent model for LUWA

This page summarizes the results of 15-fold cross-validation for LUWA, created Wed Aug 06 09:16:30 MDT 2014 using Maxent version 3.3.3k. The individual models are here: [0] [1] [2] [3] [4] [5] [6] [7] [8] [9] [10] [11] [12] [13] [14]

Analysis of omission/commission

The following picture shows the test omission rate and predicted area as a function of the cumulative threshold, averaged over the replicate runs. The omission rate should be close to the predicted omission, because of the definition of the cumulative threshold.

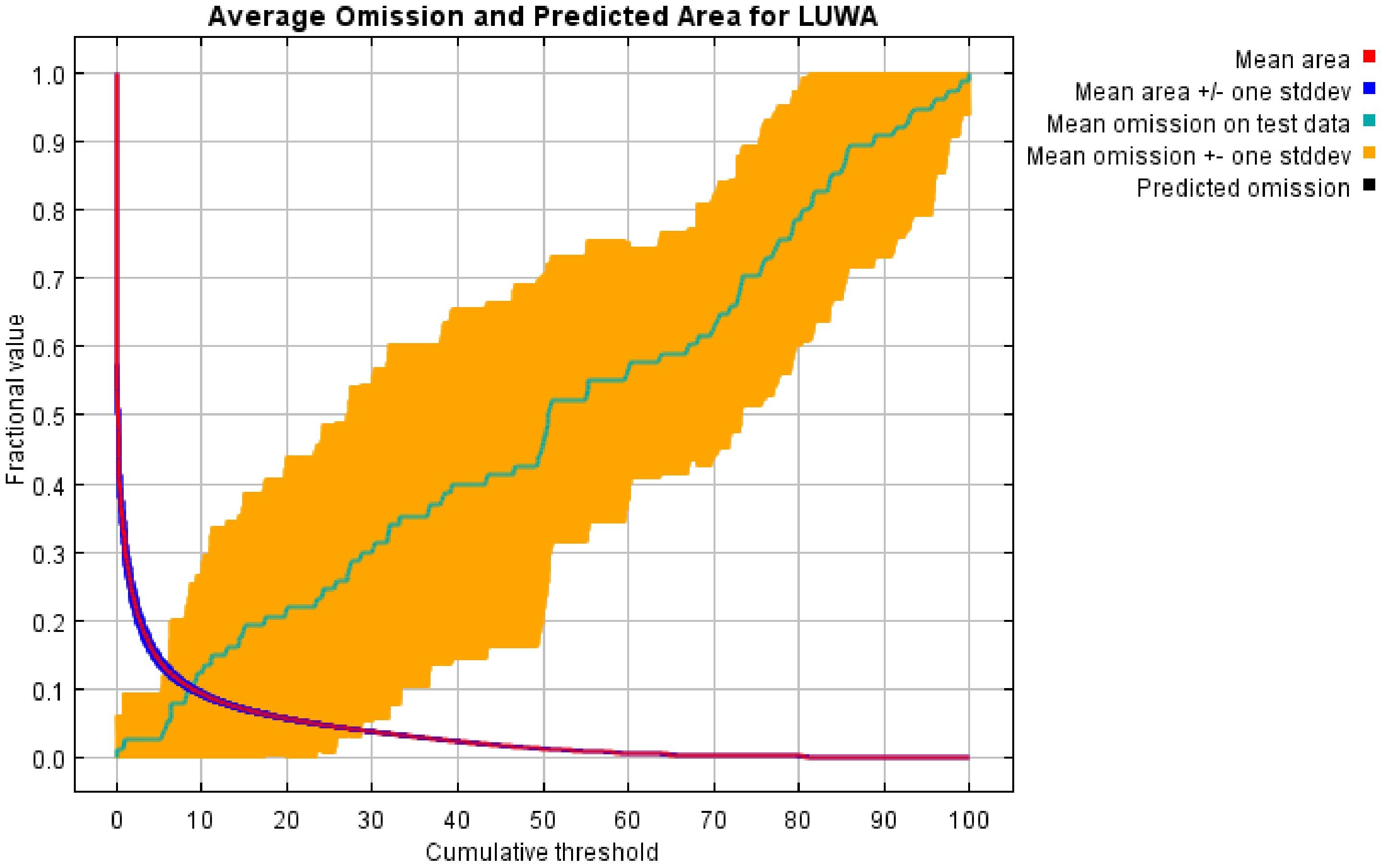

The next picture is the receiver operating characteristic (ROC) curve for the same data, again averaged over the replicate runs. Note that the specificity is defined using predicted area, rather than true commission (see the paper by Phillips, Anderson and Schapire cited on the help page for discussion of what this means). The average test AUC for the replicate runs is 0.957, and the standard deviation is 0.040.

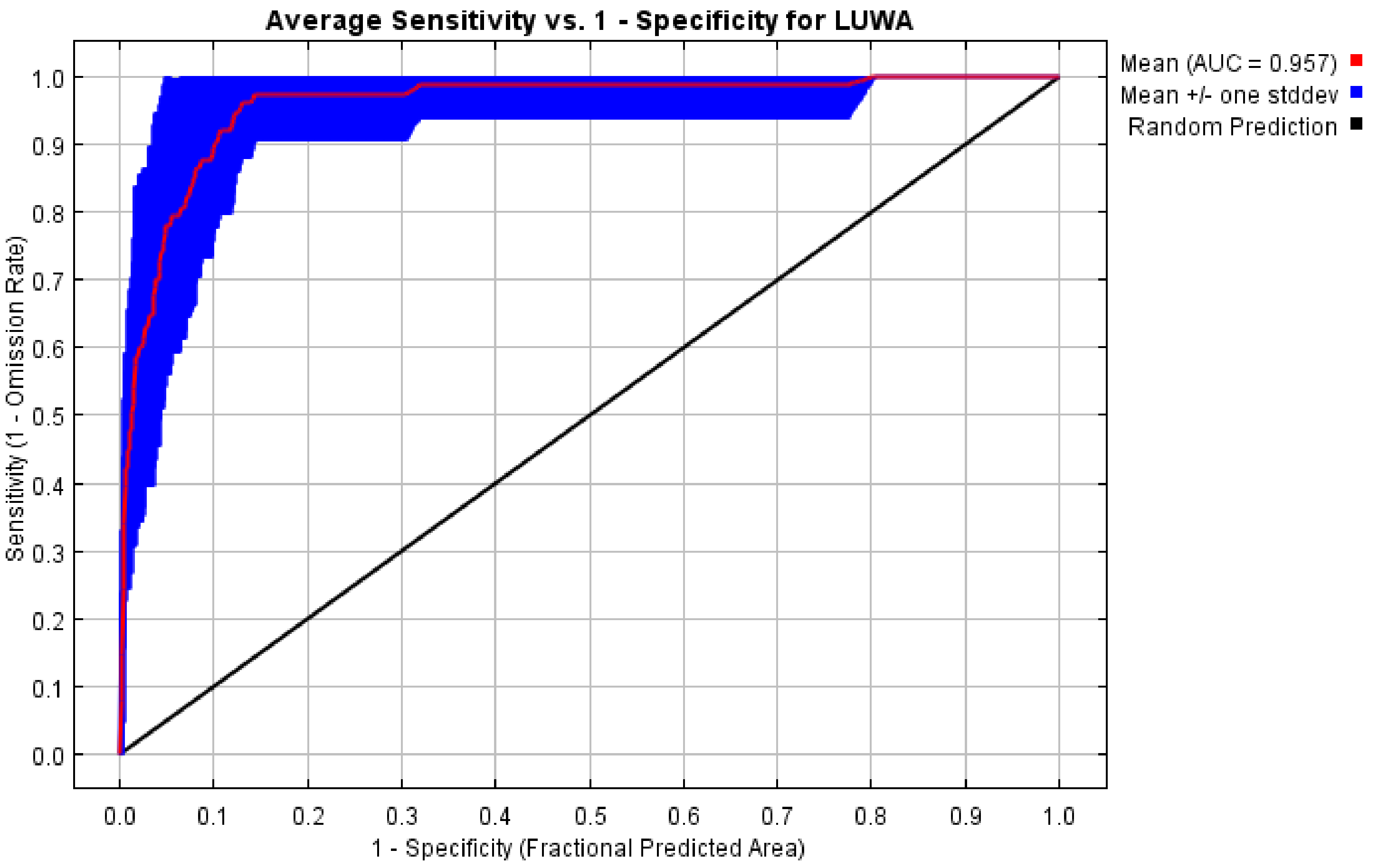

Pictures of the model

The following two pictures show the point-wise mean and standard deviation of the 15 output grids. Other available summary grids are min, max and median.

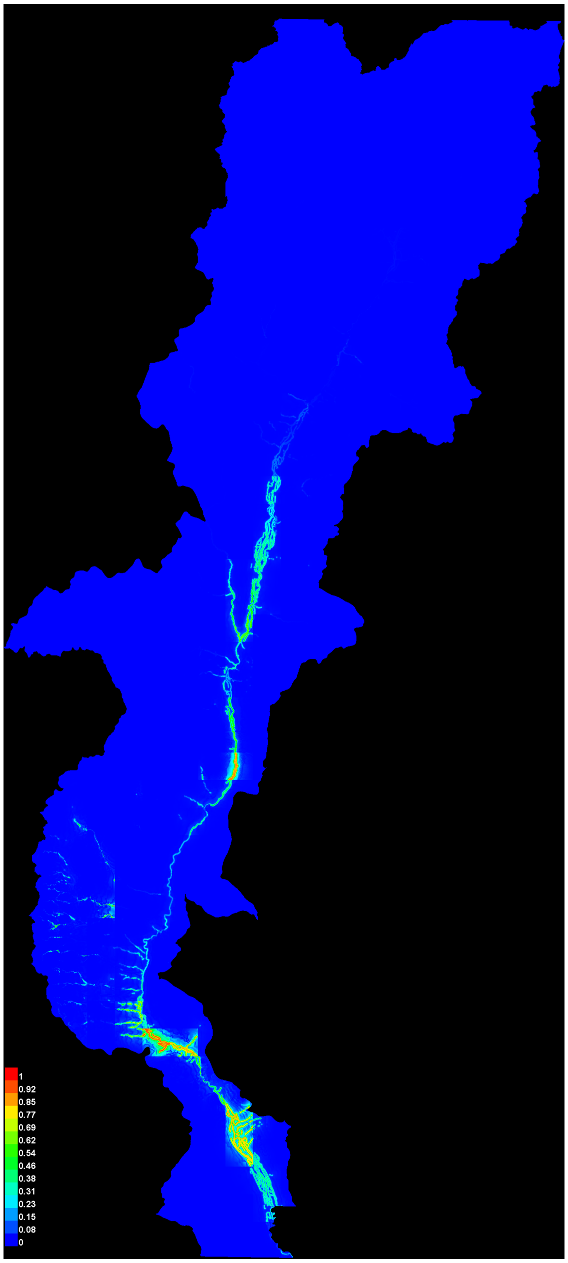

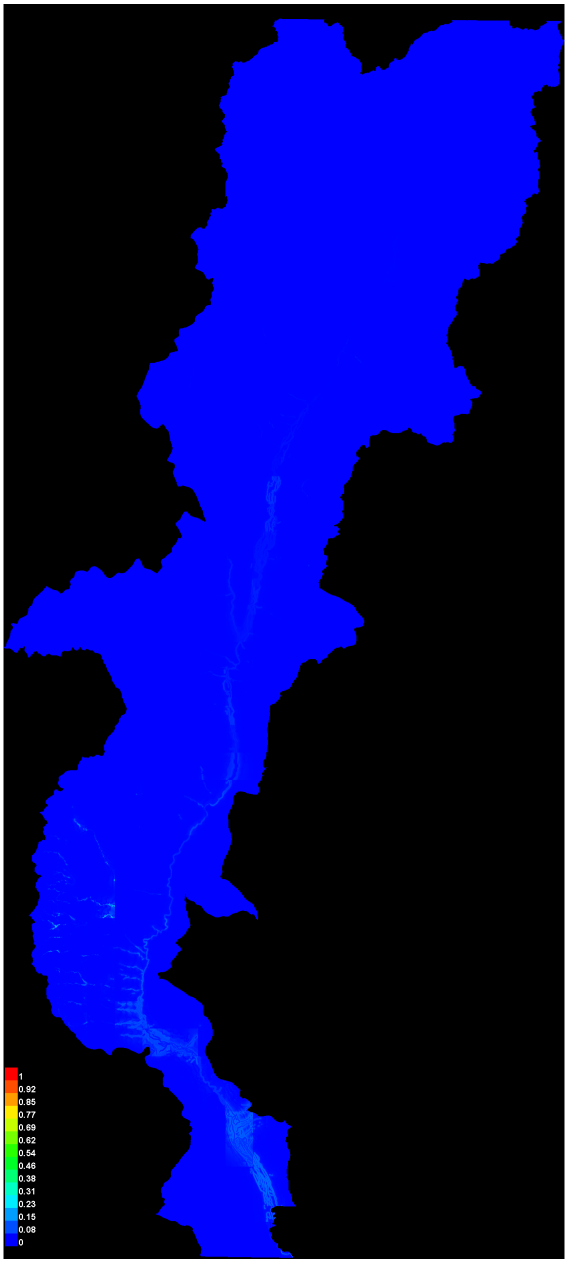

The following two pictures show the point-wise mean and standard deviation of the 15 models applied to the environmental layers in Envir_2030_ascii. Other available summary grids are min, max and median.

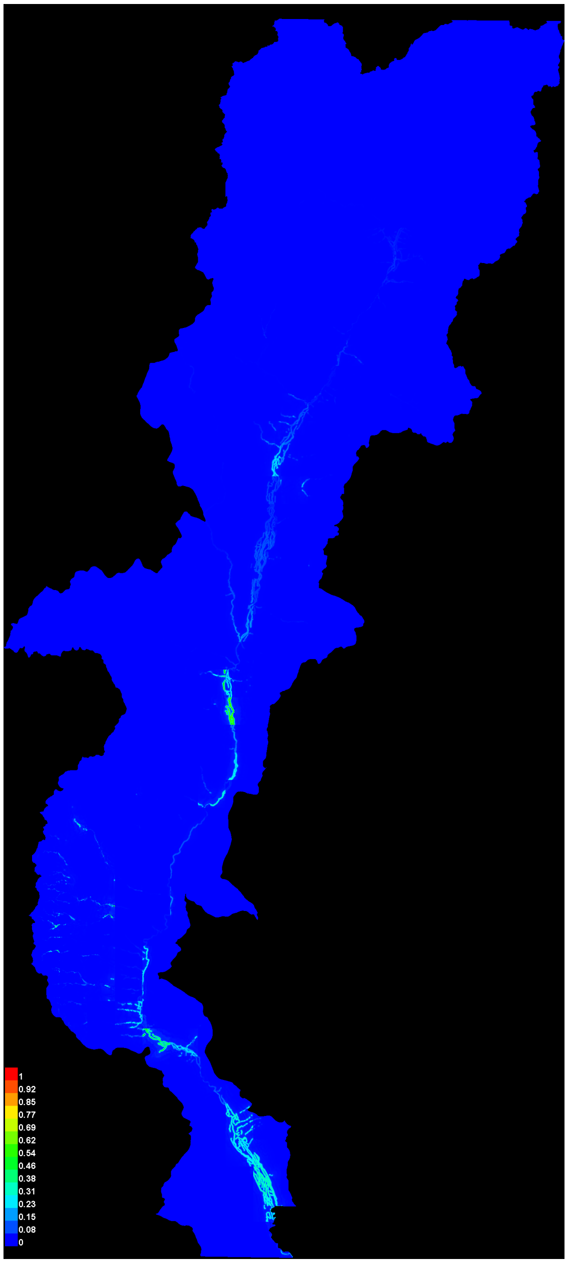

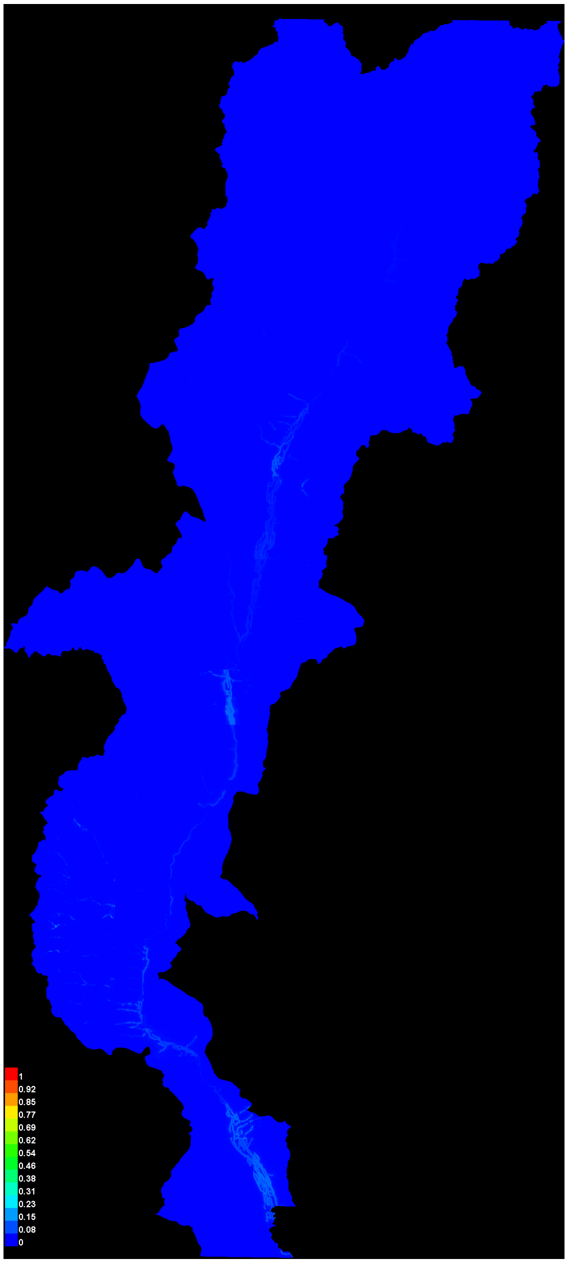

The following two pictures show the point-wise mean and standard deviation of the 15 models applied to the environmental layers in Envir_2060_ascii. Other available summary grids are min, max and median.

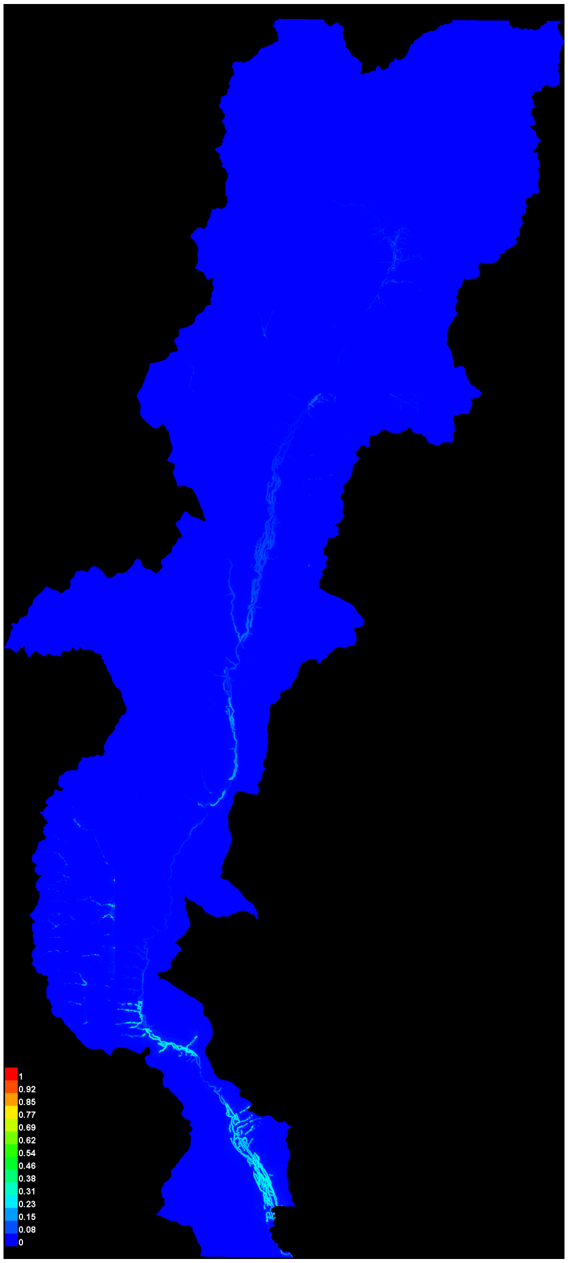

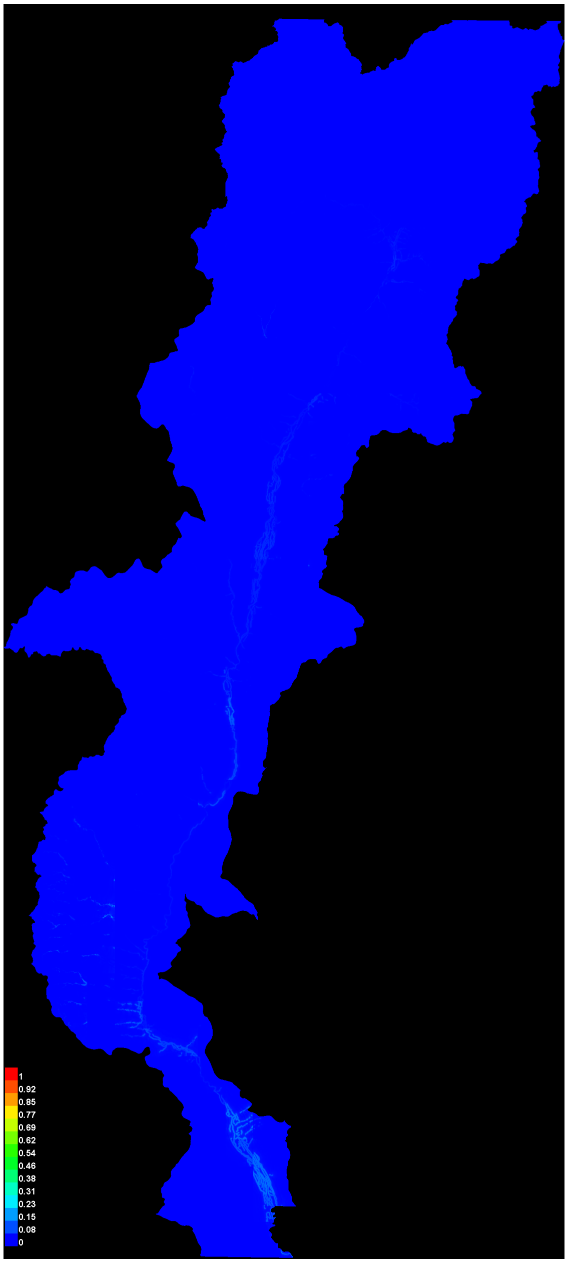

The following two pictures show the point-wise mean and standard deviation of the 15 models applied to the environmental layers in Envir_2090_ascii. Other available summary grids are min, max and median.

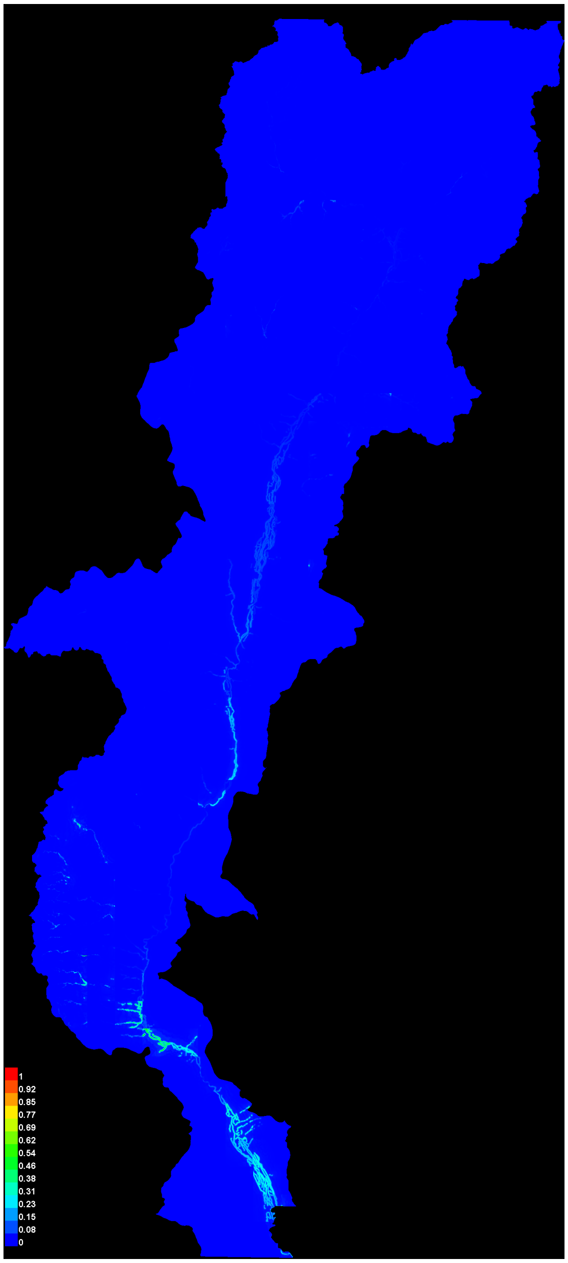

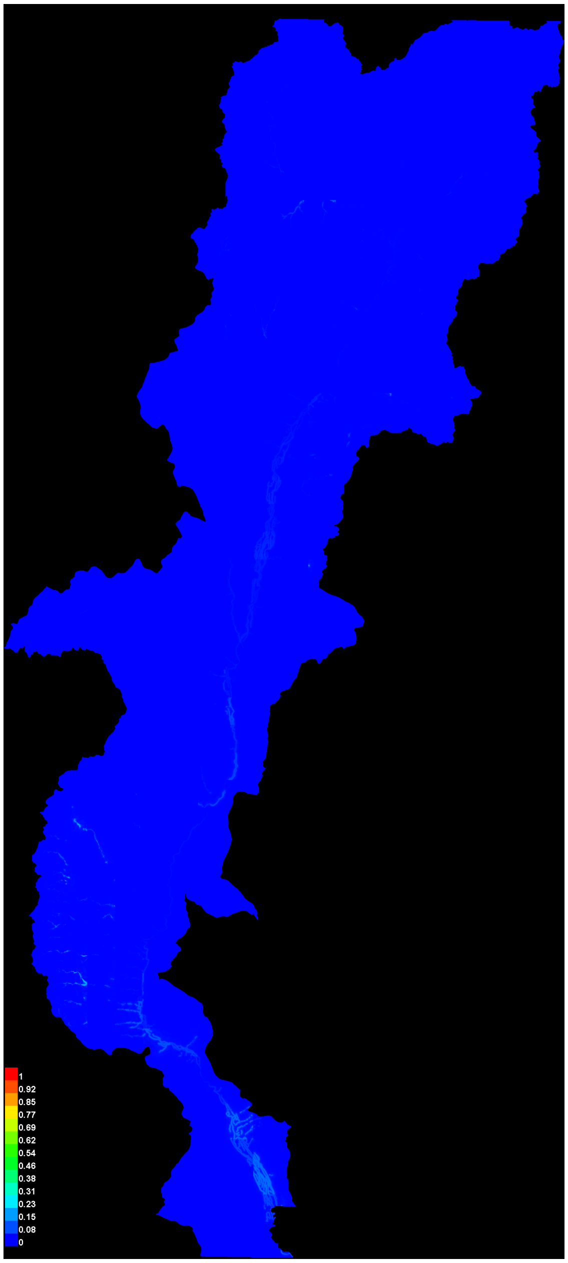

Response curves

These curves show how each environmental variable affects the Maxent prediction. The curves show how the logistic prediction changes as each environmental variable is varied, keeping all other environmental variables at their average sample value. Click on a response curve to see a larger version. Note that the curves can be hard to interpret if you have strongly correlated variables, as the model may depend on the correlations in ways that are not evident in the curves. In other words, the curves show the marginal effect of changing exactly one variable, whereas the model may take advantage of sets of variables changing together. The curves show the mean response of the 15 replicate Maxent runs (red) and and the mean +/- one standard deviation (blue, two shades for categorical variables).

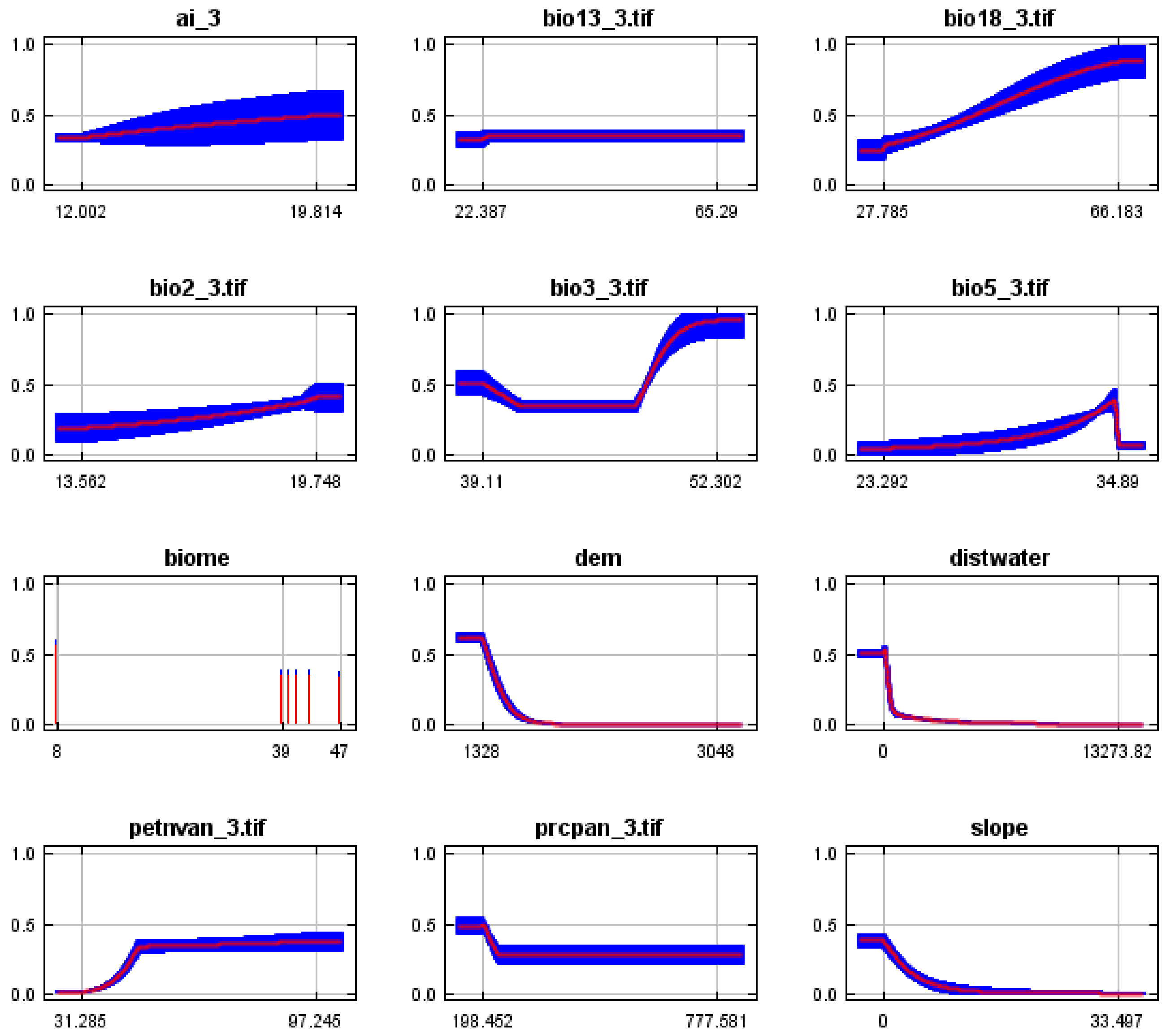

In contrast to the above marginal response curves, each of the following curves represents a different model, namely, a Maxent model created using only the corresponding variable. These plots reflect the dependence of predicted suitability both on the selected variable and on dependencies induced by correlations between the selected variable and other variables. They may be easier to interpret if there are strong correlations between variables.

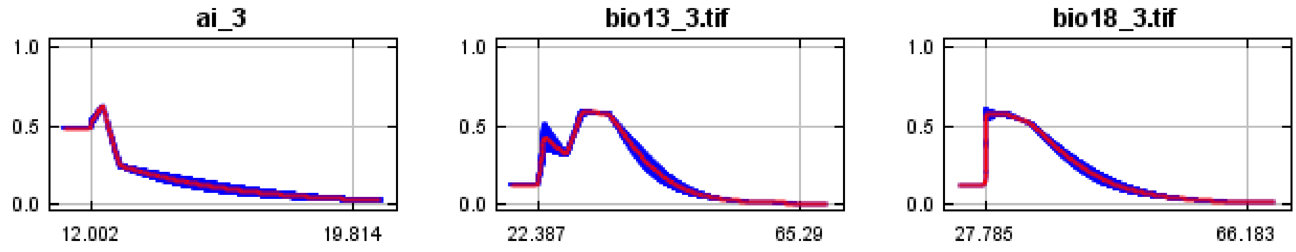

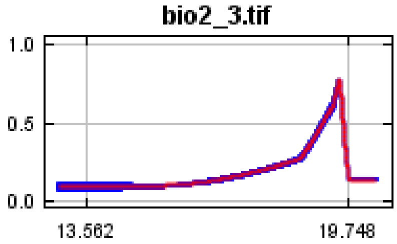

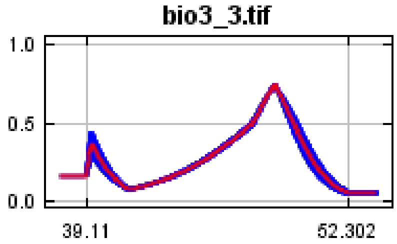

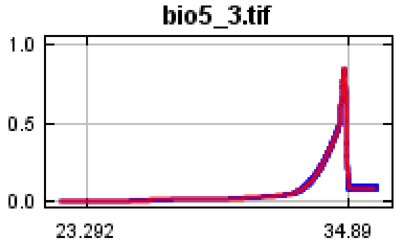

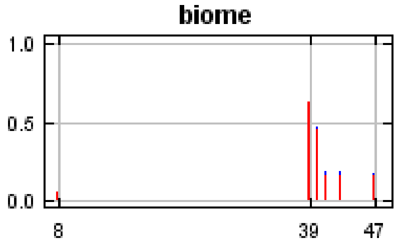

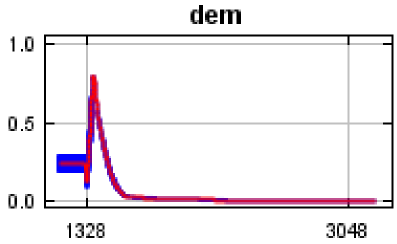

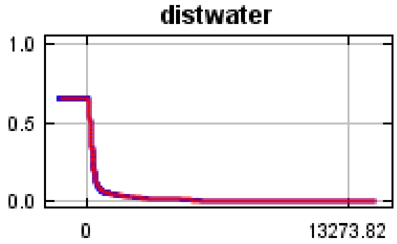

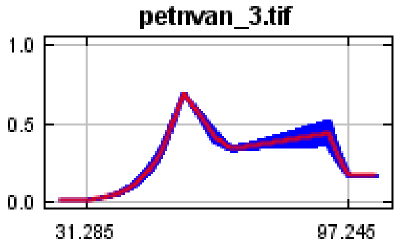

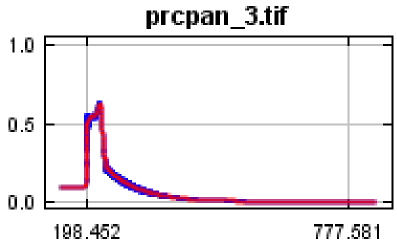

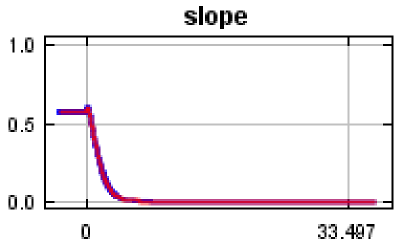

Analysis of variable contributions

The following table gives estimates of relative contributions of the environmental variables to the Maxent model. To determine the first estimate, in each iteration of the training algorithm, the increase in regularized gain is added to the contribution of the corresponding variable, or subtracted from it if the change to the absolute value of lambda is negative. For the second estimate, for each environmental variable in turn, the values of that variable on training presence and background data are randomly permuted. The model is reevaluated on the permuted data, and the resulting drop in training AUC is shown in the table, normalized to percentages. As with the variable jackknife, variable contributions should be interpreted with caution when the predictor variables are correlated. Values shown are averages over replicate runs.

Variable	Percent contribution	Permutation importance
distwater	55.6	24.1
dem	14.7	51.7
bio2_3.tif	14.5	0.8
bio5_3.tif	5.5	6.3
petnvan_3.tif	4.2	4.3
biome	2.9	0.6
slope	1.6	4.8
bio3_3.tif	0.6	4.2

prcpan_3.tif	0.2	0.3
bio18_3.tif	0.1	2.5
ai_3	0.1	0.4
bio13_3.tif	0	0

The following picture shows the results of the jackknife test of variable importance. The environmental variable with highest gain when used in isolation is distwater, which therefore appears to have the most useful information by itself. The environmental variable that decreases the gain the most when it is omitted is distwater, which therefore appears to have the most information that isn't present in the other variables. Values shown are averages over replicate runs.

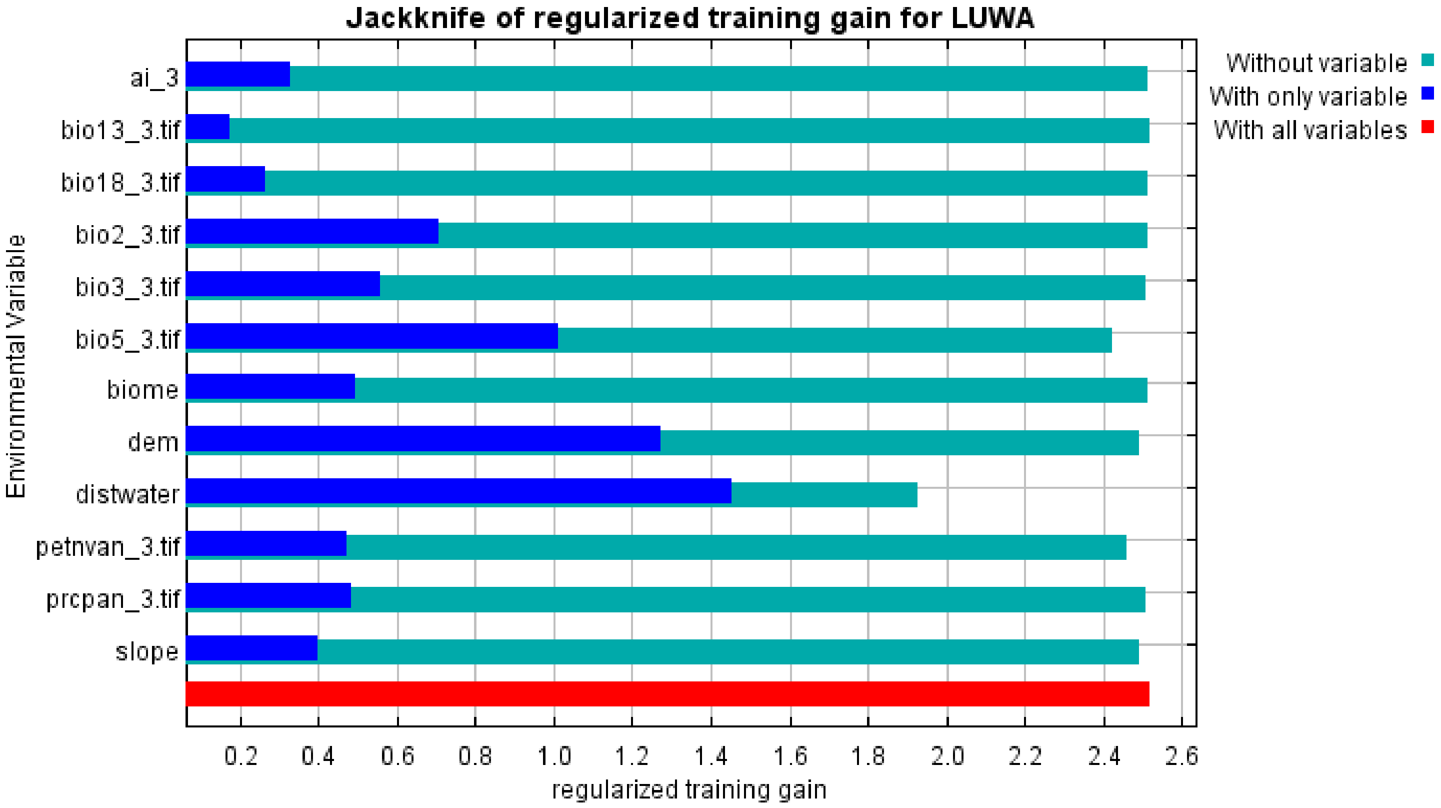

The next picture shows the same jackknife test, using test gain instead of training gain. Note that conclusions about which variables are most important can change, now that we're looking at test data.

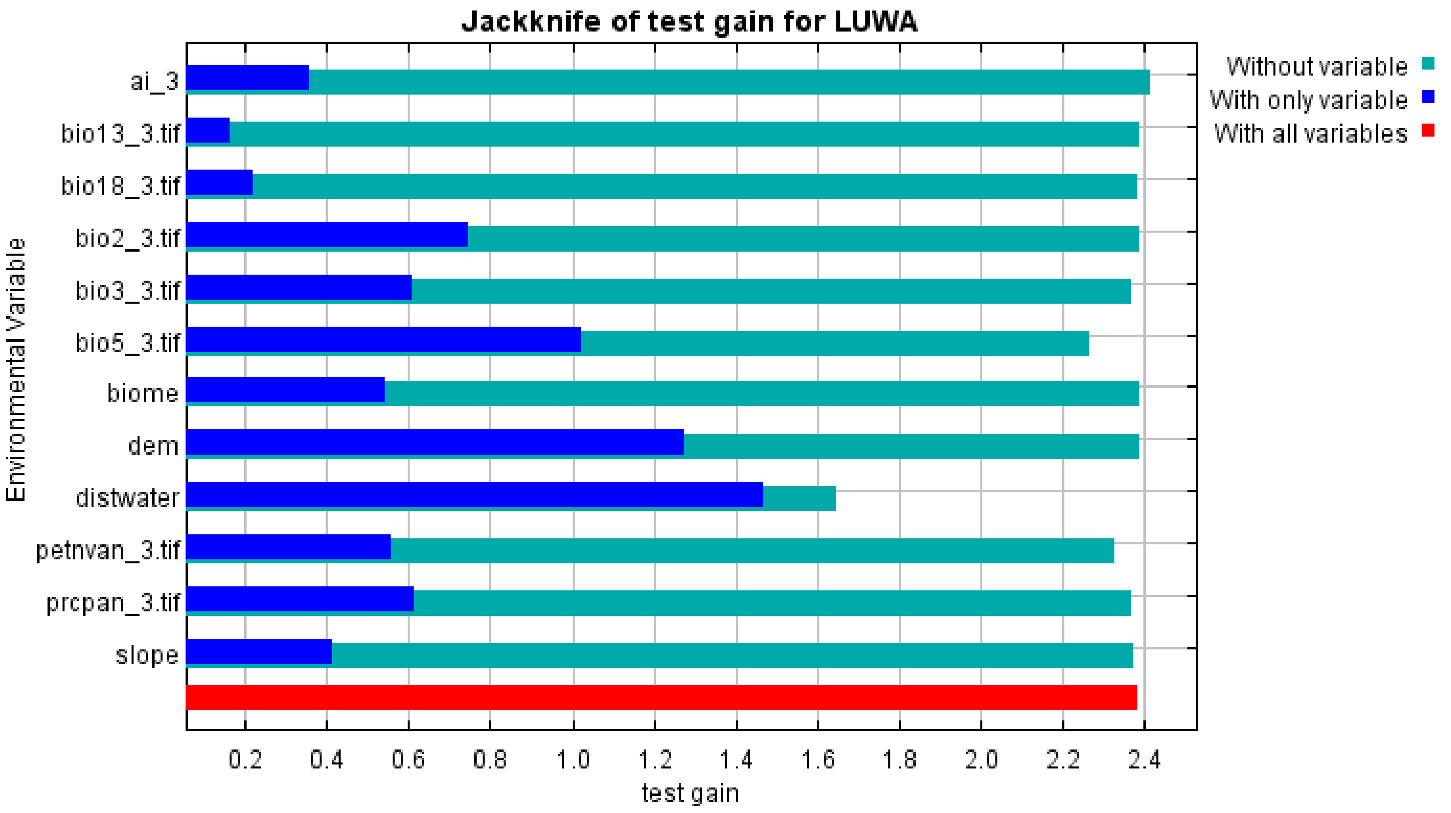

Lastly, we have the same jackknife test, using AUC on test data.

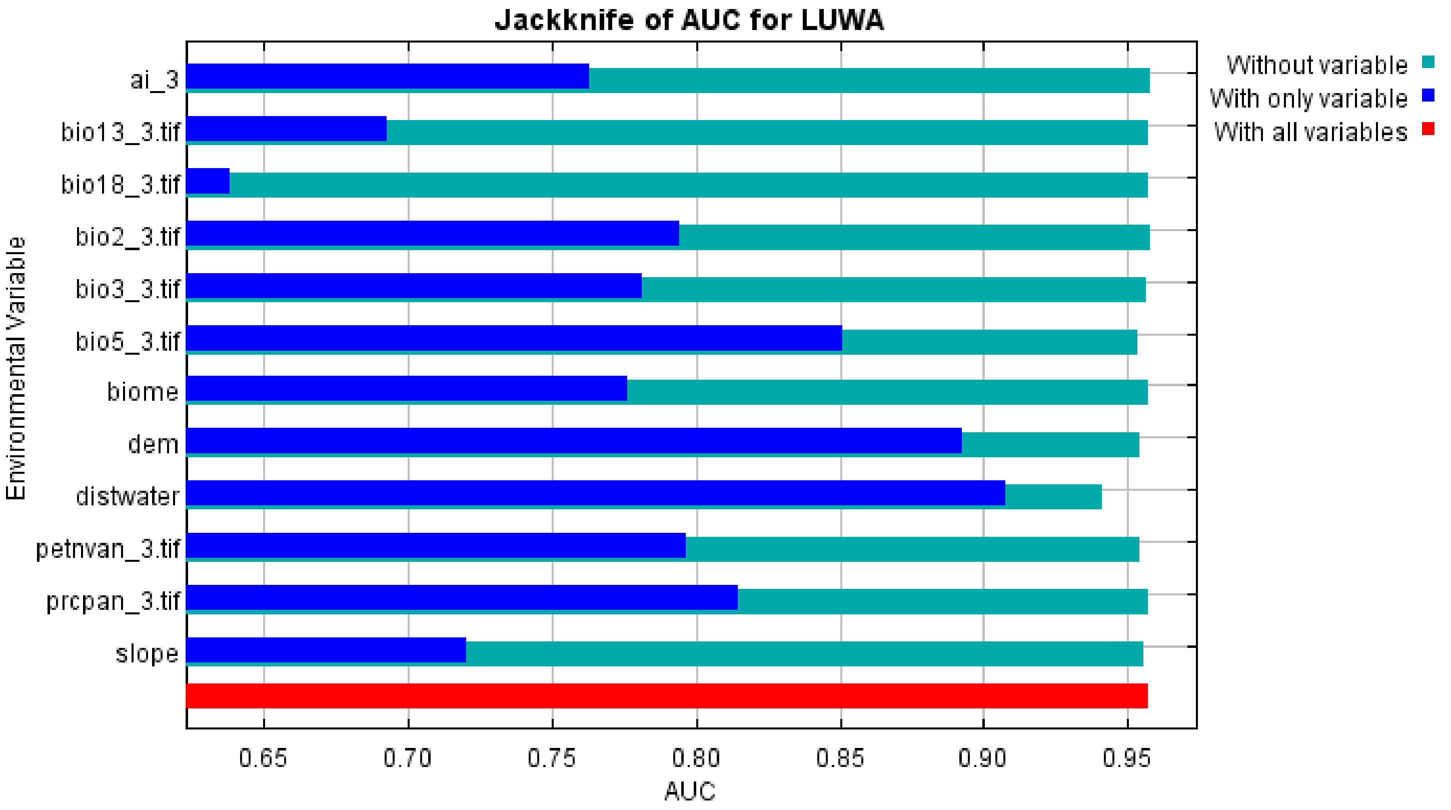

Command line to repeat this species model: java density.MaxEnt nowarnings noprefixes -E "" -E LUWA responsecurves jackknife outputdirectory=D:\MaxEnt4\BirdsHad

"projectionlayers=D:\Ascii_Files\Envir_2030_ascii, D:\Ascii_Files\Envir_2060_ascii, D:\Ascii_Files\Envir_2090_ascii" samplesfile=D:\Data\RioGrande\Birds\allbirds.csv environmentallayers=D:\Ascii_Files\Envir_Curr_ascii randomseed noaskoverwrite biasfile=D:\Data\RioGrande\Birds\all_bias.asc replicates=15 nooutputgrids maximumiterations=5000 biastype=3 "applythresholdrule=equal training sensitivity and specificity" -N ai_1 -N ai_2 -N bio10_1 -N bio10_1.tif -N bio10_2 -N bio10_2.tif -N bio10_3 -N bio10_3.tif -N bio11_1 -N bio11_1.tif -N bio11_2 -N bio11_2.tif -N bio11_3 -N bio11_3.tif -N bio13_1.tif -N bio13_2.tif -N bio14_1.tif -N bio14_2.tif -N bio14_3.tif -N bio16_1.tif -N bio16_2.tif -N bio16_3.tif -N bio17_.tif -N bio17_2.tif -N bio17_3.tif -N bio17_all.tif -N bio18_1.tif -N bio18_2.tif -N bio19_1.tif -N bio19_2.tif -N bio19_3.tif -N bio2_1.tif -N bio2_2.tif -N bio3_1.tif -N bio3_2.tif -N bio5_1.tif -N bio5_2.tif -N bio6_1.tif -N bio6_2.tif -N bio6_3.tif -N bio7_1.tif -N bio7_2.tif -N bio7_3.tif -N etan_1.tif -N etan_2.tif -N etan_3.tif -N etdjf_1.tif -N etdjf_2.tif -N etdjf_3.tif -N etjas_1.tif -N etjas_2.tif -N etjas_3.tif -N etjfm_1.tif -N etjfm_2.tif -N etjfm_3.tif -N etjja_1.tif -N etjja_2.tif -N etjja_3.tif -N etjja_all.tif -N petnvan_1.tif -N petnvan_2.tif -N petnvdjf_1.tif -N petnvdjf_2.tif -N petnvdjf_3.tif -N petnvjas_1.tif -N petnvjas_2.tif -N petnvjas_3.tif -N petnvjfm_1.tif -N petnvjfm_2.tif -N petnvjfm_3.tif -N petnvjja_1.tif -N petnvjja_2.tif -N petnvjja_3.tif -N petwan_1.tif -N petwan_2.tif -N petwan_3.tif -N petwdjf_1.tif -N petwdjf_2.tif -N petwdjf_3.tif -N petwjas_1.tif -N petwjas_2.tif -N petwjas_3.tif -N petwjas_all.tif -N petwjfm_1.tif -N petwjfm_2.tif -N petwjfm_3.tif -N petwjja_1.tif -N petwjja_2.tif -N petwjja_3.tif -N prcpan_1.tif -N prcpan_2.tif -N prcpan_all.tif -N runoffan_1.tif -N runoffan_2.tif -N runoffan_3.tif -N runoffdjf_1.tif -N runoffdjf_2.tif -N runoffdjf_3.tif -N runoffjas_1.tif -N runoffjas_2.tif -N runoffjas_3.tif -N runoffjas_all.tif -N runoffjfm_1.tif -N runoffjfm_2.tif -N runoffjfm_3.tif -N runoffjja_1.tif -N runoffjja_2.tif -N runoffjja_3.tif -N smcan_1.tif -N smcan_2.tif -N smcan_3.tif -N smcdjf_1.tif -N smcdjf_2.tif -N smcdjf_3.tif -N smcjas_1.tif -N smcjas_2.tif -N smcjas_3.tif -N smcjfm_1.tif -N smcjfm_2.tif -N smcjfm_3.tif -N smcjja_1.tif -N smcjja_2.tif -N smcjja_3.tif -N swean_1.tif -N swean_2.tif -N swean_3.tif -N swedjf_1.tif -N swedjf_2.tif -N swedjf_3.tif -N swejfm_1.tif -N swejfm_2.tif -N swejfm_3.tif -N tave_1.tif -N tave_2.tif -N tave_3.tif -N tmax_1.tif -N tmax_2.tif -N tmax_3.tif -N tmin_1.tif -N tmin_2.tif -N tmin_3.tif -t biome

Replicated maxent model for WIFL

This page summarizes the results of 15-fold cross-validation for WIFL, created Tue Aug 05 14:14:22 MDT 2014 using Maxent version 3.3.3k. The individual models are here: [0] [1] [2] [3] [4] [5] [6] [7] [8] [9] [10] [11] [12] [13] [14]

Analysis of omission/commission

The following picture shows the test omission rate and predicted area as a function of the cumulative threshold, averaged over the replicate runs. The omission rate should be close to the predicted omission, because of the definition of the cumulative threshold.

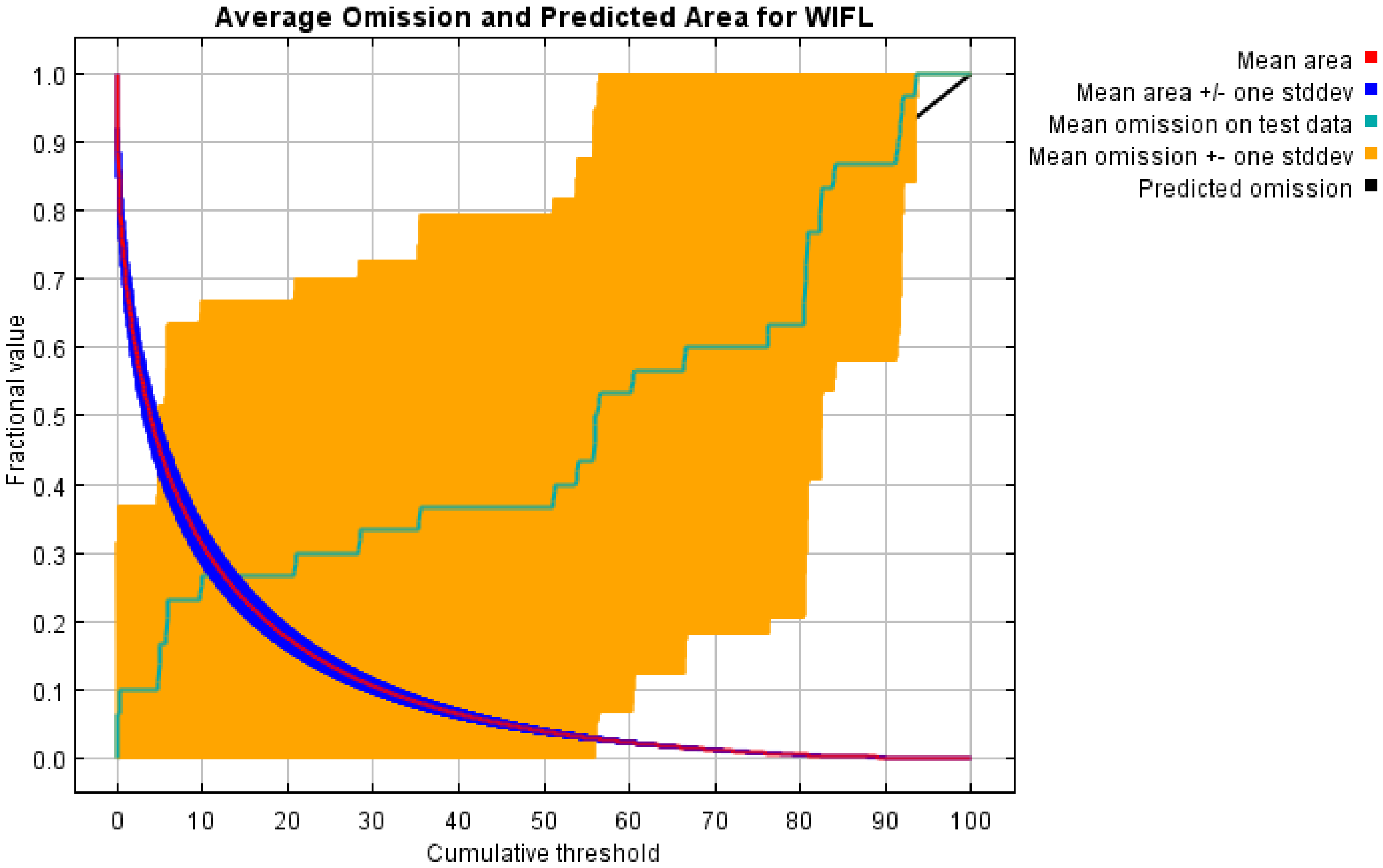

The next picture is the receiver operating characteristic (ROC) curve for the same data, again averaged over the replicate runs. Note that the specificity is defined using predicted area, rather than true commission (see the paper by Phillips, Anderson and Schapire cited on the help page for discussion of what this means). The average test AUC for the replicate runs is 0.833, and the standard deviation is 0.235.

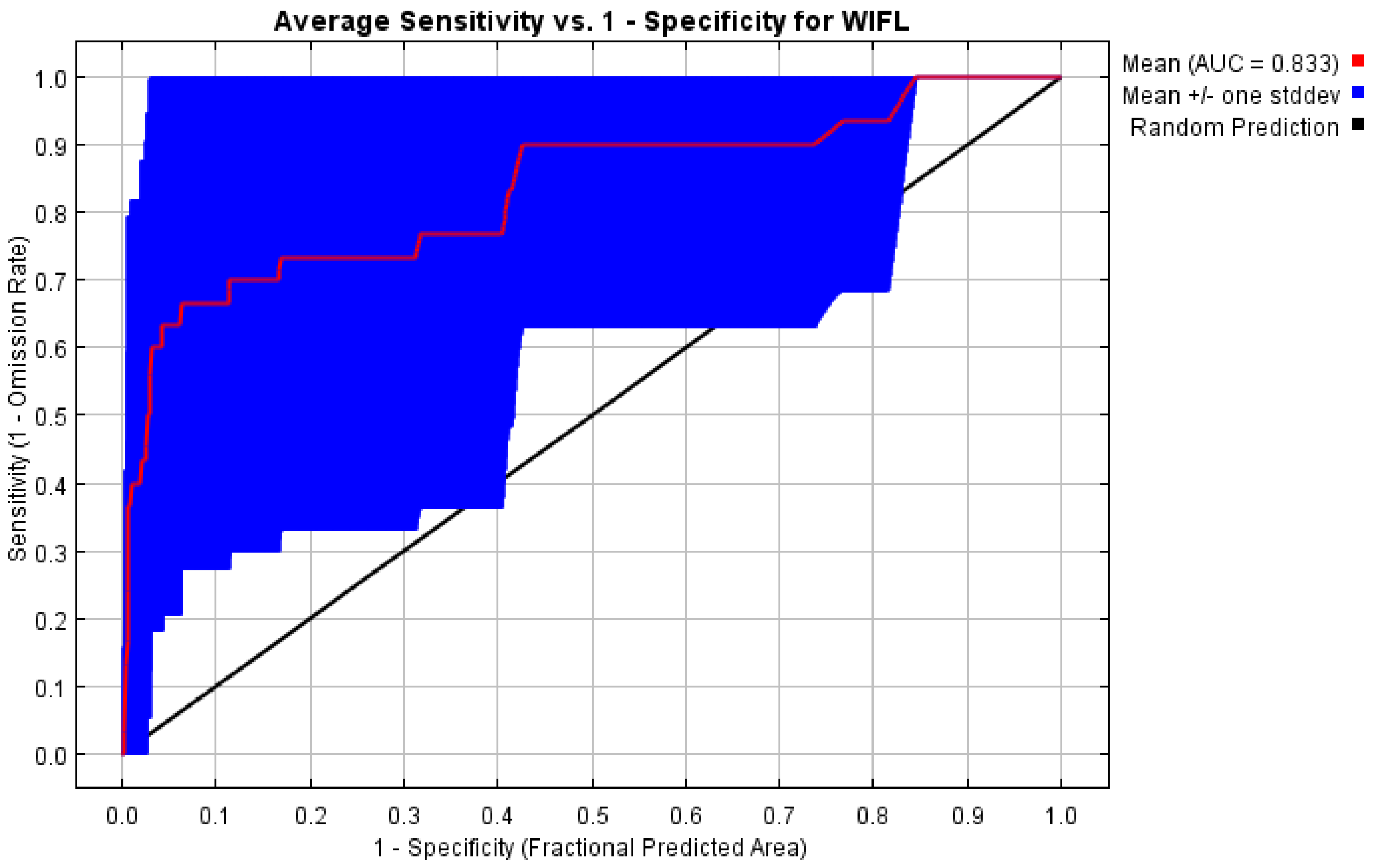

Pictures of the model

The following two pictures show the point-wise mean and standard deviation of the 15 output grids. Other available summary grids are min, max and median.

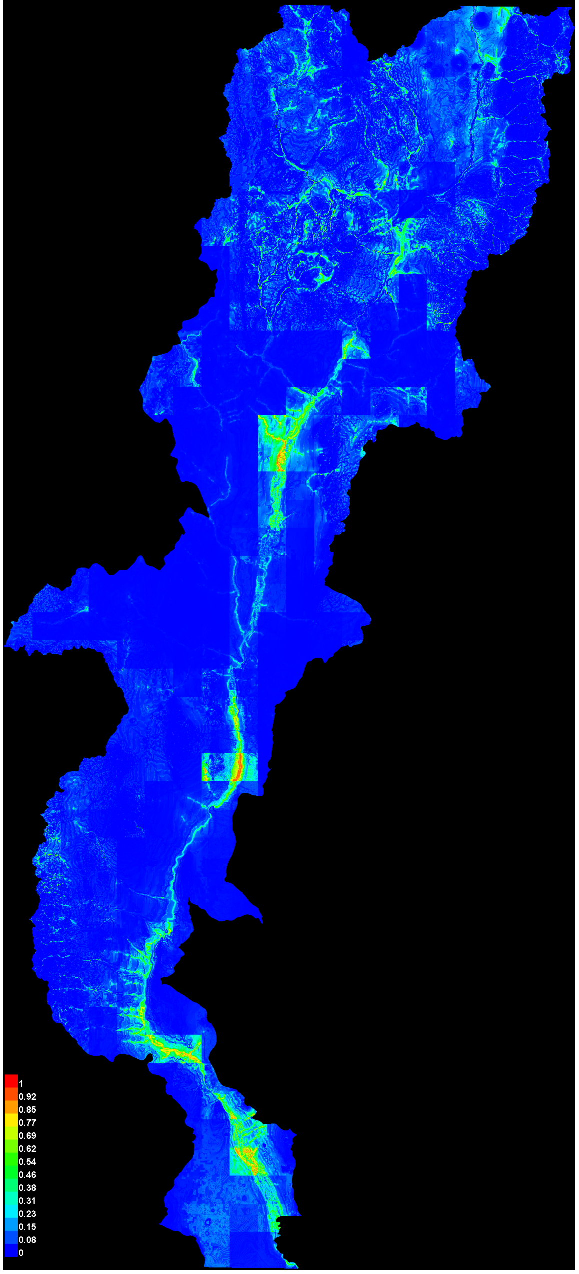

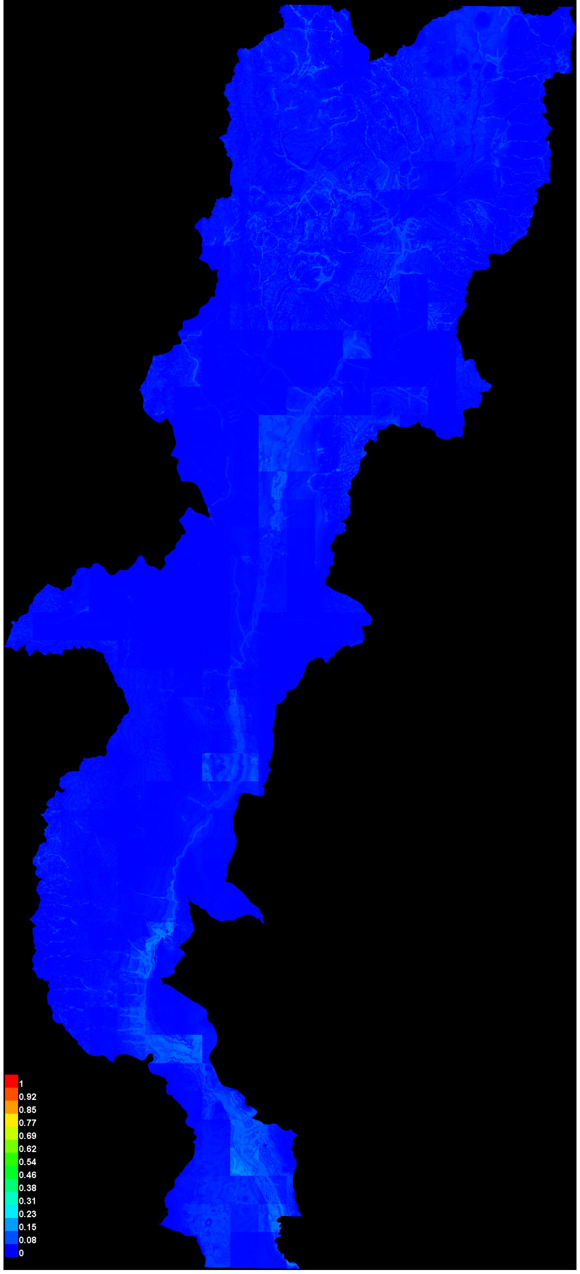

The following two pictures show the point-wise mean and standard deviation of the 15 models applied to the environmental layers in Envir_2030_ascii. Other available summary grids are min, max and median.

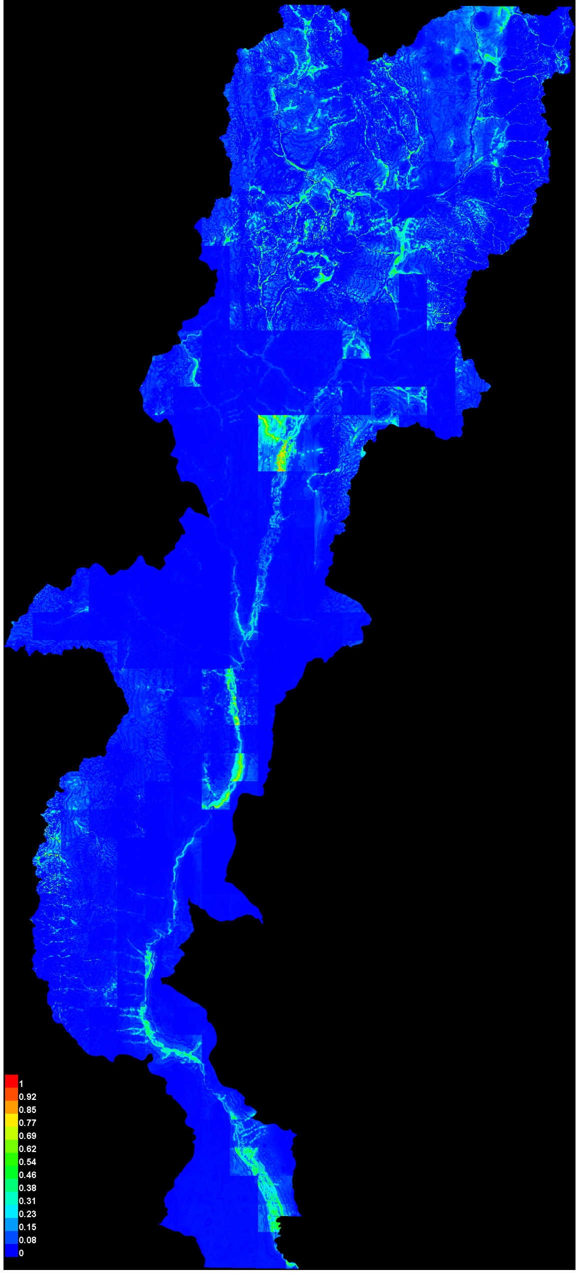

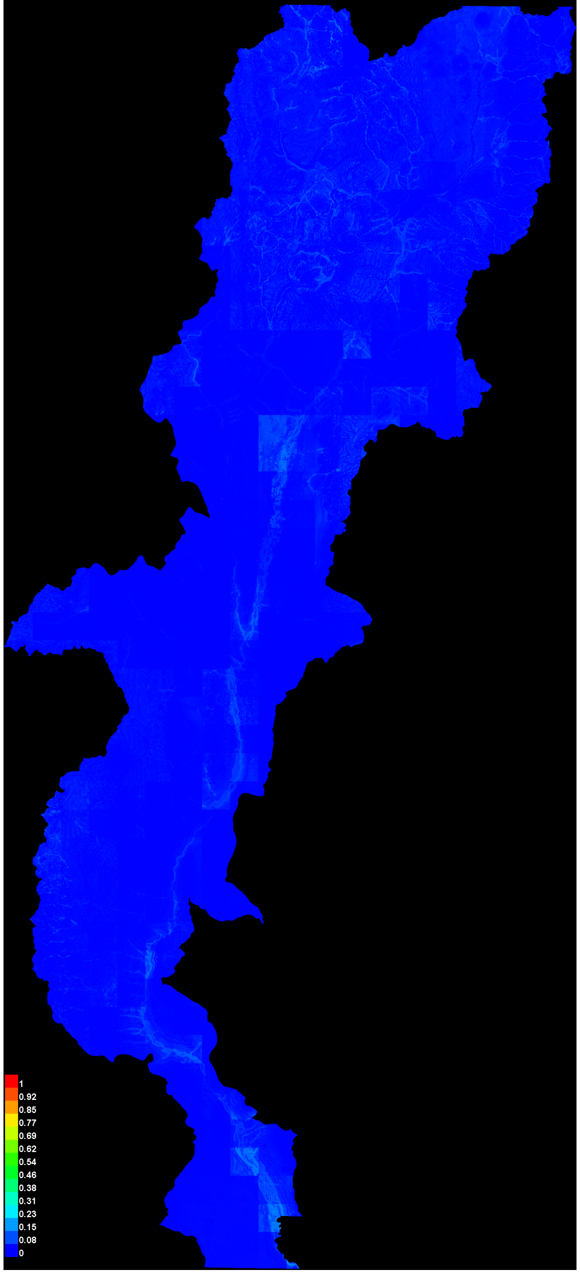

The following two pictures show the point-wise mean and standard deviation of the 15 models applied to the environmental layers in Envir_2060_ascii. Other available summary grids are min, max and median.

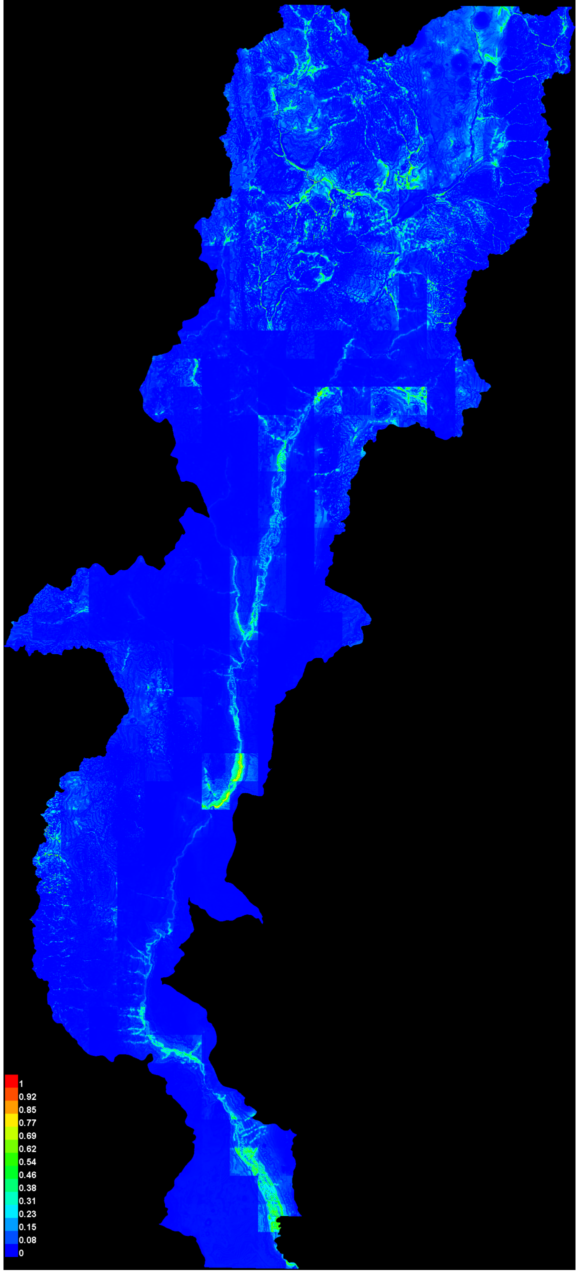

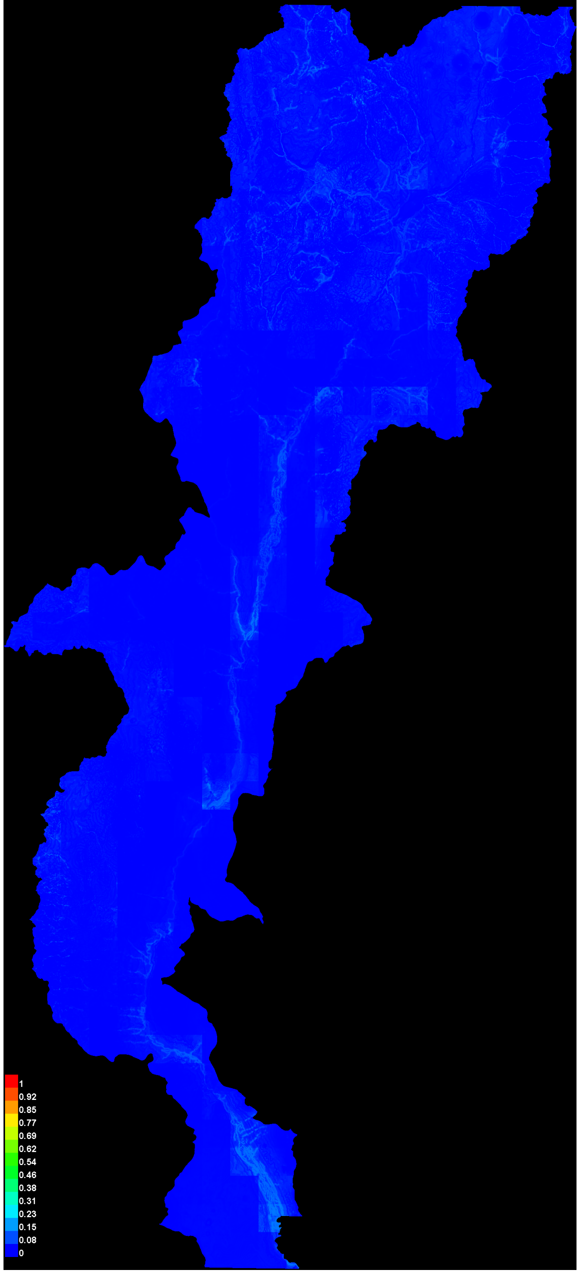

The following two pictures show the point-wise mean and standard deviation of the 15 models applied to the environmental layers in Envir_2090_ascii. Other available summary grids are min, max and median.

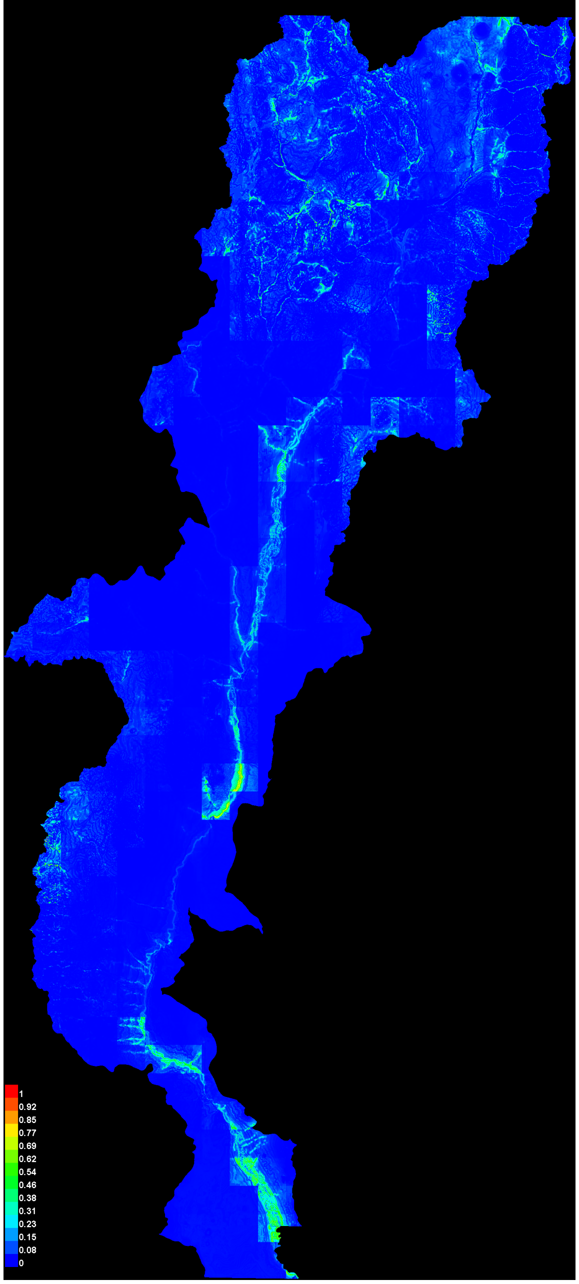

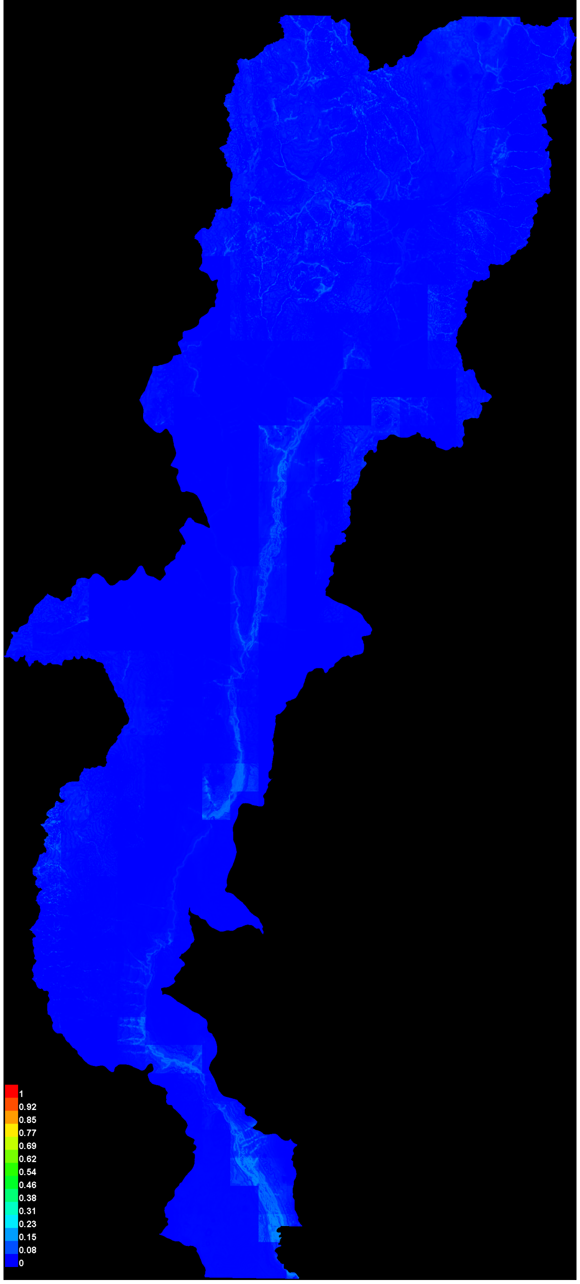

Response curves

These curves show how each environmental variable affects the Maxent prediction. The curves show how the logistic prediction changes as each environmental variable is varied, keeping all other environmental variables at their average sample value. Click on a response curve to see a larger version. Note that the curves can be hard to interpret if you have strongly correlated variables, as the model may depend on the correlations in ways that are not evident in the curves. In other words, the curves show the marginal effect of changing exactly one variable, whereas the model may take advantage of sets of variables changing together. The curves show the mean response of the 15 replicate Maxent runs (red) and and the mean +/- one standard deviation (blue, two shades for categorical variables).

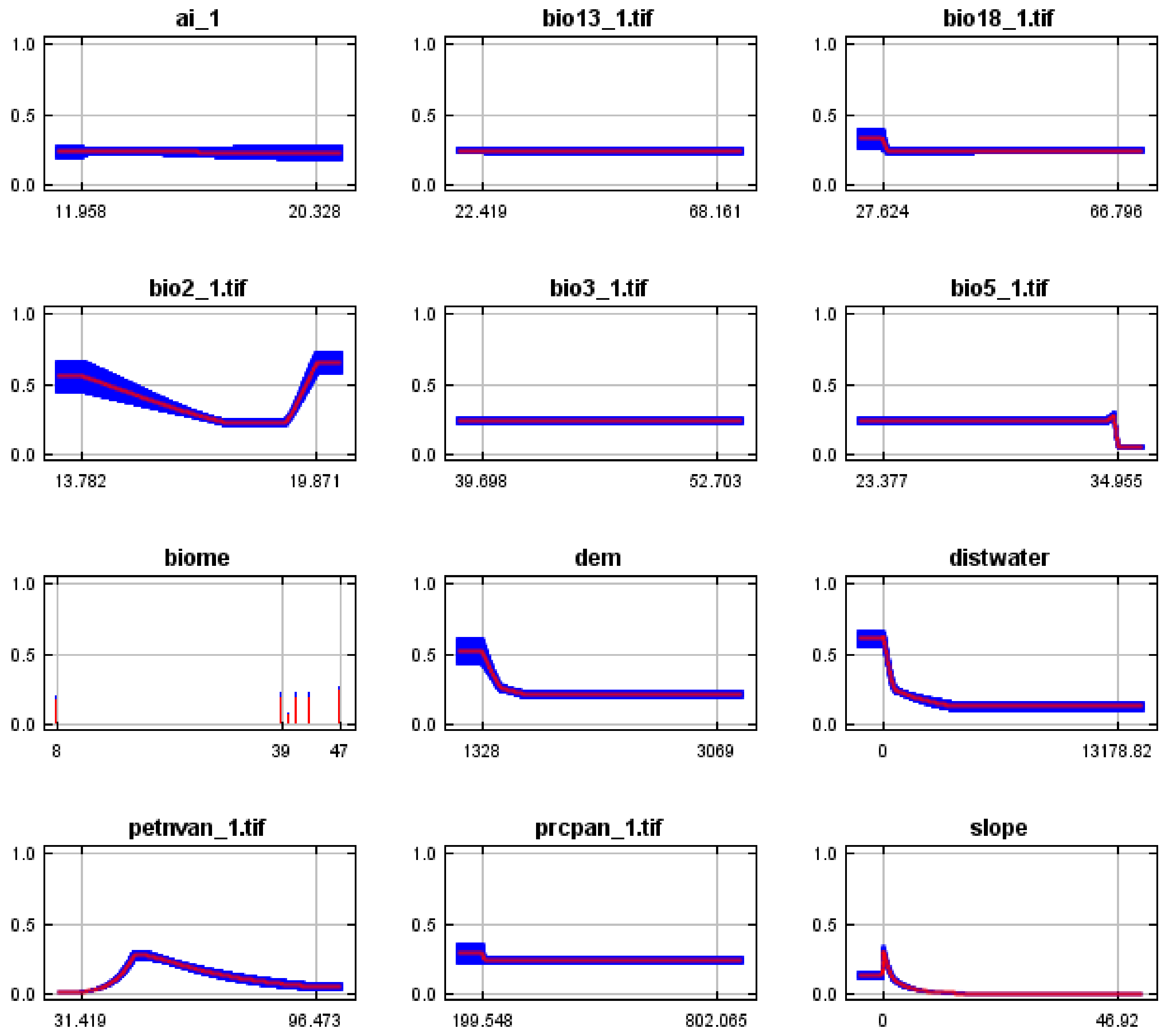

In contrast to the above marginal response curves, each of the following curves represents a different model, namely, a Maxent model created using only the corresponding variable. These plots reflect the dependence of predicted suitability both on the selected variable and on dependencies induced by correlations between the selected variable and other variables. They may be easier to interpret if there are strong correlations between variables.

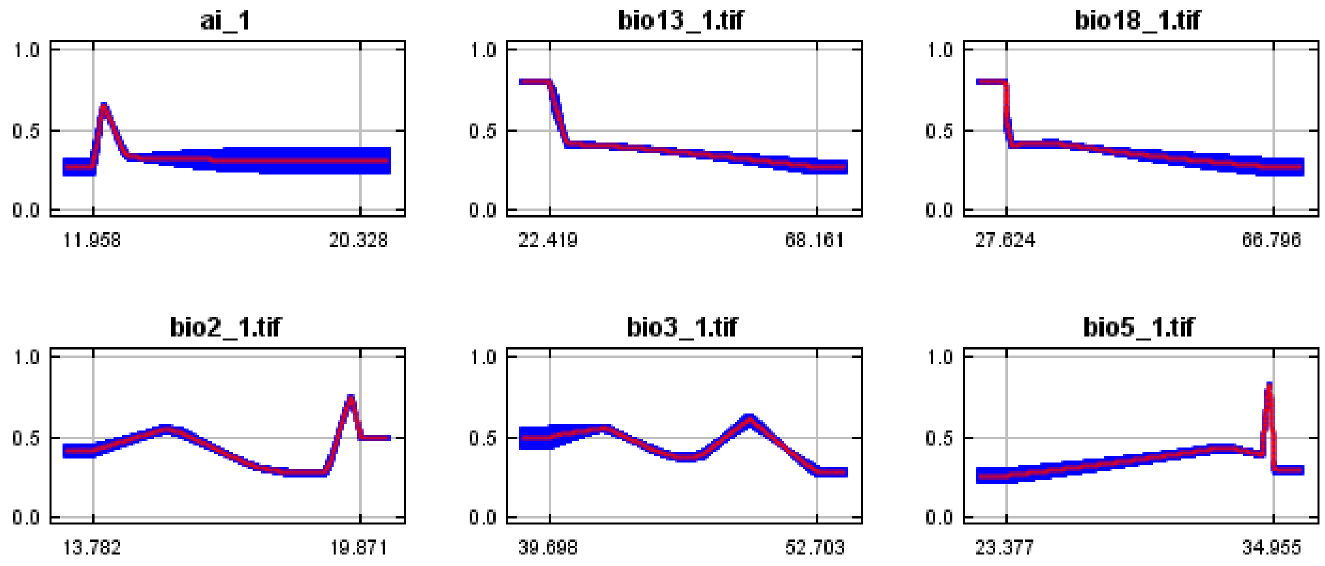

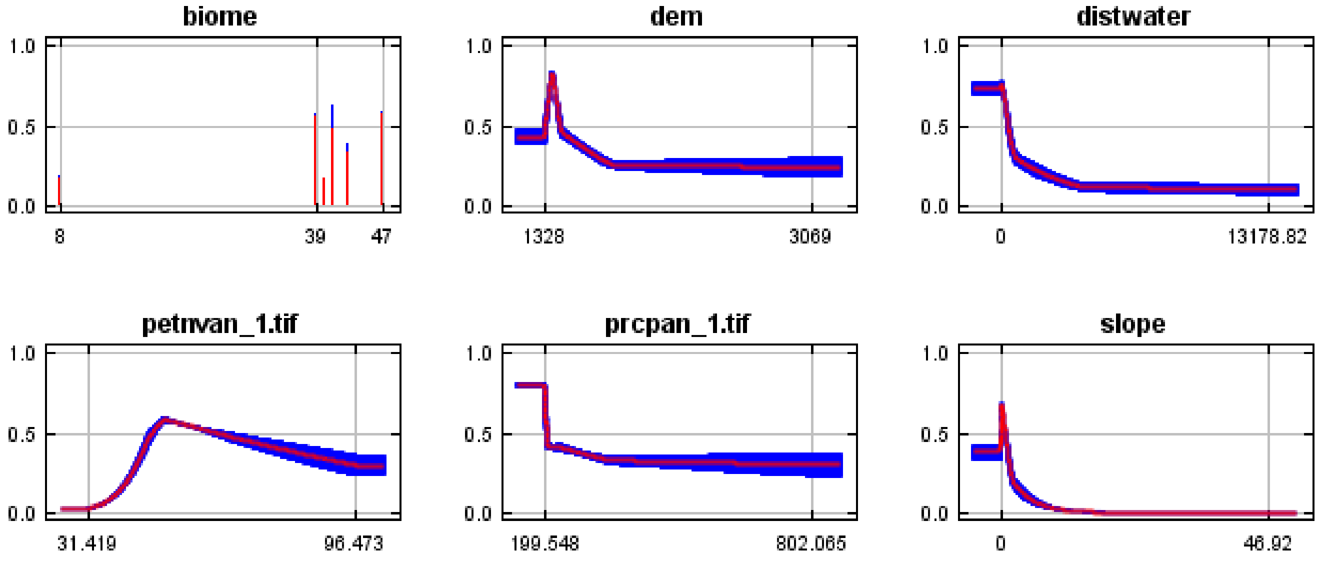

Analysis of variable contributions

The following table gives estimates of relative contributions of the environmental variables to the Maxent model. To determine the first estimate, in each iteration of the training algorithm, the increase in regularized gain is added to the contribution of the corresponding variable, or subtracted from it if the change to the absolute value of lambda is negative. For the second estimate, for each environmental variable in turn, the values of that variable on training presence and background data are randomly permuted. The model is reevaluated on the permuted data, and the resulting drop in training AUC is shown in the table, normalized to percentages. As with the variable jackknife, variable contributions should be interpreted with caution when the predictor variables are correlated. Values shown are averages over replicate runs.

Variable	Percent contribution	Permutation importance
distwater	41.6	19.9
slope	14.8	27.6
bio2_1.tif	10	9.2
biome	9.8	12.4
petnvan_1.tif	5.6	23.6
bio13_1.tif	4.6	0
bio18_1.tif	4.2	0.1
bio5_1.tif	4	1.9
prcpan_1.tif	3	0.1
dem	2.2	4.6
ai_1	0.2	0.7
bio3_1.tif	0	0

The following picture shows the results of the jackknife test of variable importance. The environmental variable with highest gain when used in isolation is distwater, which therefore appears to have the most useful information by itself. The environmental variable that decreases the gain the most when it is omitted is distwater, which therefore appears to have the most information that isn't present in the other variables. Values shown are averages over replicate runs.

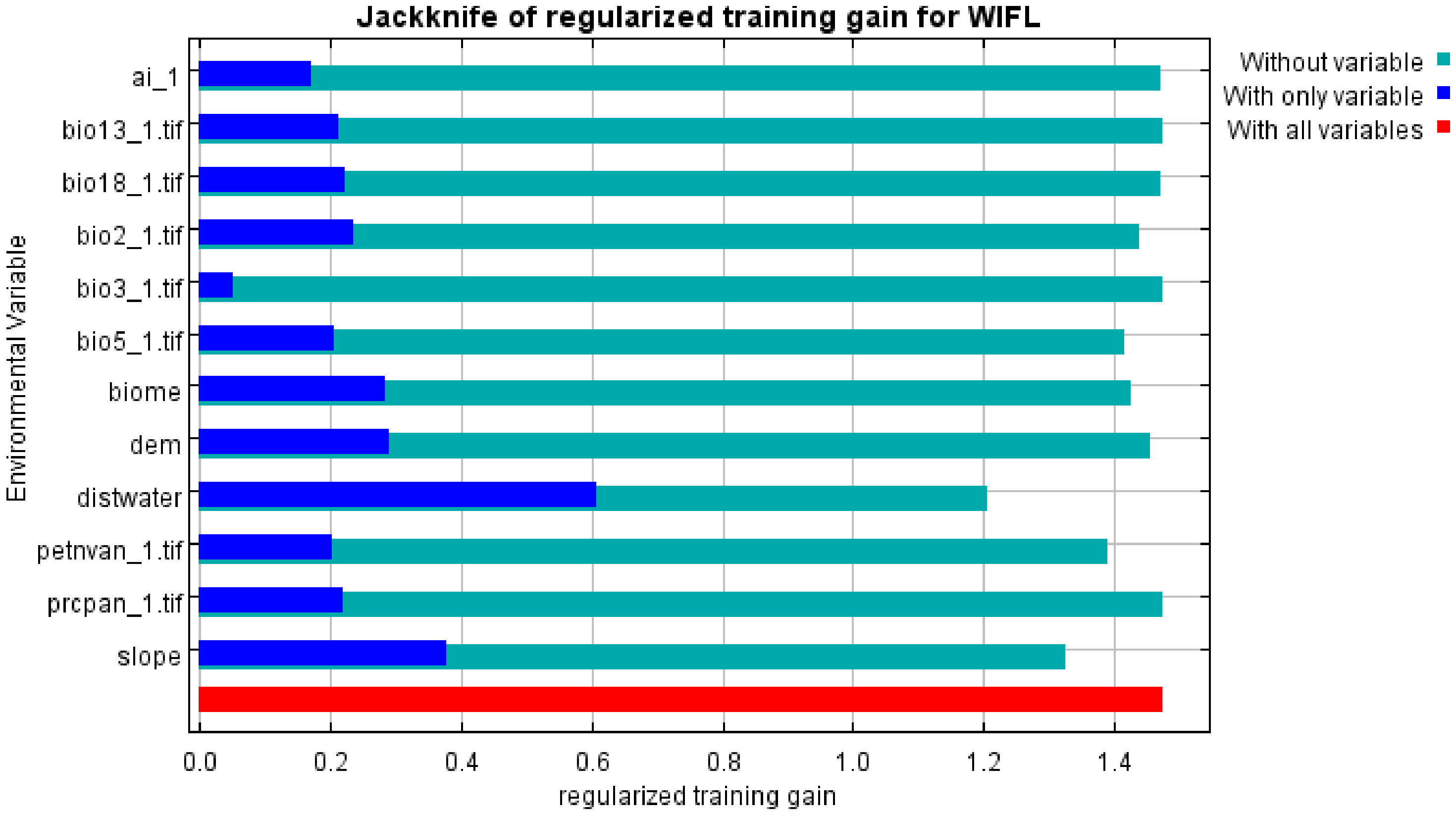

The next picture shows the same jackknife test, using test gain instead of training gain. Note that conclusions about which variables are most important can change, now that we're looking at test data.

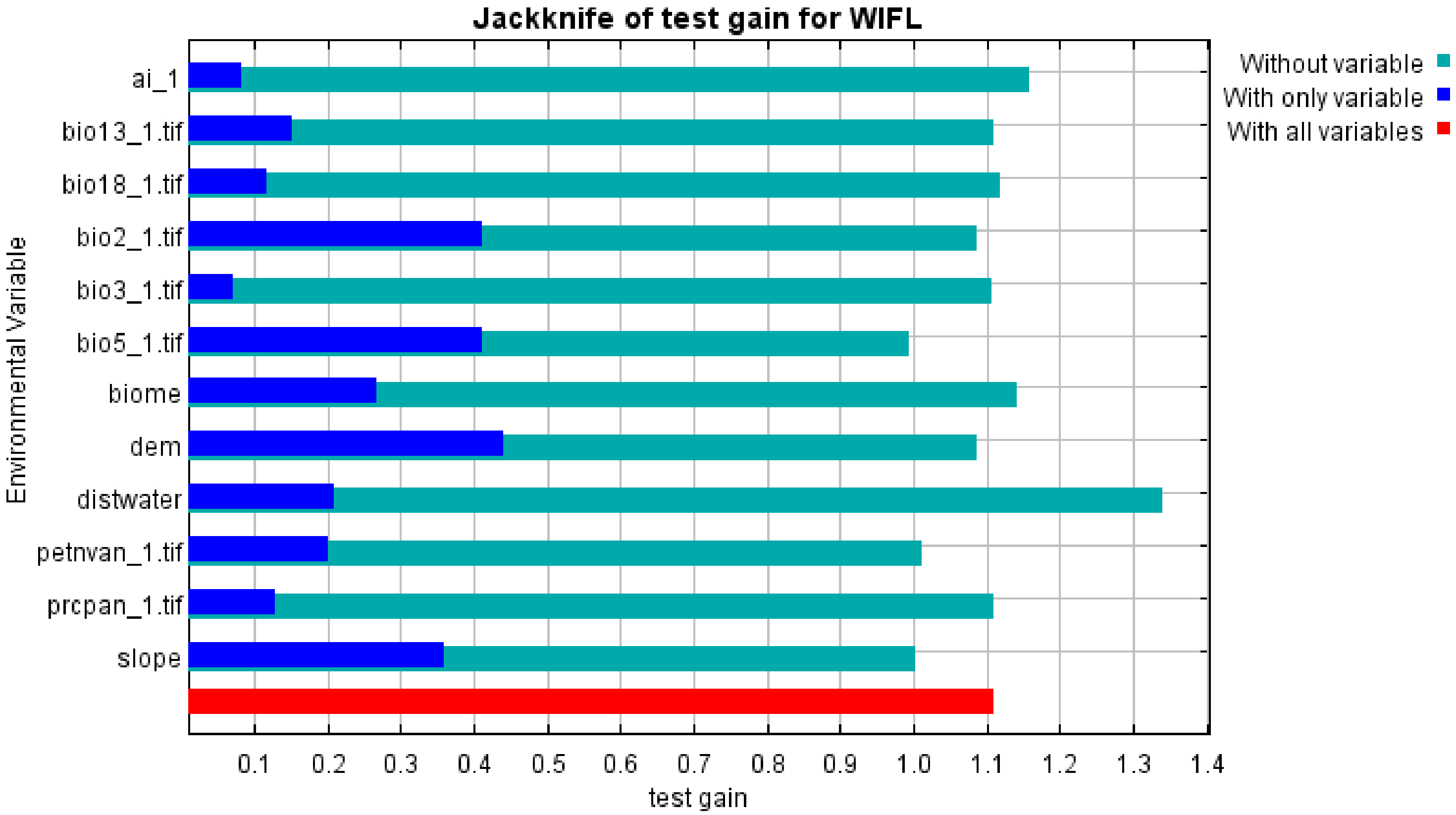

Lastly, we have the same jackknife test, using AUC on test data.

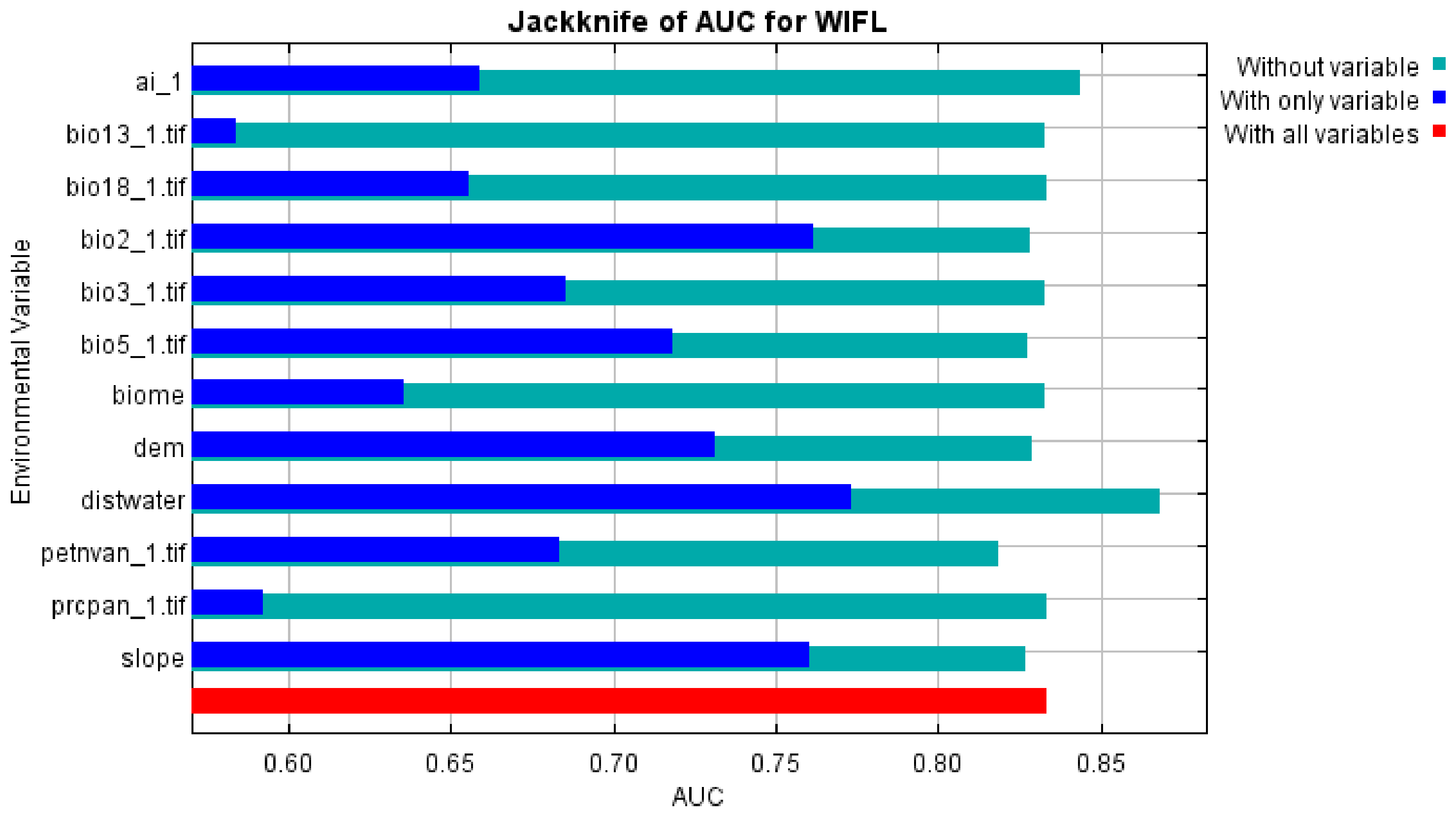

Command line to repeat this species model: java density.MaxEnt nowarnings noprefixes -E "" -E WIFL responsecurves jackknife outputdirectory=D:\MaxEnt4\BirdsCCC "projectionlayers=D:\Ascii_Files\Envir_2030_ascii, D:\Ascii_Files\Envir_2060_ascii, D:\Ascii_Files\Envir_2090_ascii" samplesfile=D:\Data\RioGrande\Birds\allbirds.csv environmentallayers=D:\Ascii_Files\Envir_Curr_ascii randomseed noaskoverwrite biasfile=D:\Data\RioGrande\Birds\all_bias.asc replicates=15 nooutputgrids maximumiterations=5000 biastype=3 "applythresholdrule=equal training sensitivity and specificity" -N ai_2 -N ai_3 -N bio10_1 -N bio10_1.tif -N bio10_2 -N bio10_2.tif -N bio10_3 -N bio10_3.tif -N bio11_1 -N bio11_1.tif -N bio11_2 -N bio11_2.tif -N bio11_3 -N bio11_3.tif -N bio13_2.tif -N bio13_3.tif -N bio14_1.tif -N bio14_2.tif -N bio14_3.tif -N bio16_1.tif -N bio16_2.tif -N bio16_3.tif -N bio17_.tif -N bio17_2.tif -N bio17_3.tif -N bio17_all.tif -N bio18_2.tif -N bio18_3.tif -N bio19_1.tif -N bio19_2.tif -N bio19_3.tif -N bio2_2.tif -N bio2_3.tif -N bio3_2.tif -N bio3_3.tif -N bio5_2.tif -N bio5_3.tif -N bio6_1.tif -N bio6_2.tif -N bio6_3.tif -N bio7_1.tif -N bio7_2.tif -N bio7_3.tif -N etan_1.tif -N etan_2.tif -N etan_3.tif -N etdjf_1.tif -N etdjf_2.tif -N etdjf_3.tif -N etjas_1.tif -N etjas_2.tif -N etjas_3.tif -N etjfm_1.tif -N etjfm_2.tif -N etjfm_3.tif -N etjja_1.tif -N etjja_2.tif -N etjja_3.tif -N etjja_all.tif -N petnvan_2.tif -N petnvan_3.tif -N petnvdjf_1.tif -N petnvdjf_2.tif -N petnvdjf_3.tif -N petnvjas_1.tif -N petnvjas_2.tif -N petnvjas_3.tif -N petnvjfm_1.tif -N petnvjfm_2.tif -N petnvjfm_3.tif -N petnvjja_1.tif -N petnvjja_2.tif -N petnvjja_3.tif -N petwan_1.tif -N petwan_2.tif -N petwan_3.tif -N petwdjf_1.tif -N petwdjf_2.tif -N petwdjf_3.tif -N petwjas_1.tif -N petwjas_2.tif -N petwjas_3.tif -N petwjas_all.tif -N petwjfm_1.tif -N petwjfm_2.tif -N petwjfm_3.tif -N petwjja_1.tif -N petwjja_2.tif -N petwjja_3.tif -N prcpan_2.tif -N prcpan_3.tif -N prcpan_all.tif -N runoffan_1.tif -N runoffan_2.tif -N runoffan_3.tif -N runoffdjf_1.tif -N runoffdjf_2.tif -N runoffdjf_3.tif -N runoffjas_1.tif -N runoffjas_2.tif -N runoffjas_3.tif -N runoffjas_all.tif -N runoffjfm_1.tif -N runoffjfm_2.tif -N runoffjfm_3.tif -N runoffjja_1.tif -N runoffjja_2.tif -N runoffjja_3.tif -N smcan_1.tif -N smcan_2.tif -N smcan_3.tif -N smcdjf_1.tif -N smcdjf_2.tif -N smcdjf_3.tif -N smcjas_1.tif -N smcjas_2.tif -N smcjas_3.tif -N smcjfm_1.tif -N smcjfm_2.tif -N smcjfm_3.tif -N smcjja_1.tif -N smcjja_2.tif -N smcjja_3.tif -N swean_1.tif -N swean_2.tif -N swean_3.tif -N swedjf_1.tif -N swedjf_2.tif -N swedjf_3.tif -N swejfm_1.tif -N swejfm_2.tif -N swejfm_3.tif -N tave_1.tif -N tave_2.tif -N tave_3.tif -N tmax_1.tif -N tmax_2.tif -N tmax_3.tif -N tmin_1.tif -N tmin_2.tif -N tmin_3.tif -t biome

Replicated maxent model for WIFL

This page summarizes the results of 15-fold cross-validation for WIFL, created Mon Aug 04 16:05:48 MDT 2014 using Maxent version 3.3.3k. The individual models are here: [0] [1] [2] [3] [4] [5] [6] [7] [8] [9] [10] [11] [12] [13] [14]

Analysis of omission/commission

The following picture shows the test omission rate and predicted area as a function of the cumulative threshold, averaged over the replicate runs. The omission rate should be close to the predicted omission, because of the definition of the cumulative threshold.

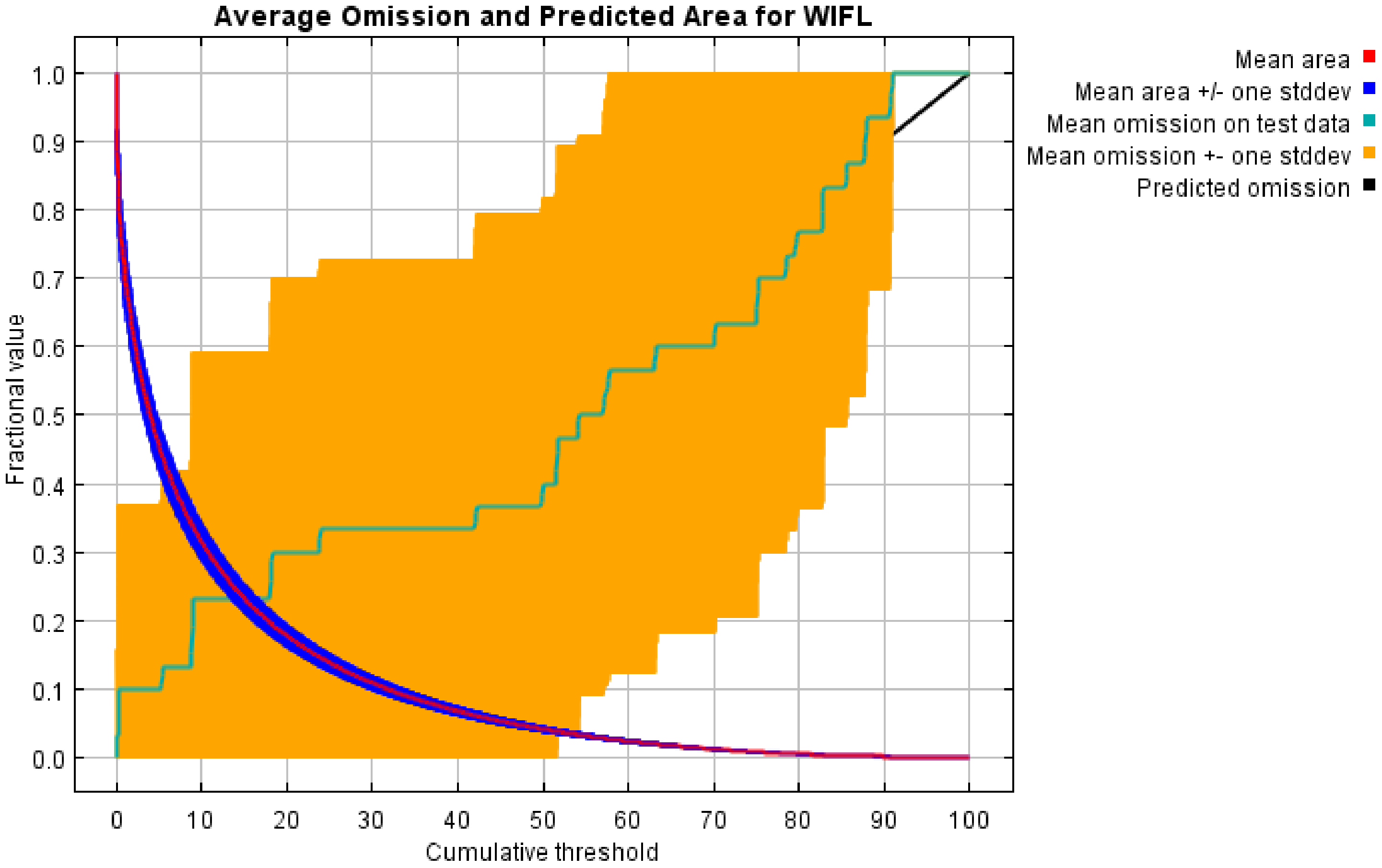

The next picture is the receiver operating characteristic (ROC) curve for the same data, again averaged over the replicate runs. Note that the specificity is defined using predicted area, rather than true commission (see the paper by Phillips, Anderson and Schapire cited on the help page for discussion of what this means). The average test AUC for the replicate runs is 0.845, and the standard deviation is 0.207.

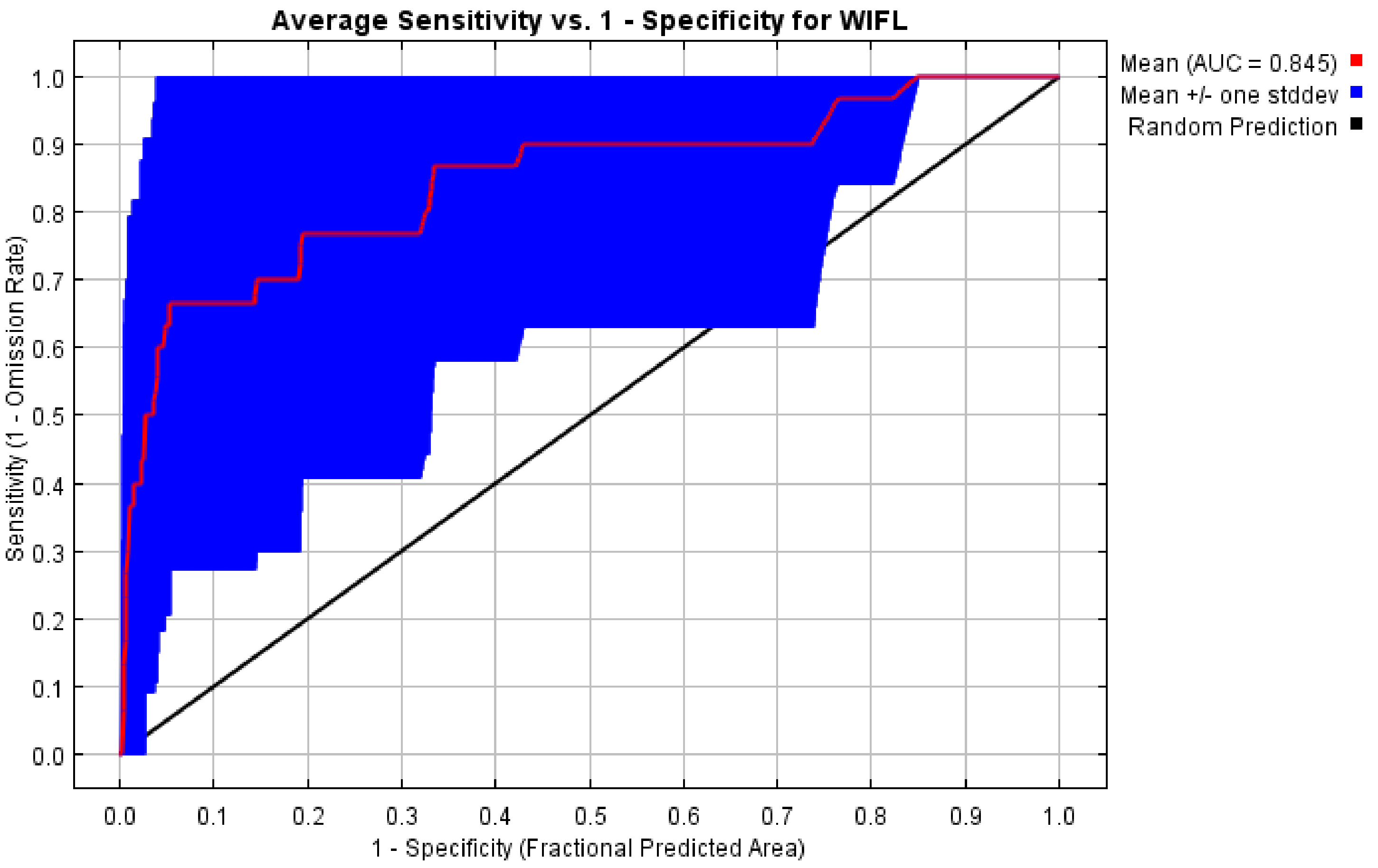

Pictures of the model

The following two pictures show the point-wise mean and standard deviation of the 15 output grids. Other available summary grids are min, max and median.

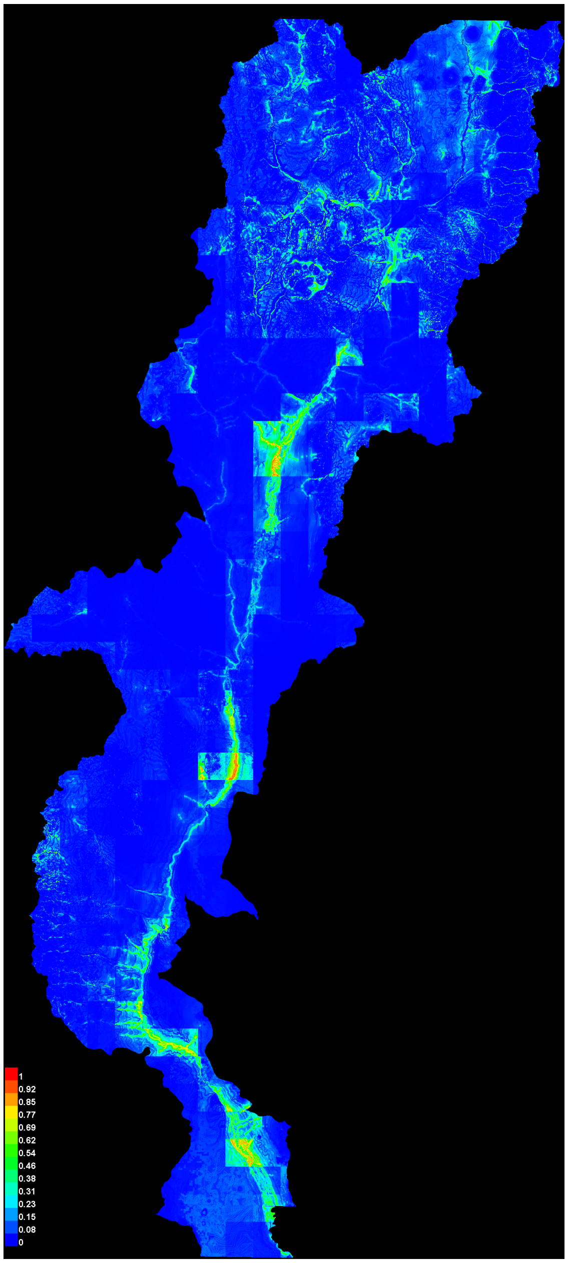

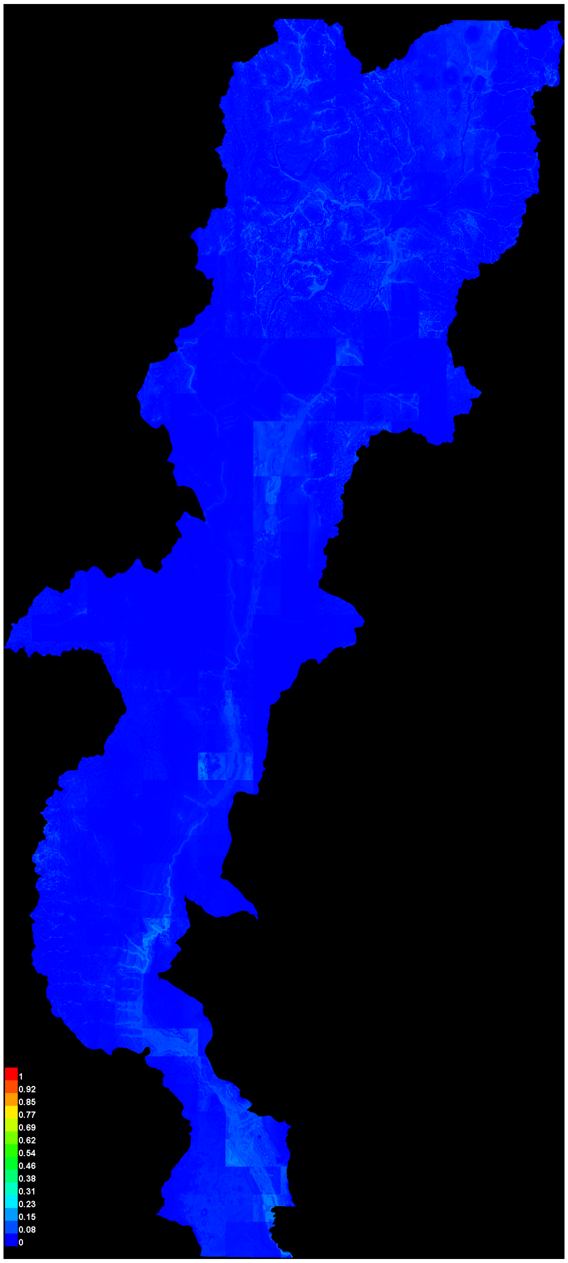

The following two pictures show the point-wise mean and standard deviation of the 15 models applied to the environmental layers in Envir_2030_ascii. Other available summary grids are min, max and median.

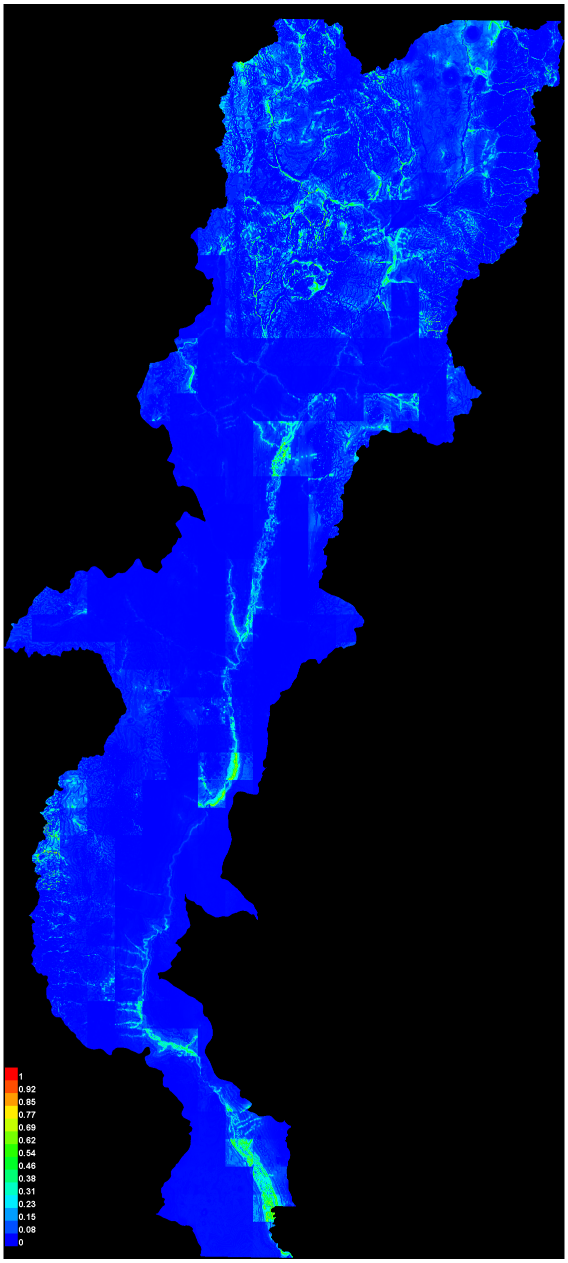

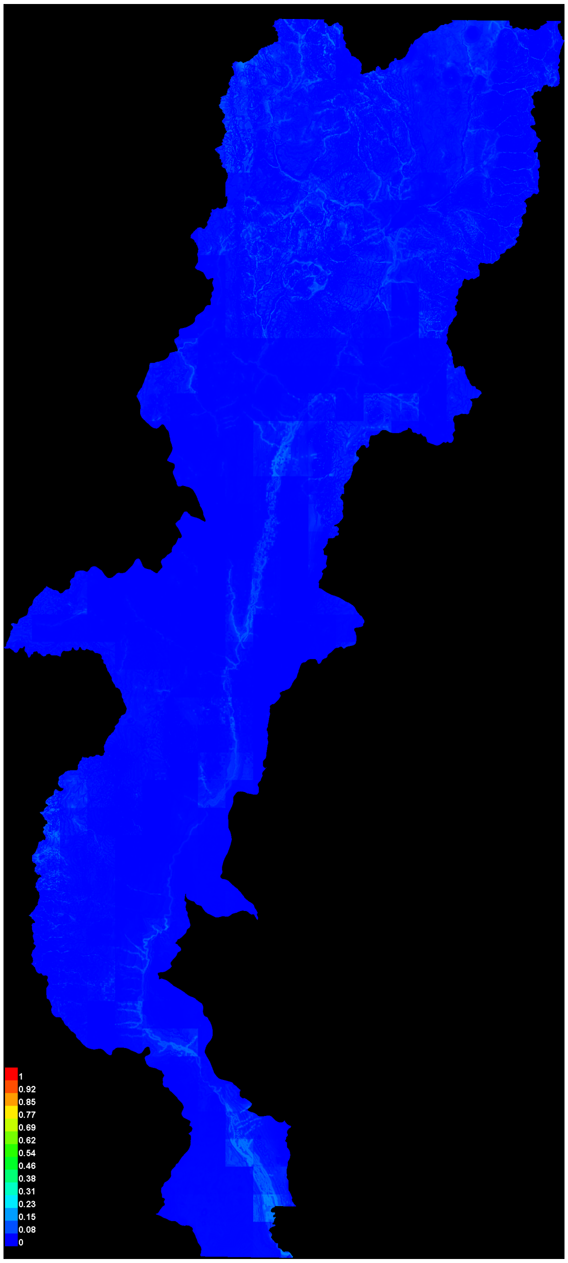

The following two pictures show the point-wise mean and standard deviation of the 15 models applied to the environmental layers in Envir_2060_ascii. Other available summary grids are min, max and median.

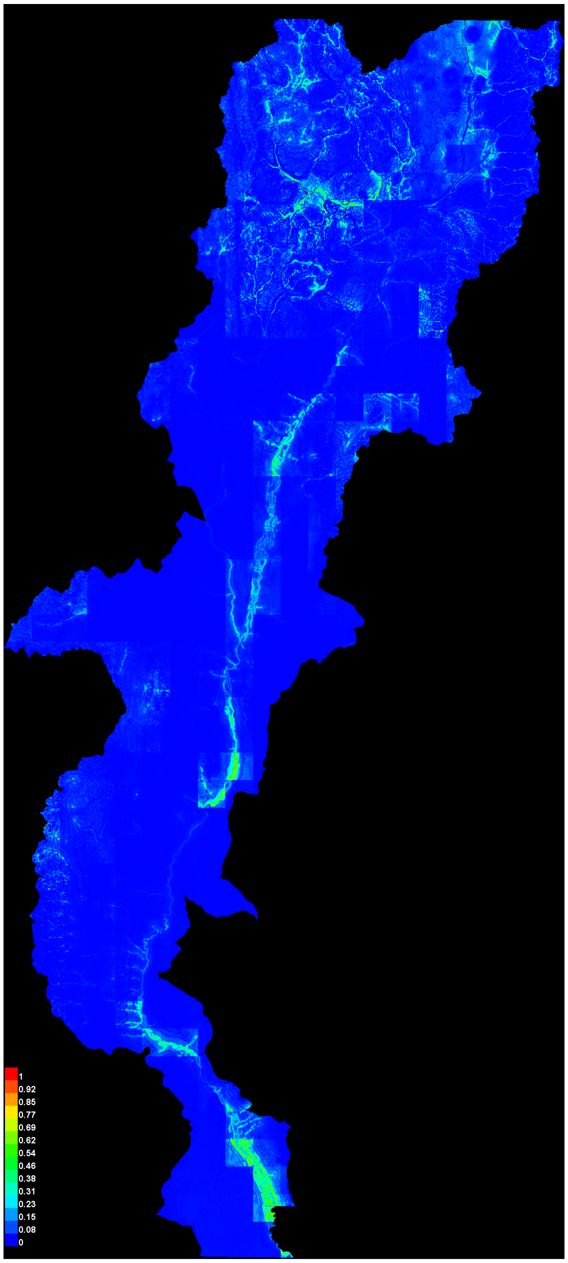

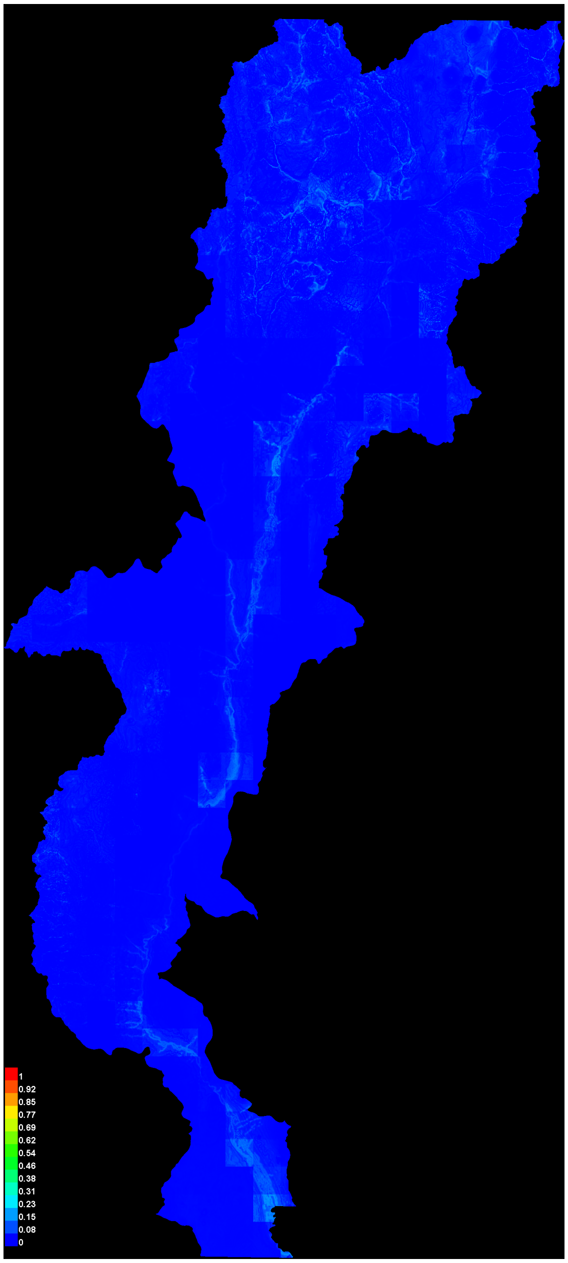

The following two pictures show the point-wise mean and standard deviation of the 15 models applied to the environmental layers in Envir_2090_ascii. Other available summary grids are min, max and median.

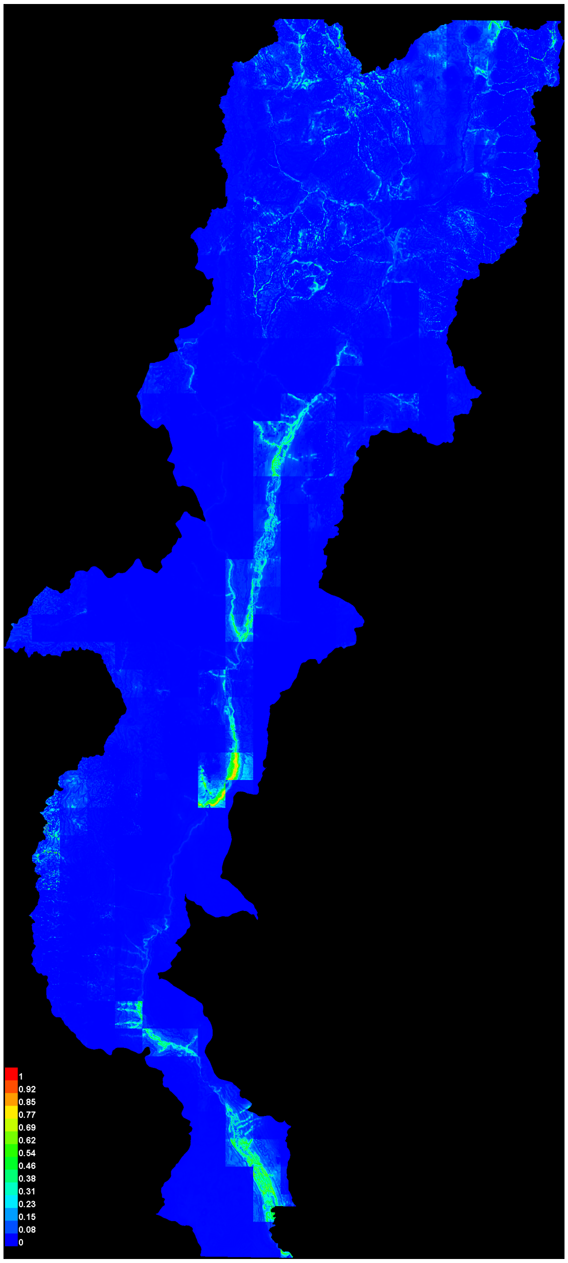

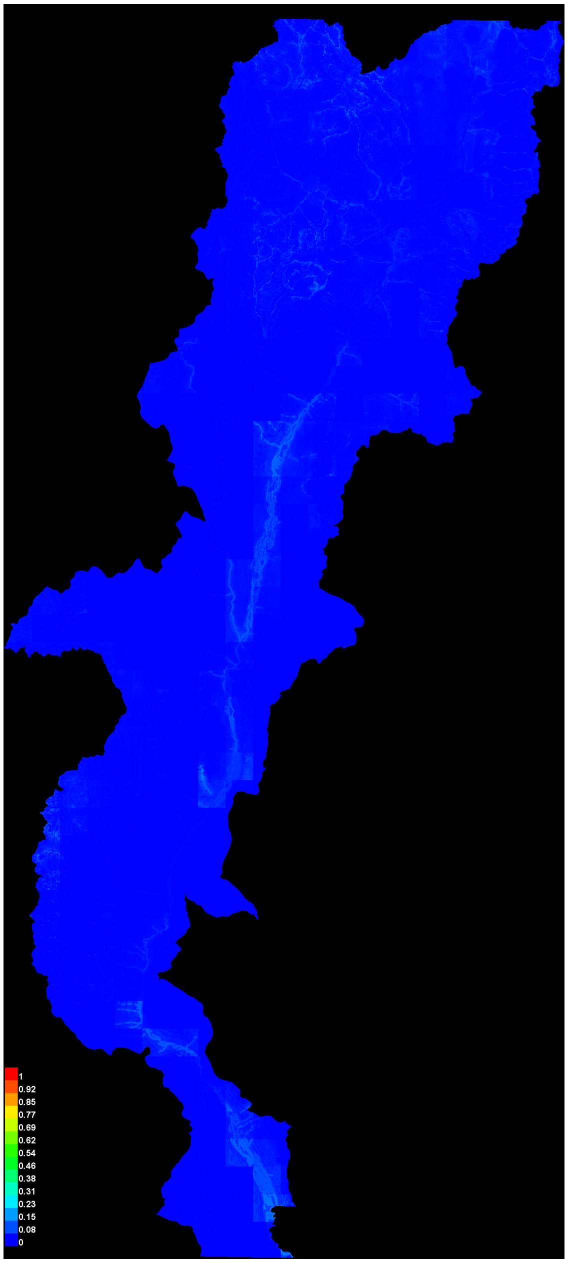

Response curves

These curves show how each environmental variable affects the Maxent prediction. The curves show how the logistic prediction changes as each environmental variable is varied, keeping all other environmental variables at their average sample value. Click on a response curve to see a larger version. Note that the curves can be hard to interpret if you have strongly correlated variables, as the model may depend on the correlations in ways that are not evident in the curves. In other words, the curves show the marginal effect of changing exactly one variable, whereas the model may take advantage of sets of variables changing together. The curves show the mean response of the 15 replicate Maxent runs (red) and and the mean +/- one standard deviation (blue, two shades for categorical variables).

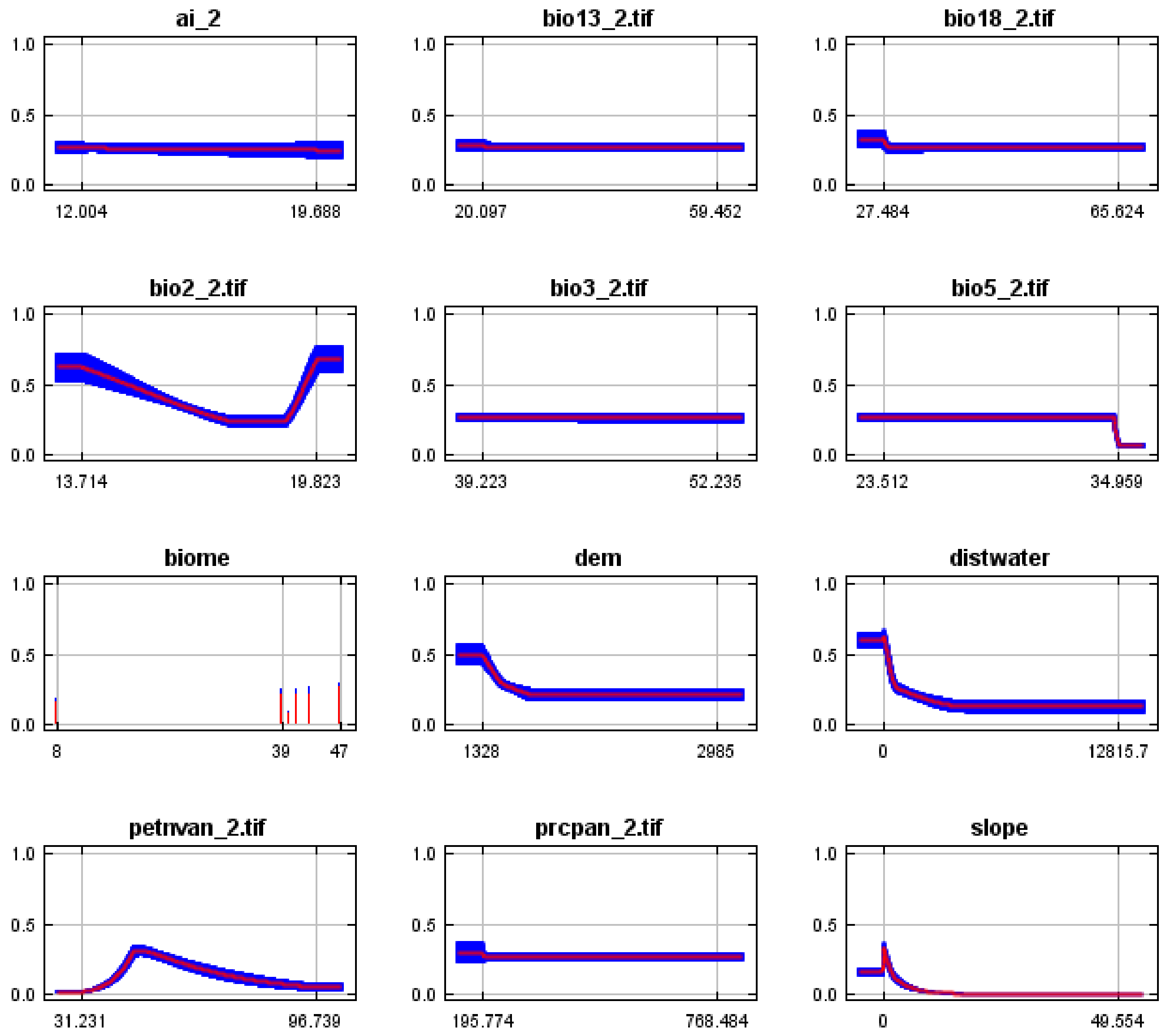

In contrast to the above marginal response curves, each of the following curves represents a different model, namely, a Maxent model created using only the corresponding variable. These plots reflect the dependence of predicted suitability both on the selected variable and on dependencies induced by correlations between the selected variable and other variables. They may be easier to interpret if there are strong correlations between variables.

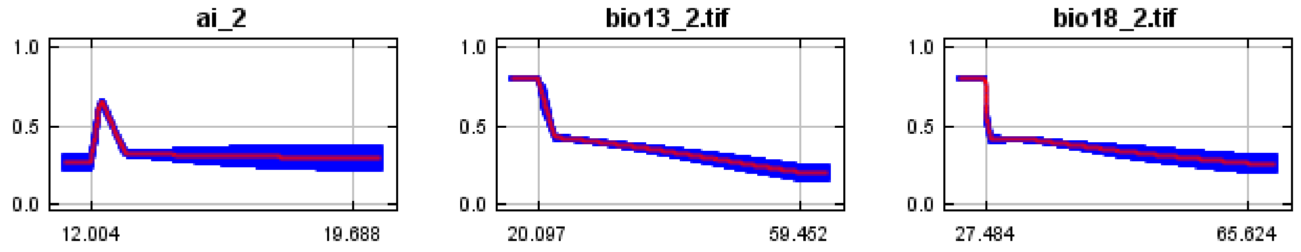

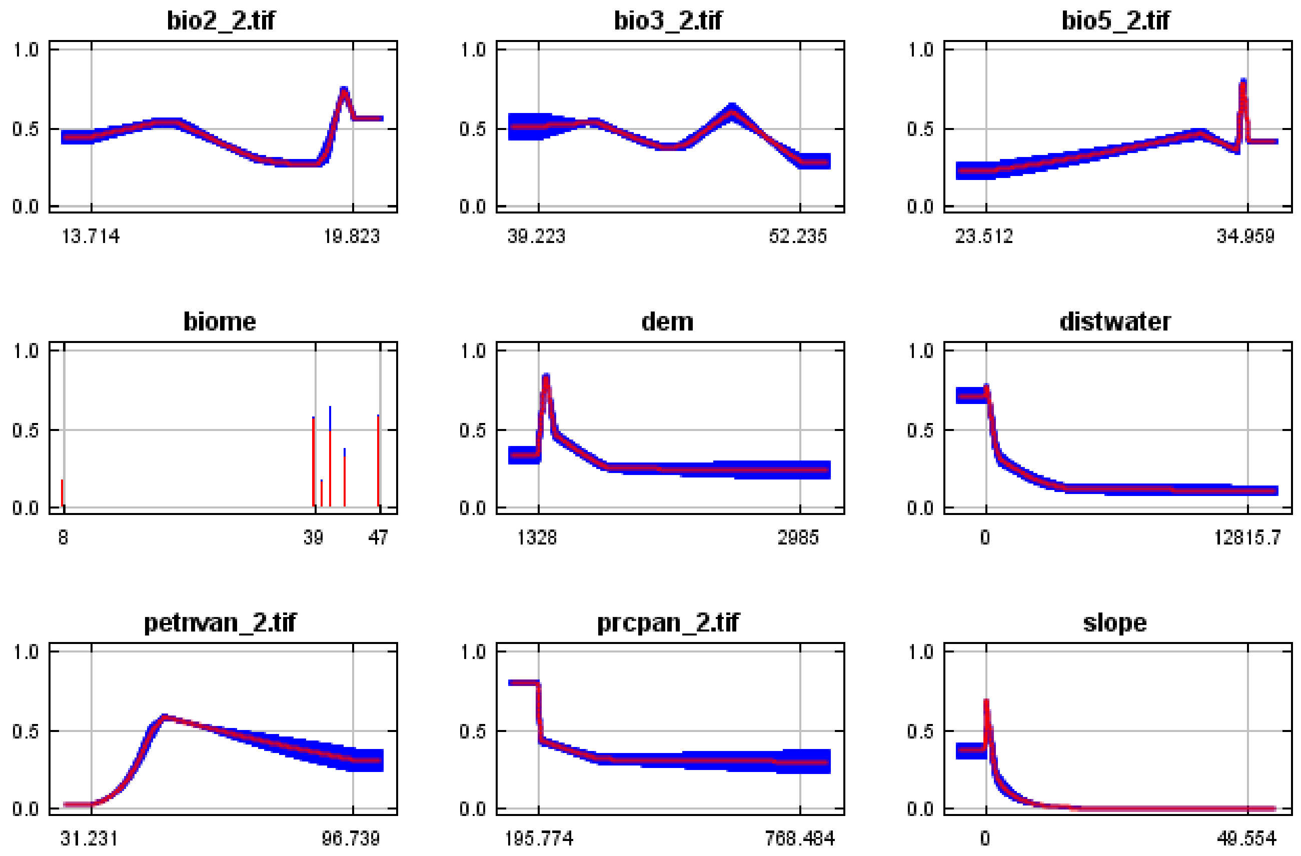

Analysis of variable contributions

The following table gives estimates of relative contributions of the environmental variables to the Maxent model. To determine the first estimate, in each iteration of the training algorithm, the increase in regularized gain is added to the contribution of the corresponding variable, or subtracted from it if the change to the absolute value of lambda is negative. For the second estimate, for each environmental variable in turn, the values of that variable on training presence and background data are randomly permuted. The model is reevaluated on the permuted data, and the resulting drop in training AUC is shown in the table, normalized to percentages. As with the variable jackknife, variable contributions should be interpreted with caution when the predictor variables are correlated. Values shown are averages over replicate runs.

Variable	Percent contribution	Permutation importance
distwater	42.6	26.8
slope	12.8	21.2
biome	11.2	5
bio2_2.tif	11	10.6
petnvan_2.tif	5.2	32.9
bio18_2.tif	4.8	0.1
prcpan_2.tif	4.2	0
bio13_2.tif	3.7	0

bio5_2.tif	3	2.1
dem	1.4	1.2
ai_2	0.2	0.1
bio3_2.tif	0	0.1

The following picture shows the results of the jackknife test of variable importance. The environmental variable with highest gain when used in isolation is distwater, which therefore appears to have the most useful information by itself. The environmental variable that decreases the gain the most when it is omitted is distwater, which therefore appears to have the most information that isn't present in the other variables. Values shown are averages over replicate runs.

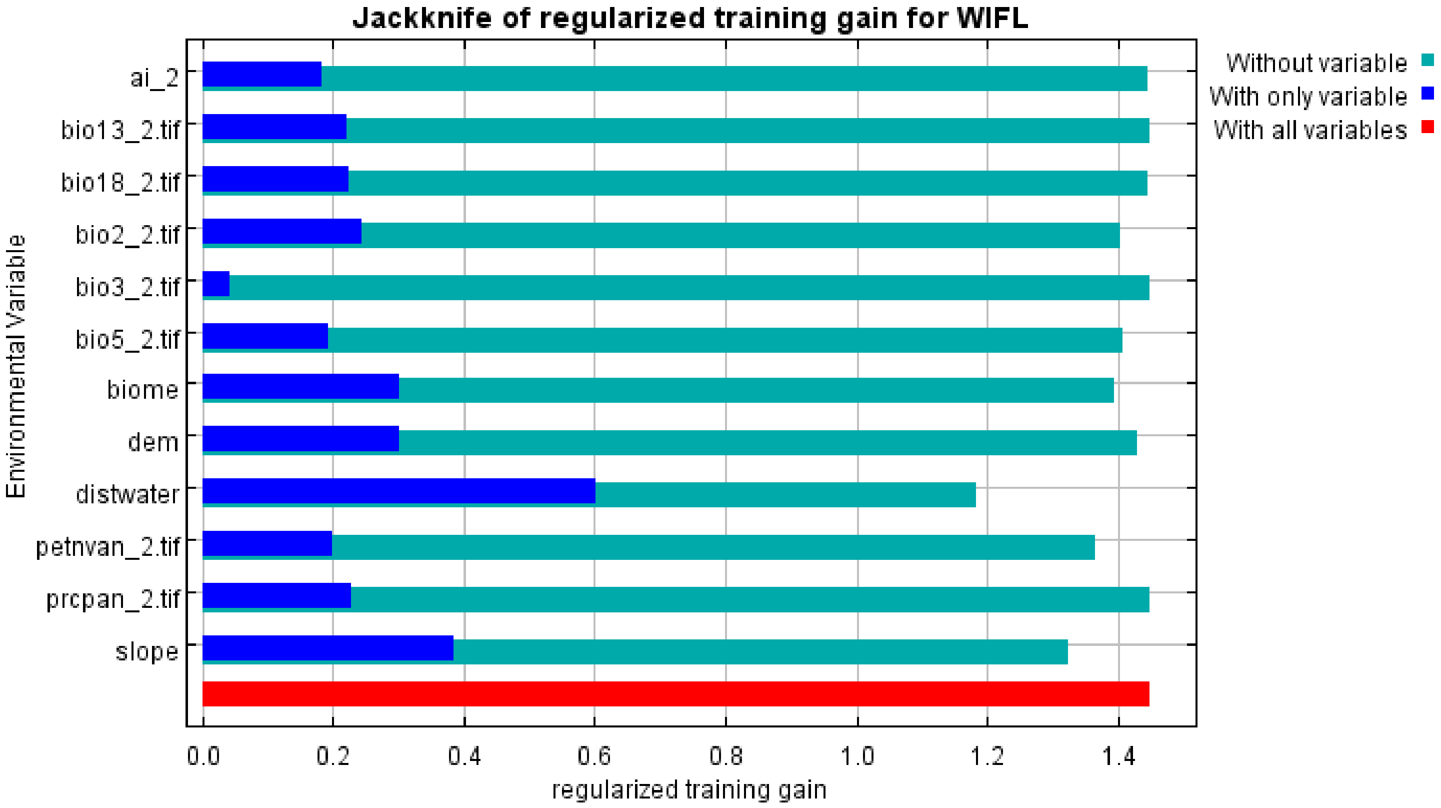

The next picture shows the same jackknife test, using test gain instead of training gain. Note that conclusions about which variables are most important can change, now that we're looking at test data.

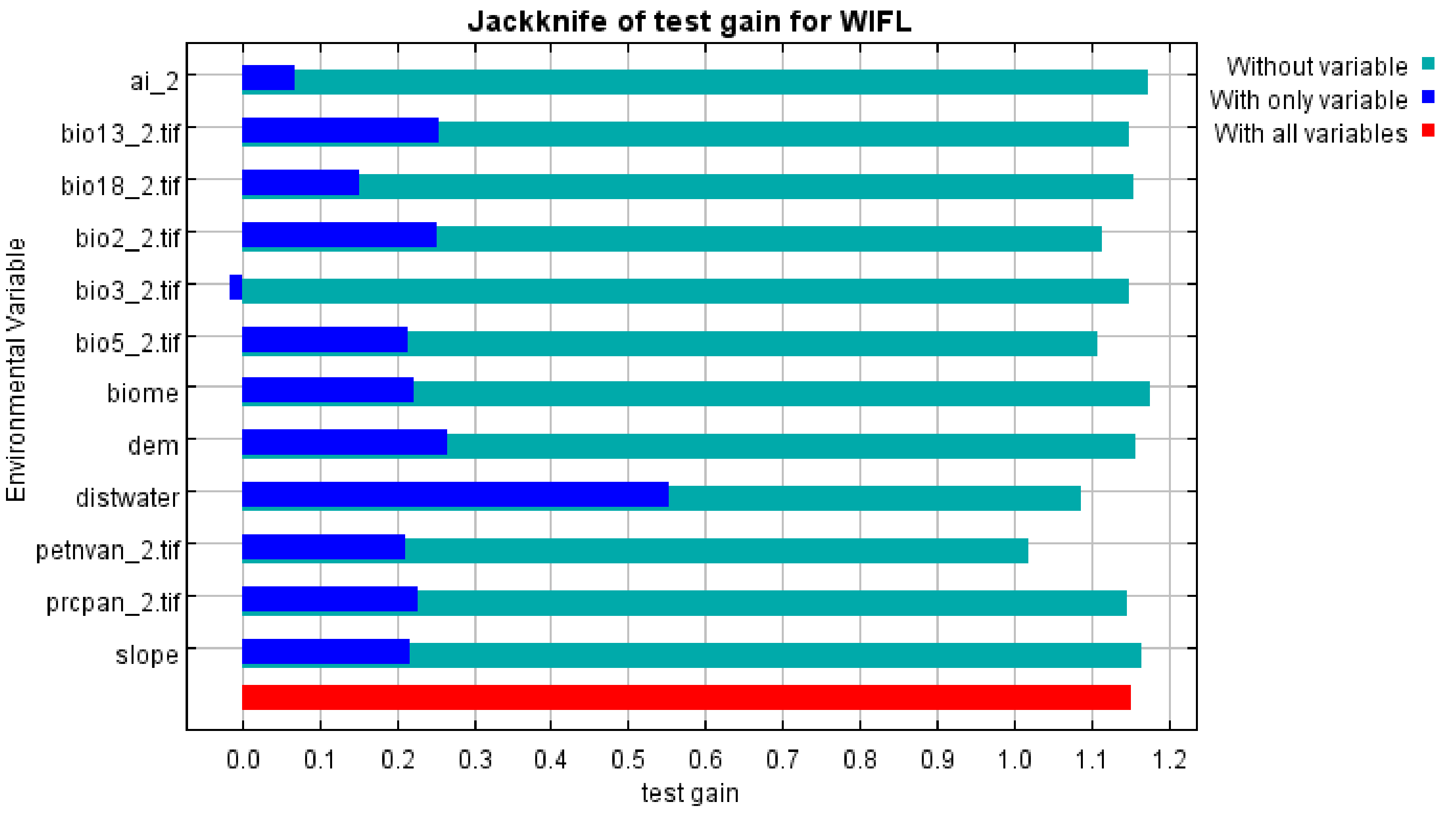

Lastly, we have the same jackknife test, using AUC on test data.

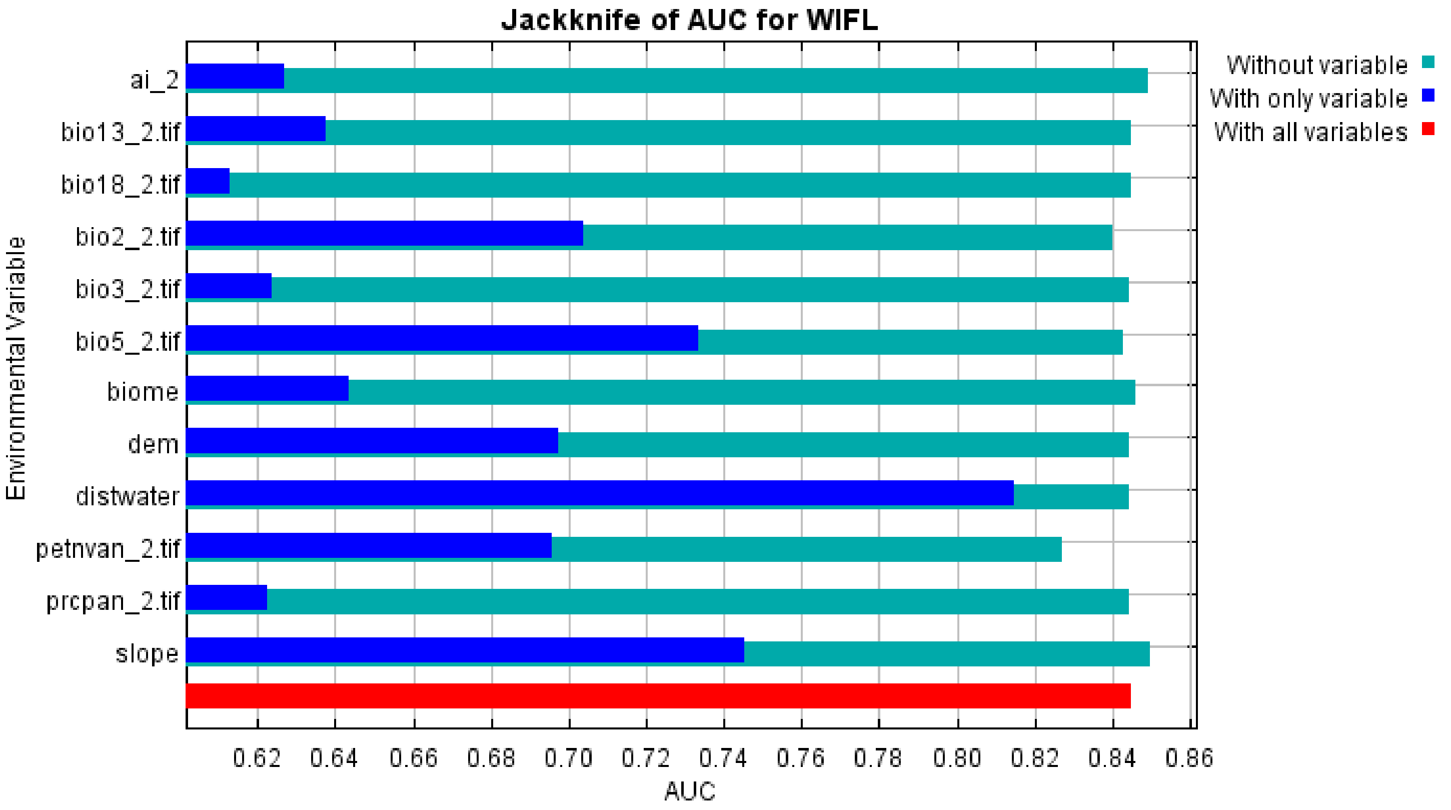

Command line to repeat this species model: java density.MaxEnt nowarnings noprefixes -E "" -E WIFL responsecurves jackknife outputdirectory=D:\MaxEnt4\BirdsGd

"projectionlayers=D:\Ascii_Files\Envir_2030_ascii, D:\Ascii_Files\Envir_2060_ascii, D:\Ascii_Files\Envir_2090_ascii" samplesfile=D:\Data\RioGrande\Birds\allbirds.csv environmentallayers=D:\Ascii_Files\Envir_Curr_ascii randomseed biasfile=D:\Data\RioGrande\Birds\all_bias.asc replicates=15 nooutputgrids maximumiterations=5000 biastype=3 "applythresholdrule=equal training sensitivity and specificity" -N ai_1 -N ai_3 -N bio10_1 -N bio10_1.tif -N bio10_2 -N bio10_2.tif -N bio10_3 -N bio10_3.tif -N bio11_1 -N bio11_1.tif -N bio11_2 -N bio11_2.tif -N bio11_3 -N bio11_3.tif -N bio13_1.tif -N bio13_3.tif -N bio14_1.tif -N bio14_2.tif -N bio14_3.tif -N bio16_1.tif -N bio16_2.tif -N bio16_3.tif -N bio17_.tif -N bio17_2.tif -N bio17_3.tif -N bio17_all.tif -N bio18_1.tif -N bio18_3.tif -N bio19_1.tif -N bio19_2.tif -N bio19_3.tif -N bio2_1.tif -N bio2_3.tif -N bio3_1.tif -N bio3_3.tif -N bio5_1.tif -N bio5_3.tif -N bio6_1.tif -N bio6_2.tif -N bio6_3.tif -N bio7_1.tif -N bio7_2.tif -N bio7_3.tif -N etan_1.tif -N etan_2.tif -N etan_3.tif -N etdjf_1.tif -N etdjf_2.tif -N etdjf_3.tif -N etjas_1.tif -N etjas_2.tif -N etjas_3.tif -N etjfm_1.tif -N etjfm_2.tif -N etjfm_3.tif -N etjja_1.tif -N etjja_2.tif -N etjja_3.tif -N etjja_all.tif -N petnvan_1.tif -N petnvan_3.tif -N petnvdjf_1.tif -N petnvdjf_2.tif -N petnvdjf_3.tif -N petnvjas_1.tif -N petnvjas_2.tif -N petnvjas_3.tif -N petnvjfm_1.tif -N petnvjfm_2.tif -N petnvjfm_3.tif -N petnvjja_1.tif -N petnvjja_2.tif -N petnvjja_3.tif -N petwan_1.tif -N petwan_2.tif -N petwan_3.tif -N petwdjf_1.tif -N petwdjf_2.tif -N petwdjf_3.tif -N petwjas_1.tif -N petwjas_2.tif -N petwjas_3.tif -N petwjas_all.tif -N petwjfm_1.tif -N petwjfm_2.tif -N petwjfm_3.tif -N petwjja_1.tif -N petwjja_2.tif -N petwjja_3.tif -N prcpan_1.tif -N prcpan_3.tif -N prcpan_all.tif -N runoffan_1.tif -N runoffan_2.tif -N runoffan_3.tif -N runoffdjf_1.tif -N runoffdjf_2.tif -N runoffdjf_3.tif -N runoffjas_1.tif -N runoffjas_2.tif -N runoffjas_3.tif -N runoffjas_all.tif -N runoffjfm_1.tif -N runoffjfm_2.tif -N runoffjfm_3.tif -N runoffjja_1.tif -N runoffjja_2.tif -N runoffjja_3.tif -N smcan_1.tif -N smcan_2.tif -N smcan_3.tif -N smcdjf_1.tif -N smcdjf_2.tif -N smcdjf_3.tif -N smcjas_1.tif -N smcjas_2.tif -N smcjas_3.tif -N smcjfm_1.tif -N smcjfm_2.tif -N smcjfm_3.tif -N smcjja_1.tif -N smcjja_2.tif -N smcjja_3.tif -N swean_1.tif -N swean_2.tif -N swean_3.tif -N swedjf_1.tif -N swedjf_2.tif -N swedjf_3.tif -N swejfm_1.tif -N swejfm_2.tif -N swejfm_3.tif -N tave_1.tif -N tave_2.tif -N tave_3.tif -N tmax_1.tif -N tmax_2.tif -N tmax_3.tif -N tmin_1.tif -N tmin_2.tif -N tmin_3.tif -t biome

Replicated maxent model for WIFL

This page summarizes the results of 15-fold cross-validation for WIFL, created Wed Aug 06 10:08:52 MDT 2014 using Maxent version 3.3.3k. The individual models are here: [0] [1] [2] [3] [4] [5] [6] [7] [8] [9] [10] [11] [12] [13] [14]

Analysis of omission/commission

The following picture shows the test omission rate and predicted area as a function of the cumulative threshold, averaged over the replicate runs. The omission rate should be close to the predicted omission, because of the definition of the cumulative threshold.

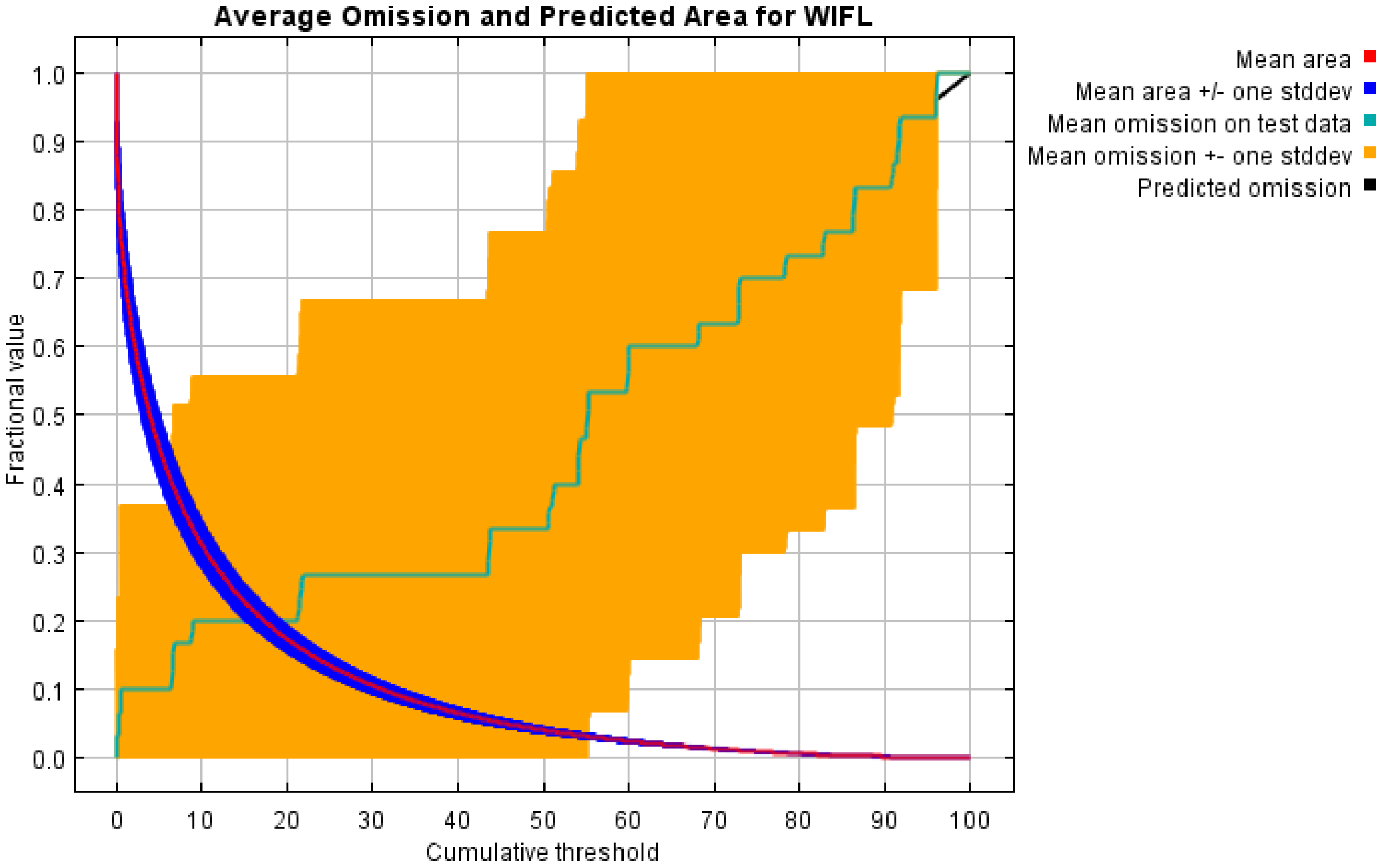

The next picture is the receiver operating characteristic (ROC) curve for the same data, again averaged over the replicate runs. Note that the specificity is defined using predicted area, rather than true commission (see the paper by Phillips, Anderson and Schapire cited on the help page for discussion of what this means). The average test AUC for the replicate runs is 0.865, and the standard deviation is 0.203.

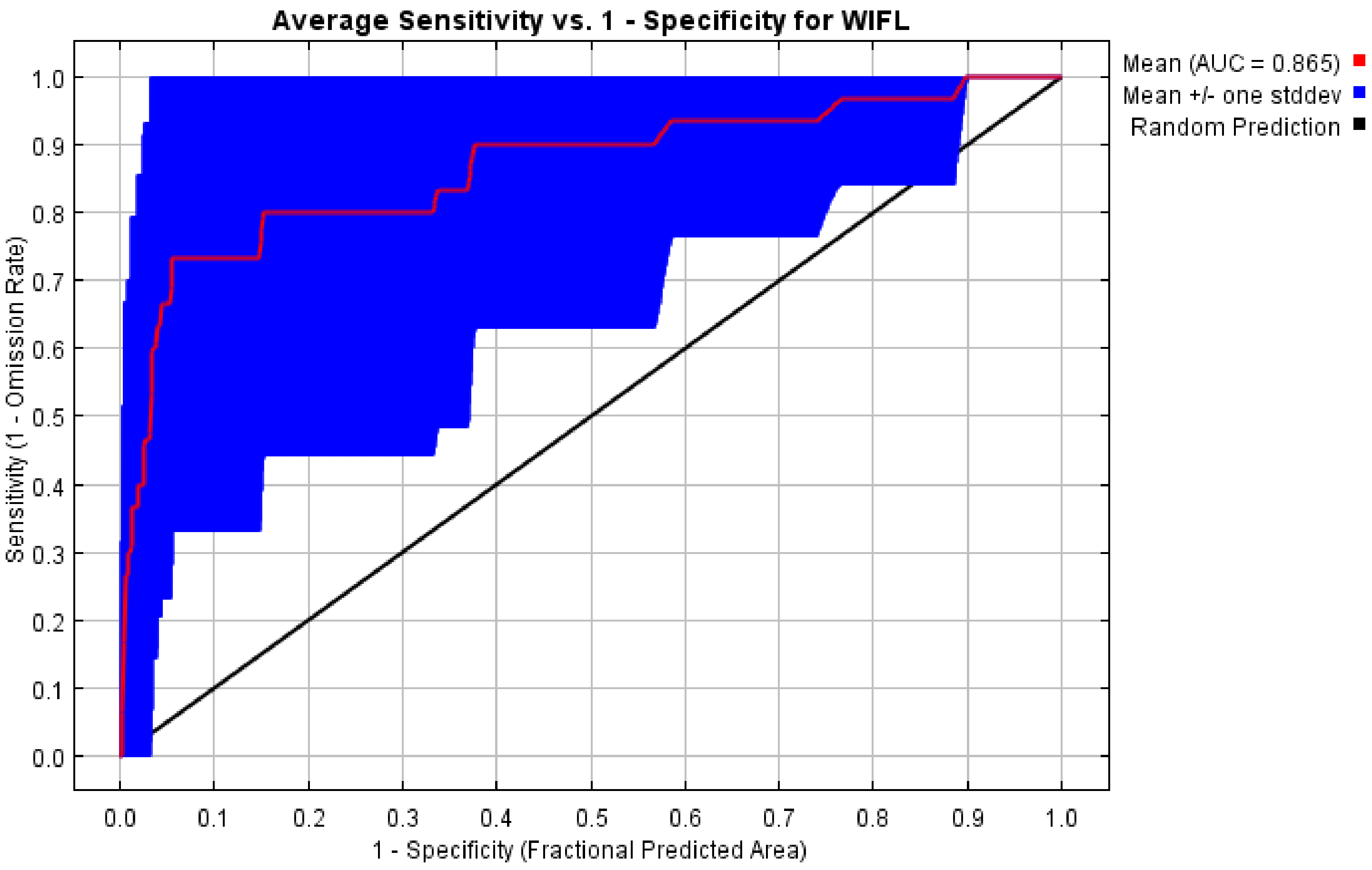

Pictures of the model

The following two pictures show the point-wise mean and standard deviation of the 15 output grids. Other available summary grids are min, max and median.

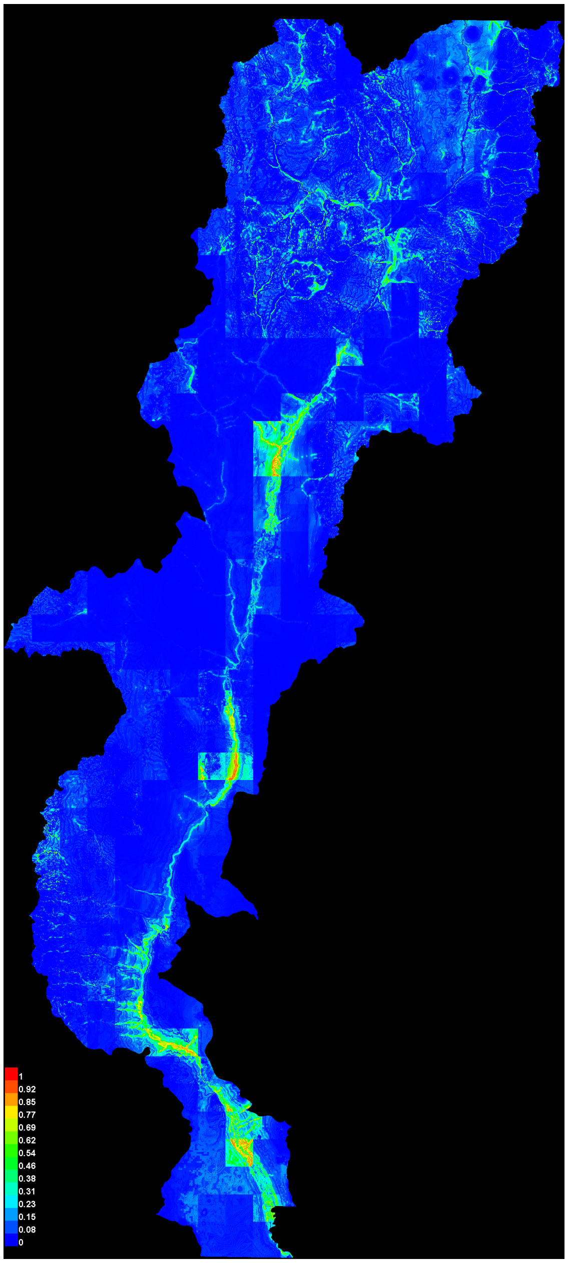

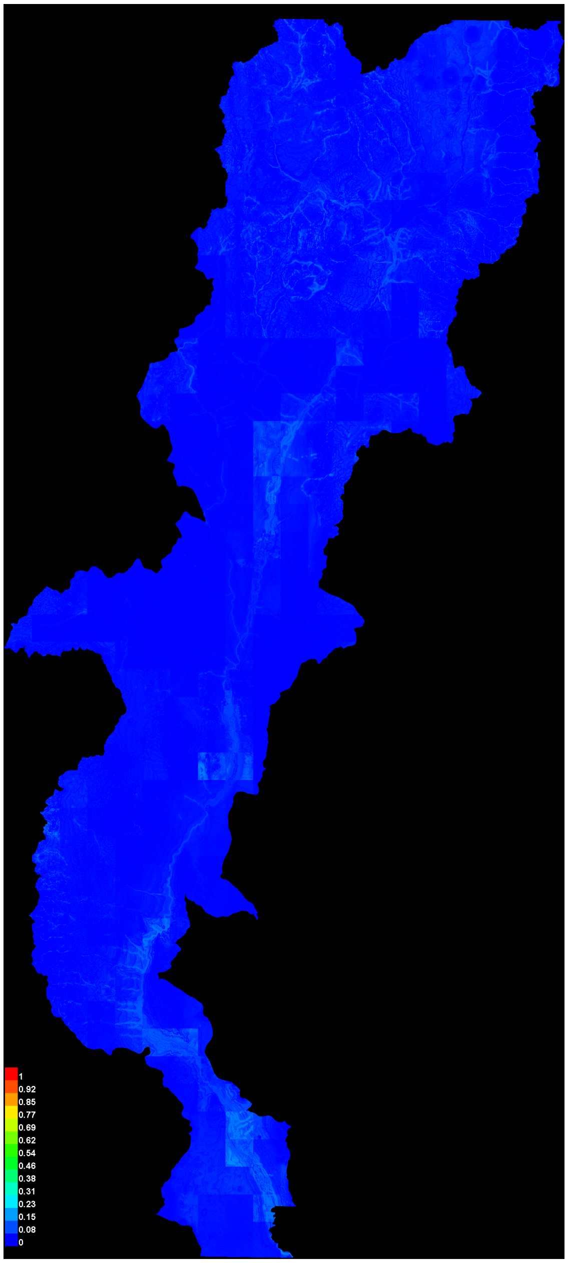

The following two pictures show the point-wise mean and standard deviation of the 15 models applied to the environmental layers in Envir_2030_ascii. Other available summary grids are min, max and median.

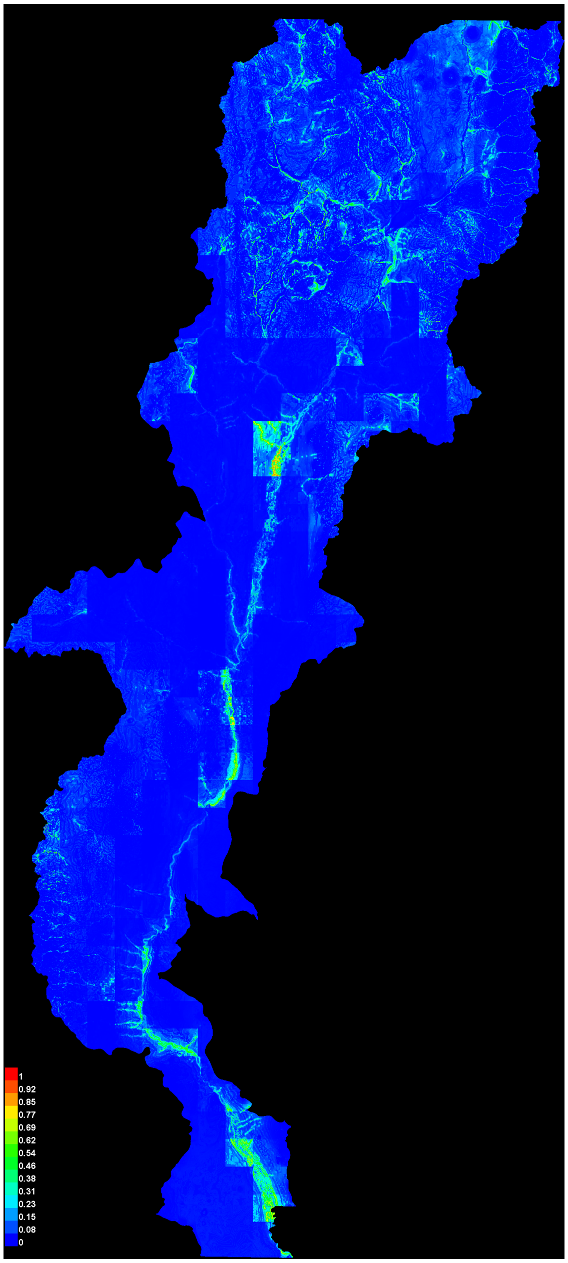

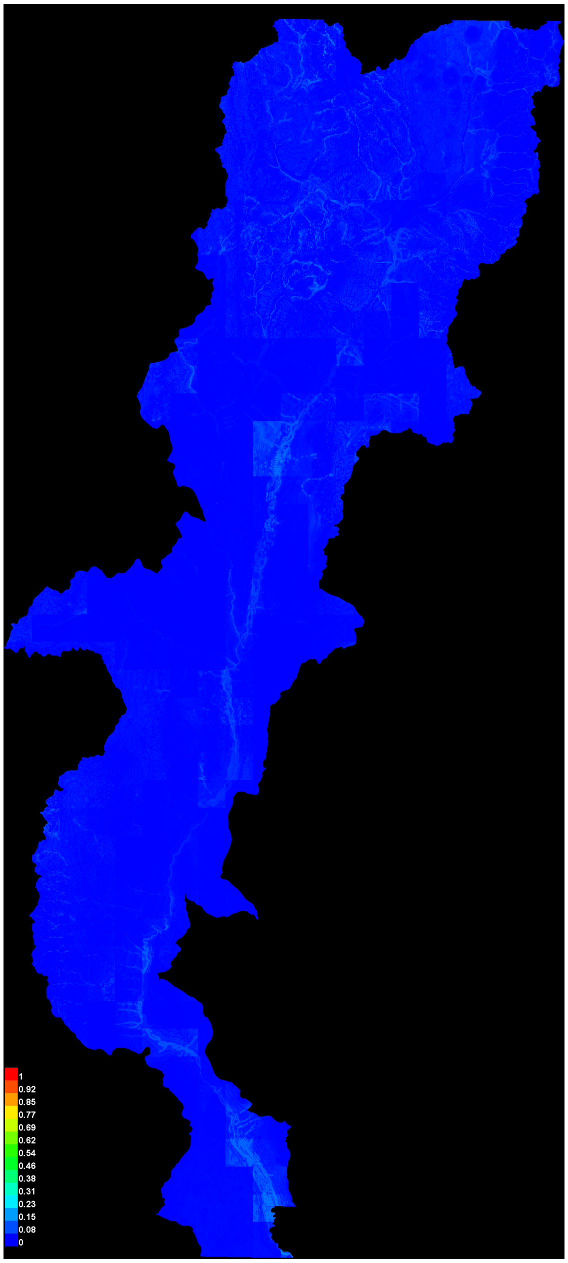

The following two pictures show the point-wise mean and standard deviation of the 15 models applied to the environmental layers in Envir_2060_ascii. Other available summary grids are min, max and median.

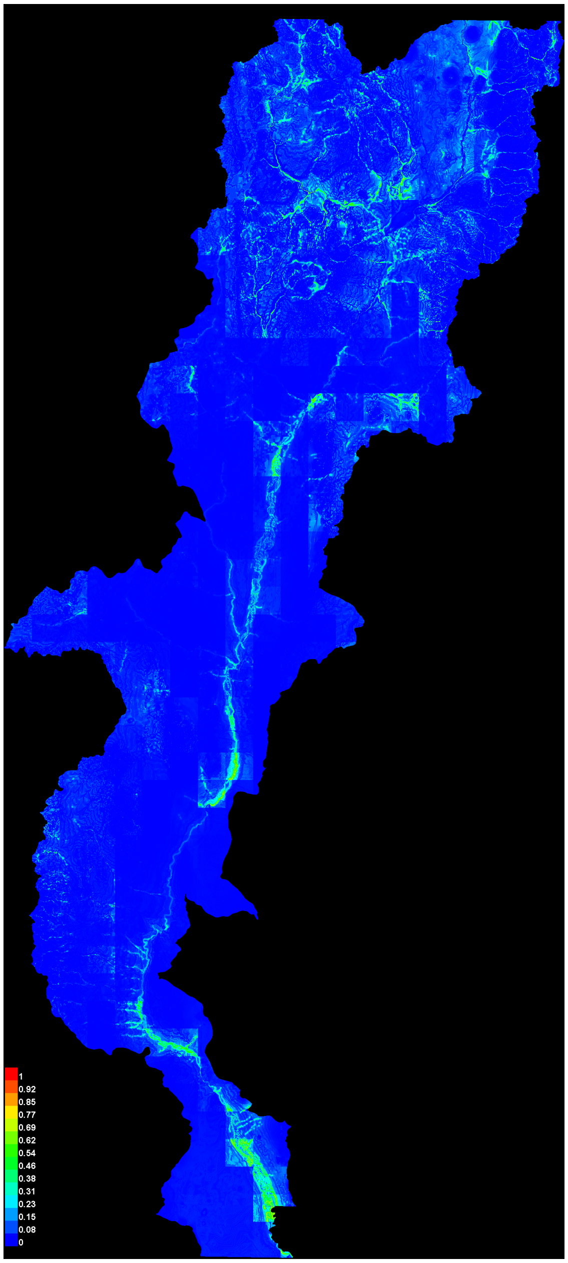

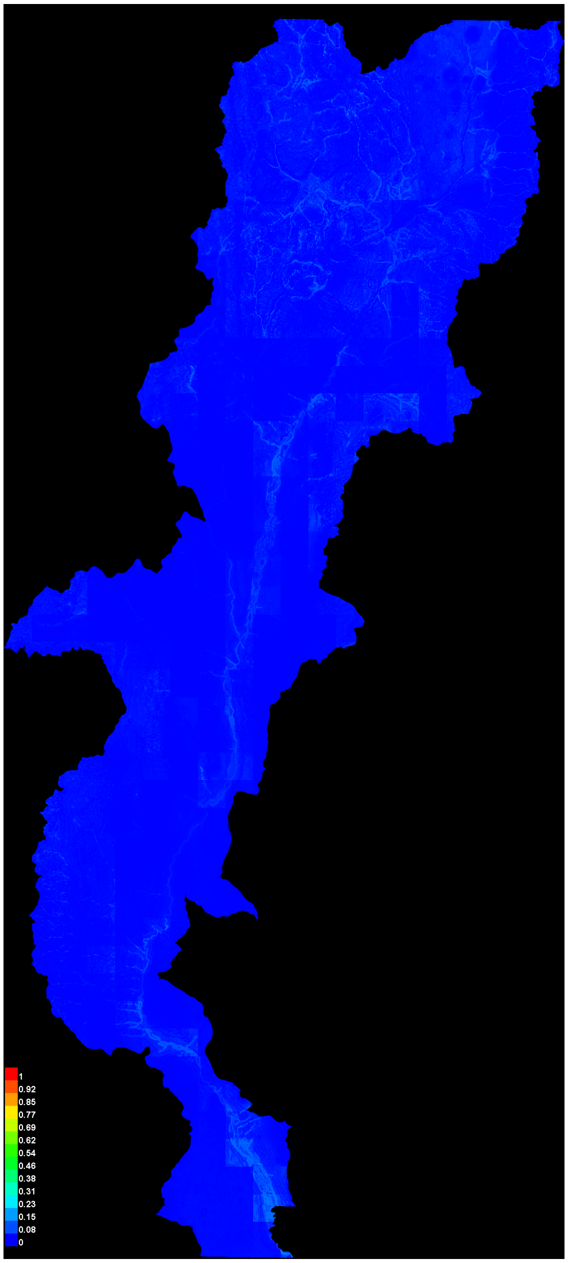

The following two pictures show the point-wise mean and standard deviation of the 15 models applied to the environmental layers in Envir_2090_ascii. Other available summary grids are [min](), [max]() and [median]().

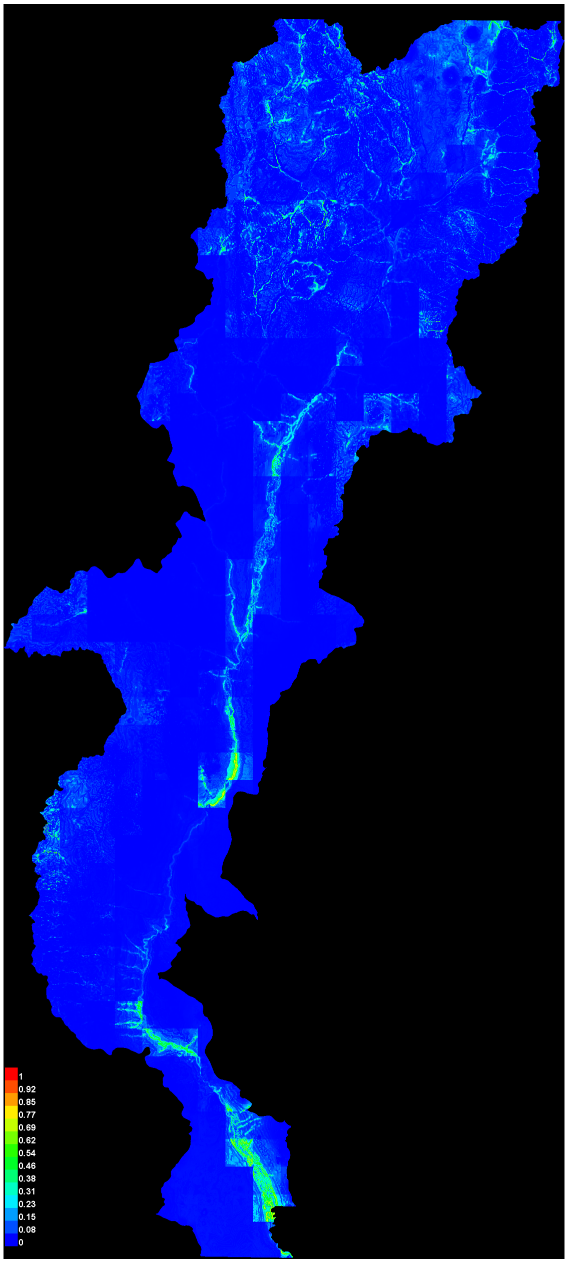

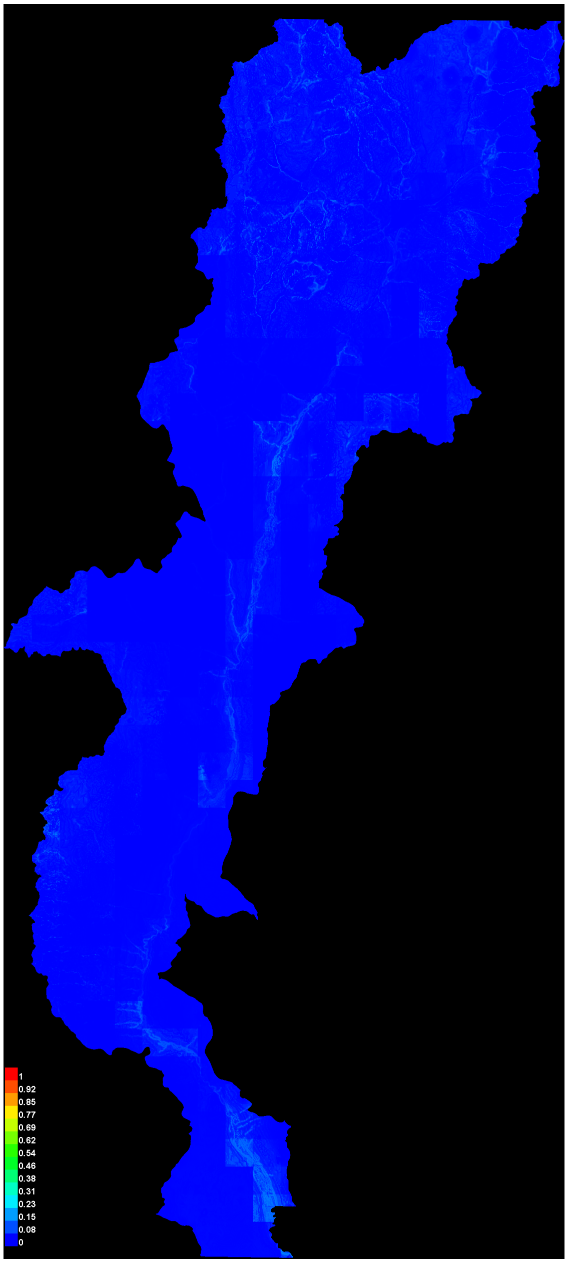

Response curves

These curves show how each environmental variable affects the Maxent prediction. The curves show how the logistic prediction changes as each environmental variable is varied, keeping all other environmental variables at their average sample value. Click on a response curve to see a larger version. Note that the curves can be hard to interpret if you have strongly correlated variables, as the model may depend on the correlations in ways that are not evident in the curves. In other words, the curves show the marginal effect of changing exactly one variable, whereas the model may take advantage of sets of variables changing together. The curves show the mean response of the 15 replicate Maxent runs (red) and and the mean +/- one standard deviation (blue, two shades for categorical variables).

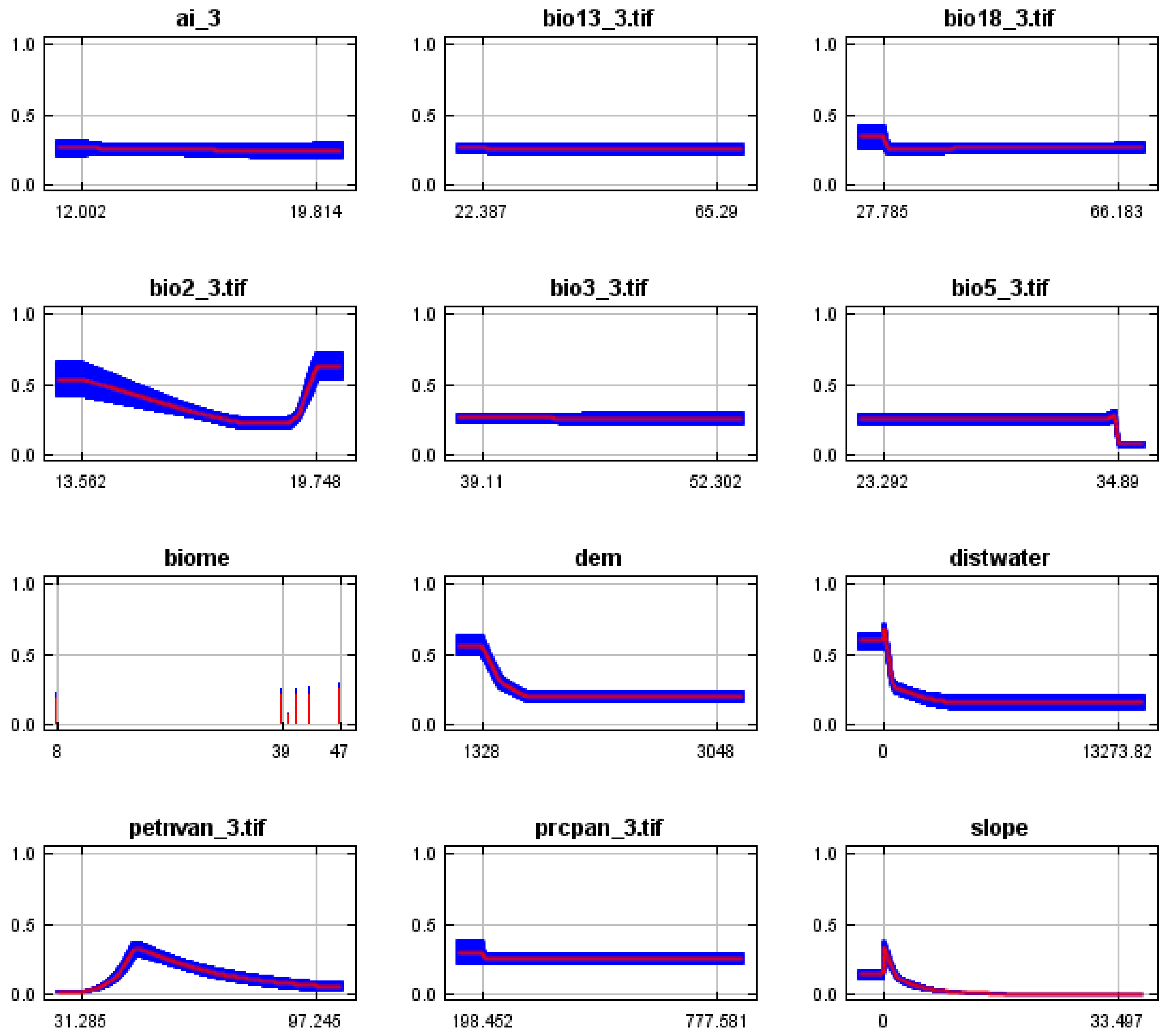

In contrast to the above marginal response curves, each of the following curves represents a different model, namely, a Maxent model created using only the corresponding variable. These plots reflect the dependence of predicted suitability both on the selected variable and on dependencies induced by correlations between the selected variable and other variables. They may be easier to interpret if there are strong correlations between variables.

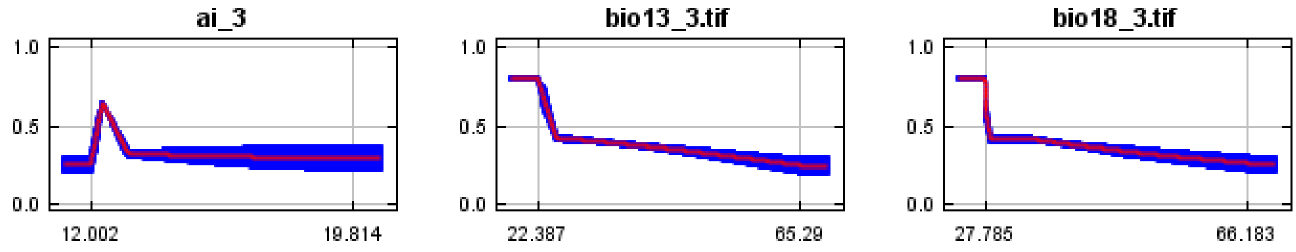

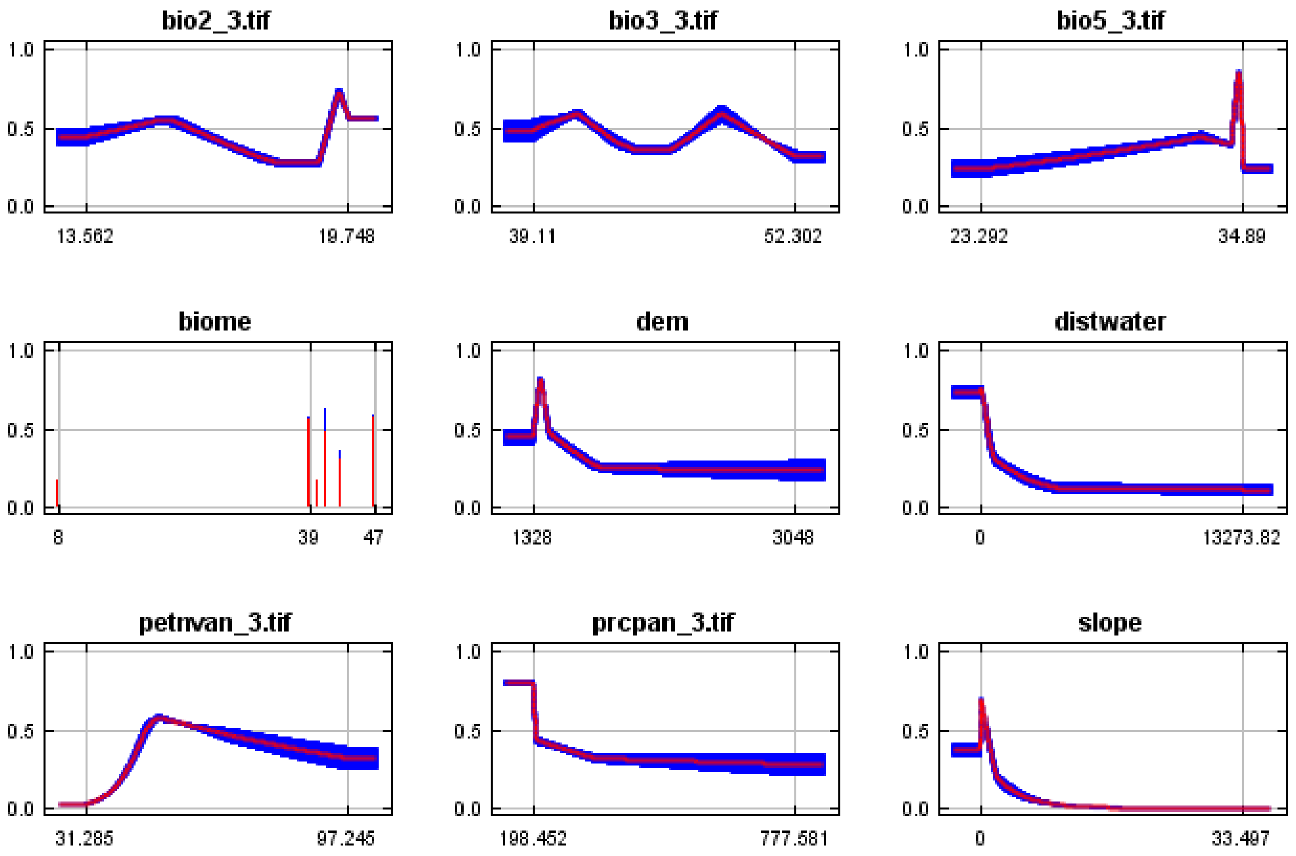

Analysis of variable contributions

The following table gives estimates of relative contributions of the environmental variables to the Maxent model. To determine the first estimate, in each iteration of the training algorithm, the increase in regularized gain is added to the contribution of the corresponding variable, or subtracted from it if the change to the absolute value of lambda is negative. For the second estimate, for each environmental variable in turn, the values of that variable on training presence and background data are randomly permuted. The model is reevaluated on the permuted data, and the resulting drop in training AUC is shown in the table, normalized to percentages. As with the variable jackknife, variable contributions should be interpreted with caution when the predictor variables are correlated. Values shown are averages over replicate runs.

Variable	Percent contribution	Permutation importance
distwater	41.8	26.3
slope	15.1	26
biome	11.1	9.7
bio2_3.tif	9.6	10.4
petnvan_3.tif	5.5	12.8
bio13_3.tif	4.6	0
bio18_3.tif	4	0.7
bio5_3.tif	3.1	7.9

prcpan_3.tif	2.8	0.1
dem	2	5.5
ai_3	0.1	0.4
bio3_3.tif	0	0.2

The following picture shows the results of the jackknife test of variable importance. The environmental variable with highest gain when used in isolation is distwater, which therefore appears to have the most useful information by itself. The environmental variable that decreases the gain the most when it is omitted is distwater, which therefore appears to have the most information that isn't present in the other variables. Values shown are averages over replicate runs.

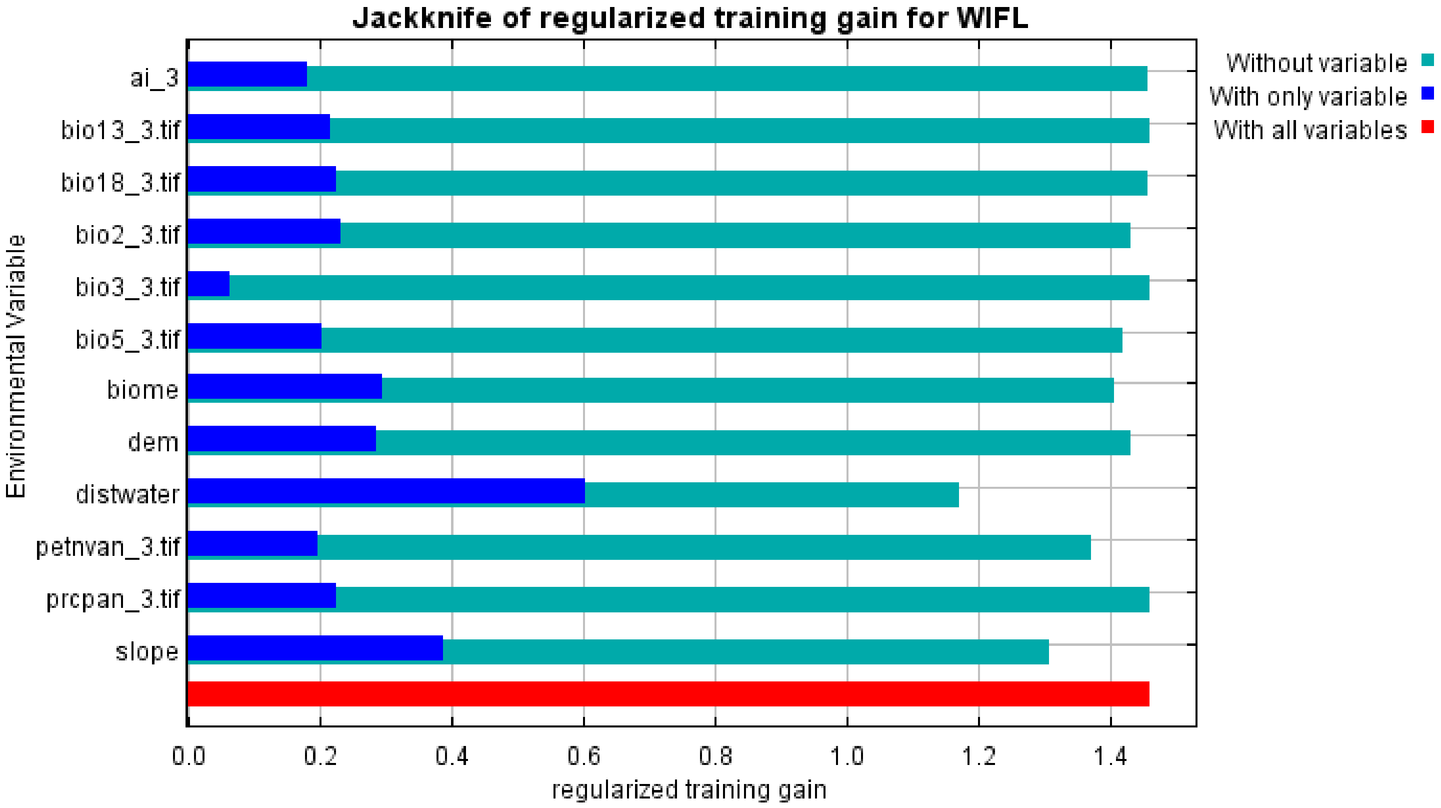

The next picture shows the same jackknife test, using test gain instead of training gain. Note that conclusions about which variables are most important can change, now that we're looking at test data.

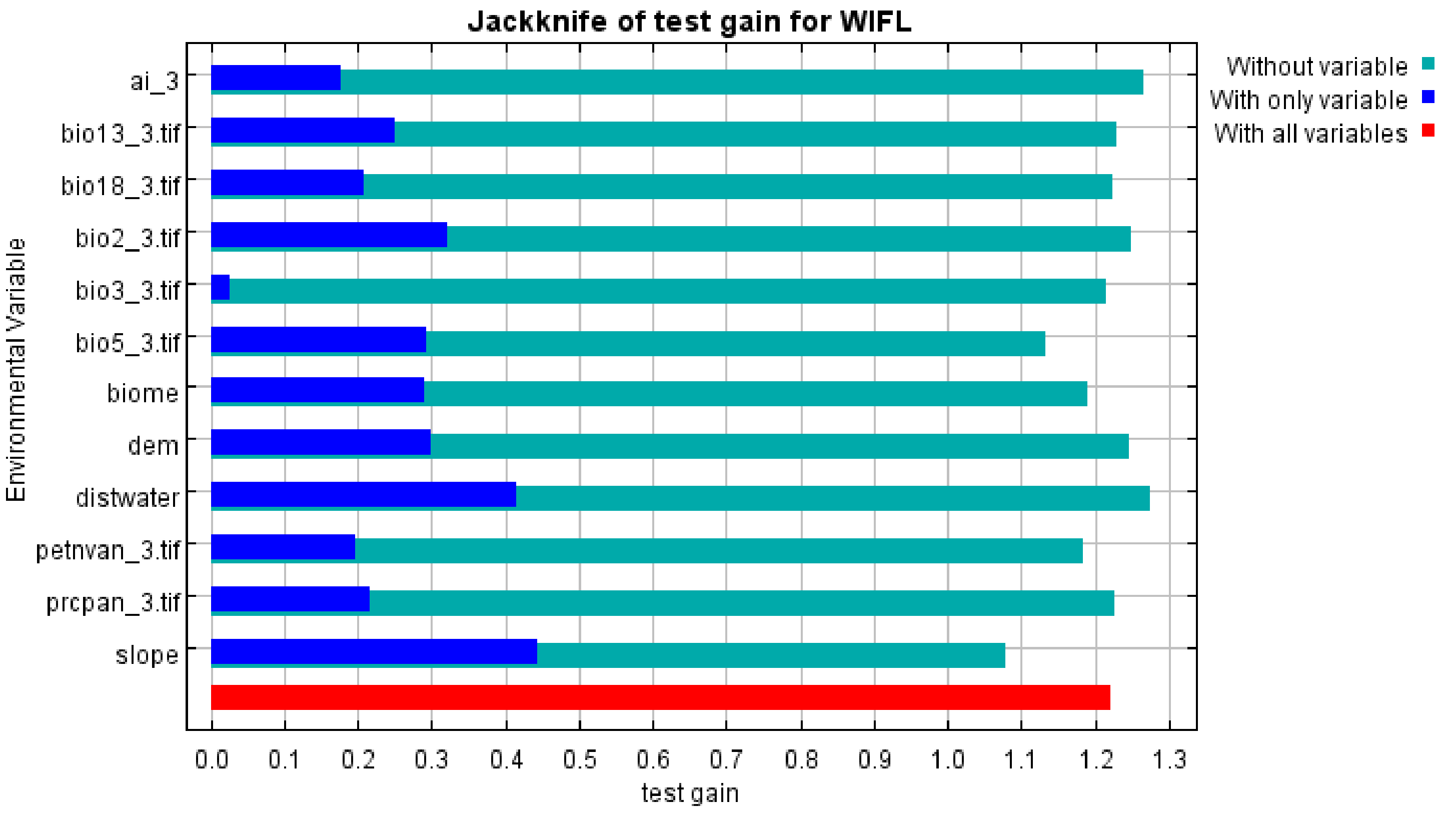

Lastly, we have the same jackknife test, using AUC on test data.

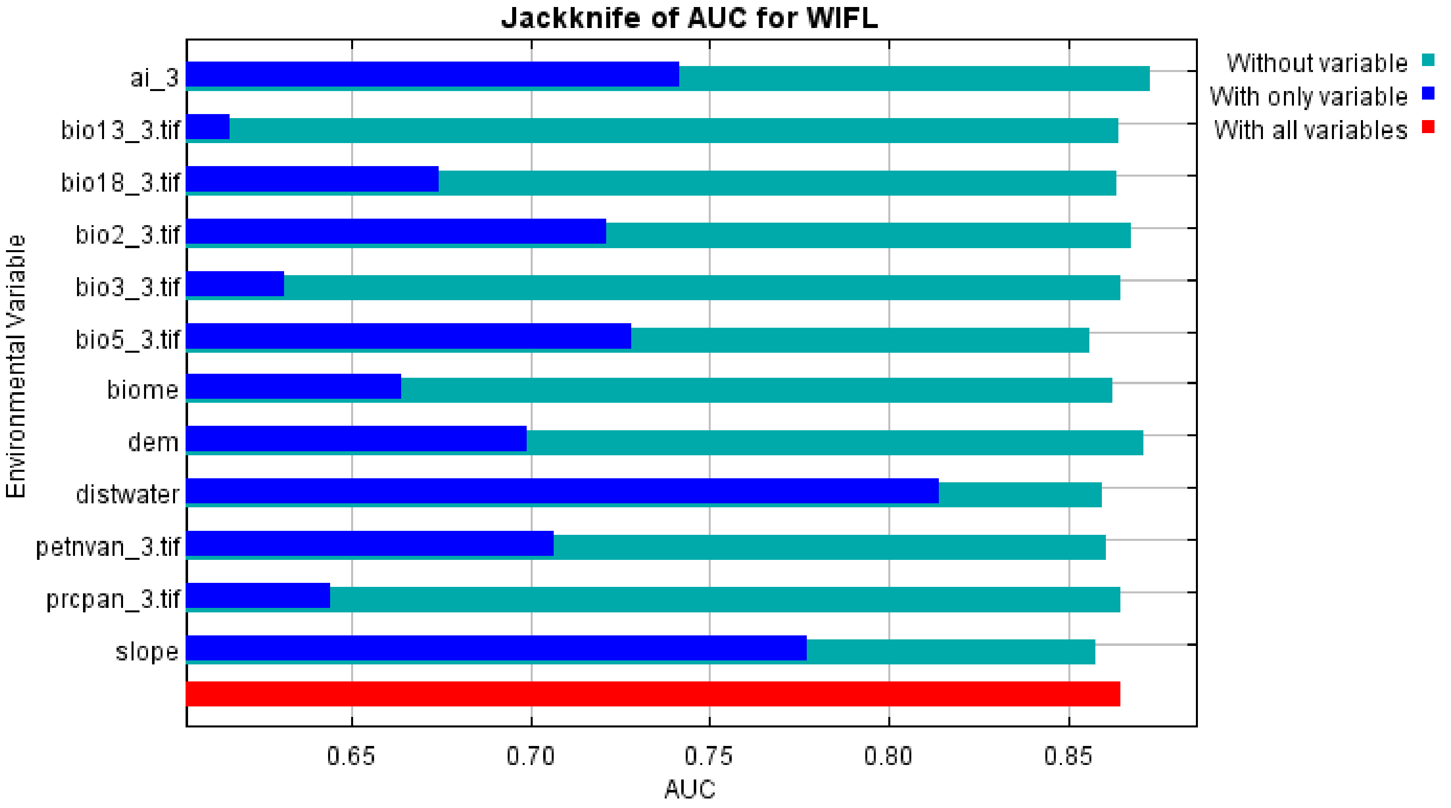

Command line to repeat this species model: java density.MaxEnt nowarnings noprefixes -E "" -E WIFL responsecurves jackknife outputdirectory=D:\MaxEnt4\BirdsHad

"projectionlayers=D:\Ascii_Files\Envir_2030_ascii, D:\Ascii_Files\Envir_2060_ascii, D:\Ascii_Files\Envir_2090_ascii" samplesfile=D:\Data\RioGrande\Birds\allbirds.csv environmentallayers=D:\Ascii_Files\Envir_Curr_ascii randomseed noaskoverwrite biasfile=D:\Data\RioGrande\Birds\all_bias.asc replicates=15 nooutputgrids maximumiterations=5000 biastype=3 "applythresholdrule=equal training sensitivity and specificity" -N ai_1 -N ai_2 -N bio10_1 -N bio10_1.tif -N bio10_2 -N bio10_2.tif -N bio10_3 -N bio10_3.tif -N bio11_1 -N bio11_1.tif -N bio11_2 -N bio11_2.tif -N bio11_3 -N bio11_3.tif -N bio13_1.tif -N bio13_2.tif -N bio14_1.tif -N bio14_2.tif -N bio14_3.tif -N bio16_1.tif -N bio16_2.tif -N bio16_3.tif -N bio17_.tif -N bio17_2.tif -N bio17_3.tif -N bio17_all.tif -N bio18_1.tif -N bio18_2.tif -N bio19_1.tif -N bio19_2.tif -N bio19_3.tif -N bio2_1.tif -N bio2_2.tif -N bio3_1.tif -N bio3_2.tif -N bio5_1.tif -N bio5_2.tif -N bio6_1.tif -N bio6_2.tif -N bio6_3.tif -N bio7_1.tif -N bio7_2.tif -N bio7_3.tif -N etan_1.tif -N etan_2.tif -N etan_3.tif -N etdjf_1.tif -N etdjf_2.tif -N etdjf_3.tif -N etjas_1.tif -N etjas_2.tif -N etjas_3.tif -N etjfm_1.tif -N etjfm_2.tif -N etjfm_3.tif -N etjja_1.tif -N etjja_2.tif -N etjja_3.tif -N etjja_all.tif -N petnvan_1.tif -N petnvan_2.tif -N petnvdjf_1.tif -N petnvdjf_2.tif -N petnvdjf_3.tif -N petnvjas_1.tif -N petnvjas_2.tif -N petnvjas_3.tif -N petnvjfm_1.tif -N petnvjfm_2.tif -N petnvjfm_3.tif -N petnvjja_1.tif -N petnvjja_2.tif -N petnvjja_3.tif -N petwan_1.tif -N petwan_2.tif -N petwan_3.tif -N petwdjf_1.tif -N petwdjf_2.tif -N petwdjf_3.tif -N petwjas_1.tif -N petwjas_2.tif -N petwjas_3.tif -N petwjas_all.tif -N petwjfm_1.tif -N petwjfm_2.tif -N petwjfm_3.tif -N petwjja_1.tif -N petwjja_2.tif -N petwjja_3.tif -N prcpan_1.tif -N prcpan_2.tif -N prcpan_all.tif -N runoffan_1.tif -N runoffan_2.tif -N runoffan_3.tif -N runoffdjf_1.tif -N runoffdjf_2.tif -N runoffdjf_3.tif -N runoffjas_1.tif -N runoffjas_2.tif -N runoffjas_3.tif -N runoffjas_all.tif -N runoffjfm_1.tif -N runoffjfm_2.tif -N runoffjfm_3.tif -N runoffjja_1.tif -N runoffjja_2.tif -N runoffjja_3.tif -N smcan_1.tif -N smcan_2.tif -N smcan_3.tif -N smcdjf_1.tif -N smcdjf_2.tif -N smcdjf_3.tif -N smcjas_1.tif -N smcjas_2.tif -N smcjas_3.tif -N smcjfm_1.tif -N smcjfm_2.tif -N smcjfm_3.tif -N smcjja_1.tif -N smcjja_2.tif -N smcjja_3.tif -N swean_1.tif -N swean_2.tif -N swean_3.tif -N swedjf_1.tif -N swedjf_2.tif -N swedjf_3.tif -N swejfm_1.tif -N swejfm_2.tif -N swejfm_3.tif -N tave_1.tif -N tave_2.tif -N tave_3.tif -N tmax_1.tif -N tmax_2.tif -N tmax_3.tif -N tmin_1.tif -N tmin_2.tif -N tmin_3.tif -t biome

Replicated maxent model for YBCU

This page summarizes the results of 15-fold cross-validation for YBCU, created Tue Aug 05 15:17:41 MDT 2014 using Maxent version 3.3.3k. The individual models are here: [0] [1] [2] [3] [4] [5] [6] [7] [8] [9] [10] [11] [12] [13] [14]

Analysis of omission/commission

The following picture shows the test omission rate and predicted area as a function of the cumulative threshold, averaged over the replicate runs. The omission rate should be close to the predicted omission, because of the definition of the cumulative threshold.

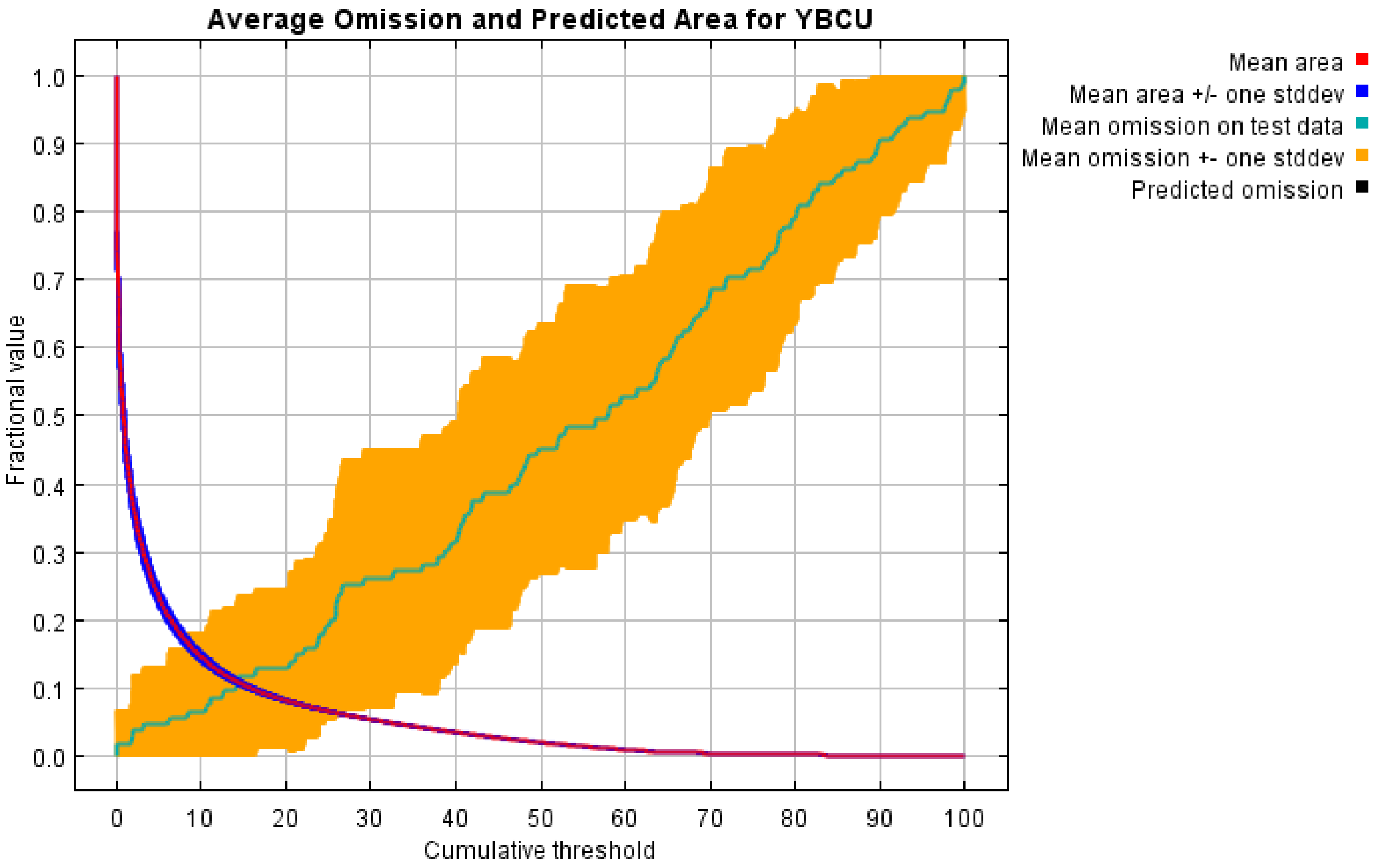

The next picture is the receiver operating characteristic (ROC) curve for the same data, again averaged over the replicate runs. Note that the specificity is defined using predicted area, rather than true commission (see the paper by Phillips, Anderson and Schapire cited on the help page for discussion of what this means). The average test AUC for the replicate runs is 0.947, and the standard deviation is 0.049.

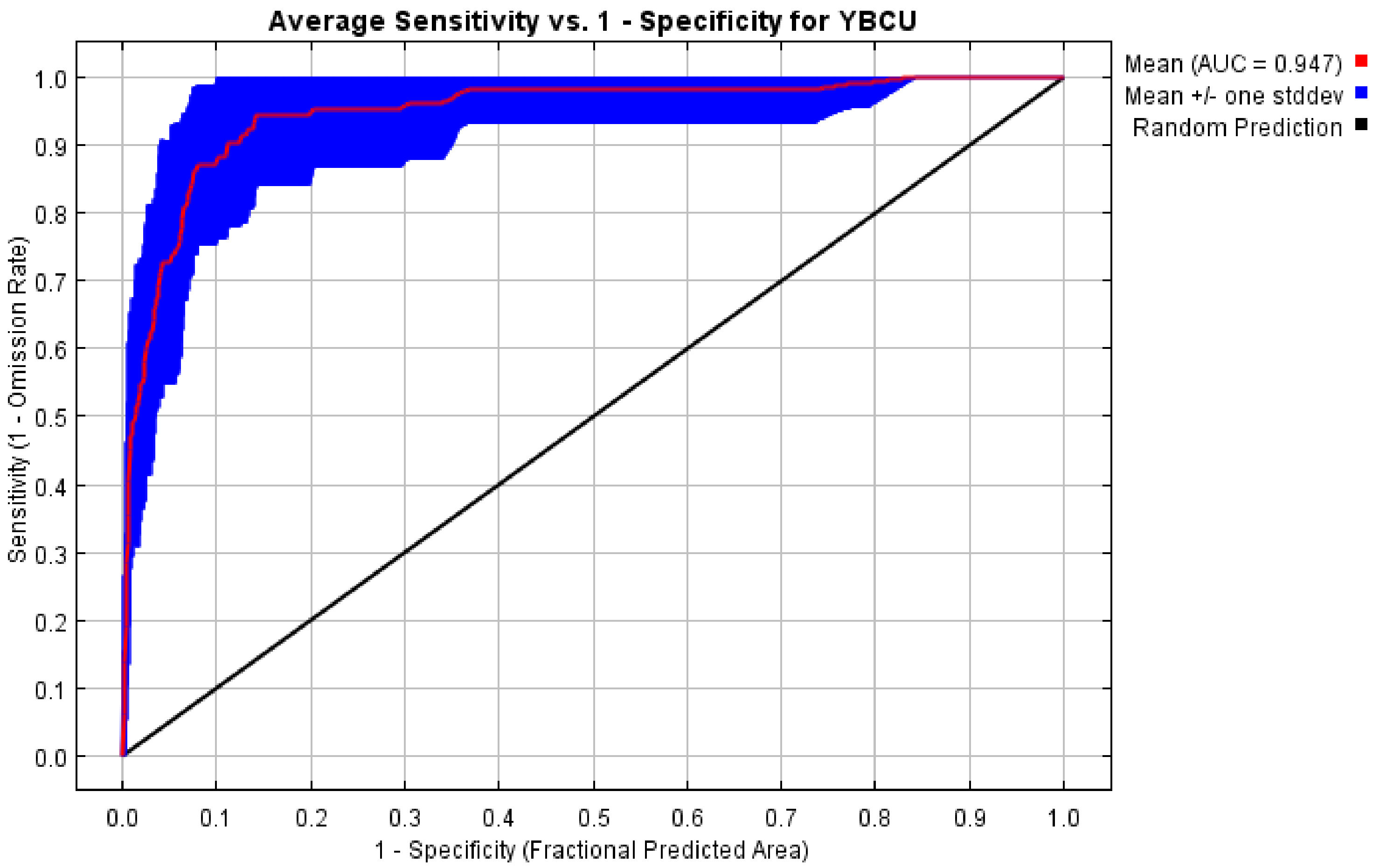

Pictures of the model

The following two pictures show the point-wise mean and standard deviation of the 15 output grids. Other available summary grids are min, max and median.

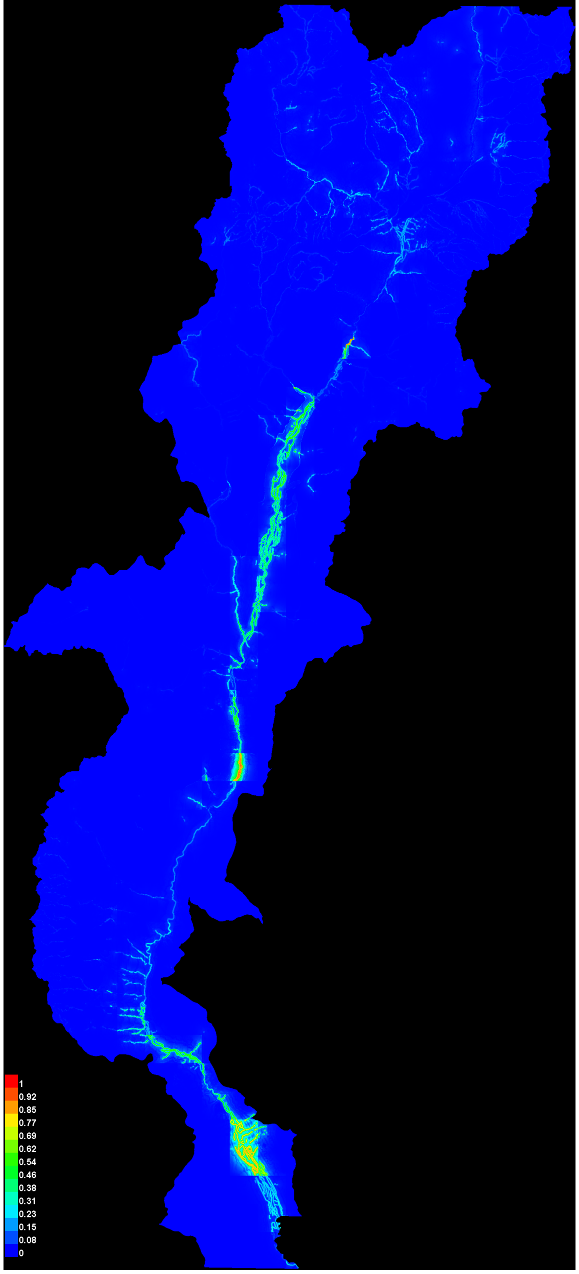

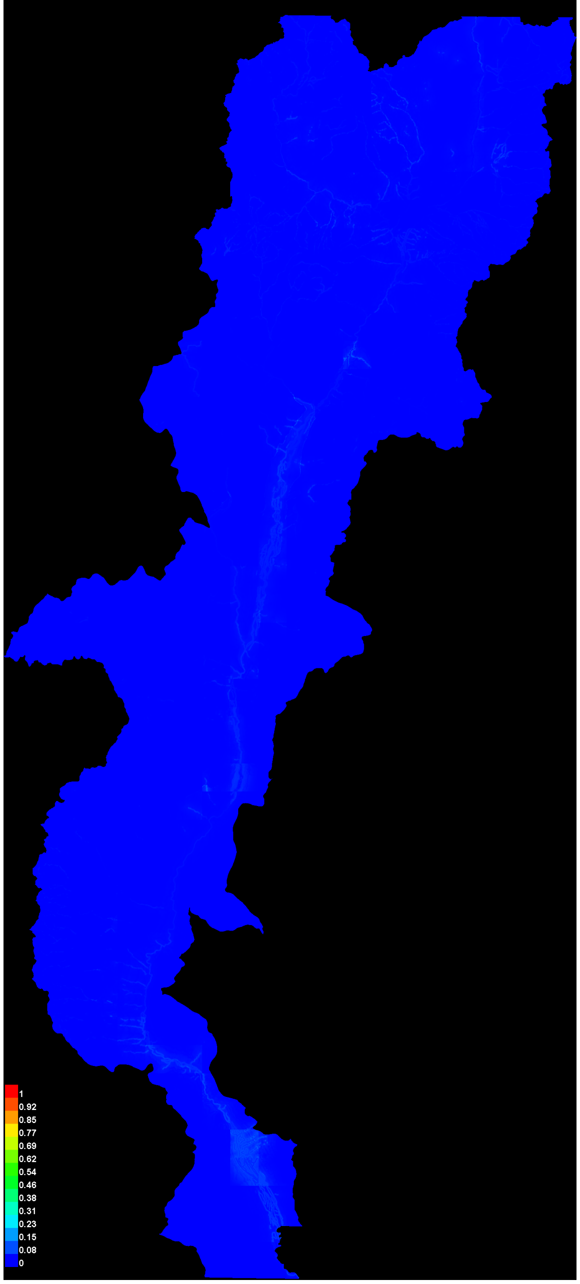

The following two pictures show the point-wise mean and standard deviation of the 15 models applied to the environmental layers in Envir_2030_ascii. Other available summary grids are min, max and median.

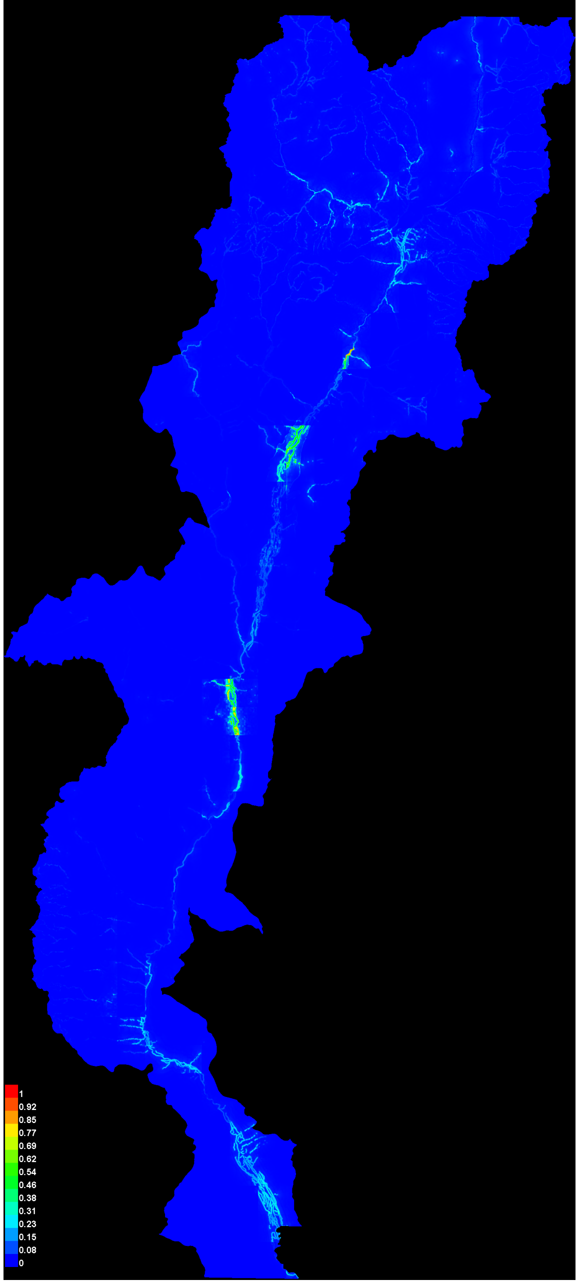

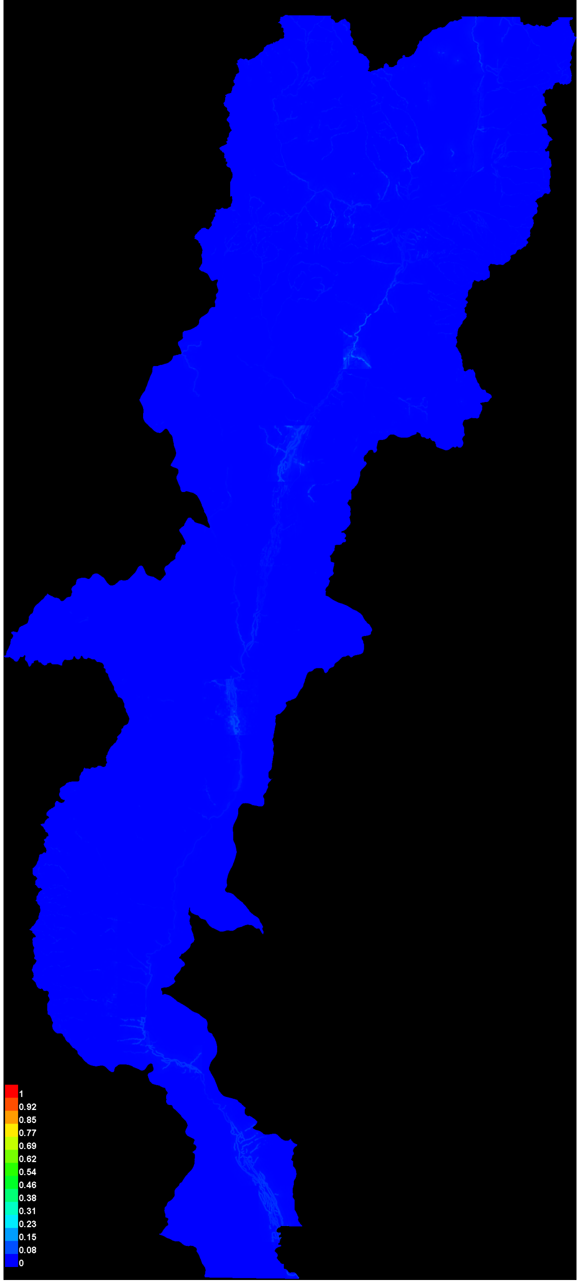

The following two pictures show the point-wise mean and standard deviation of the 15 models applied to the environmental layers in Envir_2060_ascii. Other available summary grids are min, max and median.

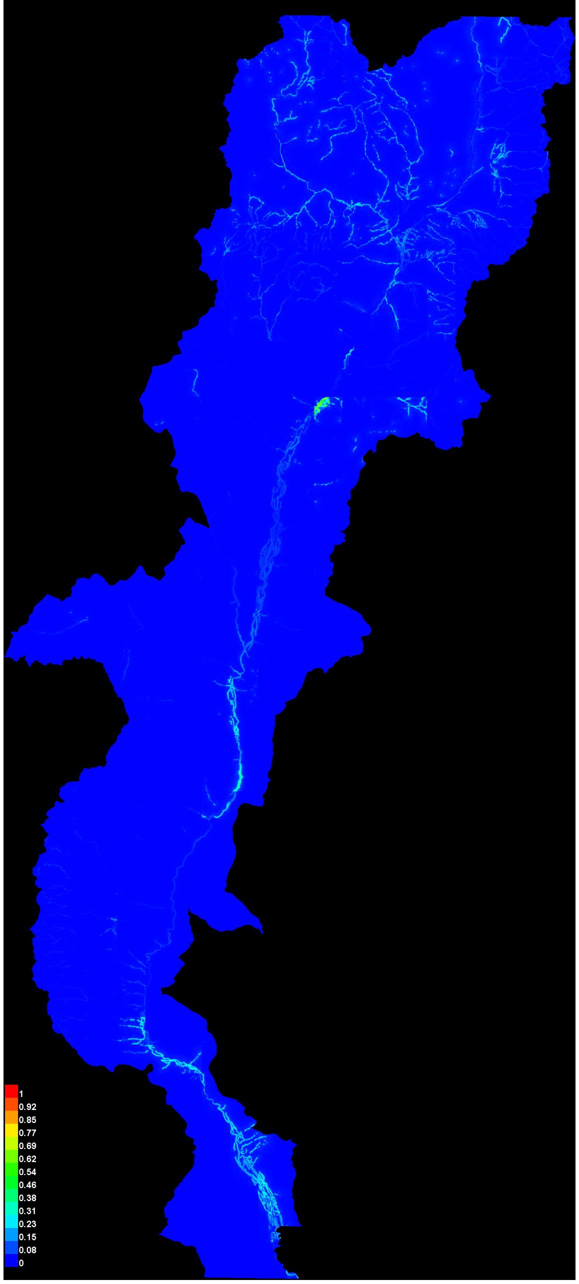

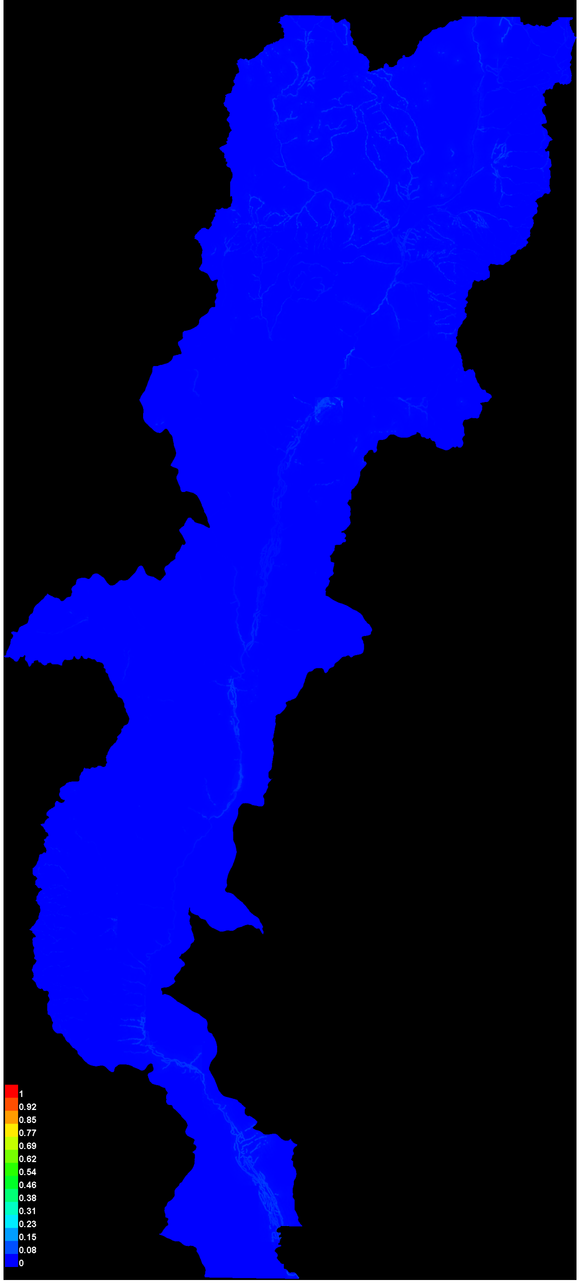

The following two pictures show the point-wise mean and standard deviation of the 15 models applied to the environmental layers in Envir_2090_ascii. Other available summary grids are min, max and median.

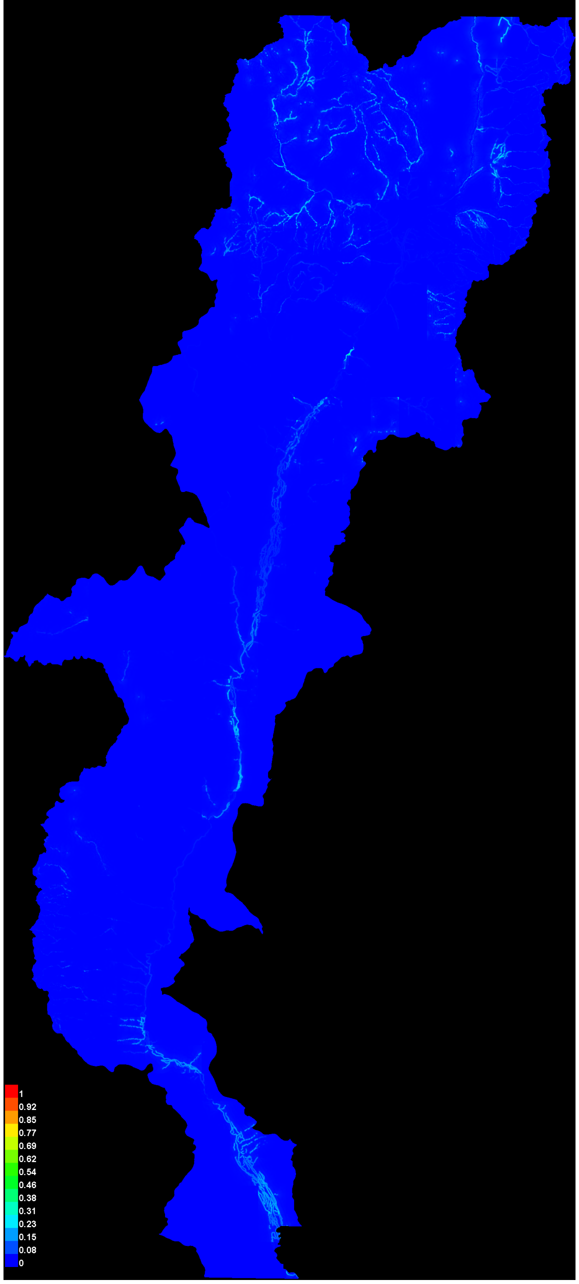

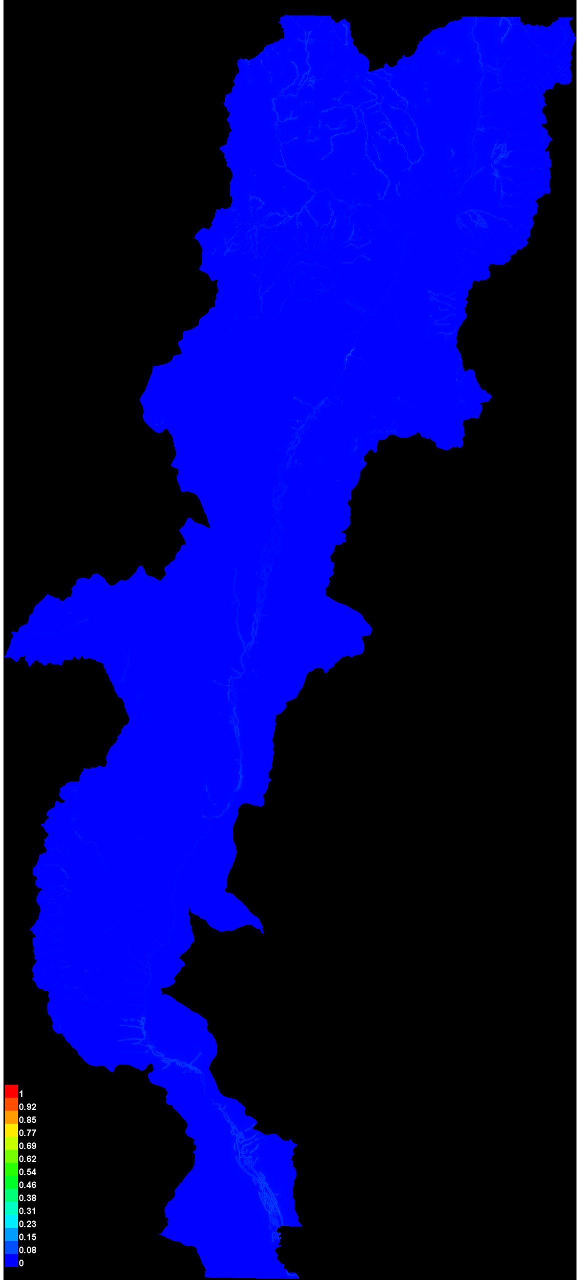

Response curves

These curves show how each environmental variable affects the Maxent prediction. The curves show how the logistic prediction changes as each environmental variable is varied, keeping all other environmental variables at their average sample value. Click on a response curve to see a larger version. Note that the curves can be hard to interpret if you have strongly correlated variables, as the model may depend on the correlations in ways that are not evident in the curves. In other words, the curves show the marginal effect of changing exactly one variable, whereas the model may take advantage of sets of variables changing together. The curves show the mean response of the 15 replicate Maxent runs (red) and and the mean +/- one standard deviation (blue, two shades for categorical variables).

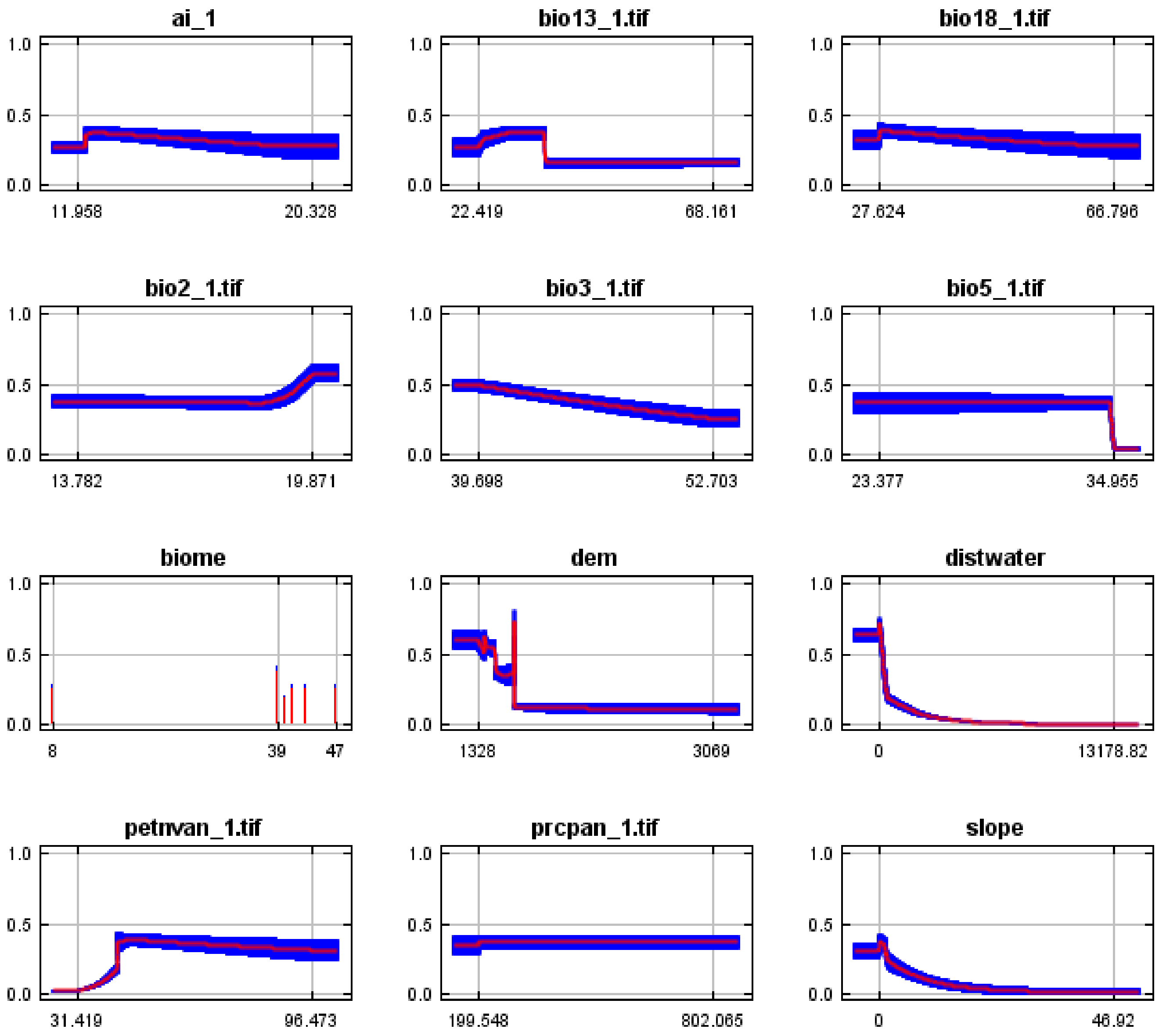

In contrast to the above marginal response curves, each of the following curves represents a different model, namely, a Maxent model created using only the corresponding variable. These plots reflect the dependence of predicted suitability both on the selected variable and on dependencies induced by correlations between the selected variable and other variables. They may be easier to interpret if there are strong correlations between variables.

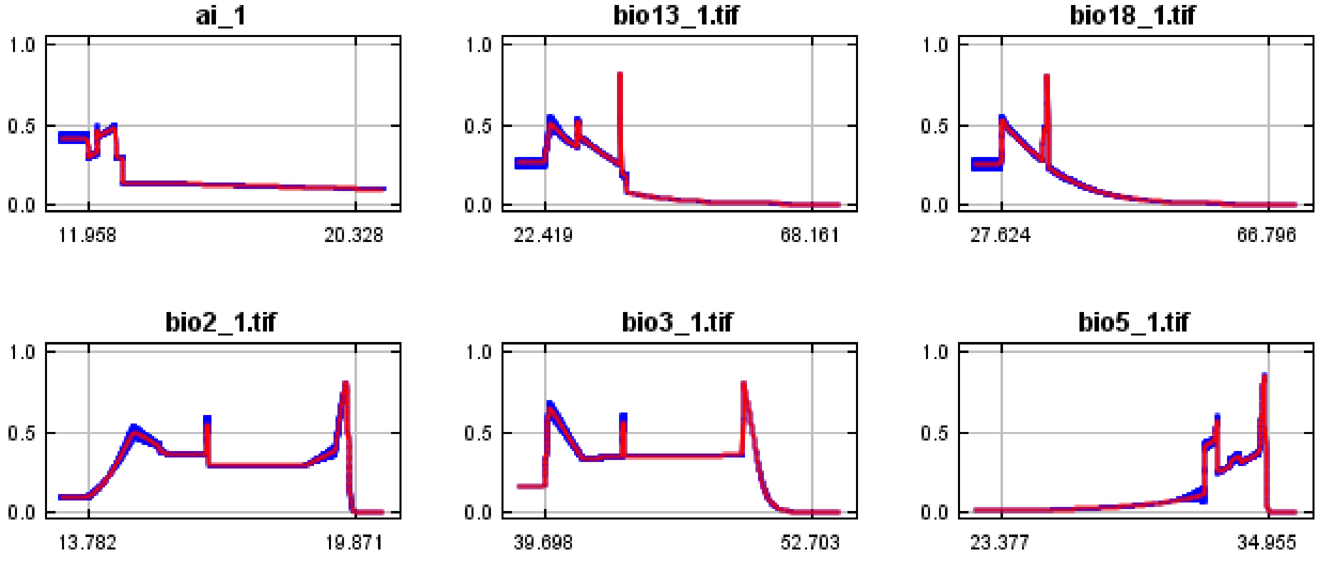

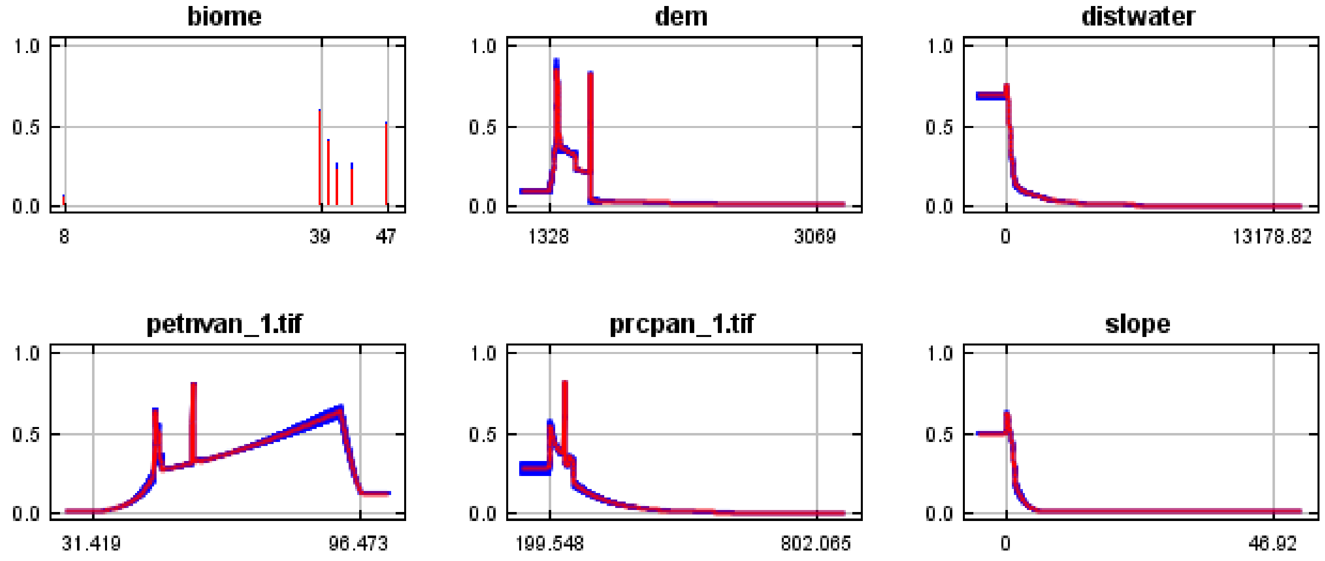

Analysis of variable contributions

The following table gives estimates of relative contributions of the environmental variables to the Maxent model. To determine the first estimate, in each iteration of the training algorithm, the increase in regularized gain is added to the contribution of the corresponding variable, or subtracted from it if the change to the absolute value of lambda is negative. For the second estimate, for each environmental variable in turn, the values of that variable on training presence and background data are randomly permuted. The model is reevaluated on the permuted data, and the resulting drop in training AUC is shown in the table, normalized to percentages. As with the variable jackknife, variable contributions should be interpreted with caution when the predictor variables are correlated. Values shown are averages over replicate runs.

Variable	Percent contribution	Permutation importance
distwater	56.9	58.4
dem	20.2	17.5
bio2_1.tif	5.3	0.4
petnvan_1.tif	4.6	7.6
slope	3.4	4.4
bio5_1.tif	2.3	2.9
bio13_1.tif	2.1	3.2
bio18_1.tif	1.6	0.8
biome	1.6	2.2
ai_1	1.5	1
bio3_1.tif	0.4	1.4
prcpan_1.tif	0.2	0.1

The following picture shows the results of the jackknife test of variable importance. The environmental variable with highest gain when used in isolation is distwater, which therefore appears to have the most useful information by itself. The environmental variable that decreases the gain the most when it is omitted is distwater, which therefore appears to have the most information that isn't present in the other variables. Values shown are averages over replicate runs.

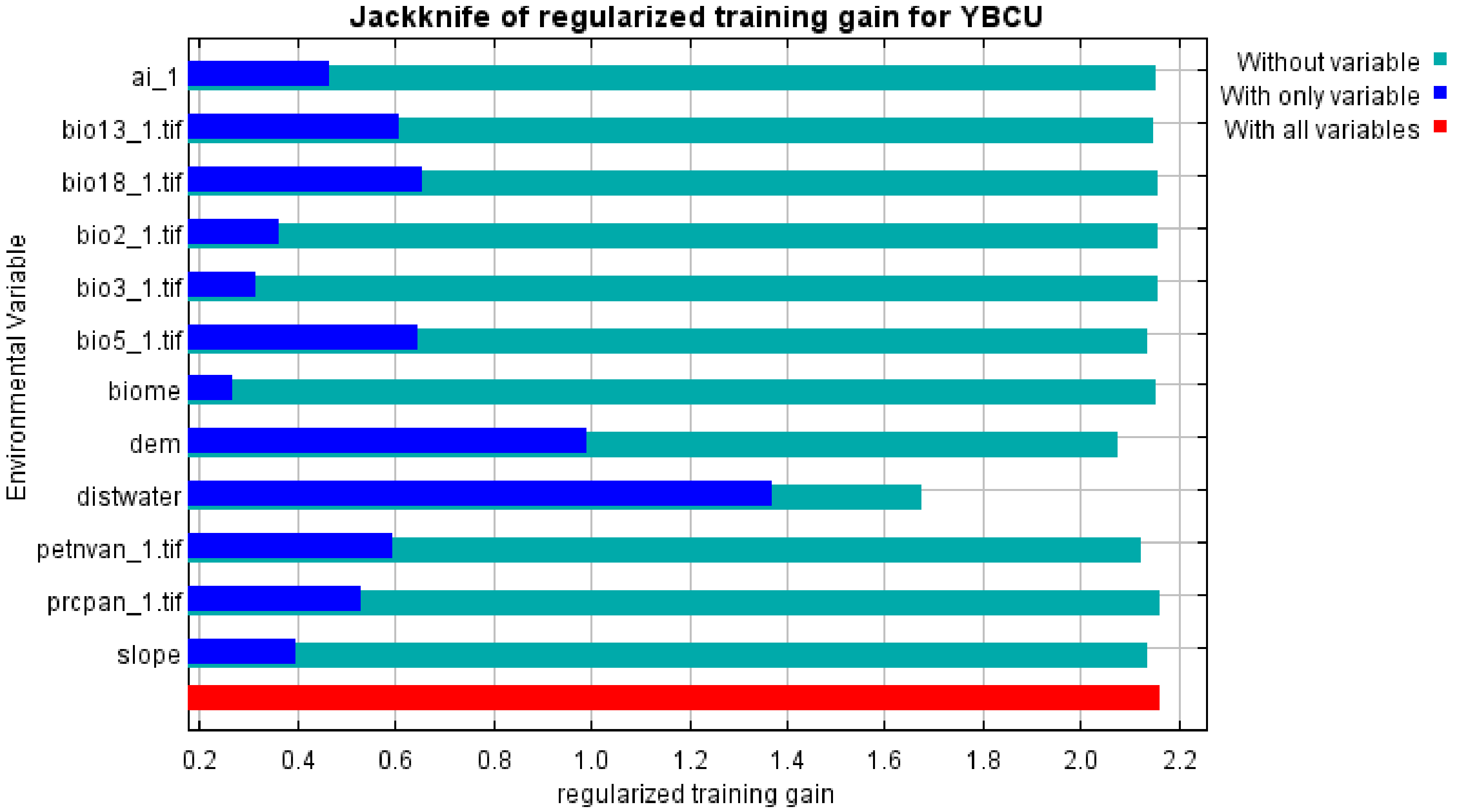

The next picture shows the same jackknife test, using test gain instead of training gain. Note that conclusions about which variables are most important can change, now that we're looking at test data.

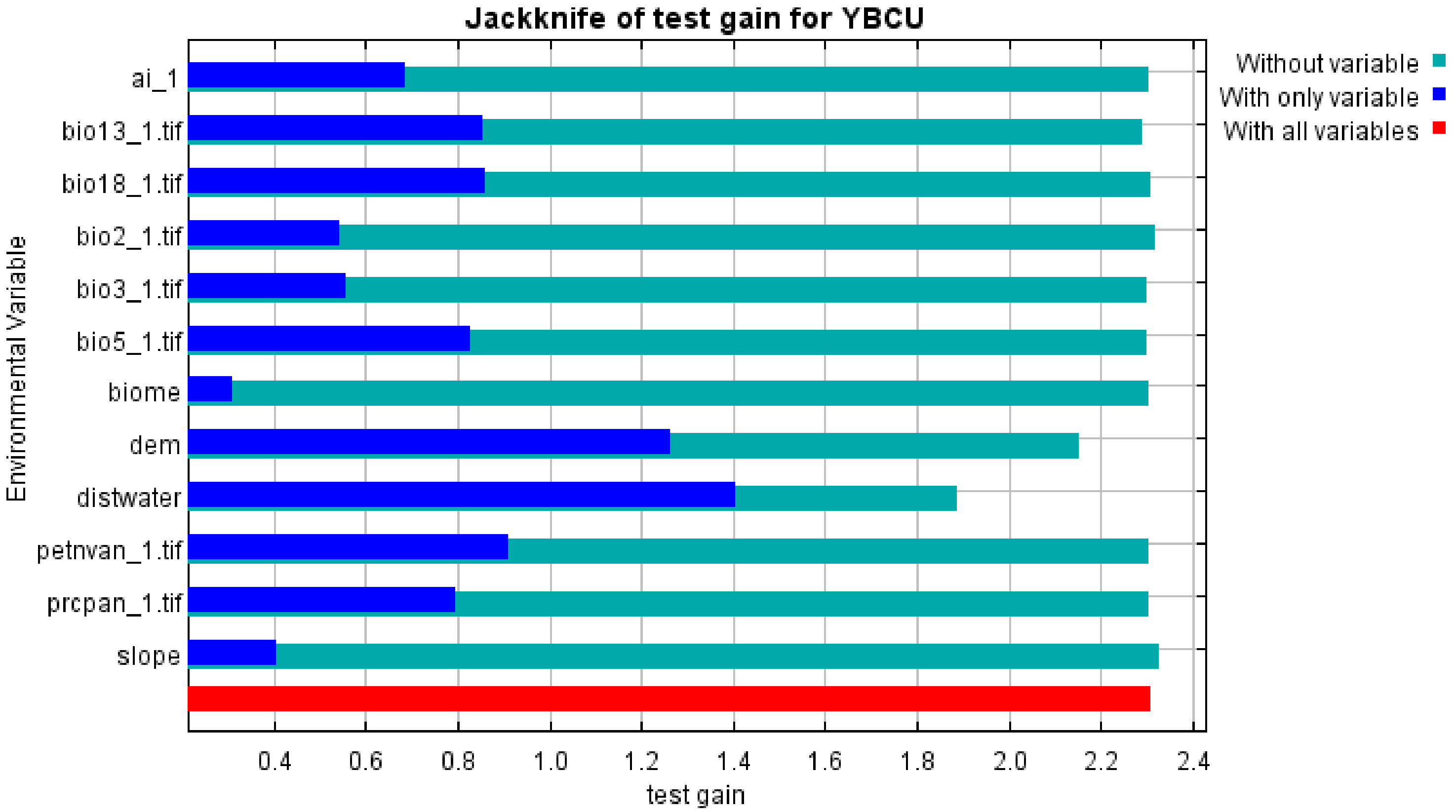

Lastly, we have the same jackknife test, using AUC on test data.

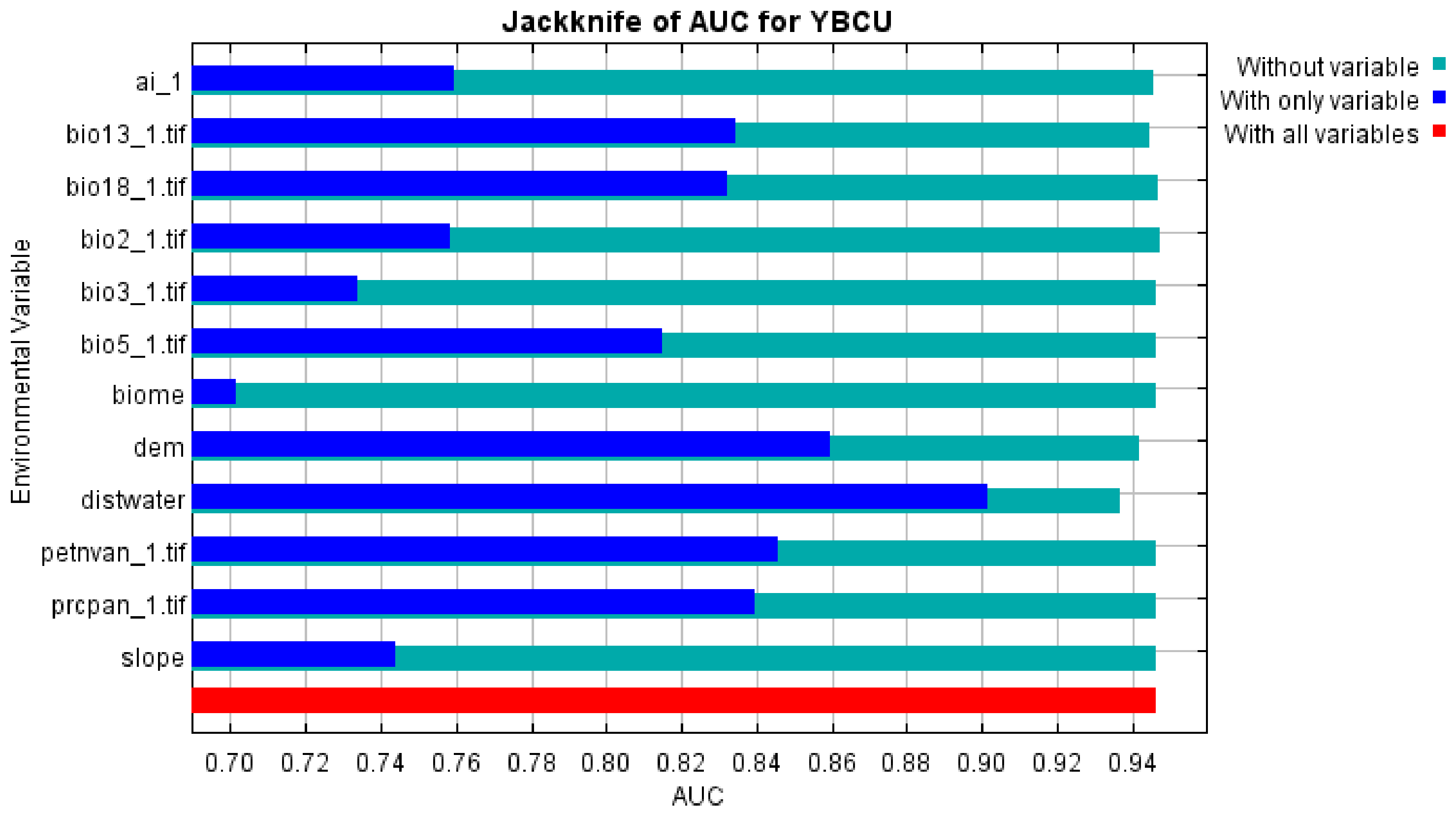

Command line to repeat this species model: java density.MaxEnt nowarnings noprefixes -E "" -E YBCU responsecurves jackknife outputdirectory=D:\MaxEnt4\BirdsCCC "projectionlayers=D:\Ascii_Files\Envir_2030_ascii, D:\Ascii_Files\Envir_2060_ascii, D:\Ascii_Files\Envir_2090_ascii" samplesfile=D:\Data\RioGrande\Birds\allbirds.csv environmentallayers=D:\Ascii_Files\Envir_Curr_ascii randomseed noaskoverwrite biasfile=D:\Data\RioGrande\Birds\all_bias.asc replicates=15 nooutputgrids maximumiterations=5000 biastype=3 "applythresholdrule=equal training sensitivity and specificity" -N ai_2 -N ai_3 -N bio10_1 -N bio10_1.tif -N bio10_2 -N bio10_2.tif -N bio10_3 -N bio10_3.tif -N bio11_1 -N bio11_1.tif -N bio11_2 -N bio11_2.tif -N bio11_3 -N bio11_3.tif -N bio13_2.tif -N bio13_3.tif -N bio14_1.tif -N bio14_2.tif -N bio14_3.tif -N bio16_1.tif -N bio16_2.tif -N bio16_3.tif -N bio17_.tif -N bio17_2.tif -N bio17_3.tif -N bio17_all.tif -N bio18_2.tif -N bio18_3.tif -N bio19_1.tif -N bio19_2.tif -N bio19_3.tif -N bio2_2.tif -N bio2_3.tif -N bio3_2.tif -N bio3_3.tif -N bio5_2.tif -N bio5_3.tif -N bio6_1.tif -N bio6_2.tif -N bio6_3.tif -N bio7_1.tif -N bio7_2.tif -N bio7_3.tif -N etan_1.tif -N etan_2.tif -N etan_3.tif -N etdjf_1.tif -N etdjf_2.tif -N etdjf_3.tif -N etjas_1.tif -N etjas_2.tif -N etjas_3.tif -N etjfm_1.tif -N etjfm_2.tif -N etjfm_3.tif -N etjja_1.tif -N etjja_2.tif -N etjja_3.tif -N etjja_all.tif -N petnvan_2.tif -N petnvan_3.tif -N petnvdjf_1.tif -N petnvdjf_2.tif -N petnvdjf_3.tif -N petnvjas_1.tif -N petnvjas_2.tif -N petnvjas_3.tif -N petnvjfm_1.tif -N petnvjfm_2.tif -N petnvjfm_3.tif -N petnvjja_1.tif -N petnvjja_2.tif -N petnvjja_3.tif -N petwan_1.tif -N petwan_2.tif -N petwan_3.tif -N petwdjf_1.tif -N petwdjf_2.tif -N petwdjf_3.tif -N petwjas_1.tif -N petwjas_2.tif -N petwjas_3.tif -N petwjas_all.tif -N petwjfm_1.tif -N petwjfm_2.tif -N petwjfm_3.tif -N petwjja_1.tif -N petwjja_2.tif -N petwjja_3.tif -N prcpan_2.tif -N prcpan_3.tif -N prcpan_all.tif -N runoffan_1.tif -N runoffan_2.tif -N runoffan_3.tif -N runoffdjf_1.tif -N runoffdjf_2.tif -N runoffdjf_3.tif -N runoffjas_1.tif -N runoffjas_2.tif -N runoffjas_3.tif -N runoffjas_all.tif -N runoffjfm_1.tif -N runoffjfm_2.tif -N runoffjfm_3.tif -N runoffjja_1.tif -N runoffjja_2.tif -N runoffjja_3.tif -N smcan_1.tif -N smcan_2.tif -N smcan_3.tif -N smcdjf_1.tif -N smcdjf_2.tif -N smcdjf_3.tif -N smcjas_1.tif -N smcjas_2.tif -N smcjas_3.tif -N smcjfm_1.tif -N smcjfm_2.tif -N smcjfm_3.tif -N smcjja_1.tif -N smcjja_2.tif -N smcjja_3.tif -N swean_1.tif -N swean_2.tif -N swean_3.tif -N swedjf_1.tif -N swedjf_2.tif -N swedjf_3.tif -N swejfm_1.tif -N swejfm_2.tif -N swejfm_3.tif -N tave_1.tif -N tave_2.tif -N tave_3.tif -N tmax_1.tif -N tmax_2.tif -N tmax_3.tif -N tmin_1.tif -N tmin_2.tif -N tmin_3.tif -t biome

Replicated maxent model for YBCU

This page summarizes the results of 15-fold cross-validation for YBCU, created Mon Aug 04 17:15:10 MDT 2014 using Maxent version 3.3.3k. The individual models are here: [0] [1] [2] [3] [4] [5] [6] [7] [8] [9] [10] [11] [12] [13] [14]

Analysis of omission/commission

The following picture shows the test omission rate and predicted area as a function of the cumulative threshold, averaged over the replicate runs. The omission rate should be close to the predicted omission, because of the definition of the cumulative threshold.

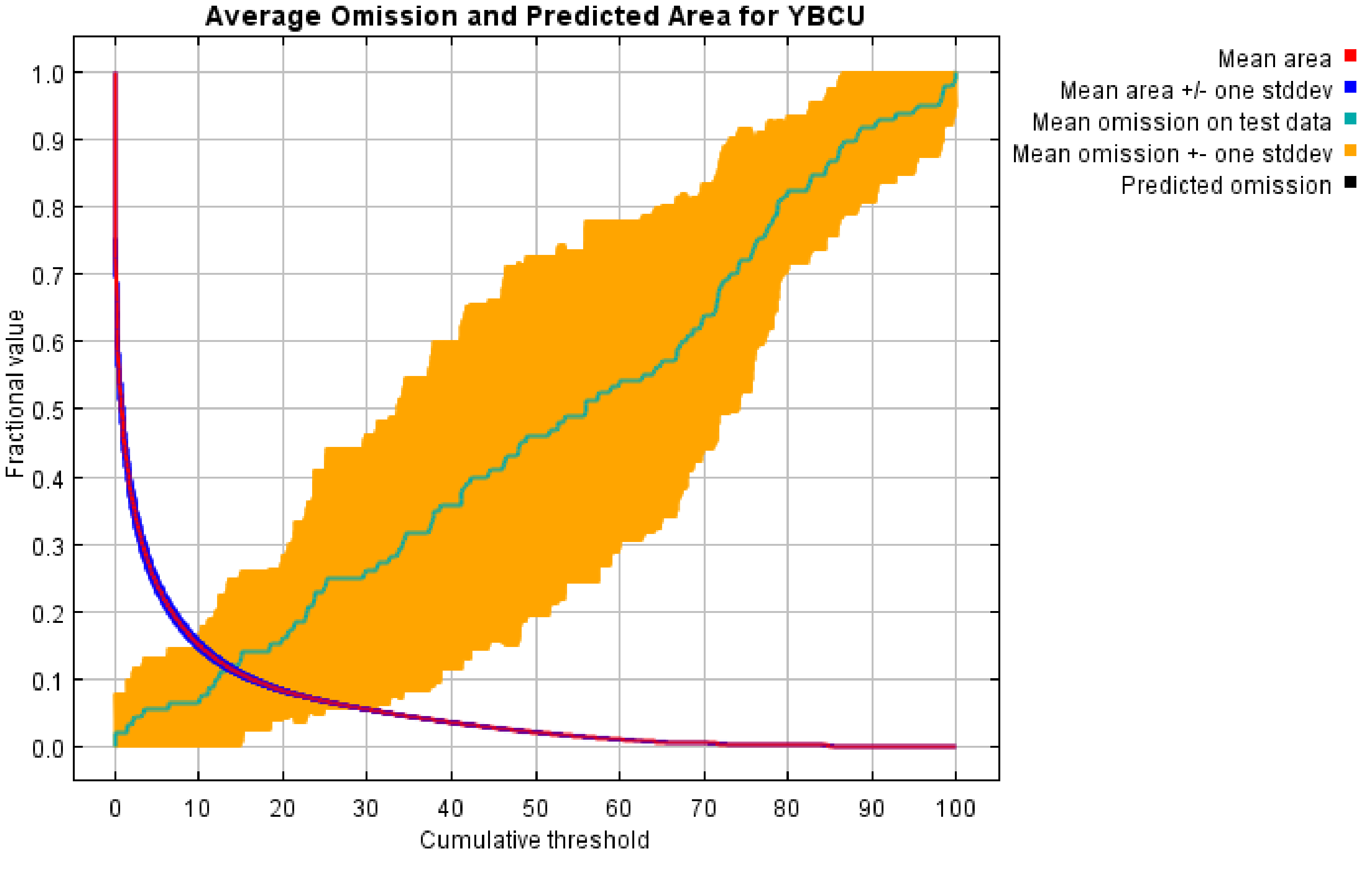

The next picture is the receiver operating characteristic (ROC) curve for the same data, again averaged over the replicate runs. Note that the specificity is defined using predicted area, rather than true commission (see the paper by Phillips, Anderson and Schapire cited on the help page for discussion of what this means). The average test AUC for the replicate runs is 0.940, and the standard deviation is 0.048.

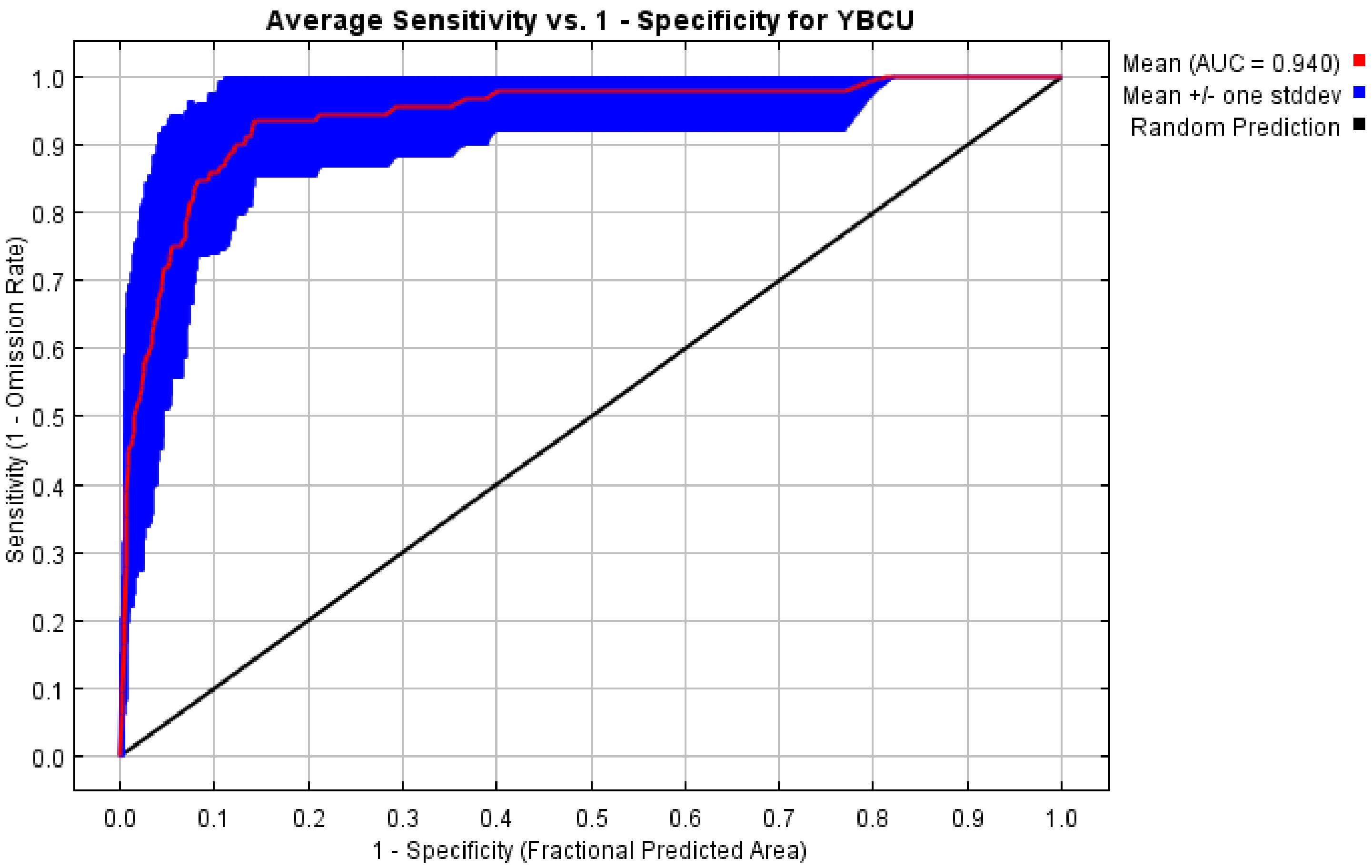

Pictures of the model

The following two pictures show the point-wise mean and standard deviation of the 15 output grids. Other available summary grids are min, max and median.

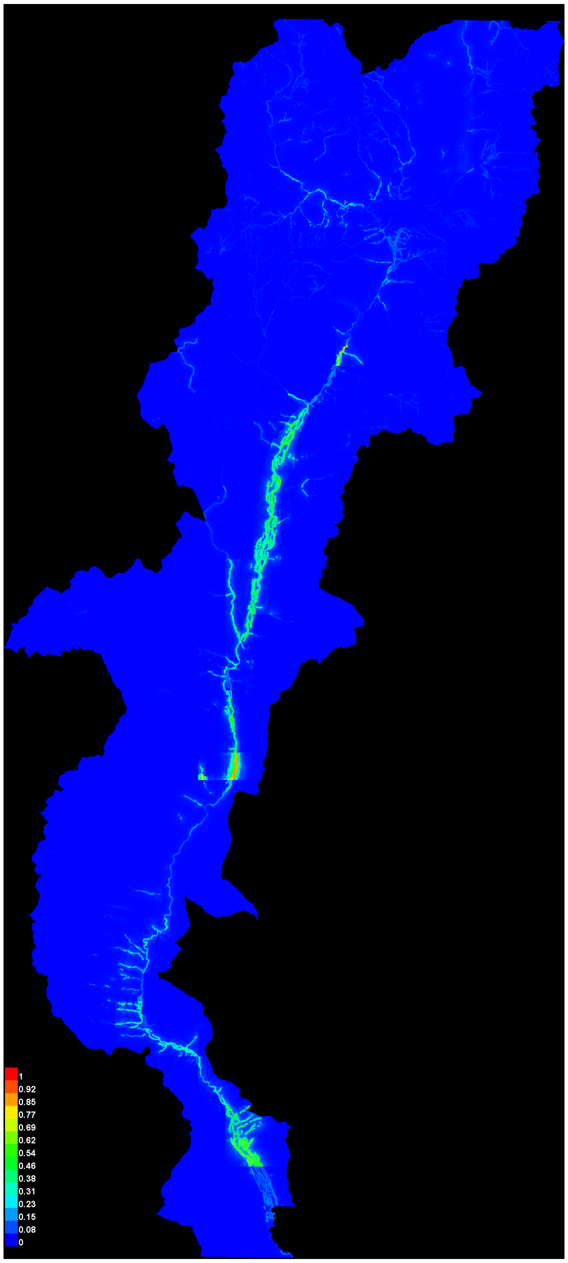

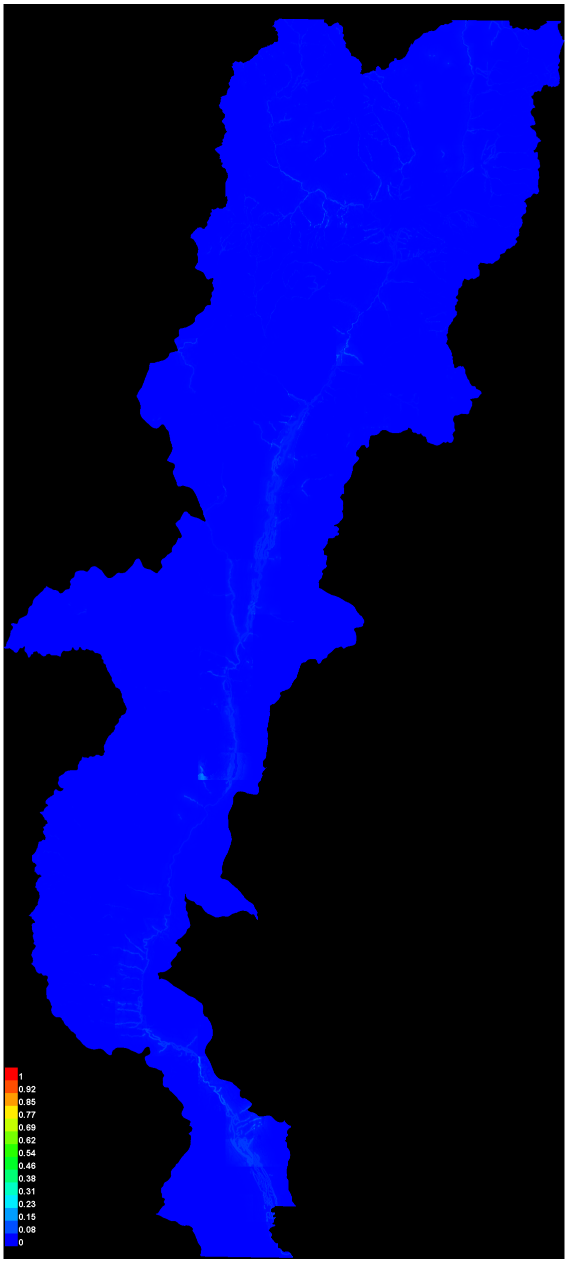

The following two pictures show the point-wise mean and standard deviation of the 15 models applied to the environmental layers in Envir_2030_ascii. Other available summary grids are min, max and median.

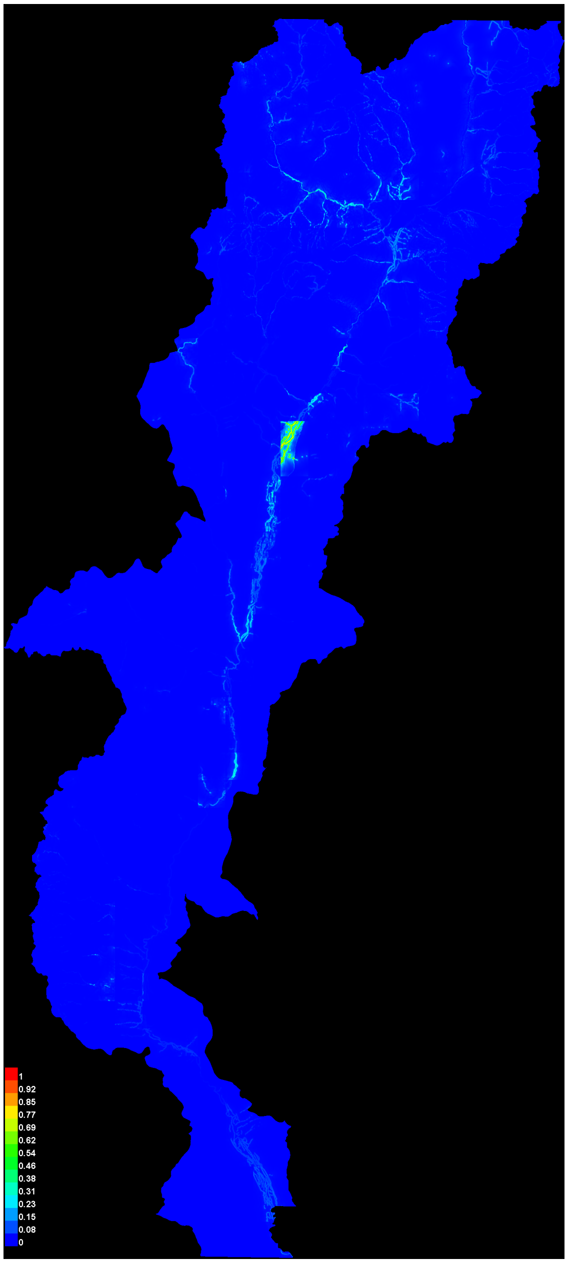

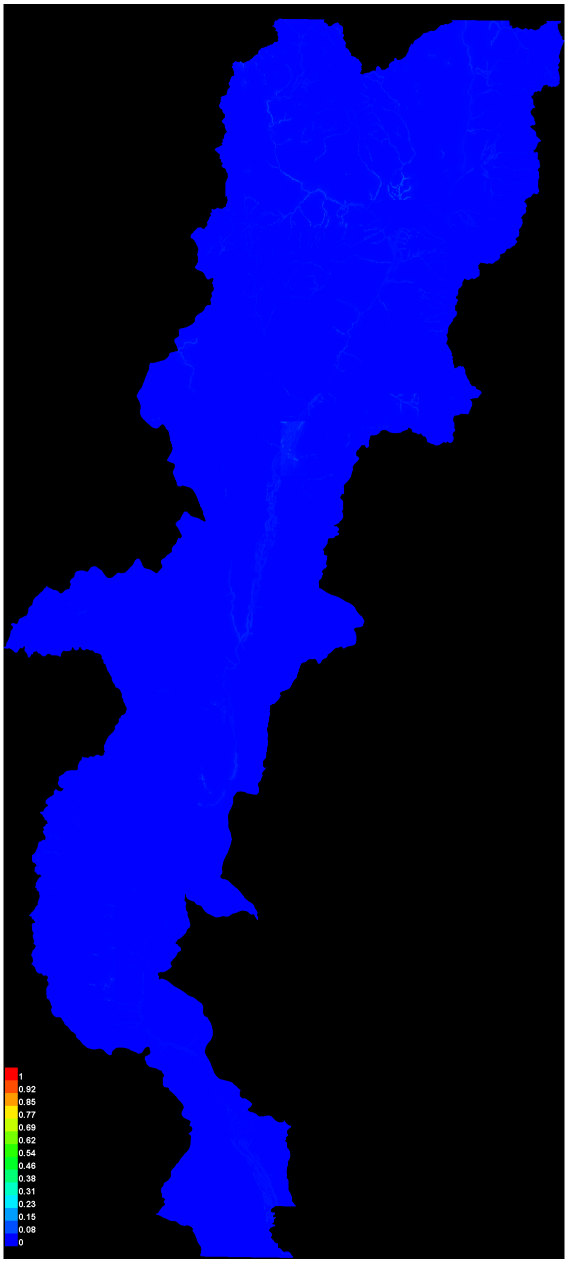

The following two pictures show the point-wise mean and standard deviation of the 15 models applied to the environmental layers in Envir_2060_ascii. Other available summary grids are min, max and median.

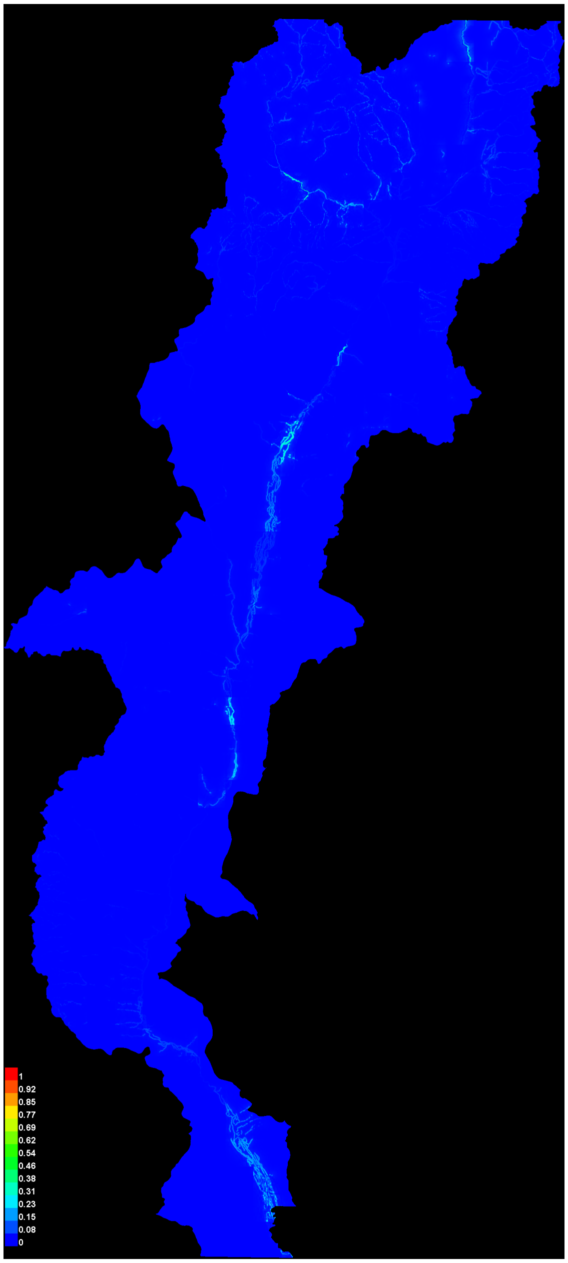

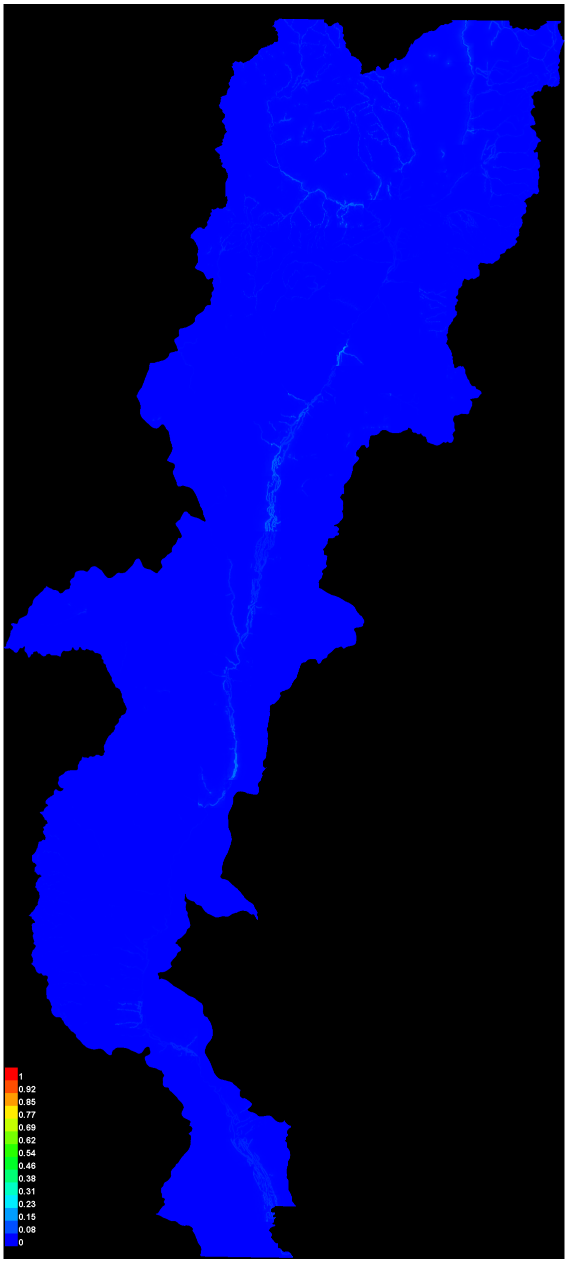

The following two pictures show the point-wise mean and standard deviation of the 15 models applied to the environmental layers in Envir_2090_ascii. Other available summary grids are min, max and median.

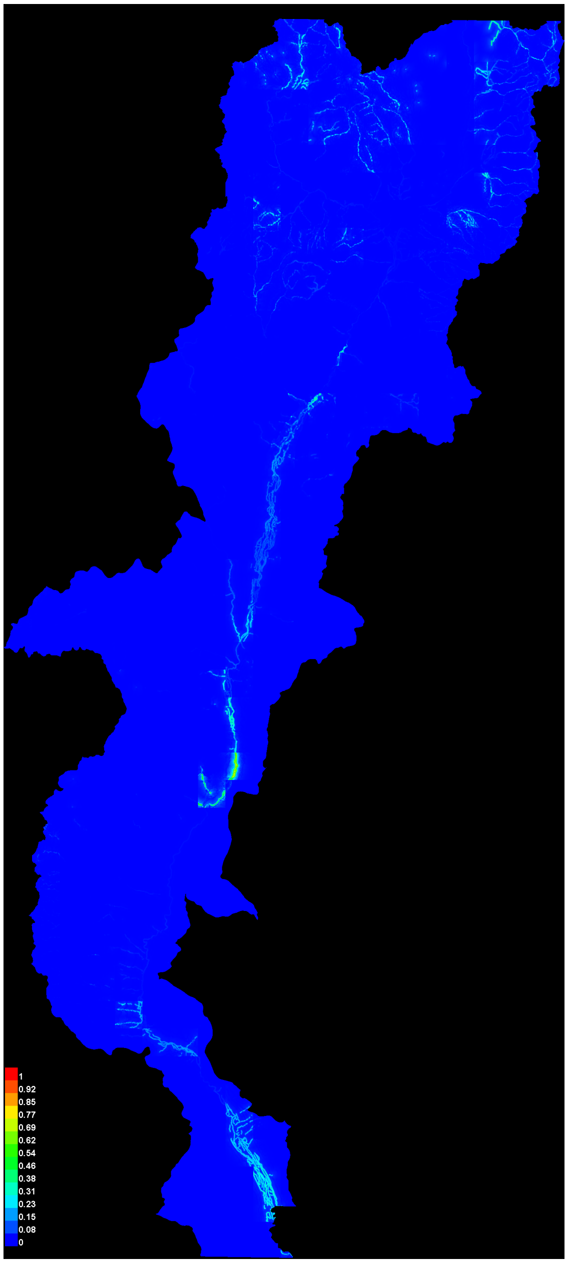

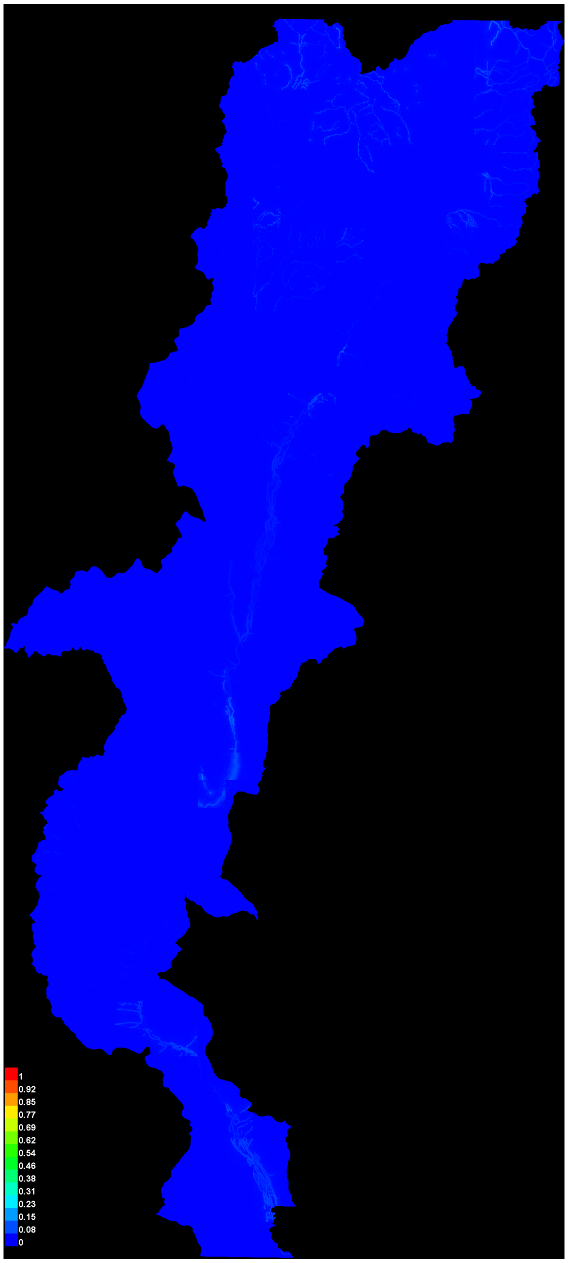

Response curves

These curves show how each environmental variable affects the Maxent prediction. The curves show how the logistic prediction changes as each environmental variable is varied, keeping all other environmental variables at their average sample value. Click on a response curve to see a larger version. Note that the curves can be hard to interpret if you have strongly correlated variables, as the model may depend on the correlations in ways that are not evident in the curves. In other words, the curves show the marginal effect of changing exactly one variable, whereas the model may take advantage of sets of variables changing together. The curves show the mean response of the 15 replicate Maxent runs (red) and and the mean +/- one standard deviation (blue, two shades for categorical variables).

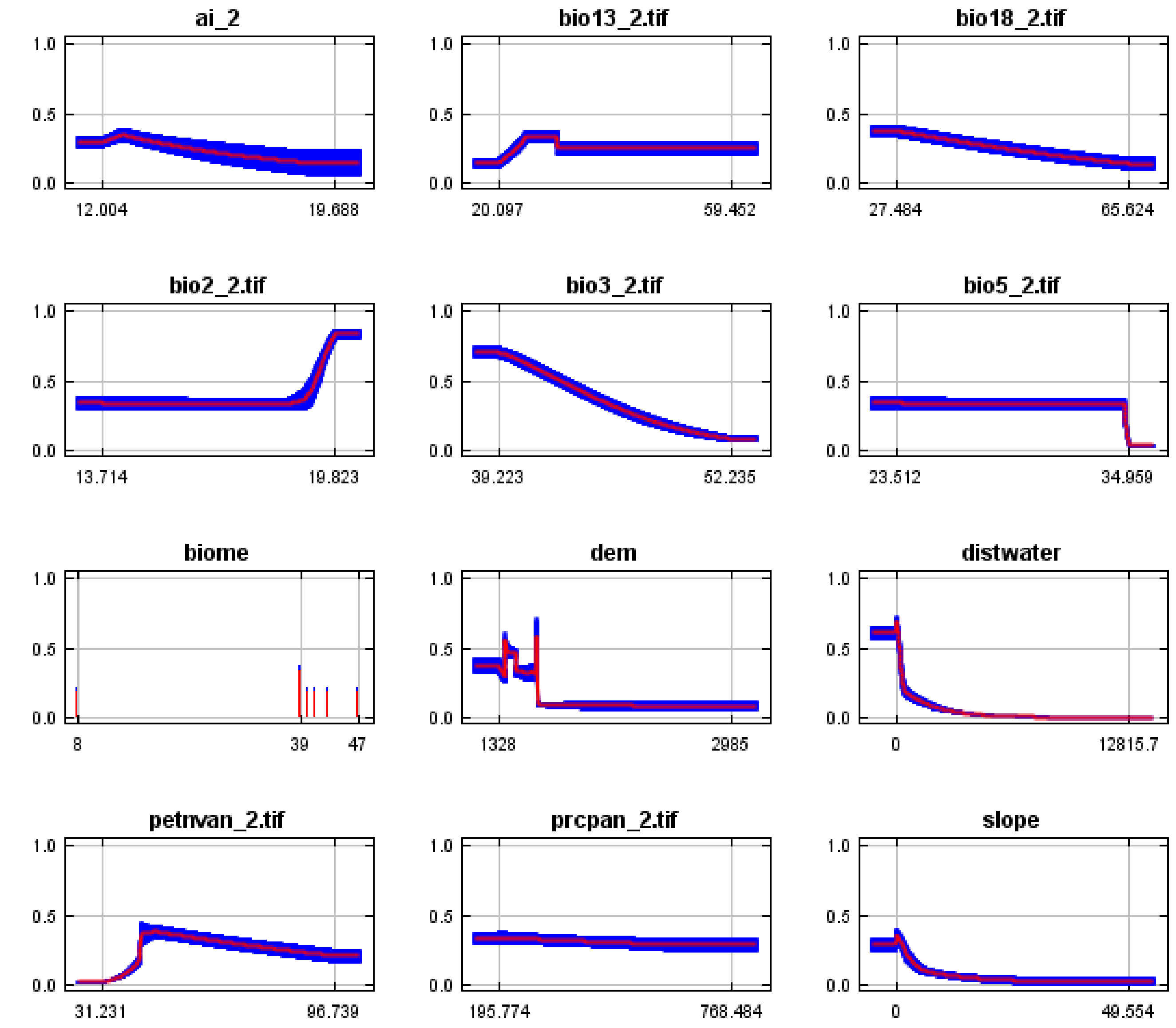

In contrast to the above marginal response curves, each of the following curves represents a different model, namely, a Maxent model created using only the corresponding variable. These plots reflect the dependence of predicted suitability both on the selected variable and on dependencies induced by correlations between the selected variable and other variables. They may be easier to interpret if there are strong correlations between variables.

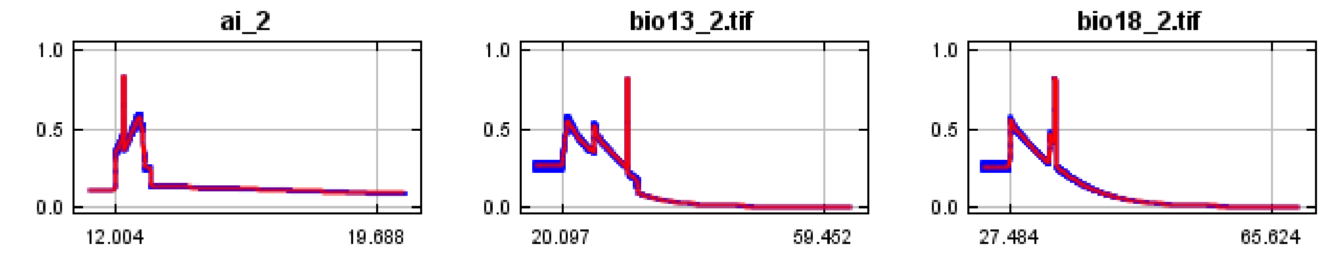

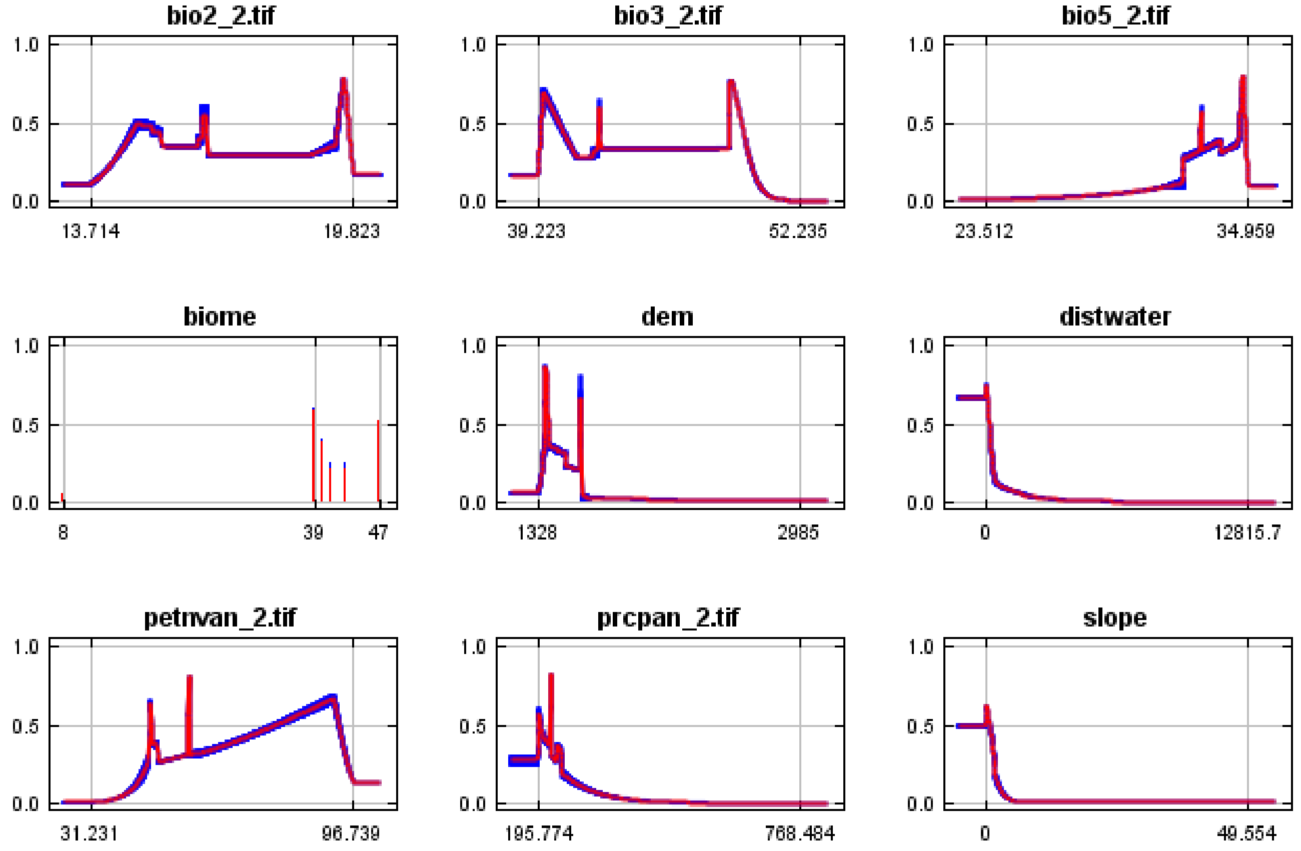

Analysis of variable contributions

The following table gives estimates of relative contributions of the environmental variables to the Maxent model. To determine the first estimate, in each iteration of the training algorithm, the increase in regularized gain is added to the contribution of the corresponding variable, or subtracted from it if the change to the absolute value of lambda is negative. For the second estimate, for each environmental variable in turn, the values of that variable on training presence and background data are randomly permuted. The model is reevaluated on the permuted data, and the resulting drop in training AUC is shown in the table, normalized to percentages. As with the variable jackknife, variable contributions should be interpreted with caution when the predictor variables are correlated. Values shown are averages over replicate runs.

Variable	Percent contribution	Permutation importance
distwater	58.1	57.5
dem	19.6	13.9
bio2_2.tif	5.7	3
petnvan_2.tif	4.8	7.2
slope	2.9	6
bio13_2.tif	2.5	0.8
bio5_2.tif	2.5	2.9
biome	1.8	1.5

bio18_2.tif	1	0.5
ai_2	0.5	0.7
bio3_2.tif	0.4	5.9
prcpan_2.tif	0.2	0

The following picture shows the results of the jackknife test of variable importance. The environmental variable with highest gain when used in isolation is distwater, which therefore appears to have the most useful information by itself. The environmental variable that decreases the gain the most when it is omitted is distwater, which therefore appears to have the most information that isn't present in the other variables. Values shown are averages over replicate runs.

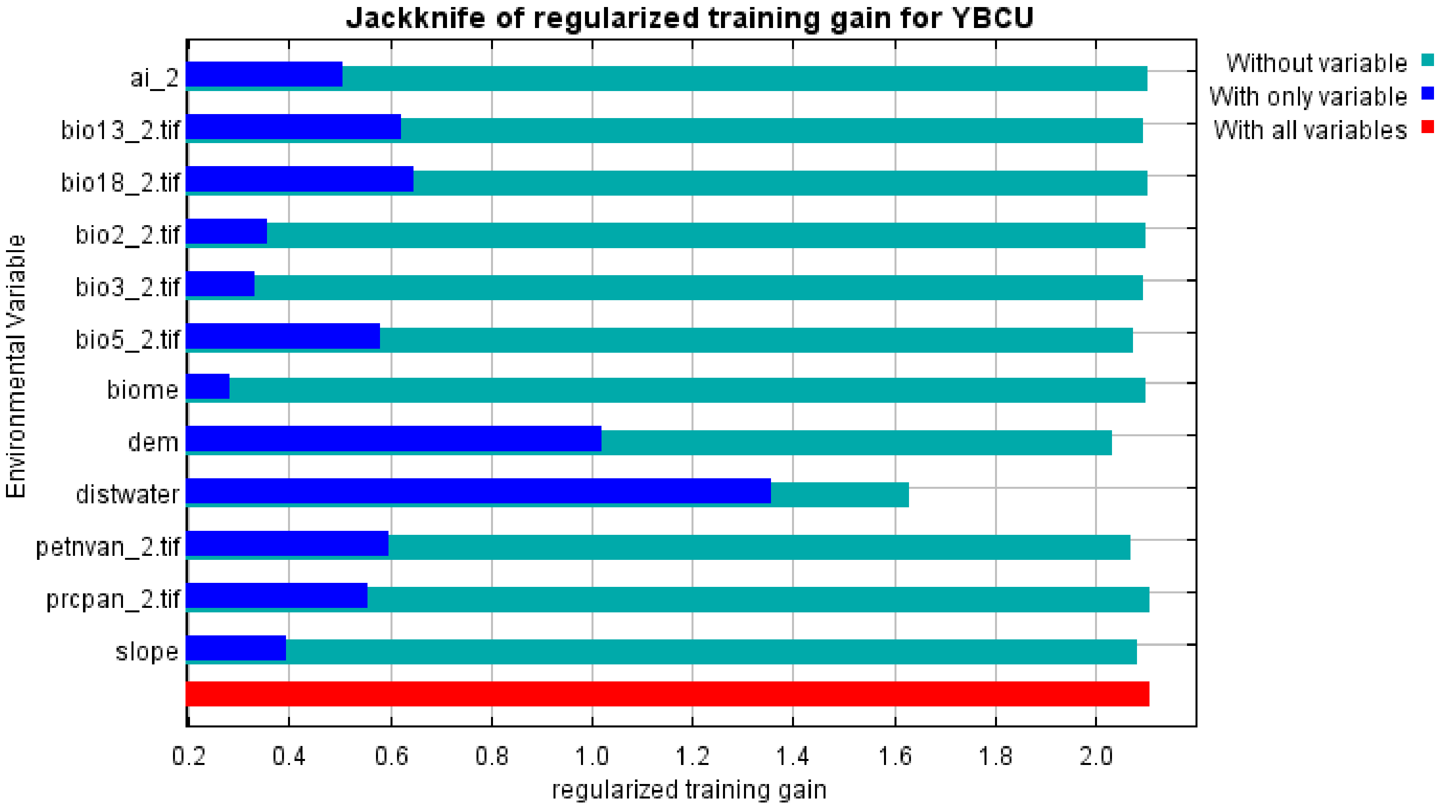

The next picture shows the same jackknife test, using test gain instead of training gain. Note that conclusions about which variables are most important can change, now that we're looking at test data.

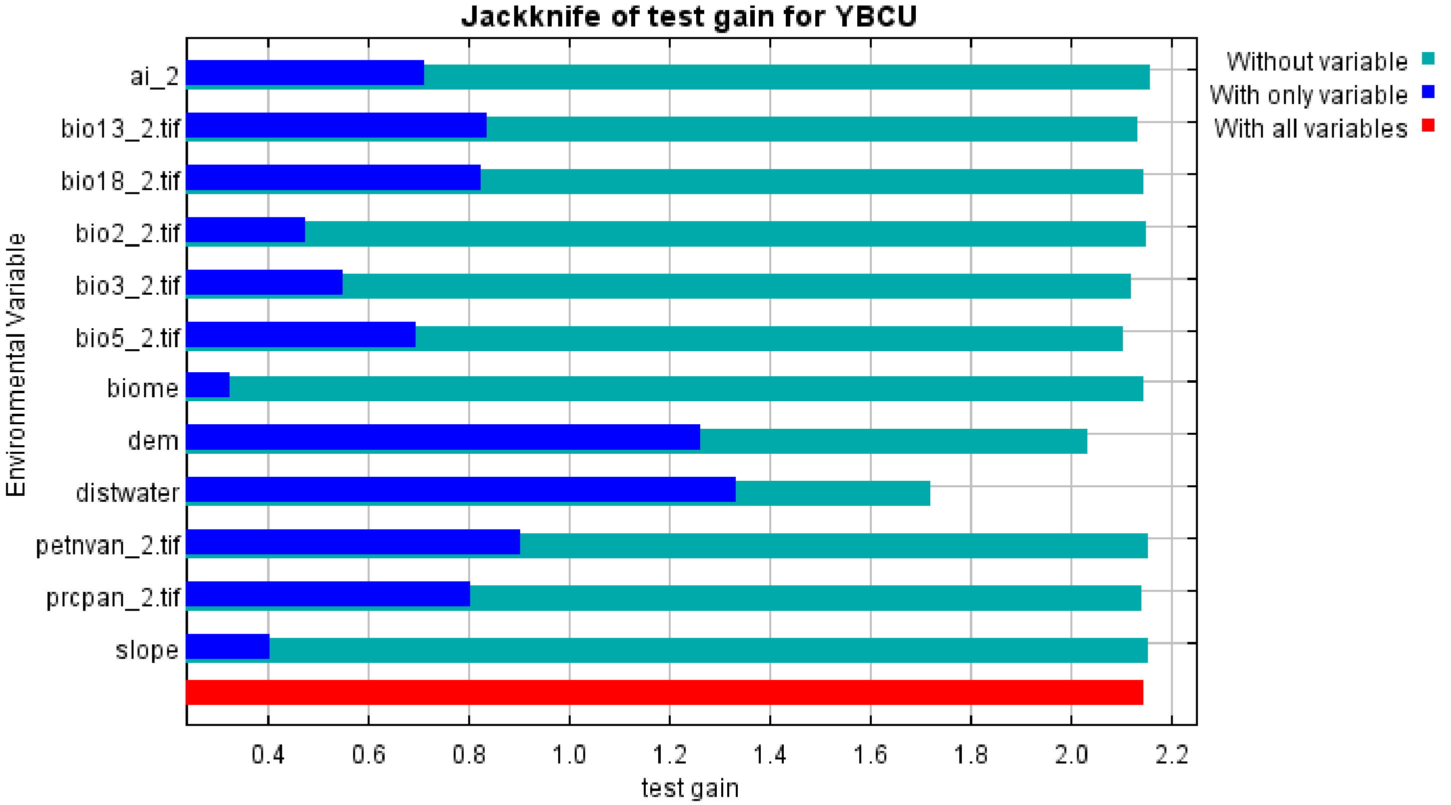

Lastly, we have the same jackknife test, using AUC on test data.

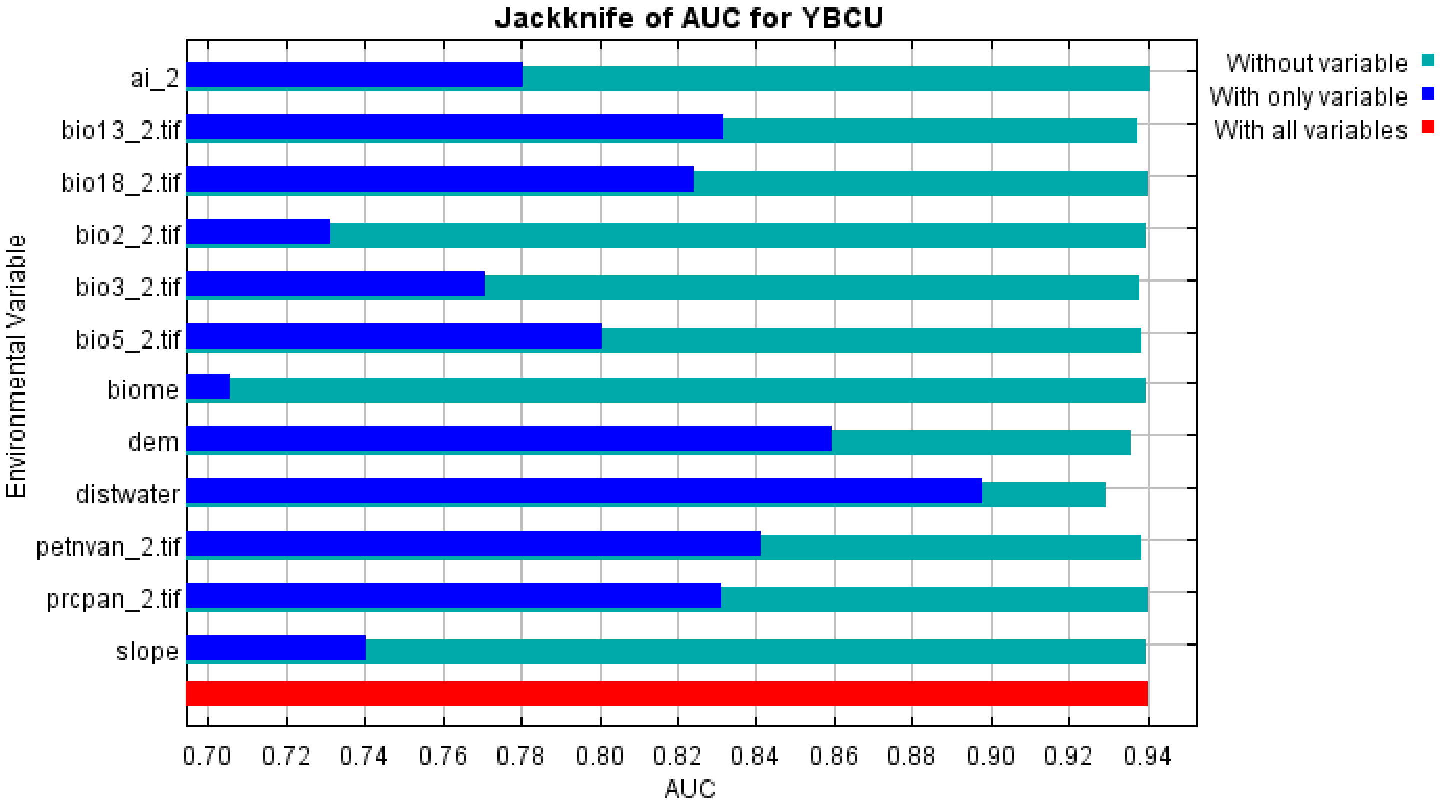

Command line to repeat this species model: java density.MaxEnt nowarnings noprefixes -E "" -E YBCU responsecurves jackknife outputdirectory=D:\MaxEnt4\BirdsGd

"projectionlayers=D:\Ascii_Files\Envir_2030_ascii, D:\Ascii_Files\Envir_2060_ascii, D:\Ascii_Files\Envir_2090_ascii" samplesfile=D:\Data\RioGrande\Birds\allbirds.csv environmentallayers=D:\Ascii_Files\Envir_Curr_ascii randomseed biasfile=D:\Data\RioGrande\Birds\all_bias.asc replicates=15 nooutputgrids maximumiterations=5000 biastype=3 "applythresholdrule=equal training sensitivity and specificity" -N ai_1 -N ai_3 -N bio10_1 -N bio10_1.tif -N bio10_2 -N bio10_2.tif -N bio10_3 -N bio10_3.tif -N bio11_1 -N bio11_1.tif -N bio11_2 -N bio11_2.tif -N bio11_3 -N bio11_3.tif -N bio13_1.tif -N bio13_3.tif -N bio14_1.tif -N bio14_2.tif -N bio14_3.tif -N bio16_1.tif -N bio16_2.tif -N bio16_3.tif -N bio17_.tif -N bio17_2.tif -N bio17_3.tif -N bio17_all.tif -N bio18_1.tif -N bio18_3.tif -N bio19_1.tif -N bio19_2.tif -N bio19_3.tif -N bio2_1.tif -N bio2_3.tif -N bio3_1.tif -N bio3_3.tif -N bio5_1.tif -N bio5_3.tif -N bio6_1.tif -N bio6_2.tif -N bio6_3.tif -N bio7_1.tif -N bio7_2.tif -N bio7_3.tif -N etan_1.tif -N etan_2.tif -N etan_3.tif -N etdjf_1.tif -N etdjf_2.tif -N etdjf_3.tif -N etjas_1.tif -N etjas_2.tif -N etjas_3.tif -N etjfm_1.tif -N etjfm_2.tif -N etjfm_3.tif -N etjja_1.tif -N etjja_2.tif -N etjja_3.tif -N etjja_all.tif -N petnvan_1.tif -N petnvan_3.tif -N petnvdjf_1.tif -N petnvdjf_2.tif -N petnvdjf_3.tif -N petnvjas_1.tif -N petnvjas_2.tif -N petnvjas_3.tif -N petnvjfm_1.tif -N petnvjfm_2.tif -N petnvjfm_3.tif -N petnvjja_1.tif -N petnvjja_2.tif -N petnvjja_3.tif -N petwan_1.tif -N petwan_2.tif -N petwan_3.tif -N petwdjf_1.tif -N petwdjf_2.tif -N petwdjf_3.tif -N petwjas_1.tif -N petwjas_2.tif -N petwjas_3.tif -N petwjas_all.tif -N petwjfm_1.tif -N petwjfm_2.tif -N petwjfm_3.tif -N petwjja_1.tif -N petwjja_2.tif -N petwjja_3.tif -N prcpan_1.tif -N prcpan_3.tif -N prcpan_all.tif -N runoffan_1.tif -N runoffan_2.tif -N runoffan_3.tif -N runoffdjf_1.tif -N runoffdjf_2.tif -N runoffdjf_3.tif -N runoffjas_1.tif -N runoffjas_2.tif -N runoffjas_3.tif -N runoffjas_all.tif -N runoffjfm_1.tif -N runoffjfm_2.tif -N runoffjfm_3.tif -N runoffjja_1.tif -N runoffjja_2.tif -N runoffjja_3.tif -N smcan_1.tif -N smcan_2.tif -N smcan_3.tif -N smcdjf_1.tif -N smcdjf_2.tif -N smcdjf_3.tif -N smcjas_1.tif -N smcjas_2.tif -N smcjas_3.tif -N smcjfm_1.tif -N smcjfm_2.tif -N smcjfm_3.tif -N smcjja_1.tif -N smcjja_2.tif -N smcjja_3.tif -N swean_1.tif -N swean_2.tif -N swean_3.tif -N swedjf_1.tif -N swedjf_2.tif -N swedjf_3.tif -N swejfm_1.tif -N swejfm_2.tif -N swejfm_3.tif -N tave_1.tif -N tave_2.tif -N tave_3.tif -N tmax_1.tif -N tmax_2.tif -N tmax_3.tif -N tmin_1.tif -N tmin_2.tif -N tmin_3.tif -t biome

Replicated maxent model for YBCU

This page summarizes the results of 15-fold cross-validation for YBCU, created Wed Aug 06 11:17:12 MDT 2014 using Maxent version 3.3.3k. The individual models are here: [0] [1] [2] [3] [4] [5] [6] [7] [8] [9] [10] [11] [12] [13] [14]

Analysis of omission/commission

The following picture shows the test omission rate and predicted area as a function of the cumulative threshold, averaged over the replicate runs. The omission rate should be close to the predicted omission, because of the definition of the cumulative threshold.

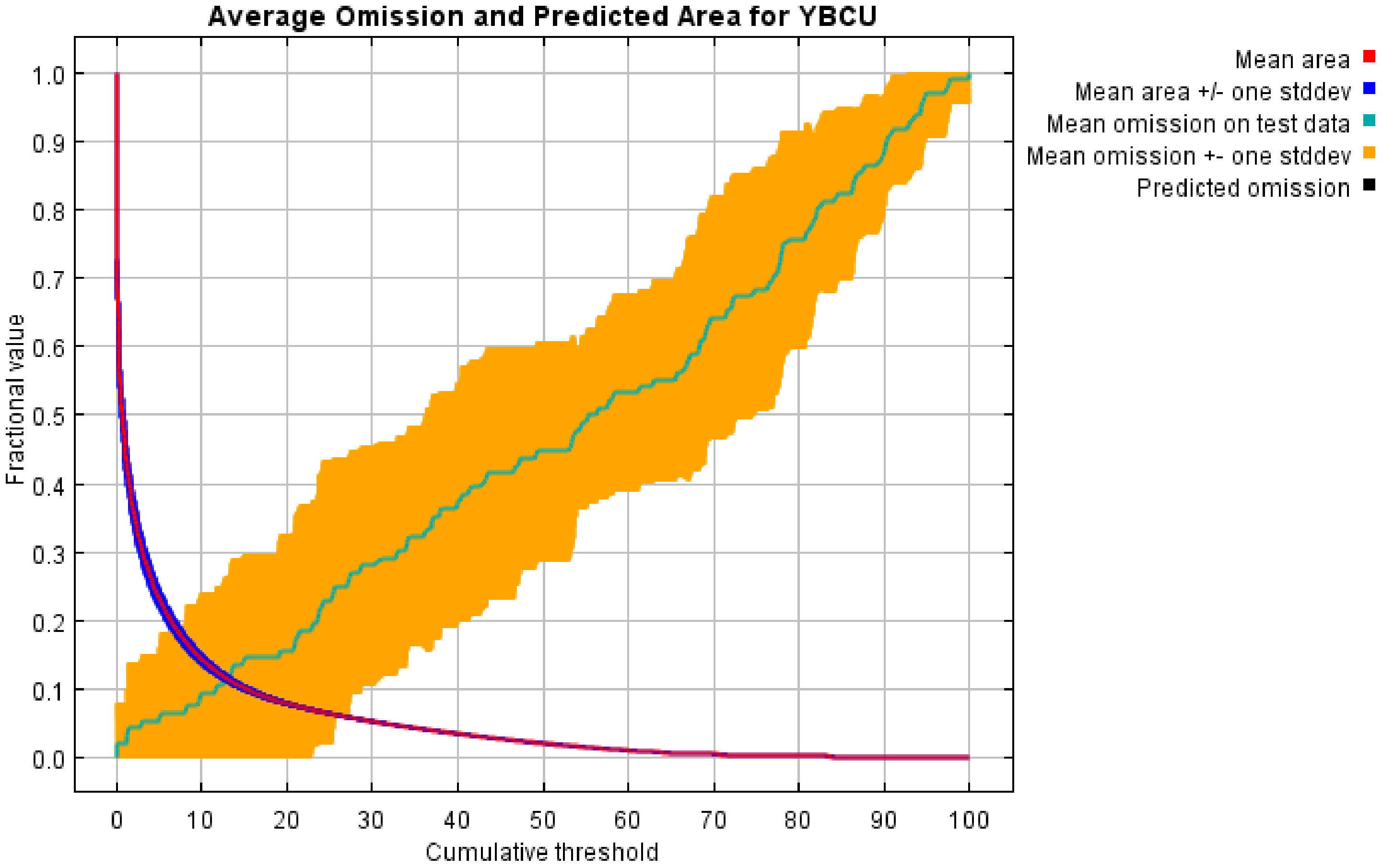

The next picture is the receiver operating characteristic (ROC) curve for the same data, again averaged over the replicate runs. Note that the specificity is defined using predicted area, rather than true commission (see the paper by Phillips, Anderson and Schapire cited on the help page for discussion of what this means). The average test AUC for the replicate runs is 0.940, and the standard deviation is 0.056.

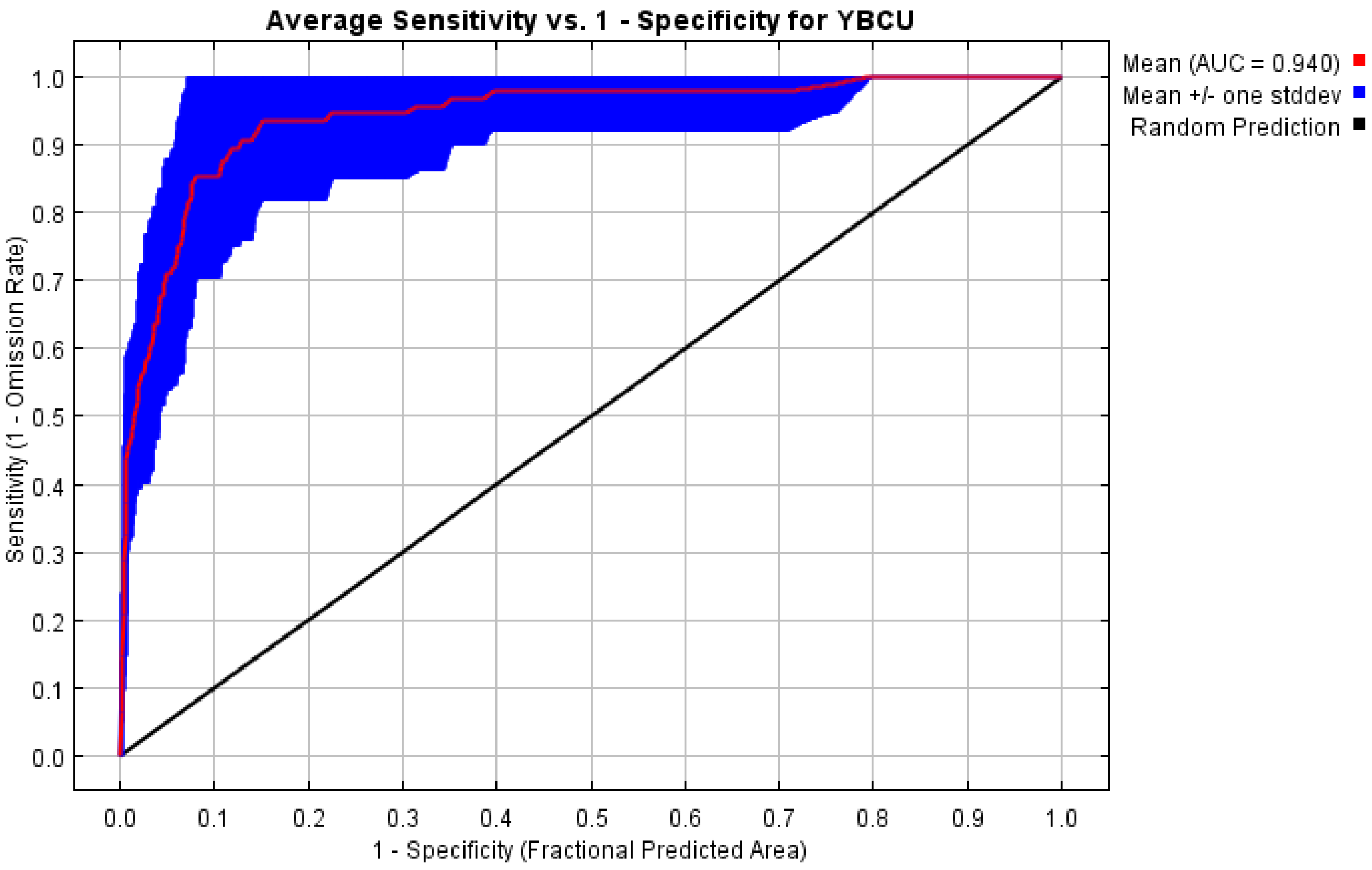

Pictures of the model

The following two pictures show the point-wise mean and standard deviation of the 15 output grids. Other available summary grids are min, max and median.

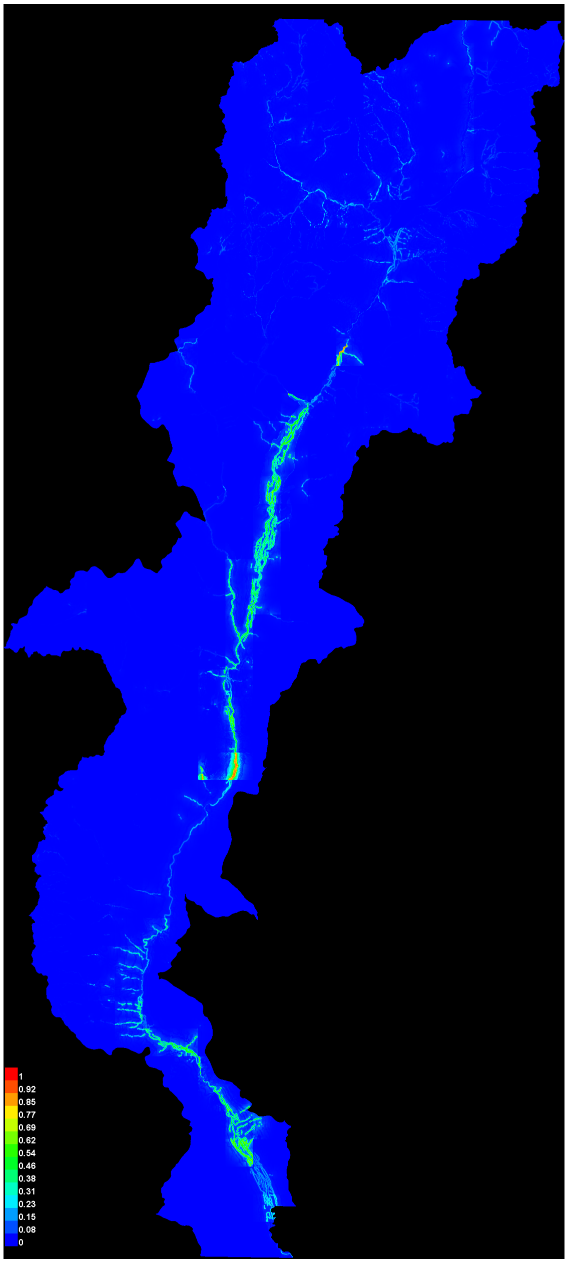

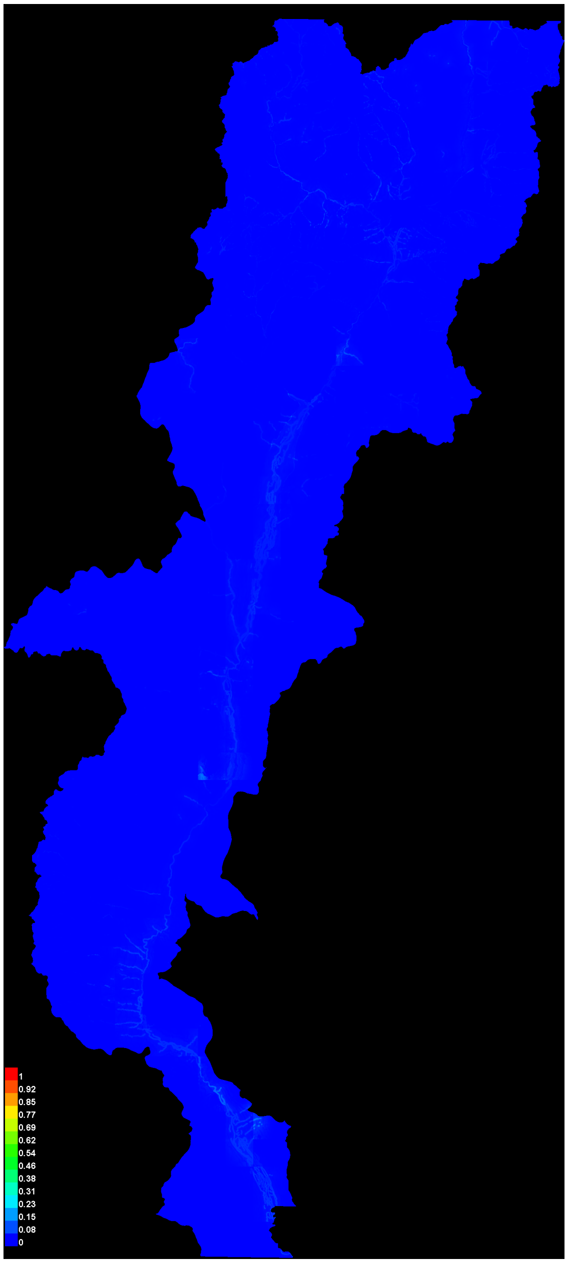

The following two pictures show the point-wise mean and standard deviation of the 15 models applied to the environmental layers in Envir_2030_ascii. Other available summary grids are min, max and median.

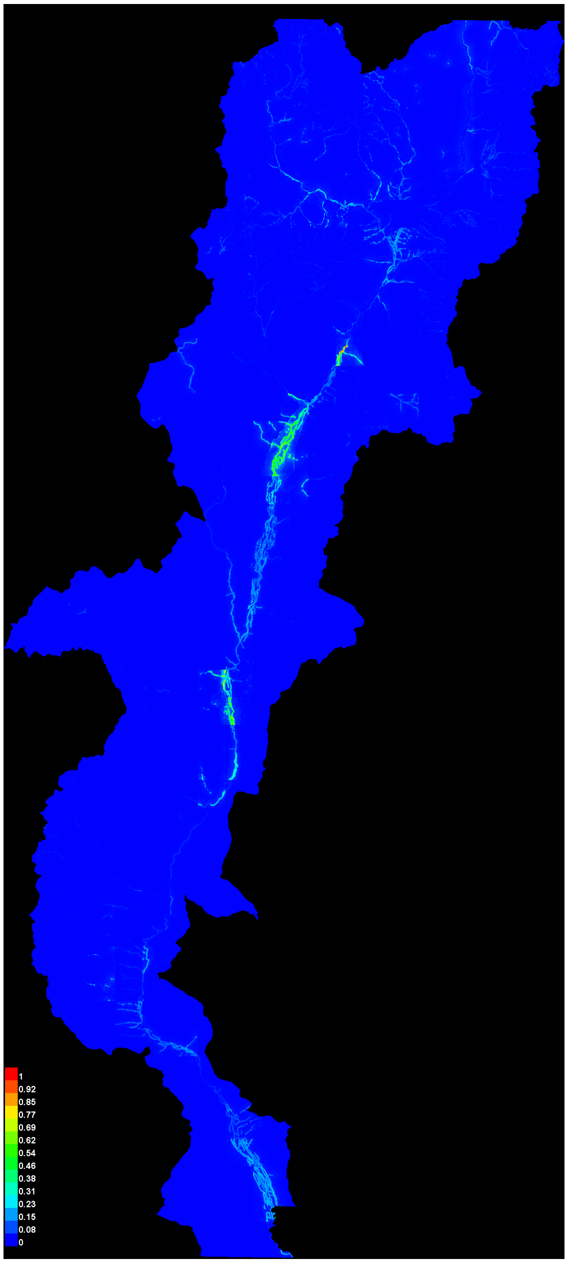

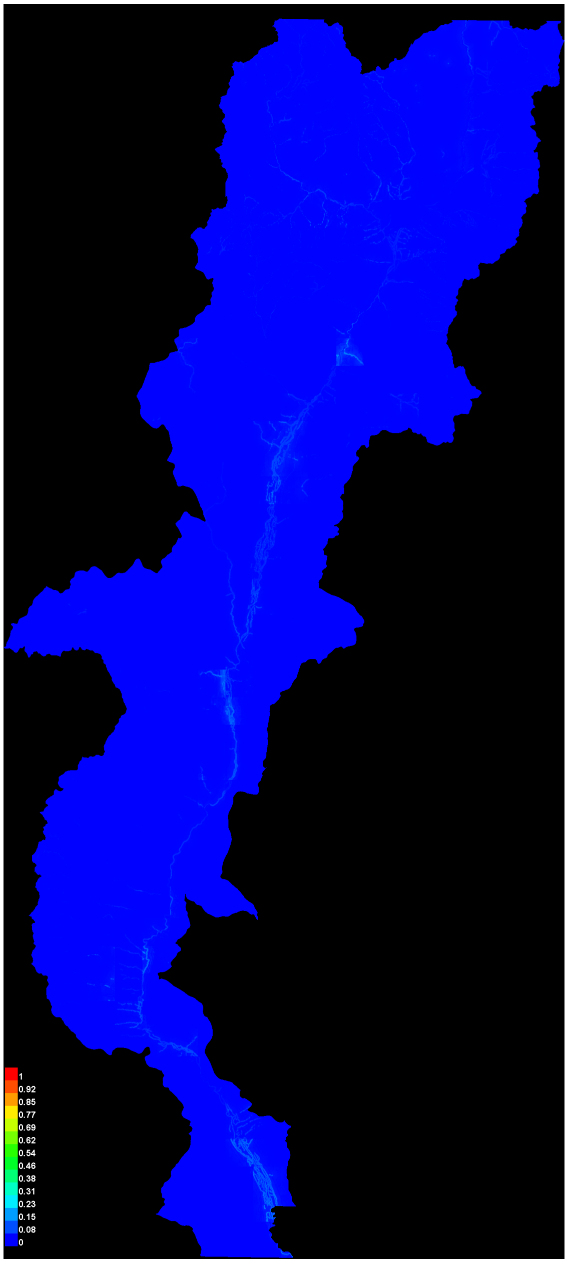

The following two pictures show the point-wise mean and standard deviation of the 15 models applied to the environmental layers in Envir_2060_ascii. Other available summary grids are min, max and median.

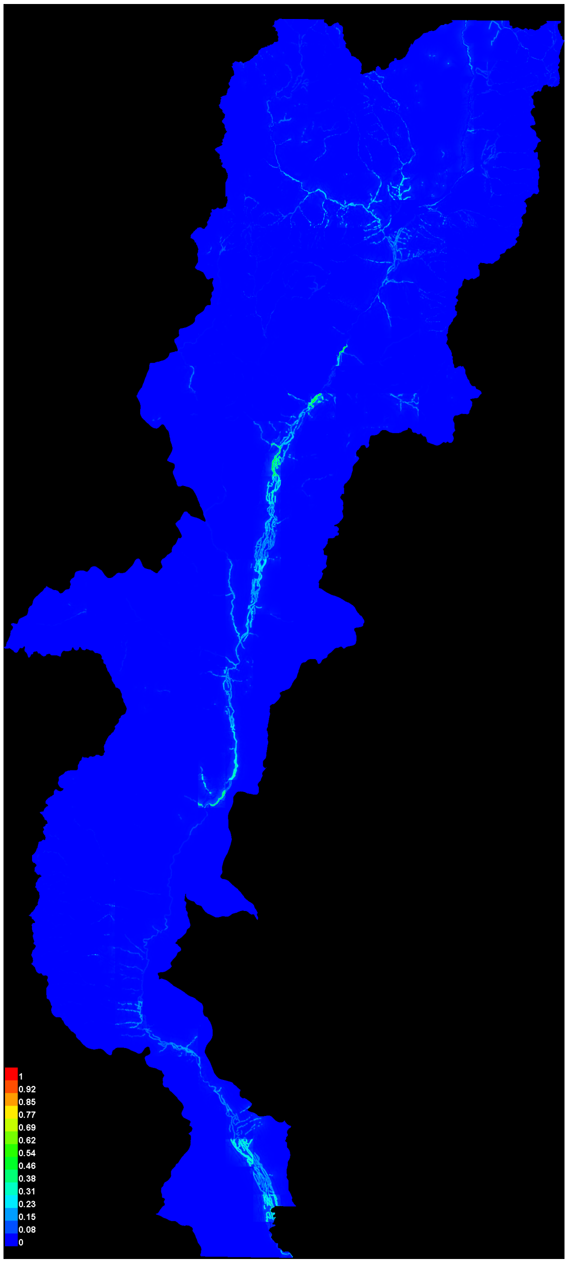

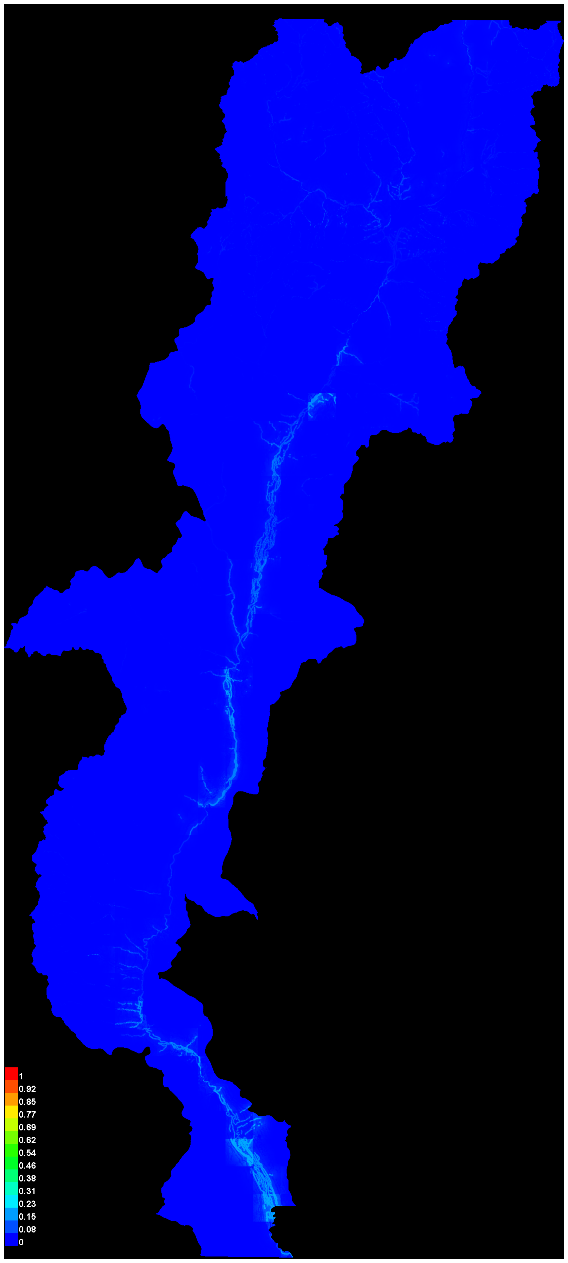

The following two pictures show the point-wise mean and standard deviation of the 15 models applied to the environmental layers in Envir_2090_ascii. Other available summary grids are min, max and median.

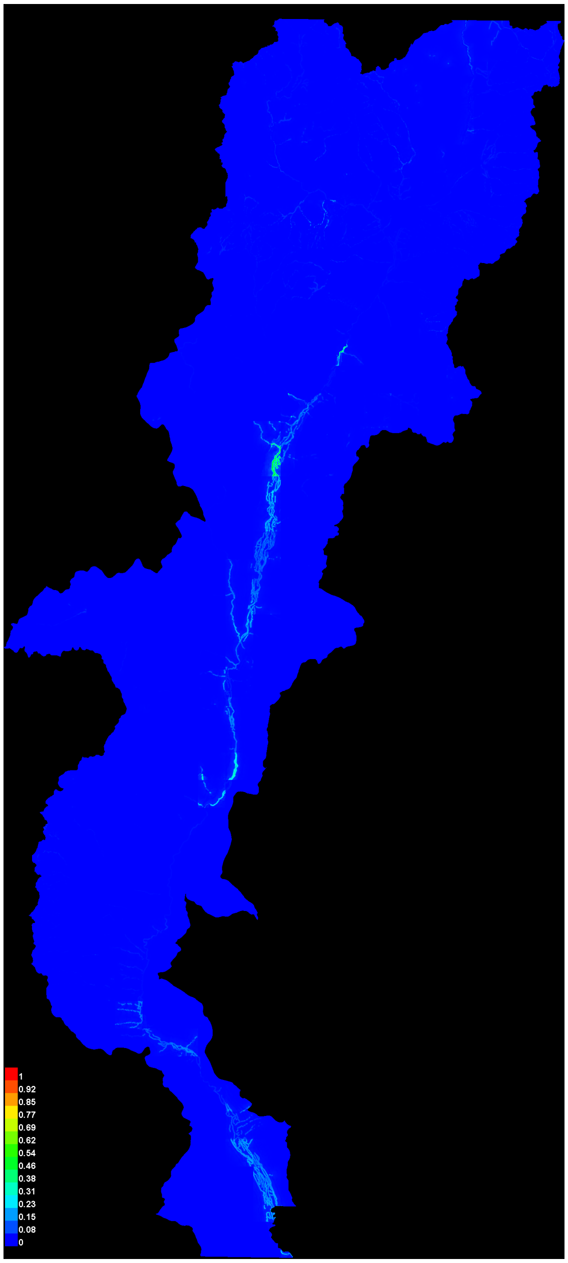

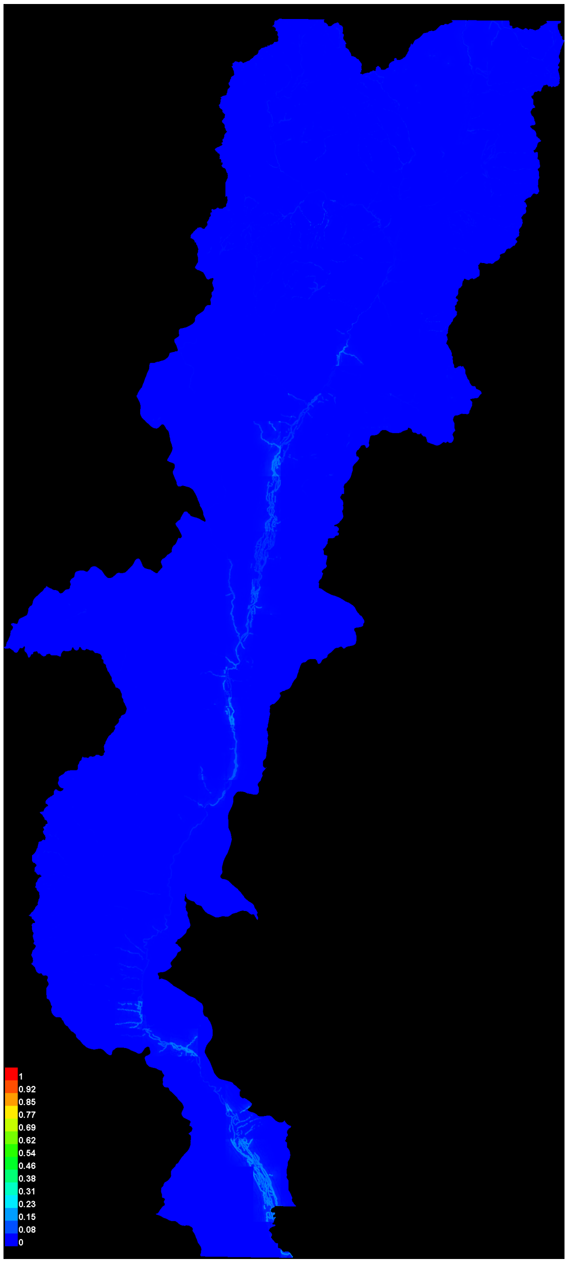

Response curves

These curves show how each environmental variable affects the Maxent prediction. The curves show how the logistic prediction changes as each environmental variable is varied, keeping all other environmental variables at their average sample value. Click on a response curve to see a larger version. Note that the curves can be hard to interpret if you have strongly correlated variables, as the model may depend on the correlations in ways that are not evident in the curves. In other words, the curves show the marginal effect of changing exactly one variable, whereas the model may take advantage of sets of variables changing together. The curves show the mean response of the 15 replicate Maxent runs (red) and and the mean +/- one standard deviation (blue, two shades for categorical variables).

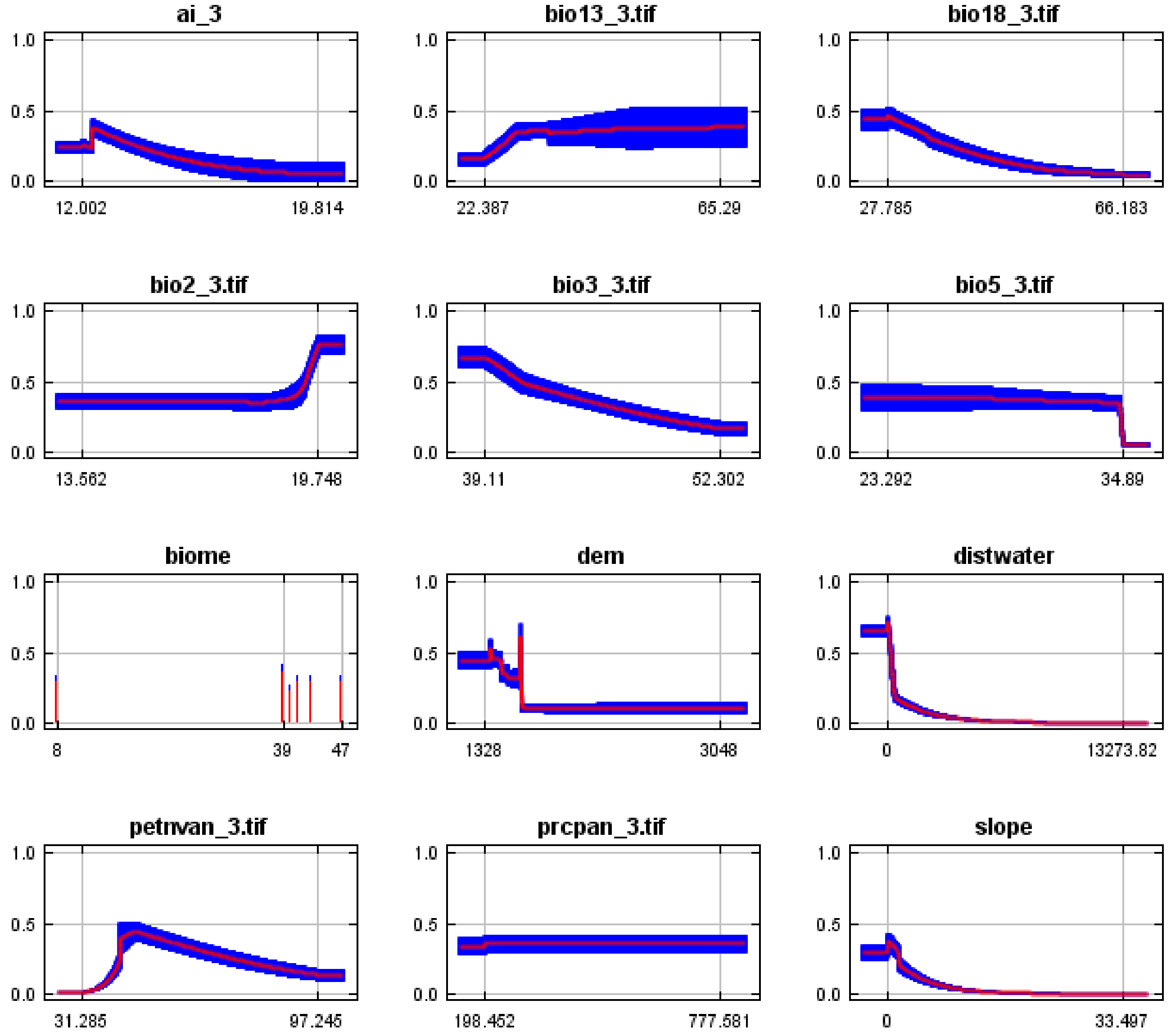

In contrast to the above marginal response curves, each of the following curves represents a different model, namely, a Maxent model created using only the corresponding variable. These plots reflect the dependence of predicted suitability both on the selected variable and on dependencies induced by correlations between the selected variable and other variables. They may be easier to interpret if there are strong correlations between variables.

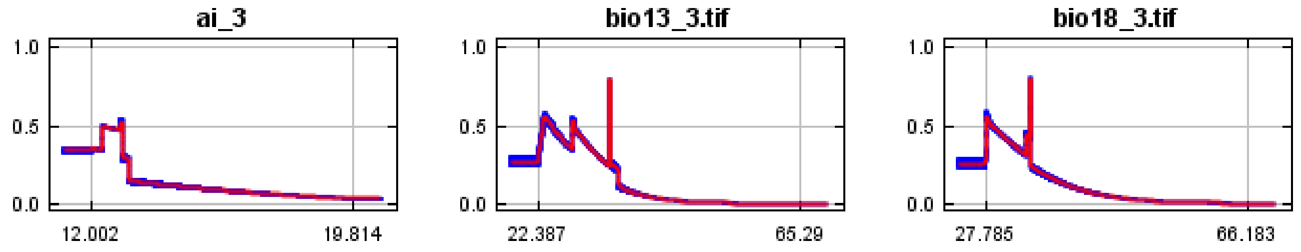

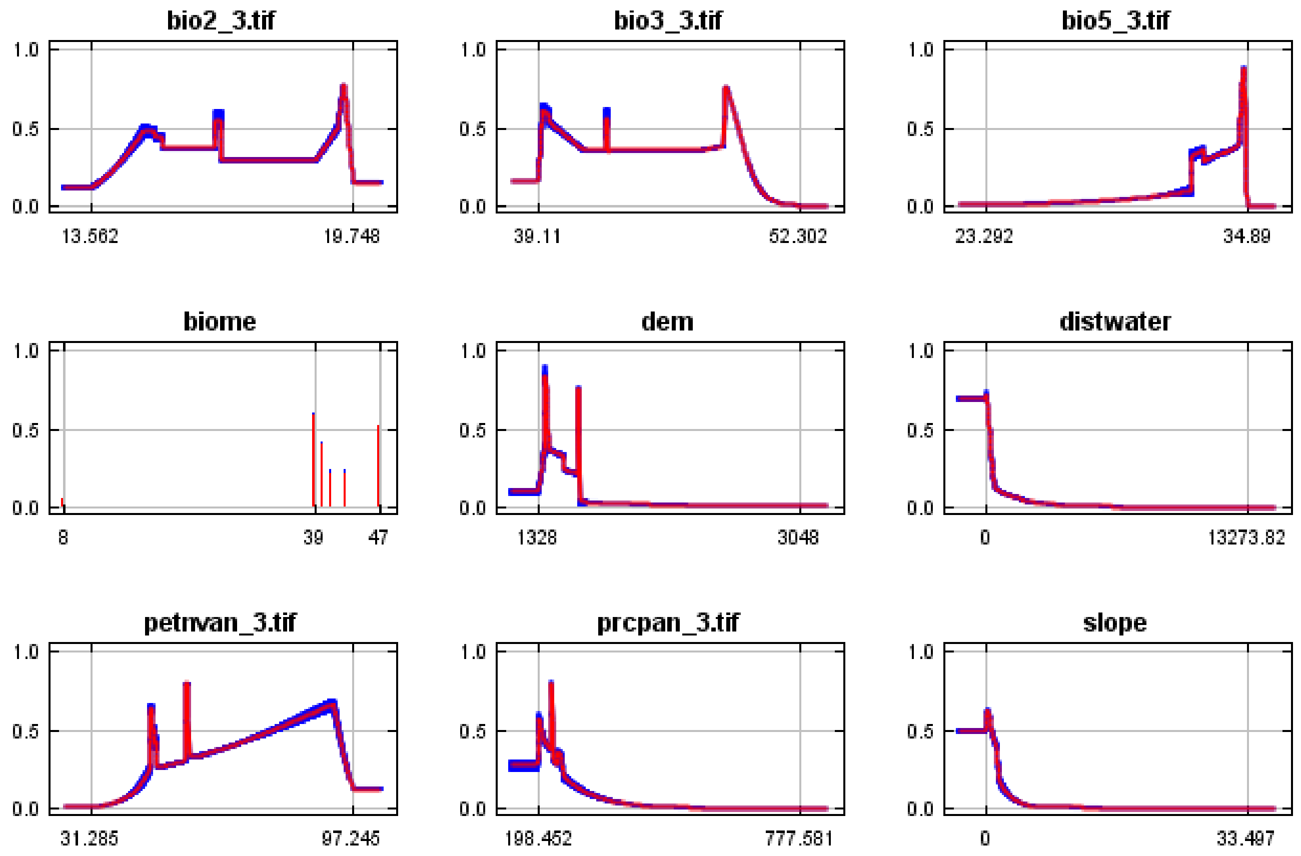

Analysis of variable contributions

The following table gives estimates of relative contributions of the environmental variables to the Maxent model. To determine the first estimate, in each iteration of the training algorithm, the increase in regularized gain is added to the contribution of the corresponding variable, or subtracted from it if the change to the absolute value of lambda is negative. For the second estimate, for each environmental variable in turn, the values of that variable on training presence and background data are randomly permuted. The model is reevaluated on the permuted data, and the resulting drop in training AUC is shown in the table, normalized to percentages. As with the variable jackknife, variable contributions should be interpreted with caution when the predictor variables are correlated. Values shown are averages over replicate runs.

Variable	Percent contribution	Permutation importance
distwater	56.7	56.8
dem	19.7	10.2
petnvan_3.tif	5.8	9.3
bio2_3.tif	4.3	0.8
slope	3.8	8.3
ai_3	3.3	1.5
bio5_3.tif	2.2	1.4
bio18_3.tif	2.2	5.8

biome	0.9	1
bio13_3.tif	0.5	1.1
prcpan_3.tif	0.3	0
bio3_3.tif	0.2	3.8

The following picture shows the results of the jackknife test of variable importance. The environmental variable with highest gain when used in isolation is distwater, which therefore appears to have the most useful information by itself. The environmental variable that decreases the gain the most when it is omitted is distwater, which therefore appears to have the most information that isn't present in the other variables. Values shown are averages over replicate runs.

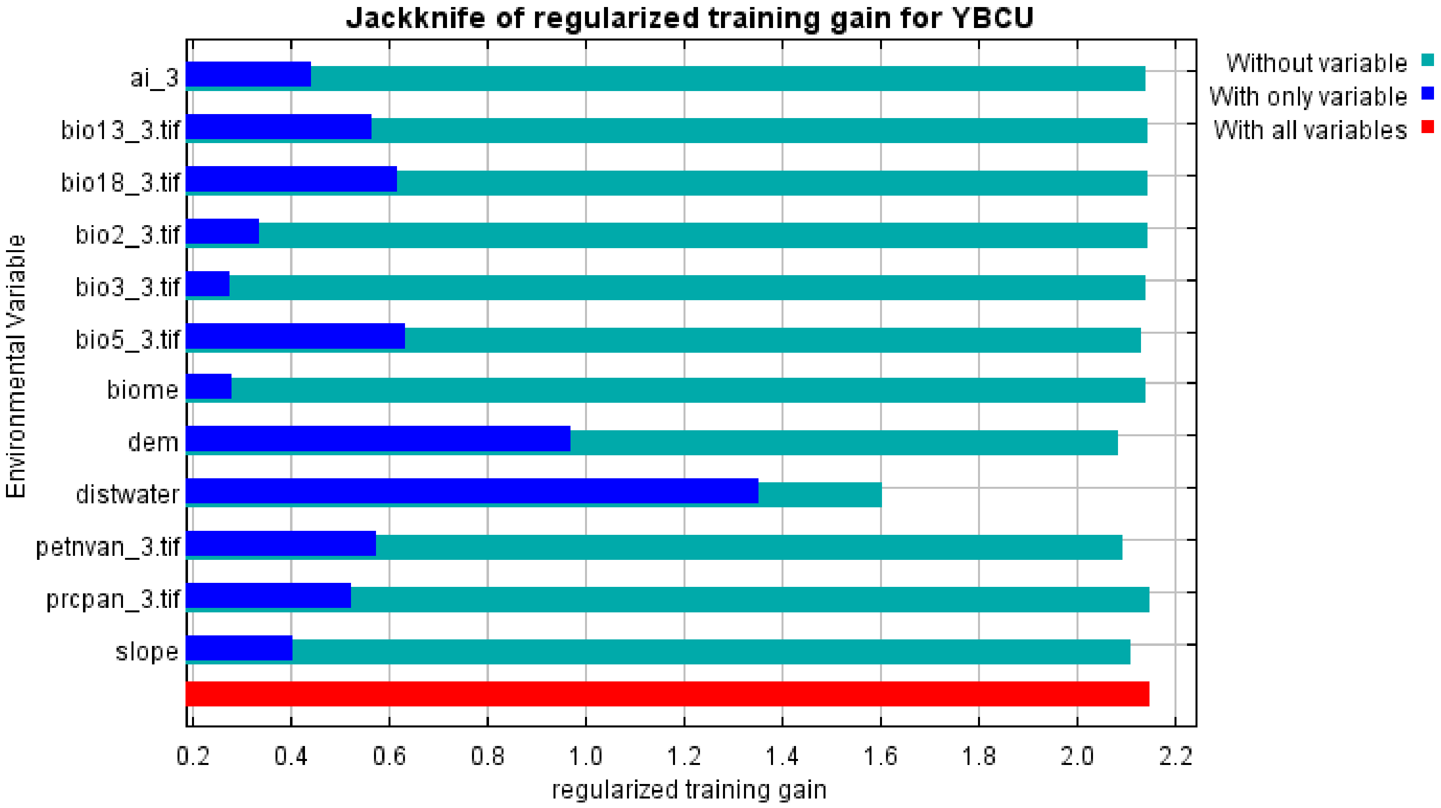

The next picture shows the same jackknife test, using test gain instead of training gain. Note that conclusions about which variables are most important can change, now that we're looking at test data.

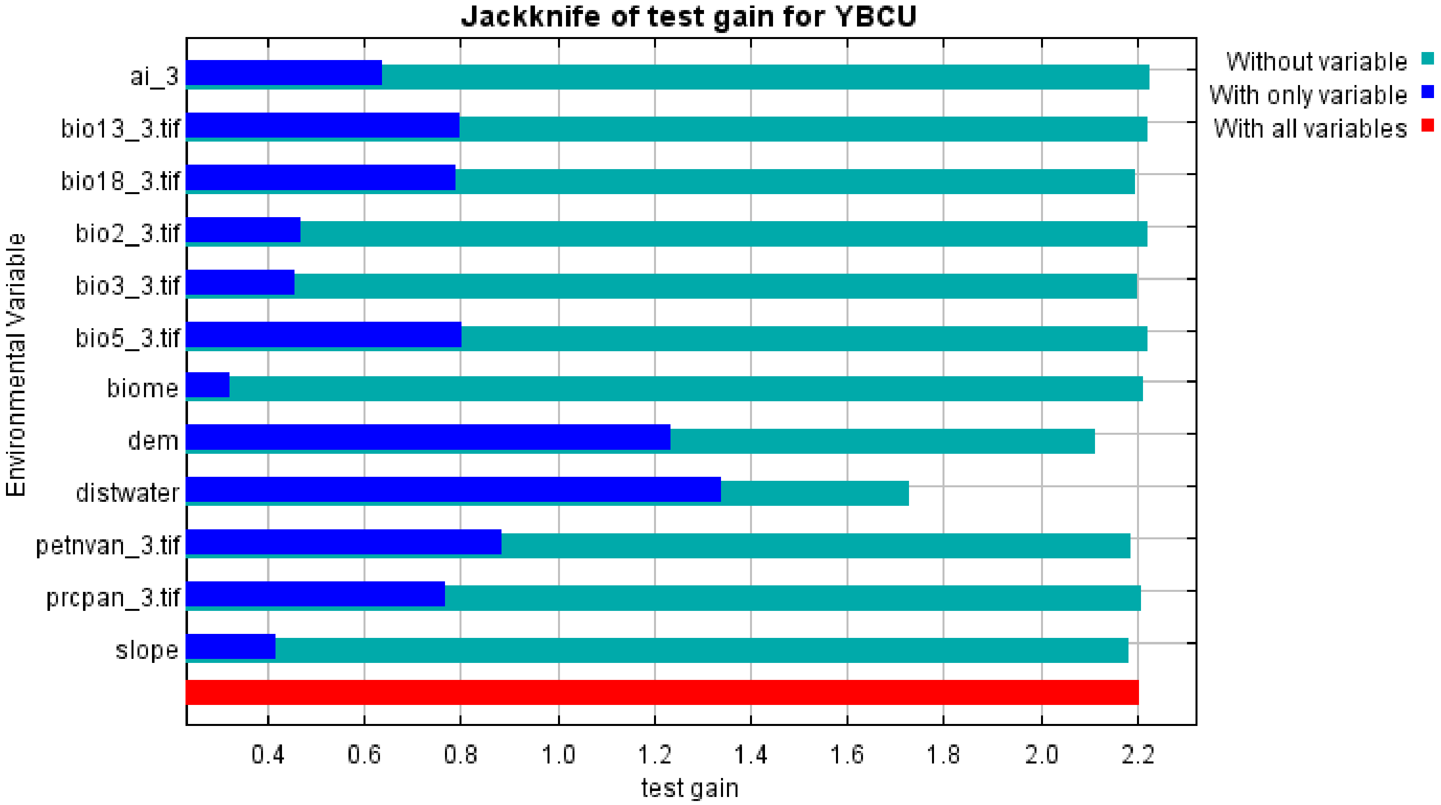

Lastly, we have the same jackknife test, using AUC on test data.

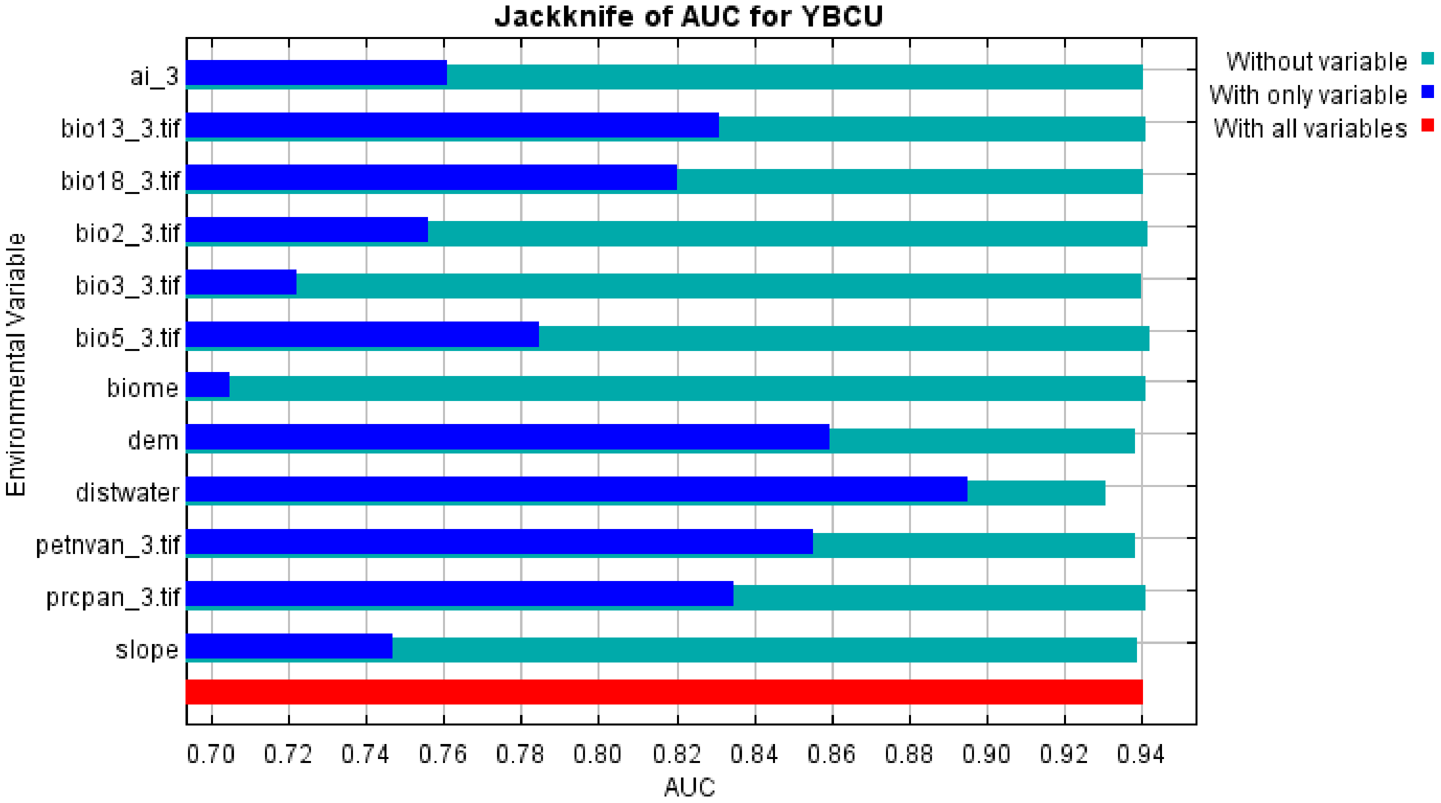

Command line to repeat this species model: java density.MaxEnt nowarnings noprefixes -E "" -E YBCU responsecurves jackknife outputdirectory=D:\MaxEnt4\BirdsHad

"projectionlayers=D:\Ascii_Files\Envir_2030_ascii, D:\Ascii_Files\Envir_2060_ascii, D:\Ascii_Files\Envir_2090_ascii" samplesfile=D:\Data\RioGrande\Birds\allbirds.csv environmentallayers=D:\Ascii_Files\Envir_Curr_ascii randomseed noaskoverwrite biasfile=D:\Data\RioGrande\Birds\all_bias.asc replicates=15 nooutputgrids maximumiterations=5000 biastype=3 "applythresholdrule=equal training sensitivity and specificity" -N ai_1 -N ai_2 -N bio10_1 -N bio10_1.tif -N bio10_2 -N bio10_2.tif -N bio10_3 -N bio10_3.tif -N bio11_1 -N bio11_1.tif -N bio11_2 -N bio11_2.tif -N bio11_3 -N bio11_3.tif -N bio13_1.tif -N bio13_2.tif -N bio14_1.tif -N bio14_2.tif -N bio14_3.tif -N bio16_1.tif -N bio16_2.tif -N bio16_3.tif -N bio17_.tif -N bio17_2.tif -N bio17_3.tif -N bio17_all.tif -N bio18_1.tif -N bio18_2.tif -N bio19_1.tif -N bio19_2.tif -N bio19_3.tif -N bio2_1.tif -N bio2_2.tif -N bio3_1.tif -N bio3_2.tif -N bio5_1.tif -N bio5_2.tif -N bio6_1.tif -N bio6_2.tif -N bio6_3.tif -N bio7_1.tif -N bio7_2.tif -N bio7_3.tif -N etan_1.tif -N etan_2.tif -N etan_3.tif -N etdjf_1.tif -N etdjf_2.tif -N etdjf_3.tif -N etjas_1.tif -N etjas_2.tif -N etjas_3.tif -N etjfm_1.tif -N etjfm_2.tif -N etjfm_3.tif -N etjja_1.tif -N etjja_2.tif -N etjja_3.tif -N etjja_all.tif -N petnvan_1.tif -N petnvan_2.tif -N petnvdjf_1.tif -N petnvdjf_2.tif -N petnvdjf_3.tif -N petnvjas_1.tif -N petnvjas_2.tif -N petnvjas_3.tif -N petnvjfm_1.tif -N petnvjfm_2.tif -N petnvjfm_3.tif -N petnvjja_1.tif -N petnvjja_2.tif -N petnvjja_3.tif -N petwan_1.tif -N petwan_2.tif -N petwan_3.tif -N petwdjf_1.tif -N petwdjf_2.tif -N petwdjf_3.tif -N petwjas_1.tif -N petwjas_2.tif -N petwjas_3.tif -N petwjas_all.tif -N petwjfm_1.tif -N petwjfm_2.tif -N petwjfm_3.tif -N petwjja_1.tif -N petwjja_2.tif -N petwjja_3.tif -N prcpan_1.tif -N prcpan_2.tif -N prcpan_all.tif -N runoffan_1.tif -N runoffan_2.tif -N runoffan_3.tif -N runoffdjf_1.tif -N runoffdjf_2.tif -N runoffdjf_3.tif -N runoffjas_1.tif -N runoffjas_2.tif -N runoffjas_3.tif -N runoffjas_all.tif -N runoffjfm_1.tif -N runoffjfm_2.tif -N runoffjfm_3.tif -N runoffjja_1.tif -N runoffjja_2.tif -N runoffjja_3.tif -N smcan_1.tif -N smcan_2.tif -N smcan_3.tif -N smcdjf_1.tif -N smcdjf_2.tif -N smcdjf_3.tif -N smcjas_1.tif -N smcjas_2.tif -N smcjas_3.tif -N smcjfm_1.tif -N smcjfm_2.tif -N smcjfm_3.tif -N smcjja_1.tif -N smcjja_2.tif -N smcjja_3.tif -N swean_1.tif -N swean_2.tif -N swean_3.tif -N swedjf_1.tif -N swedjf_2.tif -N swedjf_3.tif -N swejfm_1.tif -N swejfm_2.tif -N swejfm_3.tif -N tave_1.tif -N tave_2.tif -N tave_3.tif -N tmax_1.tif -N tmax_2.tif -N tmax_3.tif -N tmin_1.tif -N tmin_2.tif -N tmin_3.tif -t biome
