# Supplementary material for: Implications of Climate Change for Bird Conservation in the Southwestern U.S. under Three Alternative Futures
Source: PLoS One. 2015 Dec 23;10(12):e0144089. doi: 10.1371/journal.pone.0144089 (PMC4689447; doi:10.1371/journal.pone.0144089)

S1 File. Predicted change in suitable habitat for three bird species.

The following in supplied as supplemental information for the manuscript “Implications of climate change for bird conservation in the Southwestern U.S. under three alternative futures” by Friggens and Finch.

Figure A. Predicted change in suitable habitat for the Lucy’s warbler. Suitable habitat is shown as predicted by three models run under GCMs, CGCM3.1, GFDL-CM2, and HadCM3.1. Current estimates were based on average climate values taken for 1970-2013. Future time periods, 2030, 2060, and 2090 used 20 year averages.

Figure B. Predicted change in suitable habitat for the Southwestern willow flycatcher. Suitable habitat is shown as predicted by three models run under GCMs, CGCM3.1, GFDL-CM2, and HadCM3.1. Current estimates were based on average climate values taken for 1970- 2013. Future time periods, 2030, 2060, and 2090 used 20 year averages.

Figure C. Predicted change in suitable habitat for the western yellow-billed cuckoo. Suitable habitat is shown as predicted by three models run under GCMs, CGCM3.1, GFDL-CM2, and HadCM3.1. Current estimates were based on average climate values taken for 1970-2013. Future time periods, 2030, 2060, and 2090 used 20 year averages.

Lucy's Warbler

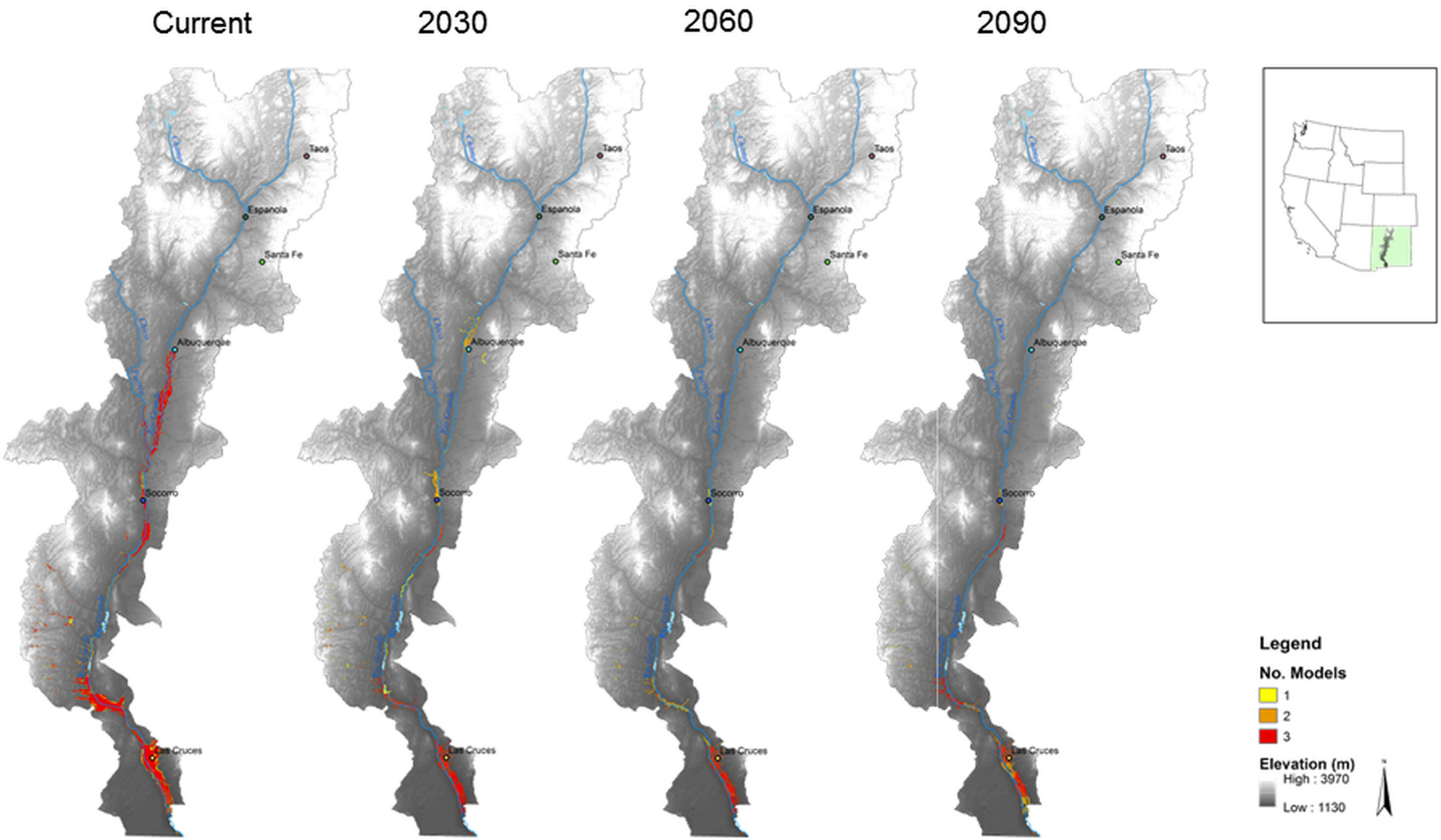

Southwestern Willow Flycatcher

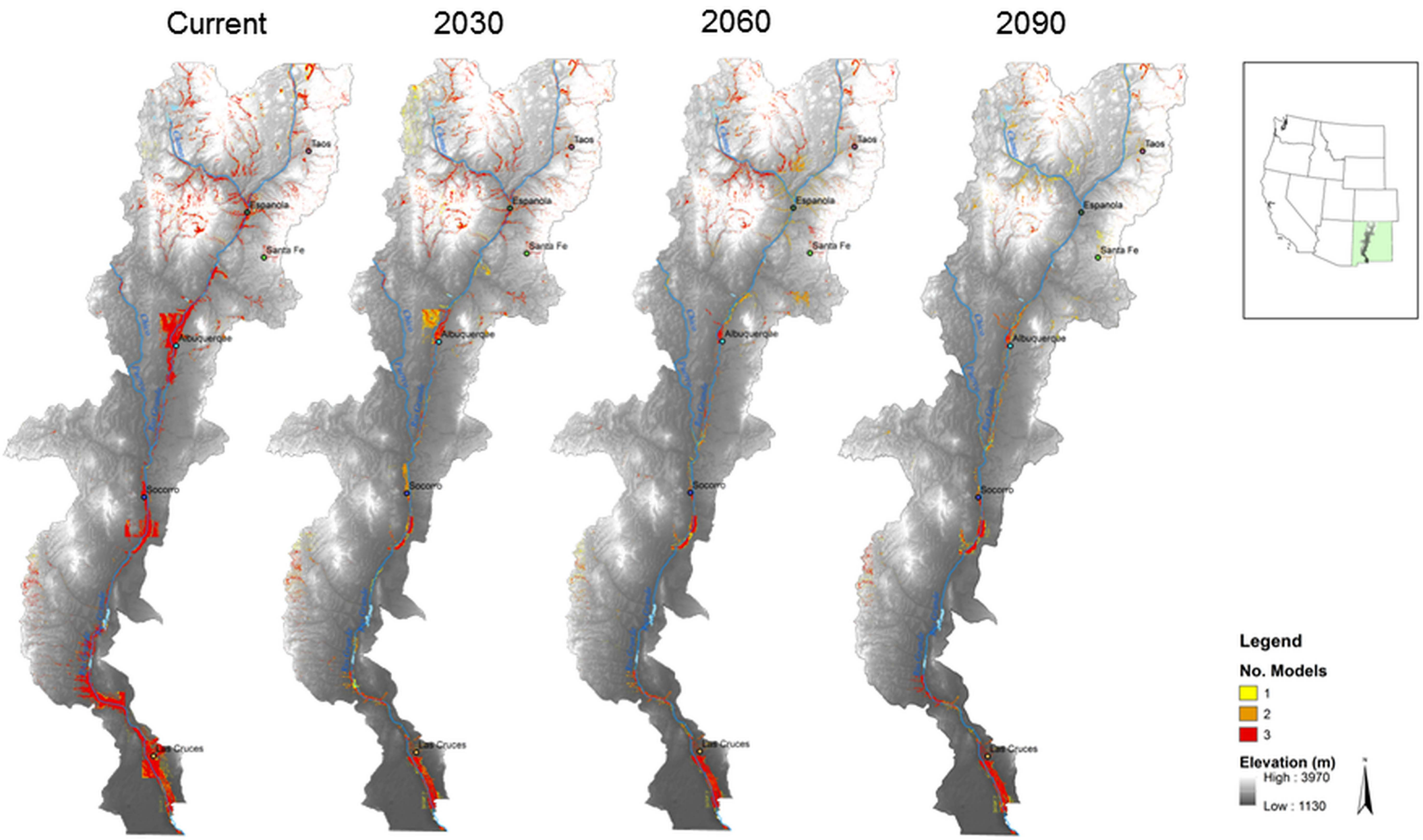

Western Yellow-billed Cuckoo

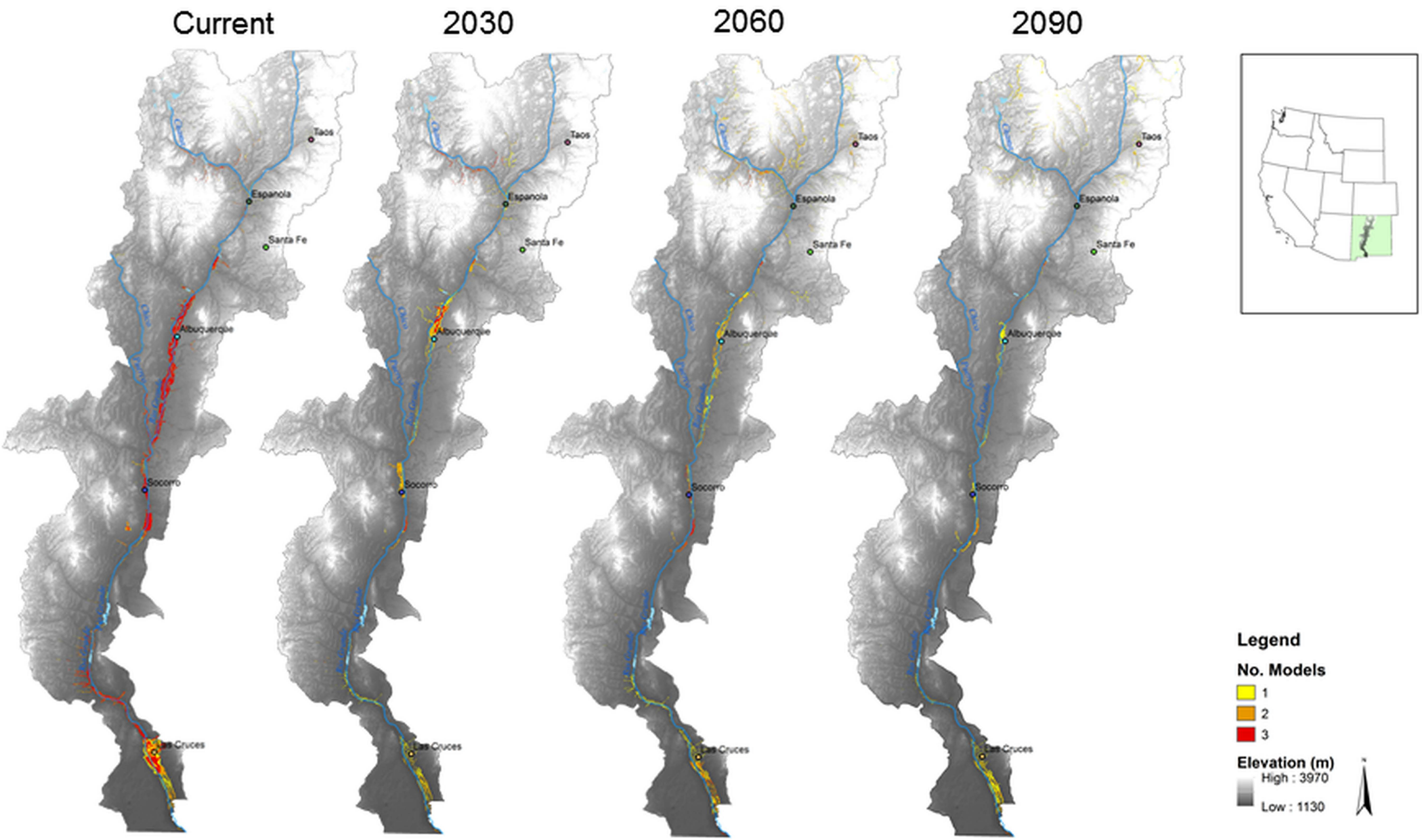

Supplement: S1 File — (PDF) [file pone.0144089.s004.pdf]
